# In-depth transcriptome reveals the potential biotechnological application of *Bothrops jararaca* venom gland

**DOI:** 10.1590/1678-9199-JVATITD-2019-0058

**Published:** 2020-10-21

**Authors:** Leandro de Mattos Pereira, Elisa Alves Messias, Bruna Pereira Sorroche, Angela das Neves Oliveira, Lidia Maria Rebolho Batista Arantes, Ana Carolina de Carvalho, Anita Mitico Tanaka-Azevedo, Kathleen Fernandes Grego, André Lopes Carvalho, Matias Eliseo Melendez

**Affiliations:** 1 Molecular Oncology Research Center, Barretos Cancer Hospital, Barretos, SP, Brazil.; 2 Laboratory of Molecular Microbial Ecology, Federal University of Rio de Janeiro (UFRJ), Rio de Janeiro, RJ, Brazil.; 3 Laboratory of Herpetology, Butantan Institute, São Paulo, SP, Brazil.; 4 Pelé Little Prince Research Institute, Curitiba, PR, Brazil.; 5 Little Prince College, Curitiba, PR, Brazil.

**Keywords:** Bothrops jararaca, Venom gland, Transcriptome, Biotechnological application, Stonustoxin, Verrucotoxin

## Abstract

**Background:**

Lack of complete genomic data of *Bothrops jararaca* impedes molecular biology research focusing on biotechnological applications of venom gland components. Identification of full-length coding regions of genes is crucial for the correct molecular cloning design.

**Methods:**

RNA was extracted from the venom gland of one adult female specimen of *Bothrops jararaca*. Deep sequencing of the mRNA library was performed using Illumina NextSeq 500 platform. *De novo* assembly of *B. jararaca* transcriptome was done using Trinity. Annotation was performed using Blast2GO. All predicted proteins after clustering step were blasted against non-redundant protein database of NCBI using BLASTP. Metabolic pathways present in the transcriptome were annotated using the KAAS-KEGG Automatic Annotation Server. Toxins were identified in the *B. jararaca* predicted proteome using BLASTP against all protein sequences obtained from Animal Toxin Annotation Project from Uniprot KB/Swiss-Pro database. Figures and data visualization were performed using ggplot2 package in R language environment.

**Results:**

We described the in-depth transcriptome analysis of *B. jararaca* venom gland, in which 76,765 *de novo* assembled isoforms, 96,044 transcribed genes and 41,196 unique proteins were identified. The most abundant transcript was the zinc metalloproteinase-disintegrin-like jararhagin. Moreover, we identified 78 distinct functional classes of proteins, including toxins, inhibitors and tumor suppressors. Other venom proteins identified were the hemolytic lethal factors stonustoxin and verrucotoxin.

**Conclusion:**

It is believed that the application of deep sequencing to the analysis of snake venom transcriptomes may represent invaluable insight on their biotechnological potential focusing on candidate molecules.

## Background

Animal venom is composed of a complex and potent mixture of molecules with different physiological activities, ranging from moderate effects, such as allergic reactions and dermatitis [1, 2], to more severe effects like hemorrhage, intravascular coagulation, necrosis, respiratory arrest and death [3-5]. These bioactive compounds are low explored bioresources for the development of new therapeutic drugs for different type of diseases and conditions [6-8].

Venomous snakes Snake venom is a promising source of therapeutic proteins, as these venoms comprise more than 95% of the dry weight of a snake’s venom is composed of peptides/proteins [9]. These venoms comprise wide variety of enzymes such as phospholipases A_2_, proteases (metal and serine), L-amino acid oxidases, and esterases, as well as many other non-enzymatic proteins and peptides, which have several biochemical and pharmaceutical properties [10-13]. 

One venomous snake of particular interest is *Bothrops jararaca*, which is endemic to the tropical/semitropical forest habitats of southeastern Brazil, northeastern Paraguay, and northern Argentina [14]. In recent years, high throughput technology has been implemented more often in snake venom analyses, allowing better understanding of proteomics and transcriptomics of venom, which has exposed its complexity [15, 16]. High throughput transcriptome analysis allows the identification of complete transcripts expressed in the snake venom gland [17, 18]. Moreover, molecular cloning of biotechnological proteins of *B. jararaca* requires the complete characterization of the full-length coding regions of interesting transcripts. The venom produced by *B. jararaca* has previously used to isolate bradykinin-potentiating factor (BPF), which was the basis for the creation of the antihypertensive agent captopril, and the oral anticoagulant Exanta, also known as ximelagatran [19, 20].

In this study, we generated an RNA-Seq transcriptome, performed *de novo* assembly and annotated the sequences from the venom gland of *Bothrops jararaca.* This work expands the current knowledge of the biotechnological potential of the venom gland of *Bothrops jararaca.* Furthermore, results may also be used for the discovery of novel candidates for cancer treatment, hypertension, inflammatory response, virus infection, and other human diseases, as well as the development of effective treatments of poisoning from *Bothrops jararaca* bite.

## Materials and Methods

### RNA extraction from venom gland

All experiments were performed in accordance with Brazil’s National Council for the Control of Animal Experimentation (CONCEA) guidelines and were authorized by the Ethic Committee on Animal Use of the Butantan Institute, under protocol n. 4390280116. Venom glands were removed 3 days after venom milking, when RNA transcription is at its highest level [21], from a chemically euthanized female adult *B. jararaca* under captivity (Herpetology Laboratory of the Butantan Institute) and immediately stored at -80 °C. Venom glands were then transported in dry ice to the Barretos Cancer Hospital for molecular biology analysis. Tissue samples from the venom glands (40 mg) were then disrupted and homogenized in a Precellys 24 homogenizer (Bertin Technologies) at 4,500 rpm, while being left to cool down on ice between the 3 repeated 20 second-cycles. Total RNA was isolated using RNeasy Mini kit (Qiagen) and quantified by spectrophotometry. In order to avoid cross-contamination during RNA extraction, we performed the RNA extraction in the Molecular Oncology Research Center of the Barretos Cancer Hospital, where no other source of nucleic acid of reptilian origin was ever extracted.

### Transcriptome sequencing and quality analysis

Deep sequencing of the mRNA library from *B. jararaca* was done using the Illumina NextSeq 500 platform (76 bp single-end) and the NextSeq 500/550 Mid Output v2 kit (150 cycles). Libraries were constructed using TruSeq Stranded mRNA LT Sample Prep Kits (Illumina), following the TruSeq Stranded mRNA Sample Prep HS protocol (Illumina). In order to avoid cross-contamination with transcripts of other related species, the venom gland sequenced for this work was the only reptilian sample in the Illumina chip. The quality analysis of the sequenced data was evaluated with the FASTQC software [22]. Reads were filtered with Trimmomatic v0.36 [23] using a 4-base sliding window. Leading or trailing bases with average Phred quality score lower than 20 were removed, along with adapters and reads with a length less than 50 base pairs (bp).

### ***De novo* assembly and quality analysis**

*De novo* assembly of the *B. jararaca* transcriptome was performed using Trinity [18] with default k-mer size of 25. The statistics of the Trinity Assembly like the Nx statistics (eg. the contig N50 value), total trinity genes, and total of trinity transcripts were obtained with perl script ‘TrinityStats.pl’ of the Trinity toolkit [24]. Gene open reading frames (ORFs) or protein coding regions within the transcripts were predicted with the TransDecoder program [24]. CD-HIT-EST version 4.6.1 [25] was subsequently used for clustering predicted proteins with 100% of sequence identity and 100% alignment coverage. For analysis of transcript abundance, the sequenced paired-end reads were realigned with the assembled transcripts for quantification by the RSEM (RNA-Seq by Expectation Maximization) software [26] using the Trinity script ‘align_and_estimate_abundance.pl’ present in the Trinity toolkit [18]. The transcriptome completeness and contigruity of *B. jararaca* was assessed by comparing the assembly transcripts to benchmarking sets of the universal single-copy (BUSCO) of Eukaryota, Metazoa, and Vertebrata using BUSCO *v3*, based on evolutionarily informed expectations of gene content from near-universal single-copy orthologs selected with the database OrthoDB v9 [27, 28] . Full-length transcript or near full length transcript of *B. jararaca* was identified using BLASTX (BLAST+ v2.2) by alignment against the predicted proteome of *Anolis carolinensis* (GCA_000090745.1, GenBank ID), *Ophiophagus Hannah* (GCA_000516915.1, GenBank ID), and *Python bivittatus (*GCA_000186305.2, GenBank ID), which were obtained from GENOME (https://www.ncbi.nlm.nih.gov/genome/) and UniProtKB/Swiss-Prot release 2019_04 May-08, 2019 using an E-value cut-off set to 1 x 10^−20^.

Full-length transcript or near full-length transcript are defined as transcripts (*query*) similar to proteins already annotated in reference genomes with high quality standards of genome completeness and functional annotation. The alignment between the transcripts (query) and the reference sequence protein obtained in the the BLASTX covered across more than 80-90% of the transcript length. The resulting table with full-length transcript or near full-length transcript from BLASTX was obtained with the perl script ‘analyze_BLASTPlus_topHit_coverage.pl’ from Trinity toolkit [24]. For each BLAST hit in the target database of the protein, the best matching Trinity transcript was selected, and the percent of the BLAST hit’s length covered by the Trinity transcript was identified. 

### Functional annotation of transcriptome

Annotation was performed using Blast2GO version 3.2 [29]. All predicted proteins after clustering step were blasted against the non-redundant protein database (NR) of NCBI (ftp://ftp.ncbi.nih.gov/blast/db/; 29-02-2015) using BLASTP with an E-value cut-off set to 1 x 10^−6^. The metabolic pathways present in the transcriptome were annotated using the KAAS - KEGG Automatic Annotation Server [30], which provides functional annotation of genes via BLAST using the method BBH (bi-directional best hit), against the manually curated KEGG GENES database. With the KEGG Orthology groups (KOs) identified via KAAS [30], the complete functional modules of the metabolic pathways present in the *B. jararaca* transcriptome were reconstructed using the tool KEGG Mapper tools [31].

### Toxin identification 

The toxins were identified in the *B. jararaca* predicted proteome using BLASTP with an E-value cut-off set to 1 x 10^−6^ and using the protein sequences obtained from the Animal Toxin Annotation Project (version 31/10/2018) from Uniprot KB/Swiss-Pro database as a reference [32]. The BLASTP annotation results obtained of alignment against the Animal Toxin Annotation Project database and with same annotation description obtained from the BLASTP against the NR database were considered toxins. In addition, all inhibitors or tumor suppressor proteins were identified in the annotation from the BLASTP results against the NR database using the search terms: inhibitors AND tumor suppressor and later these results were manually checked. For identification of the full length transcript of *B. jararaca* that aligns to the Animal Toxin Annotation Project from Uniprot KB/Swiss-Pro database [32], we processed the BLASTx hits (E-value cut-off set to 1 x 10^−20^) using the ‘analyze_BLASTPlus_topHit_coverage.pl’ script from the Trinity package (http://trinityrnaseq.sourceforge.net/), as described above.

### Phylogenetics analysis of stonustoxin and verrucotoxin

The sequences of stonustoxin and verrucotoxin of *B. jararaca* identified were aligned with BLASTP program (https://blast.ncbi.nlm.nih.gov/Blast.cgi?PAGE=Proteins) and homologous sequences recovered with an E-value cut-off above of 1 x 10^−20^ were used for Phylogenetic analyses reconstruction. Phylogenetic analyses were performed in the platform NGPhylogeny.fr [33] using the method Advanced PhyML + SMS. In this mode, the program of alignment is the MAFFT [34]. The parameters of matrix of distance chosen in the program MA FFT was BLOSUM62. After the alignment, the sequences were curated with BMGE [35] for gaps remotion and to keep the informative sites. Then, the Smart Model Selection in PhyML program assesses and chooses the best evolutionary model for phylogenetic analyzes. For phylogenetic analysis was used the program PhyML [36] with maximum likelihood method and the inference of Branch support in the tree as done with Bootstrap (FBP + TBE) using 1000 bootstrap replicates. All other parameters for program BMGE and Newick tree format Display were keept as default. All steps were done in the program NGPhylogeny.fr (https://ngphylogeny.fr/).

### Data visualization

Figures and data visualization were performed using the ggplot2 package in R [37], through the R software [38].

## Results

### **Transcriptome sequencing, *de novo* assembly and full-length transcript analysis**

Deep sequencing of the venom gland transcriptome of *B. jararaca* was performed using a NextSeq 500 (Illumina), generating 67,551,639 unpaired reads. Transcriptome raw data quality showed a Phred quality score (per base sequence quality average) higher than 30 (Additional file 1). *De novo* assembly of all reads resulted in a total assembly of 64,853,458 bp representing 76,765 genes (N50 length of 1104 bp) and 96,044 transcripts (N50 length of 1,104 bp), with a mean contig length of 675.25 bp and a GC percentage of 43.56% (Table 1).

The completeness of transcriptome assessment performed by the Benchmarking Universal Single-Copy Orthologs (BUSCO; version 3.0) showed that 85.8% of the 303 core eukaryotic genes and 88.7% of the 978 core metazoan genes were found in our *B. jararaca* transcriptome assembly (Table 2). Furthermore, the BUSCO analysis with 2,586 core vertebrata genes showed that 1,550 (59.9%) and 543 (21%) of the 2,586 expected vertebrata genes were identified as complete or fragmented, respectively, while 493 (19.1%) genes were considered missing. 

The quantity of full-length transcripts, or near full-length transcripts in *B. jararaca,* was determined by the number of predicted sequences of Reptiles and UniprotKB/Swiss-Prot databases (Table 3). Among the 96,044 transcripts of *B. jararaca*, 2,076 (2.16%) matched near full length with coding sequences of *Anolis carolinensis*, 4,555 (4.74%) with *Ophiophagus hannah*, 6,707 (6.98%) with *Python bivittatus*, and 5,472 (5.69%) with UniprotKB/Swiss-Prot (Table 3 and Additional file 2). Thus, a total of 6,835 full length or near full-length transcripts were identified using the concatenated predicted proteome of all three reptiles cited above.

**Table 1.** Statistics of Trinity *de novo* assembly.

Global Trinity Stats	
Total trinity 'genes' counts	76 765
Total trinity transcripts counts	96 044
Percent GC (%)	43.56
**Stats based on *all* transcript contigs**	
Contig N10	3 774
Contig N20	2 674
Contig N30	2 012
Contig N40	1 512
Contig N50	1 104
Median contig length	362
Average contig (%)	675.25
Total assembled bases	64 853 458
**Stats based only on *longest isoform* per *'gene'***	
Contig N10	3 302
Contig N20	2 264
Contig N30	1 642
Contig N40	1 179
Contig N50	828
Median contig length	335
Average contig	585.97
Total assembled bases	44 982 090

**Table 2.** Summary of transcriptome completeness assessment by BUSCO notation.

Eukaryotic genes	
Complete and single-copy BUSCOs	222 (73.3%)
Complete and duplicated BUSCOs	38 (12.5%)
Fragmented BUSCOs	35 (11.6%)
Missing BUSCOs	8 (2.6%)
**Total BUSCO groups searched**	**303 (100%)**
**Metazoan genes**	
Complete and single-copy BUSCOs	683 (69.8%)
Complete and duplicated BUSCOs	185 (18.9%)
Fragmented BUSCOs	82 (8.4%)
Missing BUSCOs	28 (2.9%)
**Total BUSCO groups searched**	**978 (100%)**

**Table 3.** Full-length transcript reconstruction analysis of *B. jararaca* venom gland transcriptome in relation to *Anolis carolinensis*, *Ophiophagus hannah, Python bivittatus* and UniprotKB/Swiss-Prot.

*Anolis carolinensis*			*Ophiophagus hannah*			*Python bivittatus*			UniprotKB/Swiss-Prot		
Pct_cov_Hit (%)	PC	PCS	Pct_cov_Hit (%)	PC	PCS	Pct_cov_Hit (%)	PC	PCS	Pct_cov_Hit (%)	PC	PCS
100	1438	1438	100	3069	3069	100	4974	4974	100	3739	3739
90	367	1805	90	834	3903	90	928	5902	90	1034	4773
80	271	2076	80	652	4555	80	805	6707	80	699	5472

Cumulative number of protein of the *A. carolinensis*, *O. hannah*, *Python bivittatus* and UniprotKB-Swiss-Prot databases recovery by BLASTX that aligned by at least one transcript in the assembly *B. jararaca* transcriptome across at 80-100 percentage (%) of coverage. The transcripts identified in *B. jararaca* were annotated as full-length transcripts if they match a protein in the reference proteome database at E-value threshold of 1e^-20^. Pct_cov_Hit: percentage of coverage of top matching hits of reference proteome that align across more than X% (80-100) with a transcript of *B. jararaca*. PC: protein counts of target reference proteome that aligned by at least one transcript of *B. jararaca*. PCS: protein count sum of reference proteins of reference proteome that aligned at X% (80-100) coverage by at least one transcript of *B. jararaca*.

### Functional annotation

**Figure 1.**
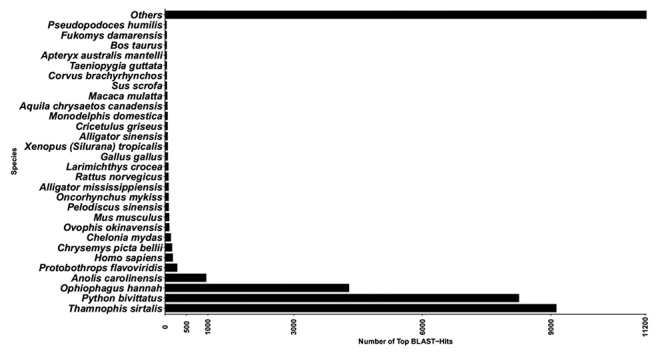
Top BLAST hit distribution of predicted proteins from of *Bothrops jararaca* venom gland transcriptome. Recovery by Blast2GO with similarity filter parameter of 55%, E-Value-Hit-Filter: 10^-6^.

From the 96,044 transcripts identified, 49,345 open reading frames (ORF) were predicted using TransDecoder [10] with *ab initio* model. In this analysis, only predicted ORFs that were at least 60 amino acids long were retained. The clustering steps of predicted proteins in the CD-HIT program (100% amino acid identity) resulted in a set of 41,916 non-redundant sequences. Functional annotations of these coding regions were inferred using BLASTP against the NR database in NCBI. Additional annotation was performed using InterProScan [39] and Gene Ontology [40] using the program Blast2GO [29]. Thus, 78.81% of unique predicted proteins (33,034) were annotated, while 8,162 (19.47%) transcripts did not have similarity against any proteins in the NR database (Additional file 3). The E-value distribution of hits obtained against NR, the average number of hits per sequence and the High Scoring Segment Pair/Coverage distribution are shown in Additional files 4, Additional file 5 and Additional file 6, respectively. Among the 33,034 annotated sequences, 22,933 (54,7%) best hits were matched in BLAST Top-hits against predicted proteins of the species *Thamnophis sirtalis, Python bivittatus, Ophiophagus hannah, Anolis carolinensis*, and *Protobothrops flavoviridis* (Figure 1). The other 1,858 sequences had best hits against other organisms, such as reptiles, humans, mammals, fishes, bacteria, virus, fungi, and others (Additional file 7). 

### Gene ontology mapping with Blast2GO

**Figure 2.**
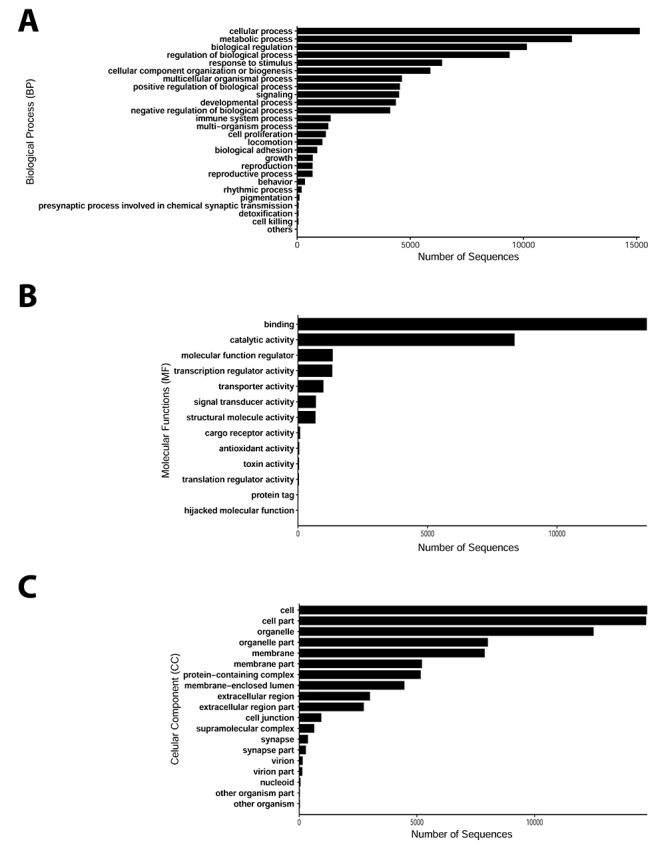
Gene Ontology category classification at level 2 and functional distribution of the transcriptome of *B. jararaca* performed by Blast2GO. The predicted proteins were functionally mapped according to the three major classifications of Gene Ontology: **(A)** biological process (BP), **(B)** molecular function (MF) and **(C)** cellular component (CC). They wereannotated by setting the following parameters - E-Value-Hit-Filter: 10^-6^ and others parameters default.

The predicted proteins identified in *B. jararaca* venom gland were mapped into putative functional group-based Gene Ontology (GO) terms assigned using NR derived BLAST hits and InterProScan using Blast2GO, categorized into three ontologies: biological processes (BP), cellular components (CC) and molecular functions (MF) (Figure 2). 

### Enzymes and metabolic pathways identification 

We identified 4,203 predicted proteins in the KO (orthologous groups) of the signal transduction metabolic pathway (Additional file 8) through the orthologous assignment KAAS - KEGG server [30]. Using 91 modules of the KEGG Reconstruct Module [41] tools, predicted proteins of the Proteasome 20S core particle (M00340), Proteasome 19S regulatory particle (PA700) (M00341), Ski complex (M00392), DNA polymerase delta complex (M00262), SCF-BTRC complex (M00380), SCF-SKP2 complex (M00381), Cul4-DDB1-DDB2 complex (M00385), and ECS complex (M00388) were identified (Additional file 9). A total of 389 metabolic pathways were annotated as having at least 1 predicted protein of *B. jararaca* described in the KEGG among the metabolic pathways identified (Additional file 10). 

### Functional domains identified 

**Figure 3.**
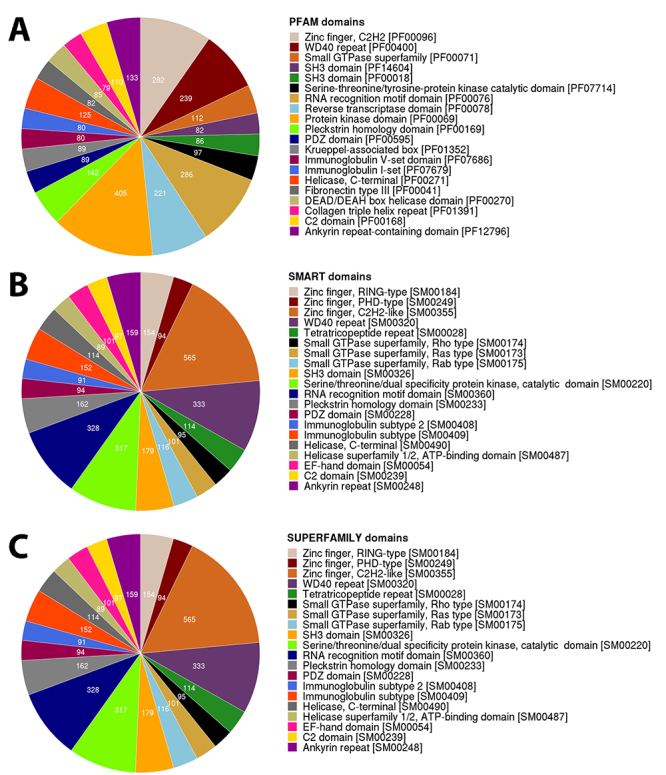
Functional domain annotation of predicted proteins of *B. jararaca* transcriptome with InterProScan: **(A)** PFAM, **(B)** SMART and **(C)** SUPERFAMILY databases.

All predicted proteins obtained from the transcript ORFs were searched for functional domains signatures present in the Smart [42], PFAM [43], and Superfamily [44] thought of the profile hidden Markov models with InterProScan [39]. Overall, a total of 20,605 predicted proteins were categorized into 3,992 domain/family signatures with PFAM (Figure 3).

### **Transcript abundance in *B. jararaca***

The top 20 genes and alternatives isoforms codified with higher expression inference of gene abundance in transcripts per million (TPM) determined by RSEM software [20] were represented by zinc metalloproteinase-disintegrin-like jararhagin, natriuretic peptide, metalloprotease, C-type lectin 8a, snake venom serine protease HS114, proteasome 26S subunit, non-ATPase 14, serine endopeptidase, metalloproteinase type II 4, acidic secretory phospholipase A2 sPLA2-II, serine proteinase 20a, metalloprotease BOJUMET II, putative disulfide-isomerase, sphingomyelin phosphodiesterase-like, metalloproteinase type III 8, snake venom serine protease homolog, L-amino-acid oxidase, and snake venom vascular endothelial growth factor toxin (Additional file 11).

### Toxins, inhibitors and tumor suppressors 

**Figure 4.**
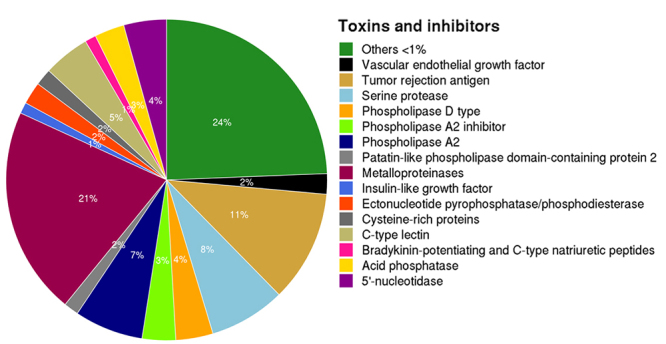
Functional class annotation of toxins and accessory family proteins identified in *B. jararaca* venom using Animal Toxin Annotation Project as reference.

Known animal toxins encoded in the *Bothrops jararaca* transcriptome, using all of the sequences from the Animal Toxin Annotation Project [45] as a reference were also searched. The 831 hits obtained from this approach (Additional file 12) were further validated in our BLASTP annotation against the NCBI non-redundant (NR) database, confirming a same description for a total of 525 coding sequences in both databases (Additional file 13). This set encoded 83 toxin and inhibitors related functional classes (Additional file 14), the more abundant (> 1%) are showed in the Figure 4. Among the less abundant toxins identified, minor toxins, such as venom factor (n = 4), L-amino acid oxidase (n = 4), phospholipase B (n = 4), Mannan-binding lectin serine protease 1 (n = 1), Mannan-binding lectin serine protease 2 (n = 3), verrucotoxin (n = 3), translationally-controlled tumor protein (n = 3), phospholipase A_1_ (n = 3), nerve growth factor (n = 2) and stonustoxin subunit alpha (n = 2) were also found (Additional file 14). 

Moreover, several inhibitors were also found, such as phosphatase inhibitor 2 (n = 1), kunitz-type protease inhibitor 1 (n = 1), kunitz-type protease inhibitor 2 (n = 1), kunitz-type protease inhibitor 4(n = 1) and protease inhibitor 3-like (n = 1) (Additional file 14). 

In addition, the 107 full-length transcripts or nearly full-length transcripts of *B. jararaca* encoding toxins were identified by BLASTX recovery against the Animal Toxin Annotation Project (version 31/10/2018) from Uniprot KB/Swiss-Pro database and with the same annotation obtained when NCBI non-redundant database was used as reference. Among these, we annotated transcripts for the vascular endothelial growth factor, acidic phospholipase A_2_, phospholipase B, zinc metalloproteinase-disintegrin-like, venom nerve growth factor, among others (Additional file 15).

### Phylogenetic analysis of stonustoxin and verrucotoxin

**Figure 5.**
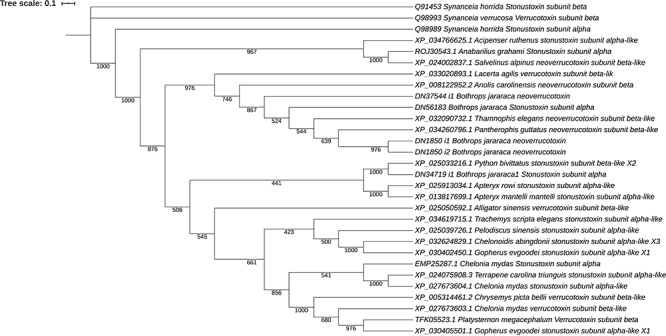
Phylogenetic tree of stonustoxin and verrucotoxin proteins.

We identified two genes (DN34719_i1, DN56183_i1) that codified similar (41.1%, using EMBOSS water local alignment program) sequences to stonustoxin subunit alpha; and two genes codifying (DN37544_i1 and DN1850) similar (41.8%, using EMBOSS water local alignment program) sequences to verrucotoxin subunit beta-like in the annotation recovered by BLASTP against the Nr database (Figure 5 and Additional file 16). Subsequently, the homologous sequences for these proteins were recovery by BLASTP against NR database of NCBI using an E-value cut-off set to 10^−20^. The bootstrapped analysis (1000:100%) of the phylogenetic tree revealed that *Acipenser ruthenus, Anabarilius grahami, Salvelinus alpinus, Lacerta agilis, Anolis carolinensis, Bothrops jararaca* (DN37544_i1, DN56183), *Python bivittatus, Bothrops jararaca* (DN34719_1), *Apteryx rowi*, *Alligator sinensis*, *Trachemys scripta elegans, Pelodiscus sinensis, Chelonoidis abingdonii, Gopherus evgoodei, Chelonia mydas, Terrapene carolina triunguis, Chrysemys picta bellii* and *Platysternon megacephalum* have a common origin with stonustoxin subunit alpha Q98989. The sonustoxin of *Synanceia horrida* 13279 have a functional domain SPRY (IPR003877, access identifier in Interproscan), but the sequences of snakes, reptiles and turtles have a functional domain GTP binding (PF01926: access identifier in Pfam) called MMR_HSR1 (PF01926) and one domain fibronectin type 3 (SM000060: access identifier in Smart). The *B. jararaca* stonustoxin have one domain MMR_HSR1 and one domain fibronectin type 3. The sequences annotated as verrucotoxin subunit beta-like in *B. jararaca* have a domain fibronectin type 3. The verrucotoxin of *Synanceia horrida* 13279 also has a domain SPRY as well as stonustoxin subunit beta. 

## Discussion

More than 90% of the dry weight of snake venom is represented by peptides and proteins, potentially representing a natural arsenal of biotechnological relevant proteins [13, 46]. Traditional snake venom protein purification involves the extraction of crude venom, followed by purification via physical or chemical methods, such as high-performance liquid chromatography purification, which produces small amounts of purified products, but not all the products are purified. While this procedure is well standardized, the amount of peptides/proteins purified is not enough for pre-clinical or clinical studies. Less abundant snake venom protein classes, well reviewed by Boldrini-França et al. [47]. With the intent to further increase the amount of known proteins, we described here the transcriptome from the *B. jararaca* venom gland and its annotations. Data presented in this study can be used to produce proteins in heterologous expression systems, such Eukaryotic, bacteria or yeast cell cultures. Recent advances in molecular biology and genomics has made possible the analysis of whole transcriptomes from different tissue sources, being found five publications describing transcriptome analyses of *B. jararaca* [14, 46-51].

An initial study of the transcriptome of an adult specimen of *B. pauloensis* caught in São Paulo State (Brazil) was described by Rodrigues and collaborators in 2012 [52], using an Expressed Sequence Tag (EST) singleton library of 668 EST sequences. Moreover, a transcriptome analysis using deep sequencing approach was recently published by Gonçalves-Machado et al. [14], in which they compared the transcriptomes of two *B. jararaca* populations from the Brazilian Southern (S) and Southeastern (SE) Atlantic rainforest. This transcriptome analysis was performed with 205,449 (SE)/281,569 (S) reads and generated 14,246 (SE)/12,240 (S) contigs, describing 15 (SE)/16 (S) different family proteins [14]. Similarly, Junqueira-de-Azevedo et al. [49] described the transcriptome of the venom gland of an adult *B. jararaca*, generating 116,236 reads. In the present work, we describe the transcriptome analysis of *B. jararaca*, with 67,551,639 unpaired reads, which was assembled using Trinity. This analysis generated 76,765 genes with an N50 length of 828 bp along with 96,044 transcripts with an N50 length of 1,104 bp and a total assembly of 64,853,458 bp with 41,916 unique predicted proteins. To date, an approximate N50 value of 1,431 bp and average contig length of 894 bp was reported for the transcriptome of the snake *Bothrops moojeni* [53], but other works [8, 10, 41-44] did not described this value.

Our transcriptome of the vertebrata core genes was more complete than any previously sequenced transcriptome of other snakes [54]. However, it is expected that not all genes are expressed in the venom gland. BUSCO recovery tends to be highest when the entire organism and/or multiple developmental stages or the same organism is used to generate the assemblies, compared to those assembled from a select number of tissues [55]. The BUSCO results of our assembly generated here was comparable to the other transcriptomes reported, where recovery varied between 68 and 95% [56-59].

Our predicted proteome had a greater number of predicted proteins with high identity or best hit obtained from BLASTP against the proteomes available for *Thamnophis sirtalis, Ophiophagus hannah, Python bivittatus, Anolis carolinensis*, *and Protobothrops flavoviridis* (Figure 1) (Additional file 7). We also identified and annotated a higher number of predicted proteins than these previously deposited genomes, which could make the current transcriptome analysis a reference or complement for the functional knowledge of the coding proteins present in other snakes or reptiles.

We identified 107 full-length or near full-length transcripts (80 -100% coverage lenght of query in relation to reference toxins) which were defined as toxins or inhibitors (Additional file 13). These transcripts included hemolytic lethal factor stonustoxin [60, 61] and verrucotoxin [62], which were identified for the first time in snakes. We identified MASP1 and MASP2 that are of alternative pathways of complement activation, involved in the activation of factor D [63], complement factor I, complement C1r subcomponent and complement component C7, which possibly participate in the tissue damage in a host bitten by *B. jararaca* and may aid in the venom poisoning*.* The excessive complement activation and immune activation could exacerbate the severity of the tissue injury [64].

Several predicted toxins were identified for the first time in this transcriptome, such as veficolin-1, ryncolin-1-like, pancreatic alpha-amylase, venom allergen 3-like, stonustoxin subunit alpha and verrucotoxin (Additional file 14). Moreover, different inhibitors and potential tumor supressors were also found, not yet reported in the venom gland of *B. jararaca*, such as the relA-associated inhibitor, ribonuclease inhibitor, reversion-inducing cysteine-rich protein with Kazal motifs and Insulin-like growth factor-binding protein 6 (Additional file 14). Moreover, up to now, the cobra venom factor and the three-finger like transcripts were never described in venom glands of *B. jararaca* [61, 65, 66].

We also identified two genes coding toxins similar to stonustoxin subunit beta (DN34719_i1, TPM:105, DN56183, TPM: 17.00) and to verrucotoxin (DN37544_i1, TPM: 16, DN1850, TMP: 34), which has been studied in venom of fishes of the species *Synanceia verrucose* [67]*.* The phylogenetic analysis indicated that *B. jararaca* stonustoxin (DN34719_c0_g1) and rerrucotoxin (DN1850_c0_g1) are related to stonustoxin and verrucotoxin of *Synanceia verrucose.* Results for stonustoxin indicated that this protein diverged from an ancestral node, with a bootstrap value of 100% significance (Figure 5 and Additional file 15). However, these proteins have different functional domains predicted. Hence, it is not possible to affirm that these homologous sequences have the same function, because neofunctionalization is a common evolutionary process found after speciation in homologous or paralogous sequences.

The *Synanceia verrucose* inhabits shallow waters of the tropical or subtropical Indo-Pacific regions and are among the most venomous and dangerous fishes in the world. The purified stonefish toxins commonly present potent hemolytic activities due to its ability to form pores in the cell membrane. Also, these toxins elicit potent hypotension, inhibit neuromuscular function, and induce cardiovascular collapse in humans and native predators [54, 68]. The stonustoxin-like were also found in the transcriptome of the venom gland of annelids [69] and are found in a variety of vertebrates, including the common ostrich, platypus, tasmanian devil, and coelacanth [70].

We identified with KEGG mapper [71] one of the largest mapped/annotated pathways of the components of the human retrovirus response. Subsequently, one of the identified components of the Gene Ontology term [34] identified by Blast2Go in our analysis is the virion part, consisting of sequences coding for several endogenous retrovirus described in snakes such as *Python curtus endogenous retrovirus*, *Python molurus endogenous retrovirus*, Endogenous retrovirus group PABLB member 1, Endogenous retrovirus group K members of families 1, 8, 9, 10, 11, 18, 19 and 25, and human endogenous retroviruses (HERVs) such as HCML-ARV already isolated from blood cells of patient with chronic myeloid leukemia [72].

One important finding was the identification of Syncytin and L1-retrotransposons genes in the venom gland of *B. jararaca.* Syncytin genes are associated with placental evolution in mammals and viviparous anim als [73]. Up to now, these coding sequences were never described in transcriptomes of viviparous snakes, mainly *B. jararaca*. Only recently it was found in the *Mabuya* lizards [74].

In addition, we also identified the metabolic pathways essential for survival such as thermogenesis, catabolism and anabolism of carbons, amino acids metabolism, fatty acid metabolism, pathways involved with splicing of RNA, and processing and degradation of proteins (Additional file 11). Among the most frequent signatures of functional domains identified in our work was the kinase domain and serine-threonine/tyrosine-protein kinase catalytic domains, which are known to regulate or activate most cellular pathways. Similarly, the RNA recognition motif was also abundant and acts in the recognition of RNA and proteins known to bind single-stranded RNAs [75]. Other prominent functional domains found within the *B. jararaca* transcriptome were the zinc finger domain, which is a DNA, RNA, protein or lipid binding domain [76]; as well as metalloproteases; WD40 repeats, involved in several functions such regulation to cell cycle control and apoptosis [77]; a reverse transcriptase domain, characteristic of retroviruses involved in replication in the host; and a pleckstrin homology domain, with a role in recruiting proteins to different membranes [78]. These signatures reflected the biologic role of the proteins identified.

The additional analysis of RSEM showed that the most abundant transcripts genes belong to zinc metalloproteinase-disintegrin-like jararhagin, natriuretic peptide metalloprotease, C-type lectin 8a and L-amino acid oxidase. The distribution of these protein types is characteristic of the *Bothrops* genus, whose species produce venoms most notable for local tissue damage such as edema, hemorrhage, and necrosis, which has already been described by other researchers [60].

So far, our work represents the largest transcriptome analysis of *B. jararaca* venom gland. All raw and analyzed data are available for further studies, making possible further exploitation for biotechnological uses. 

## Conclusion

Our transcriptome analysis strategy yielded unique insights into the diversity of *B. jararaca* venom gland transcriptome toxins. The present results bring an important contribution to the development of snake venom-derived proteins as potential biotechnological sources, especially for the search and development of candidate molecules.

## Supplementary material

The following online material is available for this article:

Additional file 1. Phred quality score (Sanger encoding, Phred+33 format) obtained with FastQC program.

Additional file 2. Full-length transcript identified in *B. jararaca* transcriptome using UniprotKB Swiss database.

Additional file 3. Distribution of annotated number of sequences with BLASTP using with BLASTP using Nr database, Interproscan and Gene Ontology throught of Blast2GO.

Additional file 4. E-value distribution of all predicted proteins obtained from *Bothrops jararaca* transcriptome.

Additional file 5. BLAST hit distribution of all predicted proteins obtained from *Bothrops jararaca* transcriptome.

Additional file 6. HSP/Seq coverage distribution of all predicted proteins obtained from *Bothrops jararaca* transcriptome*.*

Additional file 7. BLAST hits against predicted proteins.

Additional file 8. KO annotation in the predicted proteome of *B. jararaca* with KAAS - KEGG server.

Additional file 9. KO annotation in the predicted proteome of *B. jararaca* with KEGG Reconstruct Module.

Additional file 10. Metabolic pathways with at least one predicted protein of *B. jararaca* described in the KEGG annotation.

Additional file 11. Top 20 more abundant transcripts and their isoforms (based on TPM values) identified in the transcriptome of *B. jararaca.*

Additional file 12. Animal toxins encoded in the *Bothrops jararaca* transcriptome identified in the Animal Toxin Annotation Project.

Additional file 13. Animal toxins encoded in the *Bothrops jararaca* transcriptome, identified in both Animal Toxin Annotation Project and NCBI non-redundant (NR) database.

Additional file 14. Toxins and inhibitors identified in the predicted proteome of *B. jararaca.*

Additional file 15. Function of toxins and accessory proteins identified in the predicted proteome of *B. jararaca.*

Additional file 16. Alignment of proteins of stonustoxin and verrucotoxin subunit beta.
