# Supplementary material for: In-depth transcriptome reveals the potential biotechnological application of Bothrops jararaca venom gland
Source: J Venom Anim Toxins Incl Trop Dis. 2020 Oct 21;26:e20190058. doi: 10.1590/1678-9199-JVATITD-2019-0058 (PMC7579844; doi:10.1590/1678-9199-JVATITD-2019-0058)
Supplement: Additional file 7. [file 1678-9199-jvatitd-26-e20190058-s7.pdf]

J Venom Anim Toxins incl Trop Dis

https://doi.org/10.1590/1678-9199-JVATITD-2019-0058

Supplementary Material to "In-depth transcriptome reveals the potential biotechnological application of *Bothrops jararaca* venom gland"

Additional file 7. BLAST hits against predicted proteins.

Species	#BLAST Top-Hits	Percentage
Thamnophis sirtalis	9130	25.345%
Python bivittatus	8258	22.924%
Ophiophagus hannah	4295	11.923%
Anolis carolinensis	964	2.676%
Protobothrops flavoviridis	286	0.794%
Homo sapiens	184	0.511%
Chrysemys picta bellii	166	0.461%
Chelonia mydas	138	0.383%
Ovophis okinavensis	102	0.283%
Mus musculus	98	0.272%
Pelodiscus sinensis	89	0.247%
Oncorhynchus mykiss	86	0.239%
Alligator mississippiensis	86	0.239%
Rattus norvegicus	82	0.228%
Larimichthys crocea	81	0.225%
Gallus gallus	68	0.189%
Xenopus (Silurana) tropicalis	67	0.186%
Alligator sinensis	64	0.178%
Cricetulus griseus	63	0.175%
Monodelphis domestica	63	0.175%
Aquila chrysaetos canadensis	59	0.164%
Macaca mulatta	56	0.155%
Sus scrofa	47	0.130%
Corvus brachyrhynchos	45	0.125%
Taeniopygia guttata	45	0.125%
Apteryx australis mantelli	44	0.122%
Bos taurus	43	0.119%
Fukomys damarensis	43	0.119%
Pseudopodoces humilis	39	0.108%
Bothrops jararaca	37	0.103%
Haliaeetus leucocephalus	37	0.103%
Latimeria chalumnae	35	0.097%
Pteropus alecto	34	0.094%
Oryzias latipes	34	0.094%
Picoides pubescens	33	0.092%
Myotis brandtii	33	0.092%
Macaca fascicularis	32	0.089%
Trichuris suis	32	0.089%
Takifugu rubripes	32	0.089%
Cuculus canorus	31	0.086%
Tetraodon nigroviridis	31	0.086%

Species	#BLAST Top-Hits	Percentage
Calypte anna	31	0.086%
Bos mutus	31	0.086%
Zonotrichia albicollis	31	0.086%
Meleagris gallopavo	30	0.083%
Ficedula albicollis	30	0.083%
Sarcophilus harrisii	29	0.081%
Falco peregrinus	29	0.081%
Austrofundulus limnaeus	28	0.078%
Python molurus endogenous retrovirus	28	0.078%
Heterocephalus glaber	28	0.078%
Tupaia chinensis	28	0.078%
Aptenodytes forsteri	28	0.078%
Acanthochelys spixii	28	0.078%
Mycobacterium tuberculosis	27	0.075%
Opisthocomus hoazin	27	0.075%
Chinchilla lanigera	27	0.075%
Deinagkistrodon acutus	27	0.075%
Anas platyrhynchos	27	0.075%
Struthio camelus australis	26	0.072%
Myotis davidii	26	0.072%
Scleropages formosus	26	0.072%
Charadrius vociferus	26	0.072%
Ornithorhynchus anatinus	26	0.072%
Eptesicus fuscus	26	0.072%
Nipponia nippon	25	0.069%
Chaetura pelagica	25	0.069%
Canis lupus familiaris	25	0.069%
Erinaceus europaeus	25	0.069%
Nannospalax galili	25	0.069%
Cavia porcellus	25	0.069%
Pan troglodytes	25	0.069%
Felis catus	25	0.069%
Octodon degus	24	0.067%
Corvus cornix cornix	24	0.067%
Tinamus guttatus	24	0.067%
Esox lucius	24	0.067%
Bubalus bubalis	23	0.064%
Camelus ferus	23	0.064%
Serinus canaria	23	0.064%
Trichechus manatus latirostris	23	0.064%
Anser cygnoides domesticus	23	0.064%
Crotalus adamanteus	23	0.064%
Otolemur garnettii	22	0.061%
synthetic construct	22	0.061%
Strongylocentrotus purpuratus	22	0.061%
Melopsittacus undulatus	22	0.061%
Ovis aries musimon	22	0.061%
Orycteropus afer afer	21	0.058%
Danio rerio	21	0.058%
Cercocebus atys	21	0.058%
Callithrix jacchus	20	0.056%
Oryctolagus cuniculus	20	0.056%
Mustela putorius furo	20	0.056%

Species	#BLAST Top-Hits	Percentage
Vollenhovia emeryi	20	0.056%
Opisthorchis viverrini	20	0.056%
Elephantulus edwardii	20	0.056%
Echinops telfairi	19	0.053%
Sorex araneus	19	0.053%
Pygoscelis adeliae	19	0.053%
Egretta garzetta	19	0.053%
Microtus ochrogaster	19	0.053%
Dipodomys ordii	19	0.053%
Clavispora lusitaniae ATCC 42720	19	0.053%
Equus caballus	19	0.053%
Columba livia	19	0.053%
uncultured bacterium	19	0.053%
Trichinella spiralis	19	0.053%
Aotus nancymaae	19	0.053%
Eucalyptus grandis	19	0.053%
Gorilla gorilla gorilla	19	0.053%
Chlorocebus sabaeus	19	0.053%
Streptomyces	19	0.053%
Merops nubicus	18	0.050%
Leptosomus discolor	18	0.050%
Ailuropoda melanoleuca	18	0.050%
Stegodyphus mimosarum	17	0.047%
Cerapachys biroi	17	0.047%
Vicugna pacos	17	0.047%
Chrysochloris asiatica	17	0.047%
Balearica regulorum gibbericeps	17	0.047%
Saimiri boliviensis boliviensis	17	0.047%
Ictidomys tridecemlineatus	17	0.047%
Brassica napus	17	0.047%
Geospiza fortis	17	0.047%
Melipona quadrifasciata	17	0.047%
Ceratotherium simum simum	17	0.047%
Zea mays	17	0.047%
Candida albicans SC5314	17	0.047%
Oreochromis niloticus	17	0.047%
Lucilia cuprina	17	0.047%
Clupea harengus	17	0.047%
Nomascus leucogenys	17	0.047%
Orcinus orca	17	0.047%
Ochotona princeps	17	0.047%
Amborella trichopoda	16	0.044%
Loxodonta africana	16	0.044%
Solanum lycopersicum	16	0.044%
Thalassiosira oceanica	16	0.044%
Biomphalaria glabrata	16	0.044%
Vitis vinifera	16	0.044%
Papio anubis	16	0.044%
Mandrillus leucophaeus	16	0.044%
Camelus bactrianus	16	0.044%
Medicago truncatula	16	0.044%
Galeopterus variegatus	16	0.044%
Cynoglossus semilaevis	16	0.044%

Species	#BLAST Top-Hits	Percentage
Bothrops insularis	16	0.044%
Oryza sativa Japonica Group	15	0.042%
Theobroma cacao	15	0.042%
Physeter catodon	15	0.042%
Mesocricetus auratus	15	0.042%
Streptococcus pneumoniae	15	0.042%
Ursus maritimus	15	0.042%
Strigomonas culicis	15	0.042%
Saitoella complicata NRRL Y-17804	15	0.042%
Macaca nemestrina	15	0.042%
Capra hircus	15	0.042%
Colobus angolensis palliatus	15	0.042%
Odobenus rosmarus divergens	15	0.042%
Escherichia coli	15	0.042%
Astyanax mexicanus	15	0.042%
Mesitornis unicolor	15	0.042%
Jaculus jaculus	15	0.042%
Crassostrea gigas	15	0.042%
Tarsius syrichta	14	0.039%
Pantholops hodgsonii	14	0.039%
Caprimulgus carolinensis	14	0.039%
Microcebus murinus	14	0.039%
Octopus bimaculoides	14	0.039%
Manacus vitellinus	14	0.039%
Camelus dromedarius	14	0.039%
Falco cherrug	14	0.039%
Callorhinchus milii	14	0.039%
Peromyscus maniculatus bairdii	14	0.039%
Apaloderma vittatum	14	0.039%
Glycine max	14	0.039%
Lingula anatina	14	0.039%
Aplysia californica	14	0.039%
Lycodichthys dearborni	14	0.039%
Salmo salar	13	0.036%
Camelina sativa	13	0.036%
Fundulus heteroclitus	13	0.036%
Ovis aries	13	0.036%
Lipotes vexillifer	13	0.036%
Chrysochromulina sp. CCMP291	13	0.036%
Amyelois transitella	13	0.036%
Hydra vulgaris	13	0.036%
Pteropus vampyrus	13	0.036%
Notothenia coriiceps	13	0.036%
Xiphophorus maculatus	13	0.036%
Dasypus novemcinctus	13	0.036%
Daphnia pulex	13	0.036%
Sistrurus catenatus edwardsi	13	0.036%
Equus przewalskii	13	0.036%
Oxytricha trifallax	13	0.036%
Lottia gigantea	13	0.036%
Branchiostoma floridae	13	0.036%
Tyto alba	12	0.033%
Acanthisitta chloris	12	0.033%

Species	#BLAST Top-Hits	Percentage
Pan paniscus	12	0.033%
Bothrops neuwiedi	12	0.033%
Phaethon lepturus	12	0.033%
Myotis lucifugus	12	0.033%
Panthera tigris altaica	12	0.033%
Gossypium raimondii	12	0.033%
Gloydius brevicaudus	12	0.033%
Exophiala aquamarina CBS 119918	12	0.033%
Rhinopithecus roxellana	12	0.033%
Balaenoptera acutorostrata scammoni	12	0.033%
Pongo abelii	12	0.033%
Arabidopsis thaliana	12	0.033%
Lepisosteus oculatus	11	0.031%
Plutella xylostella	11	0.031%
Trypanosoma grayi	11	0.031%
Elaeis guineensis	11	0.031%
Condylura cristata	11	0.031%
Vibrio cholerae	11	0.031%
Python curtus endogenous retrovirus	11	0.031%
Orthriophis taeniurus	11	0.031%
Zootermopsis nevadensis	11	0.031%
Solenopsis invicta	11	0.031%
Bothrops atrox	11	0.031%
Trametes versicolor FP-101664 SS1	11	0.031%
Puccinia sorghi	11	0.031%
Plasmodium vivax North Korean	11	0.031%
Vitrella brassicaformis CCMP3155	11	0.031%
Haliaeetus albicilla	11	0.031%
Nuttalliella namaqua	11	0.031%
Capitella teleta	11	0.031%
Aphanomyces invadans	11	0.031%
Schistosoma mansoni	11	0.031%
Chlamydotis macqueenii	11	0.031%
Pseudomonas aeruginosa	10	0.028%
Nestor notabilis	10	0.028%
Xanthophyllomyces dendrorhous	10	0.028%
Dendroctonus ponderosae	10	0.028%
Maylandia zebra	10	0.028%
Pundamilia nyererei	10	0.028%
Saprolegnia diclina VS20	10	0.028%
Musa acuminata subsp. malaccensis	10	0.028%
Colletotrichum sublineola	10	0.028%
Clonorchis sinensis	10	0.028%
Talaromyces cellulolyticus	10	0.028%
Verticillium longisporum	10	0.028%
Lasius niger	10	0.028%
Propithecus coquereli	9	0.025%
Beta vulgaris subsp. vulgaris	9	0.025%
Phaeosphaeria nodorum SN15	9	0.025%
Stigmatella aurantiaca DW4/3-1	9	0.025%
Papilio xuthus	9	0.025%
Aureobasidium pullulans EXF-150	9	0.025%
Chlamydia trachomatis	9	0.025%

Species	#BLAST Top-Hits	Percentage
Nelumbo nucifera	9	0.025%
Camponotus floridanus	9	0.025%
Stenotrophomonas maltophilia	9	0.025%
Brassica oleracea var. oleracea	9	0.025%
Naegleria gruberi	9	0.025%
Paramecium tetraurelia	9	0.025%
Sorghum bicolor	9	0.025%
Schizophyllum commune H4-8	9	0.025%
Aedes aegypti	9	0.025%
Physcomitrella patens	9	0.025%
Culex quinquefasciatus	9	0.025%
Phoenix dactylifera	9	0.025%
Tauraco erythrolophus	9	0.025%
Setaria italica	9	0.025%
Phaseolus vulgaris	9	0.025%
Leptonychotes weddellii	9	0.025%
Pelecanus crispus	9	0.025%
Acromyrmex echinatior	9	0.025%
Gloydius halys	9	0.025%
Limulus polyphemus	9	0.025%
Selaginella moellendorffii	8	0.022%
Leptosphaeria maculans JN3	8	0.022%
Nicotiana tomentosiformis	8	0.022%
Tarenaya hassleriana	8	0.022%
Pseudogymnoascus pannorum VKM F-4520 (FW-2644)	8	0.022%
Phoenicopterus ruber ruber	8	0.022%
Plasmodium vivax Brazil I	8	0.022%
Giardia lamblia ATCC 50803	8	0.022%
Tursiops truncatus	8	0.022%
Paramecium tetraurelia strain d4-2	8	0.022%
Pyrus x bretschneideri	8	0.022%
Rhizoctonia solani	8	0.022%
Bacillus cereus	8	0.022%
Pseudomonas putida	8	0.022%
Plasmodiophora brassicae	8	0.022%
Nectria haematococca mpVI 77-13-4	8	0.022%
Oryza sativa Indica Group	8	0.022%
Stomoxys calcitrans	8	0.022%
Galerina marginata CBS 339.88	8	0.022%
Aphanomyces astaci	8	0.022%
Harpegnathos saltator	8	0.022%
Aegilops tauschii	8	0.022%
Emiliania huxleyi CCMP1516	8	0.022%
Drosophila busckii	8	0.022%
Buceros rhinoceros silvestris	8	0.022%
Protobothrops elegans	8	0.022%
Caenorhabditis briggsae	8	0.022%
Pseudomonas fluorescens	8	0.022%
Bordetella pertussis	8	0.022%
Reticulomyxa filosa	8	0.022%
Enterobacter cloacae	8	0.022%
Stegastes partitus	8	0.022%
Metaseiulus occidentalis	8	0.022%

Species	#BLAST Top-Hits	Percentage
Poecilia formosa	8	0.022%
Microplitis demolitor	8	0.022%
Gymnopus luxurians FD-317 M1	8	0.022%
Amphimedon queenslandica	8	0.022%
Diaphorina citri	8	0.022%
Cariama cristata	8	0.022%
Nannochloropsis gaditana	8	0.022%
Hirsutella minnesotensis 3608	7	0.019%
Chaetomium globosum CBS 148.51	7	0.019%
Magnaporthiopsis poae ATCC 64411	7	0.019%
Hypholoma sublateritium FD-334 SS-4	7	0.019%
Phanerochaete carnosa HHB-10118-sp	7	0.019%
Phalacrocorax carbo	7	0.019%
Saccoglossus kowalevskii	7	0.019%
Spinacia oleracea	7	0.019%
Allomyces macrogynus ATCC 38327	7	0.019%
Bradyrhizobium elkanii	7	0.019%
Ciona intestinalis	7	0.019%
Atta cephalotes	7	0.019%
Botryobasidium botryosum FD-172 SS1	7	0.019%
Trypanosoma vivax Y486	7	0.019%
Nematostella vectensis	7	0.019%
Gavia stellata	7	0.019%
Rhizopus delemar RA 99-880	7	0.019%
Caenorhabditis brenneri	7	0.019%
Plicaturopsis crispa FD-325 SS-3	7	0.019%
Sporothrix schenckii 1099-18	7	0.019%
Aspergillus nomius NRRL 13137	7	0.019%
Cladophialophora bantiana CBS 173.52	7	0.019%
Baudoinia compniacensis UAMH 10762	7	0.019%
Methylomicrobium buryatense	7	0.019%
Malus domestica	7	0.019%
Neolamprologus brichardi	7	0.019%
Caenorhabditis remanei	7	0.019%
Cystobacter fuscus	7	0.019%
Talaromyces stipitatus ATCC 10500	7	0.019%
Helobdella robusta	7	0.019%
Pterocles gutturalis	7	0.019%
Monomorium pharaonis	7	0.019%
Dictyostelium fasciculatum	6	0.017%
Neospora caninum Liverpool	6	0.017%
Yersinia enterocolitica	6	0.017%
Plasmodium yoelii yoelii	6	0.017%
Oikopleura dioica	6	0.017%
Aureobasidium subglaciale EXF-2481	6	0.017%
Leishmania major strain Friedlin	6	0.017%
Tetrahymena thermophila SB210	6	0.017%
Capronia epimyces CBS 606.96	6	0.017%
Pisolithus microcarpus 441	6	0.017%
Sphaerobolus stellatus SS14	6	0.017%
Cathartes aura	6	0.017%
Bothrops jararacussu	6	0.017%
Schizopora paradoxa	6	0.017%

Species	#BLAST Top-Hits	Percentage
Plasmodium berghei ANKA	6	0.017%
Pseudomonas syringae	6	0.017%
Ixodes scapularis	6	0.017%
Mycobacterium abscessus	6	0.017%
Macrophomina phaseolina MS6	6	0.017%
Epinephelus bruneus	6	0.017%
Podospora anserina S mat+	6	0.017%
Paxillus involutus ATCC 200175	6	0.017%
Acyrthosiphon pisum	6	0.017%
Stylonychia lemnae	6	0.017%
Laccaria amethystina LaAM-08-1	6	0.017%
Trypanosoma congolense IL3000	6	0.017%
Blumeria graminis f. sp. hordei DH14	6	0.017%
Pseudogymnoascus pannorum VKM F-4519 (FW-2642)	6	0.017%
Oidiodendron maius Zn	6	0.017%
Triticum urartu	6	0.017%
Verruconis gallopava	6	0.017%
Haemonchus contortus	6	0.017%
Cystobacter fuscus DSM 2262	6	0.017%
Eutypa lata UCREL1	6	0.017%
Stachybotrys chlorohalonata IBT 40285	6	0.017%
Drosophila melanogaster	6	0.017%
Colius striatus	6	0.017%
Scedosporium apiospermum	6	0.017%
Scleroderma citrinum Foug A	6	0.017%
Penicillium italicum	6	0.017%
Fonsecaea pedrosoi CBS 271.37	6	0.017%
Eurypyga helias	6	0.017%
Xenopus laevis	6	0.017%
Trichomonas vaginalis G3	6	0.017%
Grosmannia clavigera kw1407	6	0.017%
Shigella sonnei	6	0.017%
Rhizoctonia solani AG-1 IA	6	0.017%
Burkholderia pseudomallei 1710b	6	0.017%
Microcystis aeruginosa	6	0.017%
Wasmannia auropunctata	6	0.017%
Orussus abietinus	6	0.017%
Salinispora pacifica	6	0.017%
Drosophila grimshawi	6	0.017%
Bison bison bison	6	0.017%
Phytophthora sojae	6	0.017%
Lotus japonicus	6	0.017%
Morus notabilis	6	0.017%
Eimeria acervulina	6	0.017%
Glycine soja	5	0.014%
Amycolatopsis orientalis	5	0.014%
Fopius arisanus	5	0.014%
Pisolithus tinctorius Marx 270	5	0.014%
Habropoda laboriosa	5	0.014%
Gemmatimonas aurantiaca	5	0.014%
Plasmodium berghei	5	0.014%
Ralstonia solanacearum	5	0.014%
Pediculus humanus corporis	5	0.014%

Species	#BLAST Top-Hits	Percentage
Sporidiobolus salmonicolor	5	0.014%
Populus trichocarpa	5	0.014%
Ophiostoma piceae UAMH 11346	5	0.014%
Coffea canephora	5	0.014%
Kazachstania africana CBS 2517	5	0.014%
Hymenolepis microstoma	5	0.014%
Angomonas deanei	5	0.014%
Auricularia delicata TFB-10046 SS5	5	0.014%
Poecilia reticulata	5	0.014%
Pseudomonas	5	0.014%
Cicer arietinum	5	0.014%
Pyrenophora teres f. teres 0-1	5	0.014%
Mucor circinelloides f. circinelloides 1006PhL	5	0.014%
Bombyx mori	5	0.014%
Pogonomyrmex barbatus	5	0.014%
Jatropha curcas	5	0.014%
Chaetomium thermophilum var. thermophilum DSM 1495	5	0.014%
Colletotrichum higginsianum	5	0.014%
Streptomyces sp. MspMP-M5	5	0.014%
Oryza brachyantha	5	0.014%
Acytostelium subglobosum LB1	5	0.014%
Bothrops diporus	5	0.014%
Loa loa	5	0.014%
Linepithema humile	5	0.014%
Stachybotrys chartarum IBT 40288	5	0.014%
Pseudomonas chlororaphis	5	0.014%
Cyanidioschyzon merolae strain 10D	5	0.014%
Tolypocladium ophioglossoides CBS 100239	5	0.014%
Colletotrichum gloeosporioides Cg-14	5	0.014%
Fusarium avenaceum	5	0.014%
Echinococcus multilocularis	5	0.014%
Enterobacteriaceae	5	0.014%
Burkholderia gladioli	5	0.014%
Thelohanellus kitauei	5	0.014%
Haplochromis burtoni	5	0.014%
Exophiala sideris	5	0.014%
Hydrophis hardwickii	5	0.014%
Ancylostoma ceylanicum	5	0.014%
Guillardia theta CCMP2712	5	0.014%
Thielaviopsis punctulata	5	0.014%
Penicillium expansum	5	0.014%
Tuber melanosporum Mel28	5	0.014%
Rana catesbeiana	5	0.014%
Erythranthe guttata	5	0.014%
Metarhizium album ARSEF 1941	5	0.014%
Podiceps cristatus	5	0.014%
Babesia bigemina	5	0.014%
Rheinheimera baltica	5	0.014%
Termitomyces sp. J132	5	0.014%
Fulmarus glacialis	5	0.014%
Fomitopsis pinicola FP-58527 SS1	5	0.014%
Absidia idahoensis var. thermophila	5	0.014%
Tuber melanosporum	5	0.014%

Species	#BLAST Top-Hits	Percentage
Schizosaccharomyces japonicus yFS275	5	0.014%
Magnaporthe oryzae P131	5	0.014%
Phaeodactylum tricornutum CCAP 1055/1	5	0.014%
Penicillium digitatum PHI26	5	0.014%
Zostera marina	5	0.014%
Erythranthe guttatus	5	0.014%
Cyphellophora europaea CBS 101466	5	0.014%
Fomitiporia mediterranea MF3/22	5	0.014%
Schistosoma japonicum	5	0.014%
Pestalotiopsis fici W106-1	5	0.014%
fungal sp. No.11243	5	0.014%
Anopheles gambiae str. PEST	5	0.014%
Penicillium camemberti	5	0.014%
Schistosoma haematobium	5	0.014%
Brachypodium distachyon	5	0.014%
Trypanosoma cruzi strain CL Brener	4	0.011%
Salmonella enterica subsp. enterica serovar Bovismorbificans	4	0.011%
Mycobacterium tusciae	4	0.011%
Gemmatimonas aurantiaca T-27	4	0.011%
Aspergillus oryzae 100-8	4	0.011%
Capronia coronata CBS 617.96	4	0.011%
Madurella mycetomatis	4	0.011%
Leucobacter komagatae	4	0.011%
Ceratocystis platani	4	0.011%
Orf virus	4	0.011%
Moniliophthora roreri MCA 2997	4	0.011%
Arthrobotrys oligospora ATCC 24927	4	0.011%
Verticillium alfalfae VaMs.102	4	0.011%
Ustilaginoidea virens	4	0.011%
Botrytis cinerea BcDW1	4	0.011%
Mesorhizobium loti	4	0.011%
Aspergillus nidulans FGSC A4	4	0.011%
Trypanosoma cruzi	4	0.011%
Nitrincola lacisaponensis	4	0.011%
Pseudomonas alcaligenes	4	0.011%
Grimontia hollisae CIP 101886	4	0.011%
Tulasnella calospora MUT 4182	4	0.011%
Plasmodium vivax Mauritania I	4	0.011%
Sesamum indicum	4	0.011%
Paxillus rubicundulus Ve08.2h10	4	0.011%
Trichoderma reesei QM6a	4	0.011%
Lentzea albidocapillata	4	0.011%
Capsella rubella	4	0.011%
Enterococcus faecalis	4	0.011%
Neosartorya udagawae	4	0.011%
Megachile rotundata	4	0.011%
Methylobacter marinus	4	0.011%
Solirubrobacter soli	4	0.011%
Eutrema salsugineum	4	0.011%
Corynebacterium freiburgense	4	0.011%
Sorangium cellulosum	4	0.011%
Thamnophis elegans	4	0.011%
Coniophora puteana RWD-64-598 SS2	4	0.011%

Species	#BLAST Top-Hits	Percentage
Perkinsus marinus ATCC 50983	4	0.011%
Fragaria vesca subsp. vesca	4	0.011%
Drosophila simulans	4	0.011%
Anopheles sinensis	4	0.011%
Piriformospora indica DSM 11827	4	0.011%
Bipolaris oryzae ATCC 44560	4	0.011%
Aeromonas hydrophila	4	0.011%
Ophiocordyceps sinensis CO18	4	0.011%
Lichtheimia corymbifera JMRC:FSU:9682	4	0.011%
Trichoplax adhaerens	4	0.011%
Haematobacter massiliensis	4	0.011%
Stachybotrys chartarum IBT 40293	4	0.011%
Roseobacter sp. GAI101	4	0.011%
Cladophialophora immunda	4	0.011%
Tolypothrix bouteillei	4	0.011%
Coccidioides immitis RMSCC 2394	4	0.011%
Bacillus hemicellulosilyticus JCM 9152	4	0.011%
Trypanosoma cruzi marinkellei	4	0.011%
Chlamydia psittaci	4	0.011%
Cucumis melo	4	0.011%
Spirochaeta bajacaliforniensis	4	0.011%
Ceratosolen solmsi marchali	4	0.011%
Magnaporthe oryzae Y34	4	0.011%
Candida tropicalis MYA-3404	4	0.011%
Bacillus sp. JCM 19047	4	0.011%
Pseudomonas sp. FeS53a	4	0.011%
Citrus sinensis	4	0.011%
Clostridium butyricum	4	0.011%
Echinococcus granulosus	4	0.011%
Sporothrix brasiliensis 5110	4	0.011%
Pandoravirus salinus	4	0.011%
Nocardia brasiliensis	4	0.011%
Nannochloropsis gaditana CCMP526	4	0.011%
Vibrio parahaemolyticus	4	0.011%
Agrobacterium tumefaciens	4	0.011%
Mycobacterium ulcerans str. Harvey	4	0.011%
Rhizopus microsporus	4	0.011%
Phytophthora parasitica	4	0.011%
Thalassiosira pseudonana CCMP1335	4	0.011%
Rhizobium etli CNPAF512	4	0.011%
Rhinocladiella mackenziei CBS 650.93	4	0.011%
Martelella endophytica	4	0.011%
Phaeomoniella chlamydospora	4	0.011%
Plasmodium cynomolgi strain B	4	0.011%
Burkholderia cenocepacia	4	0.011%
Leisingera sp. ANG-M1	4	0.011%
Saccharomyces cerevisiae AWRI1631	4	0.011%
Wallemia mellicola CBS 633.66	4	0.011%
Pseudogymnoascus pannorum VKM F-4518 (FW-2643)	4	0.011%
Citromicrobium bathyomarinum	4	0.011%
Tribolium castaneum	4	0.011%
Pseudoclavibacter faecalis	4	0.011%
Gemmatirosa kalamazoonesis	4	0.011%

Species	#BLAST Top-Hits	Percentage
Gardnerella vaginalis	4	0.011%
Streptosporangium roseum	4	0.011%
Exophiala oligosperma	4	0.011%
Comamonas testosteroni	4	0.011%
Cladophialophora yegresii CBS 114405	4	0.011%
Penicillium solitum	4	0.011%
Oxyuranus scutellatus scutellatus	4	0.011%
Penicillium brasilianum	4	0.011%
Phytomonas sp. isolate EM1	4	0.011%
Myxococcus fulvus 124B02	4	0.011%
Polycyclovorans algicola	4	0.011%
Cucumis sativus	4	0.011%
Dicentrarchus labrax	4	0.011%
Rhizoctonia solani AG-1 IB	4	0.011%
Populus euphratica	4	0.011%
Mortierella verticillata NRRL 6337	4	0.011%
Jaapia argillacea MUCL 33604	4	0.011%
Exophiala xenobiotica	4	0.011%
Trichosurus vulpecula	4	0.011%
Plasmodium inui San Antonio 1	4	0.011%
Pseudomonas fuscovaginae	4	0.011%
Zygosaccharomyces bailii ISA1307	4	0.011%
Mycobacterium sp. URHB0044	4	0.011%
Oceanithermus profundus DSM 14977	4	0.011%
Plasmodium yoelii yoelii 17XNL	4	0.011%
Puccinia striiformis f. sp. tritici PST-78	4	0.011%
Polysphondylium pallidum PN500	4	0.011%
Fusarium fujikuroi	4	0.011%
Herbaspirillum	4	0.011%
Myxococcus fulvus	4	0.011%
Clostridiales bacterium 1_7_47FAA	4	0.011%
Wenzhouxiangella marina	4	0.011%
Tolypothrix campylonemoides VB511288	4	0.011%
Grimontia hollisae	4	0.011%
Fistulina hepatica ATCC 64428	4	0.011%
Paenibacillus mucilaginosus	4	0.011%
Clarias batrachus	4	0.011%
Ancylostoma duodenale	4	0.011%
Sagittula stellata E-37	4	0.011%
Sordaria macrospora k-hell	4	0.011%
Colletotrichum fioriniae PJ7	4	0.011%
Fusarium verticillioides 7600	4	0.011%
Sphaerulina musiva SO2202	4	0.011%
Halorubrum aidingense	3	0.008%
Enterobacter cloacae EC_38VIM1	3	0.008%
Giardia intestinalis	3	0.008%
Togninia minima UCRPA7	3	0.008%
Streptomyces sp. HCCB10043	3	0.008%
Rhodopirellula sp. K833	3	0.008%
Dichomitus squalens LYAD-421 SS1	3	0.008%
Acetobacter aceti	3	0.008%
Brevibacillus brevis	3	0.008%
Puccinia graminis f. sp. tritici CRL 75-36-700-3	3	0.008%

Species	#BLAST Top-Hits	Percentage
Toxoplasma gondii ME49	3	0.008%
Desulfovibrio vulgaris DP4	3	0.008%
Apis mellifera	3	0.008%
Thermosediminibacter oceani DSM 16646	3	0.008%
Pantherophis guttatus	3	0.008%
Rothia mucilaginosa DY-18	3	0.008%
Candidatus Brocadia sinica JPN1	3	0.008%
Bipolaris maydis ATCC 48331	3	0.008%
Ectocarpus siliculosus	3	0.008%
Drosophila persimilis	3	0.008%
Streptomyces sp. MUSC136T	3	0.008%
Thermosediminibacter oceani	3	0.008%
Defluviimonas sp. 20V17	3	0.008%
Bryobacter aggregatus	3	0.008%
Toxoplasma gondii p89	3	0.008%
Parcubacteria (Nomurabacteria) bacterium GW2011_GWA1_40_8	3	0.008%
Candidatus Magnetobacterium bavaricum	3	0.008%
Paenibacillus terrigena	3	0.008%
Fusarium oxysporum FOSC 3-a	3	0.008%
Brevundimonas diminuta	3	0.008%
Nicotiana sylvestris	3	0.008%
Haloferula sp. BvORR071	3	0.008%
Rothia mucilaginosa	3	0.008%
Cellvibrio sp. BR	3	0.008%
Trichoderma virens Gv29-8	3	0.008%
Chondromyces apiculatus	3	0.008%
Ceriporiopsis subvermispora B	3	0.008%
Microbulbifer variabilis	3	0.008%
Nocardia concava	3	0.008%
Caulobacter segnis	3	0.008%
Rhizobium tropici	3	0.008%
Corallococcus coralloides DSM 2259	3	0.008%
Parcubacteria bacterium GW2011_GWC1_40_11	3	0.008%
Cryptosporidium muris RN66	3	0.008%
Aureobasidium melanogenum CBS 110374	3	0.008%
Dictyocaulus viviparus	3	0.008%
Anopheles darlingi	3	0.008%
Reinekea sp. MED297	3	0.008%
Actinoplanes sp. SE50/110	3	0.008%
Parcubacteria (Nomurabacteria) bacterium GW2011_GWE2_40_10	3	0.008%
Galdieria sulphuraria	3	0.008%
Verticillium dahliae VdLs.17	3	0.008%
Thermobacillus composti	3	0.008%
Polyangium] brachysporum	3	0.008%
Actinomadura flavalba	3	0.008%
Fibroporia radiculosa	3	0.008%
Yersinia frederiksenii	3	0.008%
Streptomyces globisporus	3	0.008%
Pseudomonas simiae	3	0.008%
Apis florea	3	0.008%
Volvox carteri f. nagariensis	3	0.008%
Herbaspirillum frisingense GSF30	3	0.008%
Calothrix sp. PCC 6303	3	0.008%

Species	#BLAST Top-Hits	Percentage
Giardia intestinalis ATCC 50581	3	0.008%
Parcubacteria (Nomurabacteria) bacterium GW2011_GWB1_40_11	3	0.008%
Rhizobium leguminosarum	3	0.008%
Gordonia amicalis	3	0.008%
Bothrops alternatus	3	0.008%
Flavihumibacter petaseus NBRC 106054	3	0.008%
Sphingobium herbicidovorans NBRC 16415	3	0.008%
Babesia bovis	3	0.008%
Toxocara canis	3	0.008%
Capsaspora owczarzaki ATCC 30864	3	0.008%
Chondromyces apiculatus DSM 436	3	0.008%
Parcubacteria (Nomurabacteria) bacterium GW2011_GWD2_39_12	3	0.008%
Bosea sp. UNC402CLCol	3	0.008%
Streptomyces griseus	3	0.008%
Hammondia hammondi	3	0.008%
Melanopsichium pennsylvanicum 4	3	0.008%
Nocardia tenerifensis	3	0.008%
Sphingobium chlorophenolicum	3	0.008%
Arthrobacter sp. AK-YN10	3	0.008%
Batrachochytrium dendrobatidis JAM81	3	0.008%
Marinobacter hydrocarbonoclasticus VT8	3	0.008%
Plasmodium chabaudi chabaudi	3	0.008%
Vigna angularis	3	0.008%
Streptomyces sp. NRRL F-6491	3	0.008%
Streptomyces sp. NRRL F-6492	3	0.008%
Coniosporium apollinis CBS 100218	3	0.008%
Sphingomonas sp. ERG5	3	0.008%
Bitis gabonica	3	0.008%
Fusarium graminearum	3	0.008%
Plasmodium vivax	3	0.008%
Novosphingobium sp. MBES04	3	0.008%
Meiothermus chliarophilus	3	0.008%
Pseudomonas chlororaphis subsp. aureofaciens 30-84	3	0.008%
Amycolatopsis sp. MJM2582	3	0.008%
Spirochaeta sp. JC230	3	0.008%
Parcubacteria bacterium GW2011_GWA1_45_10	3	0.008%
Eremothecium gossypii ATCC 10895	3	0.008%
Paenibacillus massiliensis	3	0.008%
Phytophthora parasitica P10297	3	0.008%
Penicillium oxalicum 114-2	3	0.008%
Danaus plexippus	3	0.008%
Pseudogymnoascus pannorum VKM F-4513 (FW-928)	3	0.008%
Bifidobacterium bifidum	3	0.008%
Leiolepis reevesii rubritaeniata	3	0.008%
Gordonia amicalis NBRC 100051 = JCM 11271	3	0.008%
Papilio polytes	3	0.008%
Neorhizobium galegae bv. officinalis	3	0.008%
Zymoseptoria tritici IPO323	3	0.008%
Pedobacter borealis	3	0.008%
Oscillatoria nigro-viridis PCC 7112	3	0.008%
Prunus mume	3	0.008%
Stigmatella aurantiaca	3	0.008%
Drosophila erecta	3	0.008%

Species	#BLAST Top-Hits	Percentage
Streptomyces catenulae	3	0.008%
Herbidospora cretacea	3	0.008%
Ophiocordyceps unilateralis	3	0.008%
Lysinibacillus sp. FJAT-14745	3	0.008%
Chondrus crispus	3	0.008%
Corallococcus coralloides	3	0.008%
Streptomyces sp. NRRL B-24484	3	0.008%
Fonsecaea multimorphosa CBS 102226	3	0.008%
Fusarium oxysporum f. sp. melonis 26406	3	0.008%
Roseomonas gilardii	3	0.008%
Micromonas sp. RCC299	3	0.008%
Nitrospirillum amazonense Y2	3	0.008%
Prevotella bryantii	3	0.008%
Microsporum gypseum CBS 118893	3	0.008%
Neorhizobium galegae	3	0.008%
Nocardia veterana	3	0.008%
Trichoderma reesei RUT C-30	3	0.008%
Caulobacter sp. K31	3	0.008%
Bifidobacterium angulatum	3	0.008%
Oesophagostomum dentatum	3	0.008%
Malassezia sympodialis ATCC 42132	3	0.008%
Zygosaccharomyces rouxii	3	0.008%
Rhizophagus irregularis DAOM 197198w	3	0.008%
Lodderomyces elongisporus NRRL YB-4239	3	0.008%
Vibrio genomosp. F10	3	0.008%
Pseudomonas amygdali pv. sesami	3	0.008%
Ashbya gossypii ATCC 10895	3	0.008%
Fusarium oxysporum Fo5176	3	0.008%
Prunus persica	3	0.008%
Desulfovibrio vulgaris	3	0.008%
Streptomyces hygroscopicus	3	0.008%
Plasmodium vivax India VII	3	0.008%
Kitasatospora phosalacinea	3	0.008%
Burkholderia phymatum STM815	3	0.008%
Synechococcus sp. WH 7803	3	0.008%
Cryptococcus gattii IND107	3	0.008%
Croceicoccus naphthovorans	3	0.008%
Gossypium arboreum	3	0.008%
Penicillium digitatum Pd1	3	0.008%
Prevotella sp. CAG:474	3	0.008%
Operophtera brumata	3	0.008%
Citrus clementina	3	0.008%
Parcubacteria bacterium GW2011_GWF1_40_5	3	0.008%
Metarhizium anisopliae BRIP 53284	3	0.008%
Lactobacillus brevis	3	0.008%
Bifidobacterium longum	3	0.008%
Bothrops jararaca. venom. Peptide. 142 aa	3	0.008%
Pseudozyma antarctica T-34	3	0.008%
Leptothrix cholodnii SP-6	3	0.008%
Dothistroma septosporum NZE10	3	0.008%
Exophiala mesophila	3	0.008%
Caldithrix abyssi	3	0.008%
Parcubacteria (Nomurabacteria) bacterium GW2011_GWC2_39_41	3	0.008%

Species	#BLAST Top-Hits	Percentage
Leishmania mexicana MHOM/GT/2001/U1103	3	0.008%
Setosphaeria turcica Et28A	3	0.008%
Pseudomonas sp. RIT357	3	0.008%
Streptomyces vietnamensis	3	0.008%
Apis dorsata	3	0.008%
Acanthamoeba polyphaga mimivirus	3	0.008%
Pseudozyma flocculosa PF-1	3	0.008%
Millerozyma farinosa CBS 7064	3	0.008%
Clostridiales bacterium VE202-28	3	0.008%
Caldithrix abyssi DSM 13497	3	0.008%
Enterovibrio norvegicus	3	0.008%
Reyranella massiliensis	3	0.008%
Mastigocladus laminosus	3	0.008%
Xylanimonas cellulosilytica DSM 15894	3	0.008%
Salpingoeca rosetta	3	0.008%
Bothrops jararaca. venom. Peptide. 123 aa	3	0.008%
Rivularia sp. PCC 7116	3	0.008%
Rhodococcus rhodnii	3	0.008%
Amanita muscaria Koide BX008	3	0.008%
Candidatus Endolissoclinum faulkneri	3	0.008%
Candidatus Brocadia sinica	3	0.008%
Trypanosoma brucei gambiense DAL972	3	0.008%
Oceanimonas smirnovii	3	0.008%
Kitasatospora sp. NRRL B-11411	3	0.008%
Trametes cinnabarina	3	0.008%
Hebeloma cylindrosporum h7	3	0.008%
Oscillatoria nigro-viridis	3	0.008%
Budvicia aquatica	3	0.008%
Leptothrix cholodnii	3	0.008%
Microvirgula aerodenitrificans	3	0.008%
Fusarium fujikuroi IMI 58289	3	0.008%
Human immunodeficiency virus 1	3	0.008%
Streptomyces antibioticus	3	0.008%
Flavihumibacter petaseus	3	0.008%
Bothrops jararaca. venom. Peptide. 232 aa	3	0.008%
Drosophila yakuba	3	0.008%
Pseudomonas sp. 250J	3	0.008%
Aestuariivita boseongensis	3	0.008%
Rhizoctonia solani 123E	3	0.008%
Paenibacillus	3	0.008%
Caulobacter segnis ATCC 21756	3	0.008%
Saprolegnia parasitica CBS 223.65	3	0.008%
Acidithiobacillus caldus	3	0.008%
Halorubrum aidingense JCM 13560	3	0.008%
Solanum tuberosum	3	0.008%
Microbacterium gubbeenense	3	0.008%
Laticauda semifasciata	3	0.008%
Metarhizium acridum CQMa 102	3	0.008%
Burkholderia phymatum	3	0.008%
Mastigocladus laminosus UU774	3	0.008%
Comamonas sp. B-9	3	0.008%
Eremothecium gossypii FDAG1	3	0.008%
Brevundimonas diminuta ATCC 11568	3	0.008%

Species	#BLAST Top-Hits	Percentage
Fusarium graminearum PH-1	3	0.008%
Rhizoctonia solani AG-8 WAC10335	3	0.008%
Thecamonas trahens ATCC 50062	3	0.008%
Porphyrobacter cryptus	3	0.008%
Cylindrobasidium torrendii FP15055 ss-10	3	0.008%
Histoplasma capsulatum NAm1	3	0.008%
Nasonia vitripennis	3	0.008%
Auxenochlorella protothecoides	3	0.008%
Vibrio vulnificus	3	0.008%
Pseudozyma brasiliensis GHG001	3	0.008%
Neurospora crassa	3	0.008%
Ricinus communis	3	0.008%
Fonticula alba	3	0.008%
Caenorhabditis elegans	3	0.008%
Micromonospora chokoriensis	3	0.008%
Paenibacillus sp. GD11	3	0.008%
Candidatus Solibacter usitatus	3	0.008%
Microbispora sp. ATCC PTA-5024	3	0.008%
Melampsora larici-populina 98AG31	3	0.008%
Xylanimonas cellulosilytica	3	0.008%
Vibrio genomosp. F6	3	0.008%
Coccidioides posadasii RMSCC 3488	3	0.008%
Candidatus Solibacter usitatus Ellin6076	3	0.008%
Longilinea arvoryzae	3	0.008%
Paenibacillus polymyxa	3	0.008%
Tolypothrix campylonemoides	3	0.008%
Methanoregula boonei 6A8	3	0.008%
Exophiala spinifera	3	0.008%
Mycobacterium iranicum	3	0.008%
Naumovozyma dairenensis CBS 421	3	0.008%
Paenibacillus odorifer	3	0.008%
Methanobrevibacter ruminantium	3	0.008%
Theileria orientalis strain Shintoku	3	0.008%
Blastomyces dermatitidis ATCC 18188	3	0.008%
bacterium UASB14	3	0.008%
Drosophila sechellia	3	0.008%
Arabidopsis lyrata subsp. lyrata	3	0.008%
Paracoccidioides sp. 'lutzii' Pb01	3	0.008%
Emmonsia crescens UAMH 3008	3	0.008%
Gordonia sp. NB4-1Y	3	0.008%
Endocarpon pusillum Z07020	3	0.008%
Enhygromyxa salina	3	0.008%
Marssonina brunnea f. sp. 'multigermtubi' MB_m1	3	0.008%
Suillus luteus UH-Slu-Lm8-n1	3	0.008%
Emmonsia parva UAMH 139	3	0.008%
Stachybotrys chartarum IBT 7711	3	0.008%
Kribbella catacumbae	3	0.008%
Treponema succinifaciens DSM 2489	3	0.008%
Hydnomerulius pinastri MD-312	3	0.008%
Salinibacter ruber M8	3	0.008%
Streptomyces viridochromogenes	3	0.008%
Saccharomyces cerevisiae	3	0.008%
Streptomyces sp. MUSC149T	3	0.008%

Species	#BLAST Top-Hits	Percentage
Arthrobacter sp. H5	3	0.008%
Pseudoalteromonas flavipulchra	3	0.008%
Neisseria meningitidis	3	0.008%
Solirubrobacter sp. URHD0082	3	0.008%
Musca domestica	3	0.008%
Streptomyces exfoliatus	3	0.008%
Postia placenta Mad-698-R	3	0.008%
Methanobrevibacter ruminantium M1	3	0.008%
Theileria annulata	3	0.008%
Torrubiella hemipterigena	3	0.008%
Chlorella variabilis	3	0.008%
Microcystis aeruginosa PCC 7806	3	0.008%
Micromonospora sp. RV43	3	0.008%
Trypanosoma rangeli SC58	3	0.008%
Colletotrichum gloeosporioides Nara gc5	3	0.008%
Legionella hackeliae	3	0.008%
Bacillus sp. FJAT-18019	2	0.006%
Pseudomonas pseudoalcaligenes	2	0.006%
Burkholderia bannensis	2	0.006%
Sulfurovum sp. AS07-7	2	0.006%
Babesia equi strain WA	2	0.006%
Trichophyton interdigitale H6	2	0.006%
Nocardia sp. BMG111209	2	0.006%
Syntrophus aciditrophicus	2	0.006%
Streptococcus pseudopneumoniae	2	0.006%
Desulforegula conservatrix	2	0.006%
Aeromonas caviae	2	0.006%
Candidatus Blastococcus massiliensis	2	0.006%
Streptomyces europaeiscabiei	2	0.006%
Pseudoalteromonas tunicata	2	0.006%
Desulfitobacterium hafniense	2	0.006%
Eimeria maxima	2	0.006%
Burkholderia pseudomallei	2	0.006%
Nonomuraea candida	2	0.006%
Mycobacterium setense	2	0.006%
Bilophila sp. 4_1_30	2	0.006%
Simiduia agarivorans	2	0.006%
Nonomuraea sp. SBT364	2	0.006%
Hyphomonadaceae bacterium BRH_c29	2	0.006%
Acaryochloris marina	2	0.006%
Exiguobacterium	2	0.006%
Chryseobacterium koreense	2	0.006%
Streptomyces sp. NRRL S-37	2	0.006%
Pelosinus fermentans	2	0.006%
Pseudomonas sp. VLB120	2	0.006%
Trichophyton tonsurans CBS 112818	2	0.006%
Sideroxydans lithotrophicus	2	0.006%
Schizosaccharomyces octosporus yFS286	2	0.006%
Frankia sp. CpI1-S	2	0.006%
Bradyrhizobium diazoefficiens USDA 110	2	0.006%
Methylocystis parvus	2	0.006%
Trichosporon oleaginosus	2	0.006%
Streptomyces davawensis JCM 4913	2	0.006%

Species	#BLAST Top-Hits	Percentage
Haemophilus] parasuis	2	0.006%
Desulfatitalea sp. BRH_c12	2	0.006%
Nocardia transvalensis	2	0.006%
Streptomyces pristinaespiralis	2	0.006%
Uncinocarpus reesii 1704	2	0.006%
Roseomonas aerilata	2	0.006%
Lachancea thermotolerans	2	0.006%
Dialister succinatiphilus	2	0.006%
Toxoplasma gondii MAS	2	0.006%
Coraliomargarita akajimensis DSM 45221	2	0.006%
Acidithiobacillus caldus SM-1	2	0.006%
Hydrogenobaculum sp. SN	2	0.006%
Bacillus sonorensis	2	0.006%
Marinobacter lipolyticus	2	0.006%
Hydrogenivirga sp. 128-5-R1-1	2	0.006%
Kutzneria albida DSM 43870	2	0.006%
Actinomyces sp. oral taxon 171 str. F0337	2	0.006%
Streptomyces sp. NBRC 110027	2	0.006%
Bacillus cereus VD136	2	0.006%
Paenibacillus sp. Y412MC10	2	0.006%
Mycobacterium marinum	2	0.006%
Rhizobium gallicum	2	0.006%
Rhodococcus	2	0.006%
Lactobacillus delbrueckii	2	0.006%
Haemophilus influenzae	2	0.006%
Streptomyces sp. 303MFCol5.2	2	0.006%
Streptomyces sp. MUSC164	2	0.006%
Rheinheimera sp. A13L	2	0.006%
Bordetella hinzii	2	0.006%
Saccharothrix sp. NRRL B-16314	2	0.006%
Hydrogenobaculum sp. HO	2	0.006%
Bacillus sp. FJAT-22058	2	0.006%
Methylocystis sp. SB2	2	0.006%
Bifidobacterium asteroides	2	0.006%
Desulfobacca acetoxidans	2	0.006%
Parcubacteria (Nomurabacteria) bacterium GW2011_GWF2_40_31	2	0.006%
Saccharothrix sp. NRRL B-16348	2	0.006%
Thiomicrospira crunogena	2	0.006%
Anoxybacillus flavithermus NBRC 109594	2	0.006%
Mesorhizobium	2	0.006%
Lactobacillus composti DSM 18527 = JCM 14202	2	0.006%
Reinekea blandensis	2	0.006%
Geobacter uraniireducens	2	0.006%
Shewanella sp. MR-4	2	0.006%
Geobacter uraniireducens Rf4	2	0.006%
Punctularia strigosozonata HHB-11173 SS5	2	0.006%
Streptomyces sp. NRRL F-2747	2	0.006%
Cryocola sp. 340MFSha3.1	2	0.006%
Marinobacter hydrocarbonoclasticus	2	0.006%
Myroides odoratimimus	2	0.006%
Sphingobium lactosutens DS20	2	0.006%
Sphingobium herbicidovorans	2	0.006%
Pseudogymnoascus pannorum VKM F-3808	2	0.006%

Species	#BLAST Top-Hits	Percentage
Helicobacter pylori	2	0.006%
Streptomyces sp. NRRL F-2799	2	0.006%
Pseudomonas psychrophila	2	0.006%
Vibrio halioticoli NBRC 102217	2	0.006%
Dactylellina haptotyla CBS 200.50	2	0.006%
Trichosporon asahii var. asahii CBS 2479	2	0.006%
Eubacterium limosum	2	0.006%
Schizosaccharomyces pombe	2	0.006%
Gloydius blomhoffii	2	0.006%
Yersinia pseudotuberculosis	2	0.006%
Sorangium cellulosum So ce56	2	0.006%
Aspergillus niger	2	0.006%
Syntrophus aciditrophicus SB	2	0.006%
Mycobacterium sp. URHD0025	2	0.006%
Streptomyces sp. CNQ329	2	0.006%
Streptomyces sp. NRRL F-3213	2	0.006%
Exophiala dermatitidis NIH/UT8656	2	0.006%
Dyadobacter crusticola	2	0.006%
Chlamydomonas reinhardtii	2	0.006%
Frankia sp. CeD	2	0.006%
Tannerella sp. CAG:118	2	0.006%
Clostridium sp. ATCC 29733	2	0.006%
Taphrina deformans PYCC 5710	2	0.006%
Heterobasidion irregulare TC 32-1	2	0.006%
Rhodovulum sp. PH10	2	0.006%
Paenibacillus daejeonensis	2	0.006%
archaeon GW2011_AR3	2	0.006%
Azoarcus sp. KH32C	2	0.006%
Clostridium sp. Ade.TY	2	0.006%
Streptomyces sp. LaPpAH-108	2	0.006%
Arthroderma otae CBS 113480	2	0.006%
Martelella sp. AD-3	2	0.006%
Belnapia moabensis	2	0.006%
Bacteroides pyogenes	2	0.006%
Klebsiella oxytoca	2	0.006%
Chelonid herpesvirus 5	2	0.006%
Jiangella muralis	2	0.006%
Exiguobacterium sp. AB2	2	0.006%
Penicillium roqueforti FM164	2	0.006%
Mycobacterium septicum	2	0.006%
Nocardioides sp. URHA0020	2	0.006%
Acidobacteriaceae bacterium URHE0068	2	0.006%
Candidatus Clostridium anorexicamassiliense	2	0.006%
Acipenser ruthenus	2	0.006%
Colwellia psychrerythraea	2	0.006%
Acaryochloris marina MBIC11017	2	0.006%
Micromonas pusilla CCMP1545	2	0.006%
Drosophila willistoni	2	0.006%
Acidithiobacillus caldus ATCC 51756	2	0.006%
Xanthomonas vesicatoria ATCC 35937	2	0.006%
Babesia bovis T2Bo	2	0.006%
Oscillatoria acuminata	2	0.006%
Pseudogymnoascus pannorum VKM F-3775	2	0.006%

Species	#BLAST Top-Hits	Percentage
Coraliomargarita akajimensis	2	0.006%
Lechevalieria aerocolonigenes	2	0.006%
Cupriavidus necator	2	0.006%
Alcaligenes faecalis	2	0.006%
Adhaeribacter aquaticus	2	0.006%
Streptomyces sp. NTK 937	2	0.006%
Streptomyces lavenduligriseus	2	0.006%
Bipolaris victoriae FI3	2	0.006%
Streptomyces violaceusniger	2	0.006%
Photobacterium angustum	2	0.006%
Chloroflexus aurantiacus J-10-fl	2	0.006%
Bradyrhizobium diazoefficiens	2	0.006%
Klebsiella michiganensis	2	0.006%
Rhodococcus sp. UNC23MFCrub1.1	2	0.006%
Bothrops erythromelas	2	0.006%
Ustilago maydis 521	2	0.006%
Cyanobacterium aponinum	2	0.006%
Hylemonella gracilis str. Niagara R	2	0.006%
Pseudomonas syringae CC1557	2	0.006%
Chlorobium limicola DSM 245	2	0.006%
Butyrivibrio sp. AE2032	2	0.006%
Beauveria bassiana D1-5	2	0.006%
Amycolatopsis mediterranei S699	2	0.006%
Archangium gephyra	2	0.006%
Idiomarina zobellii	2	0.006%
Monosiga brevicollis MX1	2	0.006%
Thioalkalivibrio	2	0.006%
Oscillatoria acuminata PCC 6304	2	0.006%
Bitis arietans	2	0.006%
Nocardia vinacea	2	0.006%
Pseudoalteromonas luteoviolacea 2ta16	2	0.006%
Pyrenophora tritici-repentis Pt-1C-BFP	2	0.006%
Chlorobium limicola	2	0.006%
Cryobacterium sp. MLB-32	2	0.006%
Taterapox virus	2	0.006%
Psychrilyobacter atlanticus	2	0.006%
Jeotgalibacillus alimentarius	2	0.006%
Entamoeba histolytica HM-3:IMSS	2	0.006%
Cupriavidus basilensis	2	0.006%
Sphingobacterium sp. ACCC 05744	2	0.006%
Paenibacillus sp. FSL R5-0912	2	0.006%
Wickerhamomyces ciferrii	2	0.006%
Plasmodium falciparum IGH-CR14	2	0.006%
Streptomyces sp. NRRL S-384	2	0.006%
Piloderma croceum F 1598	2	0.006%
Deinococcus maricopensis	2	0.006%
Strongyloides ratti	2	0.006%
Marinobacter sp. EVN1	2	0.006%
Streptomyces viridochromogenes Tue57	2	0.006%
Neofusicoccum parvum UCRNP2	2	0.006%
Leucobacter chironomi	2	0.006%
Rhodopseudomonas palustris	2	0.006%
Jeotgalibacillus soli Cunha et al. 2012	2	0.006%

Species	#BLAST Top-Hits	Percentage
Streptomyces sp. NRRL F-5126	2	0.006%
Prevotella ruminicola	2	0.006%
Vibrio halioticoli	2	0.006%
Streptomyces sp. NRRL S-325	2	0.006%
Clostridium sp. KLE 1755	2	0.006%
Chloroflexus sp. Y-400-fl	2	0.006%
Calothrix parietina	2	0.006%
Streptomyces albulus	2	0.006%
Acinetobacter radioresistens	2	0.006%
Leptospirillum ferriphilum YSK	2	0.006%
Brevibacillus thermoruber	2	0.006%
Natrialba taiwanensis DSM 12281	2	0.006%
Methylobacter sp. BBA5.1	2	0.006%
Claviceps purpurea 20.1	2	0.006%
Micromonospora sp. CNB394	2	0.006%
Herbaspirillum sp. CF444	2	0.006%
Pirellula staleyi	2	0.006%
Cronobacter malonaticus	2	0.006%
Desulfurispora thermophila	2	0.006%
Leishmania panamensis	2	0.006%
Hymenobacter sp. MIMtkLc17	2	0.006%
Odoribacter splanchnicus CAG:14	2	0.006%
Gordonia rhizosphera NBRC 16068	2	0.006%
Brevibacterium massiliense	2	0.006%
Microcoleus sp. PCC 7113	2	0.006%
Brevibacterium linens	2	0.006%
Caedibacter varicaedens	2	0.006%
Aliiglaciecola lipolytica	2	0.006%
Octadecabacter temperatus	2	0.006%
Frankia alni ACN14a	2	0.006%
Streptomyces acidiscabies	2	0.006%
Pseudomonas stutzeri ATCC 14405 = CCUG 16156	2	0.006%
Flavobacterium sp. VMW	2	0.006%
Paenibacillus barengoltzii G22	2	0.006%
uncultured organism	2	0.006%
Bacillus sp. SA1-12	2	0.006%
Dickeya zeae	2	0.006%
Phytophthora parasitica P1569	2	0.006%
Acidobacteriaceae bacterium KBS 89	2	0.006%
Ilumatobacter coccineus YM16-304	2	0.006%
Phytophthora parasitica INRA-310	2	0.006%
Sclerotinia sclerotiorum 1980	2	0.006%
Erwinia typographi	2	0.006%
Planctopirus limnophila	2	0.006%
Ruminococcus bromii L2-63	2	0.006%
Methylocapsa acidiphila	2	0.006%
Parcubacteria (Nomurabacteria) bacterium GW2011_GWA2_40_97	2	0.006%
Oscillibacter sp. 1-3	2	0.006%
Shewanella loihica	2	0.006%
Ferrimonas balearica	2	0.006%
Ruegeria mobilis F1926	2	0.006%
Gorilla gorilla	2	0.006%
Dialister succinatiphilus YIT 11850	2	0.006%

Species	#BLAST Top-Hits	Percentage
Kurthia massiliensis	2	0.006%
Virgibacillus halodenitrificans	2	0.006%
Drysdalia coronoides	2	0.006%
Ostreococcus tauri	2	0.006%
Pseudomonas sp. DSM 28141	2	0.006%
Geitlerinema sp. PCC 7407	2	0.006%
Paraglaciecola psychrophila 170	2	0.006%
Klebsiella pneumoniae IS22	2	0.006%
Micrurus corallinus	2	0.006%
Sphingobium lactosutens	2	0.006%
Bdellovibrio exovorus	2	0.006%
Caenispirillum salinarum AK4	2	0.006%
Paenibacillus mucilaginosus KNP414	2	0.006%
Algoriphagus marincola	2	0.006%
Marmoricola sp. URHB0036	2	0.006%
Desulfatibacillum alkenivorans AK-01	2	0.006%
Trichoderma atroviride IMI 206040	2	0.006%
Mycobacterium sp. 360MFTsu5.1	2	0.006%
Natronococcus amylolyticus	2	0.006%
Deinococcus deserti	2	0.006%
uncultured bacterium (gcode 4)	2	0.006%
Syntrophomonas zehnderi	2	0.006%
Hydrogenobaculum sp. 3684	2	0.006%
Actinobaculum sp. oral taxon 183	2	0.006%
Shimwellia blattae DSM 4481 = NBRC 105725	2	0.006%
Actinomyces georgiae	2	0.006%
Penicillium nordicum	2	0.006%
Vibrio rhizosphaerae	2	0.006%
Microlunatus phosphovorus	2	0.006%
Cyclobacterium marinum	2	0.006%
Burkholderia grimmiae	2	0.006%
Trabulsiella guamensis ATCC 49490	2	0.006%
Eubacterium sp. CAG:841	2	0.006%
Leptolyngbya sp. PCC 7375	2	0.006%
Bipolaris maydis C5	2	0.006%
Photorhabdus temperata	2	0.006%
Salinispira pacifica	2	0.006%
Streptomyces olivaceus	2	0.006%
Bothrops asper	2	0.006%
Blautia sp. CAG:37	2	0.006%
Paenibacillus algorifonticola	2	0.006%
Trimeresurus stejnegeri	2	0.006%
Sphingobacterium sp. 21	2	0.006%
Coprinopsis cinerea okayama7#130	2	0.006%
Gammaproteobacteria bacterium MFB021	2	0.006%
Theileria parva strain Muguga	2	0.006%
Paenibacillus sp. P22	2	0.006%
Mixia osmundae IAM 14324	2	0.006%
Bacillus mycoides	2	0.006%
Cladophialophora psammophila CBS 110553	2	0.006%
Luteibacter sp. 9133	2	0.006%
Oribacterium sinus F0268	2	0.006%
Caldibacillus debilis	2	0.006%

Species	#BLAST Top-Hits	Percentage
Fluviicola taffensis	2	0.006%
Streptomyces ochraceiscleroticus	2	0.006%
Naja atra	2	0.006%
Labrenzia sp. DG1229	2	0.006%
Cyanobacterium aponinum PCC 10605	2	0.006%
Colletotrichum orbiculare MAFF 240422	2	0.006%
endosymbiont of Riftia pachyptila (vent Ph05)	2	0.006%
Desulfovibrio alcoholivorans	2	0.006%
Terrimonas ferruginea	2	0.006%
Cellulomonas sp. URHE0023	2	0.006%
Nautilia profundicola AmH	2	0.006%
Pectobacterium carotovorum	2	0.006%
Blastomyces dermatitidis ER-3	2	0.006%
Desulfonatronovibrio hydrogenovorans	2	0.006%
Thiocapsa marina 5811	2	0.006%
Paraglaciecola psychrophila	2	0.006%
Sporomusa ovata DSM 2662	2	0.006%
Acetobacter persici	2	0.006%
Herminiimonas arsenicoxydans	2	0.006%
Anaerostipes sp. CAG:276	2	0.006%
Shewanella halifaxensis	2	0.006%
Myxococcus xanthus DK 1622	2	0.006%
Kluyveromyces marxianus DMKU3-1042	2	0.006%
candidate division TM7 genomosp. GTL1	2	0.006%
Crotalus oreganus helleri	2	0.006%
Pseudoalteromonas tunicata D2	2	0.006%
Jeotgalicoccus sp. 13MG44_air	2	0.006%
Butyrivibrio proteoclasticus	2	0.006%
Shewanella halifaxensis HAW-EB4	2	0.006%
Clostridium sp. JCD	2	0.006%
Oxya chinensis	2	0.006%
Ketogulonicigenium vulgare	2	0.006%
Spirosoma radiotolerans	2	0.006%
Genlisea aurea	2	0.006%
Azospirillum thiophilum	2	0.006%
Xylella fastidiosa	2	0.006%
Desulfarculus sp. SPR	2	0.006%
Burkholderia fungorum	2	0.006%
Stenotrophomonas rhizophila	2	0.006%
Paenibacillus sp. FSL H8-237	2	0.006%
Rhodococcus wratislaviensis	2	0.006%
Drosophila subobscura	2	0.006%
alpha proteobacterium Q-1	2	0.006%
Mesorhizobium sp. LSJC280B00	2	0.006%
Clostridium sp. CAG:149	2	0.006%
Sporisorium scitamineum	2	0.006%
Brevibacterium casei	2	0.006%
Rhodomicrobium vannielii	2	0.006%
Thermodesulfobium narugense	2	0.006%
Paenibacillus sp. FSL R7-0331	2	0.006%
Sagittula stellata	2	0.006%
Cryptococcus gattii CA1873	2	0.006%
Halomonas sp. S2151	2	0.006%

Species	#BLAST Top-Hits	Percentage
Thiocapsa marina	2	0.006%
Agkistrodon piscivorus leucostoma	2	0.006%
Aliiglaciecola lipolytica E3	2	0.006%
Schizosaccharomyces pombe 972h-	2	0.006%
Amycolatopsis orientalis HCCB10007	2	0.006%
Aeromonas salmonicida	2	0.006%
Bungarus flaviceps	2	0.006%
Pseudogymnoascus pannorum VKM F-4517 (FW-2822)	2	0.006%
Trypanosoma cruzi Dm28c	2	0.006%
Corynebacterium casei	2	0.006%
Kitasatospora cheerisanensis KCTC 2395	2	0.006%
Paraglaciecola chathamensis S18K6	2	0.006%
Prochlorococcus marinus	2	0.006%
beta proteobacterium L13	2	0.006%
Serinicoccus marinus	2	0.006%
Streptomyces rimosus	2	0.006%
Parcubacteria (Uhrbacteria) bacterium GW2011_GWF2_39_13	2	0.006%
Salinivibrio socompensis	2	0.006%
Bacillus selenatarsenatis	2	0.006%
Capra sibirica	2	0.006%
Firmicutes bacterium CAG:114	2	0.006%
Plasmodium hylobati	2	0.006%
Arthrobacter sp. 35/47	2	0.006%
Enterococcus phoeniculicola	2	0.006%
Halomonas sp. BC04	2	0.006%
Geitlerinema sp. PCC 7105	2	0.006%
Hungatella hathewayi	2	0.006%
Laccaria bicolor S238N-H82	2	0.006%
Streptococcus gordonii	2	0.006%
Arthrobacter sp. Br18	2	0.006%
Mycobacterium yongonense 05-1390	2	0.006%
Thermus oshimai	2	0.006%
Candidatus Methylopumilus planktonicus	2	0.006%
Streptomyces xanthophaeus	2	0.006%
Hydrocarboniphaga effusa AP103	2	0.006%
Acinetobacter	2	0.006%
Pseudogymnoascus pannorum VKM F-4514 (FW-929)	2	0.006%
Natrialba taiwanensis	2	0.006%
Paraglaciecola chathamensis	2	0.006%
Hyphomicrobium sp. GJ21	2	0.006%
Nocardia jiangxiensis	2	0.006%
Rhodococcus opacus	2	0.006%
Burkholderia ambifaria MEX-5	2	0.006%
Trichoderma harzianum	2	0.006%
Aequorivita sublithincola	2	0.006%
Rubrobacter radiotolerans	2	0.006%
Tolypothrix bouteillei VB521301	2	0.006%
Actinomadura madurae	2	0.006%
Dyadobacter fermentans	2	0.006%
Talaromyces marneffei PM1	2	0.006%
Burkholderiaceae bacterium 16	2	0.006%
Natrinema pellirubrum DSM 15624	2	0.006%
Pseudogymnoascus pannorum VKM F-4281 (FW-2241)	2	0.006%

Species	#BLAST Top-Hits	Percentage
Enterobacter aerogenes	2	0.006%
Corynebacterium ulcerans	2	0.006%
Streptomyces pluripotens	2	0.006%
Herbaspirillum sp. TSA66	2	0.006%
Actinopolyspora mortivallis	2	0.006%
Thermosphaera aggregans	2	0.006%
Rahnella aquatilis CIP 78.65 = ATCC 33071	2	0.006%
Sporocytophaga myxococcoides	2	0.006%
Bradyrhizobium oligotrophicum S58	2	0.006%
Streptomyces sp. ACT-1	2	0.006%
Gordonia rhizosphera	2	0.006%
Pseudonocardia autotrophica	2	0.006%
Eubacterium ventriosum ATCC 27560	2	0.006%
Amycolatopsis taiwanensis	2	0.006%
uncultured marine microorganism HF4000_APKG10K24	2	0.006%
Trabulsiella odontotermitis	2	0.006%
Burkholderia gladioli BSR3	2	0.006%
Bacillus cereus VDM021	2	0.006%
Teredinibacter turnerae	2	0.006%
Rasamsonia emersonii CBS 393.64	2	0.006%
Streptomyces decoyicus	2	0.006%
Hamadaea tsunoensis	2	0.006%
Pseudomonas sp. 655	2	0.006%
Bradyrhizobium sp. LTSP885	2	0.006%
Bacillus manliponensis	2	0.006%
Kluyveromyces dobzhanskii CBS 2104	2	0.006%
Pneumocystis jirovecii	2	0.006%
Streptomyces regensis	2	0.006%
Leptospira kirschneri	2	0.006%
Dictyostelium discoideum AX4	2	0.006%
Trichophyton rubrum CBS 118892	2	0.006%
Shimwellia blattae	2	0.006%
Meyerozyma guilliermondii ATCC 6260	2	0.006%
Pleurotus ostreatus PC15	2	0.006%
Streptomyces griseoflavus	2	0.006%
Albugo candida	2	0.006%
Crotalus horridus	2	0.006%
Agaricus bisporus var. burnettii JB137-S8	2	0.006%
Candida maltosa Xu316	2	0.006%
Cupriavidus sp. HPC(L)	2	0.006%
Candidatus Accumulibacter sp. BA-92	2	0.006%
Lysinimicrobium mangrovi	2	0.006%
Listeria monocytogenes	2	0.006%
Bradyrhizobium sp. STM 3843	2	0.006%
Pseudomonas batumici	2	0.006%
Luteibacter yeojuensis	2	0.006%
Streptomyces albus	2	0.006%
Methylopila sp. M107	2	0.006%
Mesoflavibacter zeaxanthinifaciens	2	0.006%
Brucella melitensis 548	2	0.006%
Theileria annulata strain Ankara	2	0.006%
Bacillus sp. SA2-6	2	0.006%
Ruminococcus torques L2-14	2	0.006%

Species	#BLAST Top-Hits	Percentage
Streptomyces sp. 351MFTsu5.1	2	0.006%
Acinetobacter baumannii	2	0.006%
Histoplasma capsulatum H143	2	0.006%
Streptomyces sp. MNU77	2	0.006%
Acidobacteriaceae bacterium KBS 96	2	0.006%
Bacillus selenatarsenatis SF-1	2	0.006%
Pseudomonas mandelii	2	0.006%
Shewanella loihica PV-4	2	0.006%
Campylobacter concisus	2	0.006%
Leisingera sp. ANG-M6	2	0.006%
Lachancea thermotolerans CBS 6340	2	0.006%
Pseudoalteromonas citrea DSM 8771	2	0.006%
Pseudomonas alcaligenes OT 69	2	0.006%
Neurospora crassa OR74A	2	0.006%
Clostridiales bacterium DRI-13	2	0.006%
Azospirillum brasilense	2	0.006%
Corynebacterium glutamicum	2	0.006%
Kibdelosporangium sp. MJ126-NF4	2	0.006%
Frankia sp. QA3	2	0.006%
Aphanizomenon flos-aquae	2	0.006%
Nocardia takedensis	2	0.006%
Pseudoalteromonas haloplanktis	2	0.006%
Agkistrodon piscivorus	2	0.006%
Aspergillus kawachii IFO 4308	2	0.006%
Burkholderia sp. RPE67	2	0.006%
Vibrio pacinii	2	0.006%
Plasmodium knowlesi strain H	2	0.006%
Pseudoalteromonas citrea	2	0.006%
Olsenella sp. oral taxon 809 str. F0356	2	0.006%
Rhodobacter sphaeroides	2	0.006%
Haliangium ochraceum DSM 14365	2	0.006%
Corynebacterium casei UCMA 3821	2	0.006%
Lactobacillus composti	2	0.006%
SAR86 cluster bacterium SAR86B	2	0.006%
Natronococcus amylolyticus DSM 10524	2	0.006%
Pseudomonas trivialis	2	0.006%
Pseudovibrio sp. FO-BEG1	2	0.006%
Glycomyces tenuis	2	0.006%
Persephonella sp. KM09-Lau-8	2	0.006%
Brevibacterium casei S18	2	0.006%
Bacillus ligniniphilus	2	0.006%
gamma proteobacterium HdN1	2	0.006%
Plasmodium vinckei vinckei	2	0.006%
Sulfuricella sp. T08	2	0.006%
Serendipita vermifera MAFF 305830	2	0.006%
Frateuria aurantia DSM 6220	2	0.006%
Trichophyton rubrum MR1459	2	0.006%
Thioalkalivibrio sp. ALJ16	2	0.006%
Lachnospiraceae bacterium AC2012	2	0.006%
Plasmodium falciparum NF54	2	0.006%
Theileria parva	2	0.006%
Algicola sagamiensis	2	0.006%
Streptacidiphilus oryzae	2	0.006%

Species	#BLAST Top-Hits	Percentage
Lachnospiraceae bacterium P6B14	2	0.006%
Bacillus sp. MT2	2	0.006%
Frankia sp. EuI1c	2	0.006%
Drosophila ananassae	2	0.006%
Burkholderia ambifaria	2	0.006%
Actinospica acidiphila	2	0.006%
Talaromyces islandicus	2	0.006%
Dacryopinax sp. DJM-731 SS1	2	0.006%
Microcystis aeruginosa PCC 9809	2	0.006%
Oceanithermus profundus	2	0.006%
Alicyclobacillus macrosporangiidus	2	0.006%
Synechococcus sp. KORDI-49	2	0.006%
Hydrocarboniphaga effusa	2	0.006%
Trichodesmium erythraeum	2	0.006%
Ketogulonicigenium vulgare WSH-001	2	0.006%
Pseudomonas stutzeri	2	0.006%
Nocardiopsis sp. RV163	2	0.006%
Pseudonocardia acaciae	2	0.006%
Thermopetrobacter sp. TC1	2	0.006%
Novosphingobium pentaromativorans	2	0.006%
Roseobacter sp. AzwK-3b	2	0.006%
Bacillus subtilis	2	0.006%
Leishmania donovani	2	0.006%
Corynebacterium glutamicum ATCC 14067	2	0.006%
Anaerotruncus sp. CAG:390	2	0.006%
Acute bee paralysis virus	2	0.006%
Brevibacillus panacihumi	2	0.006%
Gordonia sp. KTR9	2	0.006%
Aspergillus oryzae 3.042	2	0.006%
Microlunatus phosphovorus NM-1	2	0.006%
Microgenomates bacterium GW2011_GWC1_37_8	2	0.006%
Microbacterium oxydans	2	0.006%
Erythrobacter sp. NAP1	2	0.006%
Psychrobacter	2	0.006%
Methyloglobulus morosus KoM1	2	0.006%
Chthoniobacter flavus	2	0.006%
Candidatus Pelagibacter sp. IMCC9063	2	0.006%
Mycobacterium heraklionense	2	0.006%
Actinomyces georgiae F0490	2	0.006%
Aureimonas altamirensis	2	0.006%
Pseudomonas fluorescens PICF7	2	0.006%
Brevibacterium] flavum	2	0.006%
Methanoregula boonei	2	0.006%
Bacillus amyloliquefaciens	2	0.006%
Faecalibacterium sp. CAG:74	2	0.006%
Gelidibacter mesophilus	2	0.006%
Aliagarivorans taiwanensis	2	0.006%
Olsenella sp. oral taxon 809	2	0.006%
Mycobacterium chlorophenolicum	2	0.006%
Sclerotinia sclerotiorum 1980 UF-70	2	0.006%
Ruminococcus sp. CAG:55	2	0.006%
Agrobacterium	2	0.006%
Halocynthiibacter sp. PAMC 20958	2	0.006%

Species	#BLAST Top-Hits	Percentage
Streptomyces mirabilis	2	0.006%
Bacillus hemicellulosilyticus	2	0.006%
Candidatus Arthromitus sp. SFB-mouse-Yit	2	0.006%
Escherichia hermannii NBRC 105704	2	0.006%
Streptomyces sp. NRRL WC-3742	2	0.006%
Deinococcus maricopensis DSM 21211	2	0.006%
Edaphobacter aggregans	2	0.006%
Cyclobacterium marinum DSM 745	2	0.006%
Ruminococcus sp. CAG:90	2	0.006%
Halomonadaceae	2	0.006%
Leptospirillum ferriphilum	2	0.006%
Carnobacterium sp. ZWU0011	2	0.006%
Pelosinus fermentans JBW45	2	0.006%
Haloglycomyces albus	2	0.006%
Actinocatenispora sera	2	0.006%
uncultured Mediterranean phage uvMED	2	0.006%
Plasmodium fragile	2	0.006%
Hydrogenobaculum	2	0.006%
Delftia sp. Cs1-4	2	0.006%
Agaricus bisporus var. bisporus H97	2	0.006%
Enterobacter cancerogenus	2	0.006%
Streptomyces niveus	2	0.006%
Rheinheimera nanhaiensis	2	0.006%
Calothrix sp. PCC 7103	2	0.006%
Epilithonimonas lactis	2	0.006%
Kiloniella laminariae	2	0.006%
Streptomyces sp. AcH 505	2	0.006%
Aeromonas eucrenophila	2	0.006%
Thermus sp. CCB_US3_UF1	2	0.006%
Methyloglobulus morosus	2	0.006%
Synechococcus sp. WH 8016	2	0.006%
Byssochlamys spectabilis No. 5	2	0.006%
Solirubrobacterales bacterium URHD0059	2	0.006%
Debaryomyces hansenii CBS767	2	0.006%
Methylobacterium aquaticum	2	0.006%
Spongiibacter tropicus	2	0.006%
Novosphingobium sp. AP12	2	0.006%
Rikenella microfusus	2	0.006%
Candida albicans WO-1	2	0.006%
Chryseobacterium koreense CCUG 49689	2	0.006%
Corynebacterium	2	0.006%
Pantoea agglomerans	2	0.006%
Desulfonatronum thioautotrophicum	2	0.006%
Aspergillus niger CBS 513.88	2	0.006%
Campylobacter showae	2	0.006%
Bothrops pauloensis	2	0.006%
Desulfuromonas sp. WTL	2	0.006%
Delftia sp. ZNC0008	2	0.006%
Aspergillus terreus NIH2624	2	0.006%
Plasmodium vivax Sal-1	2	0.006%
Pseudomonas sp. URMO17WK12:I4	2	0.006%
Nautilia profundicola	2	0.006%
Arthrobacter nicotinovorans	2	0.006%

Species	#BLAST Top-Hits	Percentage
bacterium YEK0313	2	0.006%
Lactobacillus reuteri	2	0.006%
Acremonium chrysogenum ATCC 11550	2	0.006%
Coccidioides immitis RS	2	0.006%
Fusarium oxysporum f. sp. lycopersici 4287	2	0.006%
Sphaeroforma arctica JP610	2	0.006%
Gluconobacter frateurii	2	0.006%
Parasitella parasitica	2	0.006%
Chondromyces crocatus	2	0.006%
Drosophila mojavensis	2	0.006%
Campylobacter jejuni	2	0.006%
Brachybacterium muris	2	0.006%
Rhodospirillum centenum SW	2	0.006%
Haliangium ochraceum	2	0.006%
Thermosphaera aggregans DSM 11486	2	0.006%
Paenibacillus sp. FJAT-27812	2	0.006%
Malassezia globosa CBS 7966	2	0.006%
Methylococcaceae bacterium 73a	2	0.006%
Simiduia agarivorans SA1 = DSM 21679	2	0.006%
Thiobacillus denitrificans	2	0.006%
Pseudomonas mandelii JR-1	2	0.006%
Clavibacter michiganensis	2	0.006%
Ochrobactrum intermedium 229E	2	0.006%
Vibrio crassostreae	2	0.006%
Aequorivita sublithincola DSM 14238	2	0.006%
Candidatus Endolissoclinum faulkneri L2	2	0.006%
Bacteroides cellulosilyticus CAG:158	2	0.006%
Candidatus Pelagibacter ubique	2	0.006%
Nocardia thailandica	2	0.006%
Streptomyces venezuelae	2	0.006%
Comamonas badia	2	0.006%
Tetrapisispora blattae CBS 6284	2	0.006%
Streptomyces sp. NRRL F-2580	2	0.006%
Sphingomonas-like bacterium B12	2	0.006%
Draconibacterium sp. JN14CK-3	2	0.006%
Leishmania infantum JPCM5	2	0.006%
Schlesneria paludicola	2	0.006%
Sideroxydans lithotrophicus ES-1	2	0.006%
Ochrobactrum anthropi	2	0.006%
Thalassomonas viridans	2	0.006%
Beauveria bassiana ARSEF 2860	2	0.006%
Thauera sp. 28	2	0.006%
Brevibacillus panacihumi W25	2	0.006%
Monoraphidium neglectum	2	0.006%
Babesia equi	2	0.006%
Streptomyces sp. CT34	2	0.006%
Georgenia sp. SUBG003	2	0.006%
Bacillus niacini	2	0.006%
Aspergillus ruber CBS 135680	2	0.006%
Novosphingobium pentaromativorans US6-1	2	0.006%
Gloydius brevicaudus siniticus	2	0.006%
Pseudomonas syringae pv. cilantro	2	0.006%
Bacillus thuringiensis	2	0.006%

Species	#BLAST Top-Hits	Percentage
Sphingobium sp. KK22	2	0.006%
Syntrophomonas zehnderi OL-4	2	0.006%
Photorhabdus temperata J3	2	0.006%
Opitutaceae bacterium TAV1	2	0.006%
Butyrivibrio sp. NC2007	2	0.006%
Listeria monocytogenes str. 1/2a F6854	2	0.006%
Burkholderia sp. USM B20	2	0.006%
Sphingobacterium spiritivorum	2	0.006%
Thermodesulfobium narugense DSM 14796	2	0.006%
Thiomicrospira crunogena XCL-2	2	0.006%
Fusarium oxysporum f. sp. vasinfectum 25433	2	0.006%
Kluyvera cryocrescens	2	0.006%
Methyloversatilis universalis	2	0.006%
Collinsella sp. CAG:398	2	0.006%
Paenibacillus alginolyticus	2	0.006%
Verrucomicrobium sp. BvORR034	2	0.006%
Microbacterium profundi	2	0.006%
Pontibacter roseus	2	0.006%
Rheinheimera nanhaiensis E407-8	2	0.006%
Trichophyton interdigitale MR816	2	0.006%
Tsukamurella paurometabola DSM 20162	2	0.006%
Frankia sp. EUN1f	2	0.006%
Halorhodospira halophila	2	0.006%
Holdemania massiliensis	2	0.006%
Deinococcus deserti VCD115	2	0.006%
Aspergillus parasiticus SU-1	2	0.006%
Saccharothrix sp. ST-888	2	0.006%
Nocardia sp. BMG51109	2	0.006%
Epilithonimonas sp. FH1	2	0.006%
Caenispirillum salinarum	2	0.006%
Pichia etchellsii	2	0.006%
Anoxybacillus flavithermus	2	0.006%
Coccomyxa subellipsoidea C-169	2	0.006%
Chloroflexus	2	0.006%
Streptomyces sp. NRRL S-813	2	0.006%
Streptomyces sulphureus	2	0.006%
Hahella ganghwensis	2	0.006%
Phycicoccus jejuensis	2	0.006%
Kribbella flavida	2	0.006%
Achromobacter xylosoxidans	2	0.006%
Dyadobacter fermentans DSM 18053	2	0.006%
Morganella morganii	2	0.006%
Streptomyces davawensis	2	0.006%
Paenibacillus pinihumi	2	0.006%
Streptomyces pristinaespiralis ATCC 25486	2	0.006%
Pristionchus pacificus	2	0.006%
Micromonospora sp. M42	2	0.006%
Staphylococcus epidermidis	2	0.006%
Anaerophaga thermohalophila	2	0.006%
Myceliophthora thermophila ATCC 42464	2	0.006%
Rhodoferax saidenbachensis	2	0.006%
Leptolyngbya sp. KIOST-1	2	0.006%
Brevibacillus laterosporus	2	0.006%

Species	#BLAST Top-Hits	Percentage
Kitasatospora griseola	2	0.006%
Bdellovibrio exovorus JSS	2	0.006%
Klebsiella pneumoniae subsp. pneumoniae DSM 30104	2	0.006%
Lactobacillus brevis ATCC 14869 = DSM 20054	2	0.006%
Enterococcus phoeniculicola ATCC BAA-412	2	0.006%
Azospirillum lipoferum	2	0.006%
Synechococcus sp. WH 8102	2	0.006%
Sphaerotilus natans subsp. natans DSM 6575	2	0.006%
Bradyrhizobium japonicum	2	0.006%
Erysiphe necator	2	0.006%
Bacillus sonorensis L12	2	0.006%
Brachybacterium muris UCD-AY4	2	0.006%
Staphylococcus epidermidis BVS058A4	2	0.006%
Bradyrhizobium oligotrophicum	2	0.006%
Hydrogenobaculum sp. SHO	2	0.006%
Myroides odoratimimus CCUG 10230	1	0.003%
Granulicella tundricola MP5ACTX9	1	0.003%
Ehrlichia ruminantium str. Gardel	1	0.003%
Lysinibacillus sinduriensis	1	0.003%
Pseudomonas sp. URHB0015	1	0.003%
Streptomyces sp. NRRL F-5727	1	0.003%
Clostridium celatum DSM 1785	1	0.003%
Polaromonas sp. JS666	1	0.003%
Mycobacterium vanbaalenii PYR-1	1	0.003%
Lactobacillus equi DPC 6820	1	0.003%
Roseobacter litoralis Och 149	1	0.003%
Thermoanaerobaculum aquaticum	1	0.003%
Ruminococcus] gnavus	1	0.003%
Butyrivibrio sp. XPD2006	1	0.003%
Crotalus ruber exsul	1	0.003%
Bifidobacterium angulatum DSM 20098 = JCM 7096	1	0.003%
Streptomyces sp. 769	1	0.003%
Vibrio sp. J2-17	1	0.003%
Ramlibacter tataouinensis TTB310	1	0.003%
Frankia sp. CN3	1	0.003%
Halobacillus halophilus	1	0.003%
Aquaspirillum serpens	1	0.003%
Fibrisoma limi	1	0.003%
Paenibacillus sp. VT-400	1	0.003%
Escherichia coli KOEGE 118 (317a)	1	0.003%
Serratia marcescens BIDMC 44	1	0.003%
Klebsiella variicola CAG:634	1	0.003%
Gemmatimonas phototrophica	1	0.003%
Vibrio sp. B183	1	0.003%
Anaerococcus senegalensis	1	0.003%
Lolium perenne	1	0.003%
Devosia sp. H5989	1	0.003%
Pseudomonas syringae pv. actinidiae ICMP 19102	1	0.003%
Euglenaformis proxima	1	0.003%
Pseudomonas syringae pv. actinidiae ICMP 19101	1	0.003%
Parcubacteria bacterium C7867-001	1	0.003%
Listeria fleischmannii	1	0.003%
Odoribacter sp. CAG:788	1	0.003%

Species	#BLAST Top-Hits	Percentage
Hippea maritima	1	0.003%
Leadbetterella byssophila	1	0.003%
Xanthomonas albilineans	1	0.003%
Dehalobacter sp. FTH1	1	0.003%
Leuconostoc citreum LBAE C10	1	0.003%
Devosia subaequoris	1	0.003%
Pseudomonas syringae pv. actinidiae ICMP 19104	1	0.003%
Butyrivibrio sp. AE2015	1	0.003%
Pseudomonas syringae pv. actinidiae ICMP 19103	1	0.003%
Salmonella enterica subsp. enterica serovar Typhimurium str. L-3553	1	0.003%
Leifsonia xyli	1	0.003%
Bartonella alsatica	1	0.003%
Spirochaeta cellobiosiphila	1	0.003%
Thauera linaloolentis	1	0.003%
Burkholderia phytofirmans PsJN	1	0.003%
Bacillus methylotrophicus	1	0.003%
Afroedura broadleyi	1	0.003%
Lactobacillus florum	1	0.003%
Ferrimonas balearica DSM 9799	1	0.003%
Desulfovibrio sp. TomC	1	0.003%
butyrate-producing bacterium SS3/4	1	0.003%
Anaerotruncus colihominis	1	0.003%
Parcubacteria bacterium GW2011_GWA1_59_11	1	0.003%
Rickettsia japonica	1	0.003%
Zetaproteobacteria bacterium TAG-1	1	0.003%
Fusobacterium sp. CM21	1	0.003%
Photobacterium angustum S14	1	0.003%
Natronolimnobius innermongolicus	1	0.003%
Paenibacillus forsythiae	1	0.003%
Novosphingobium subterraneum	1	0.003%
Lactobacillus jensenii 115-3-CHN	1	0.003%
Streptomyces sp. NRRL F-5755	1	0.003%
Arthrobacter sp. MA-N2	1	0.003%
Candidatus Hamiltonella defensa	1	0.003%
Pseudorhodobacter wandonensis	1	0.003%
Streptomyces sp. MUSC 125	1	0.003%
Streptomyces cyaneogriseus	1	0.003%
Streptomyces iranensis	1	0.003%
Rhizobium sp. 42MFCr.1	1	0.003%
Thiomonas intermedia	1	0.003%
Chryseobacterium sp. YR561	1	0.003%
Desulfurispirillum indicum S5	1	0.003%
Desulfosporosinus acidiphilus SJ4	1	0.003%
Amycolatopsis methanolica 239	1	0.003%
Bdellovibrio bacteriovorus str. Tiberius	1	0.003%
Paenibacillus pasadenensis	1	0.003%
Necator americanus	1	0.003%
Kitasatospora setae	1	0.003%
Pseudomonas sp. 15AGV	1	0.003%
Prochlorococcus marinus str. MIT 9123	1	0.003%
Serratia sp. ATCC 39006	1	0.003%
Enterobacteria phage Min27	1	0.003%
Paracoccidioides brasiliensis Pb03	1	0.003%

Species	#BLAST Top-Hits	Percentage
Clostridium] hiranonis	1	0.003%
Enterobacter asburiae	1	0.003%
Loktanella vestfoldensis	1	0.003%
Flavobacterium tegetincola	1	0.003%
Treponema pedis	1	0.003%
Crotalus molossus	1	0.003%
Streptomyces caelestis	1	0.003%
Providencia alcalifaciens PAL-2	1	0.003%
Prochlorococcus marinus str. MIT 9116	1	0.003%
Vibrio ichthyoenteri	1	0.003%
Streptomyces sp. CNT360	1	0.003%
Mus musculus domesticus	1	0.003%
Kitasatospora cheerisanensis	1	0.003%
Rhodomicrobium vannielii ATCC 17100	1	0.003%
Intestinimonas sp. GD2	1	0.003%
Simian retrovirus 4	1	0.003%
Magnetospirillum magneticum AMB-1	1	0.003%
Microgenomates bacterium GW2011_GWA1_48_10	1	0.003%
candidate division TM6 bacterium GW2011_GWA2_36_9	1	0.003%
Comamonadaceae bacterium URHA0028	1	0.003%
Streptomyces katrae	1	0.003%
Mycobacterium gilvum	1	0.003%
Prochlorococcus marinus str. MIT 9107	1	0.003%
Sterolibacterium denitrificans	1	0.003%
Lactobacillus ultunensis DSM 16047	1	0.003%
Paenibacillus sp. URHA0014	1	0.003%
Cryptococcus gattii R265	1	0.003%
Methylobacterium	1	0.003%
Burkholderia sp. CCGE1002	1	0.003%
Python nidovirus	1	0.003%
Streptococcus sinensis	1	0.003%
Oscillatoriales cyanobacterium JSC-12	1	0.003%
Synchiropus agassizii	1	0.003%
Bacteroides intestinalis	1	0.003%
Mytilus galloprovincialis	1	0.003%
Methanosarcina vacuolata Z-761	1	0.003%
Paracoccidioides brasiliensis Pb18	1	0.003%
Methylobacterium tarhaniae	1	0.003%
Sphingopyxis baekryungensis	1	0.003%
Pontibacillus chungwhensis BH030062	1	0.003%
Vibrio maritimus	1	0.003%
Pseudogymnoascus pannorum VKM F-4246	1	0.003%
Tegillarca granosa	1	0.003%
Devosia sp. LC5	1	0.003%
Microgenomates (Roizmanbacteria) bacterium GW2011_GWC2_35_12	1	0.003%
Hyphomonas adhaerens	1	0.003%
Thermomicrobium roseum	1	0.003%
Demansia vestigiata	1	0.003%
Nocardiopsis xinjiangensis	1	0.003%
Rhodococcus rhodnii LMG 5362	1	0.003%
Lethenteron camtschaticum	1	0.003%
Gryllus assimilis	1	0.003%
Mesorhizobium plurifarium	1	0.003%

Species	#BLAST Top-Hits	Percentage
Blastococcus saxobsidens	1	0.003%
Spiroplasma sabaudiense	1	0.003%
Bacillus aquimaris	1	0.003%
Nocardiopsis chromatogenes	1	0.003%
Sphingomonas sp. BHC-A	1	0.003%
Salinivibrio sp. KP-1	1	0.003%
Bradyrhizobium sp. ARR65	1	0.003%
Streptomyces ahygroscopicus	1	0.003%
Actinomadura atramentaria	1	0.003%
Yersinia	1	0.003%
Clostridium] straminisolvens JCM 21531	1	0.003%
Propionibacterium acnes HL030PA2	1	0.003%
Streptomyces sp. PCS3-D2	1	0.003%
Pseudomonas japonica	1	0.003%
Porphyromonas uenonis	1	0.003%
Lactobacillus johnsonii	1	0.003%
Aeromonas media	1	0.003%
Vibrio sp. J2-29	1	0.003%
Campylobacter iguaniorum	1	0.003%
Arsukibacterium sp. MJ3	1	0.003%
Escherichia coli ED1a	1	0.003%
Microgenomates (Woesebacteria) bacterium GW2011_GWA2_44_33	1	0.003%
Stemphylium lycopersici	1	0.003%
Actinoplanes rectilineatus	1	0.003%
Alicycliphilus denitrificans K601	1	0.003%
Bifidobacterium bifidum S17	1	0.003%
Paracoccus sp. TRP	1	0.003%
Protobothrops maolanensis	1	0.003%
Rhodococcus sp. R04	1	0.003%
Mycobacterium sp. TKK-01-0059	1	0.003%
Rhizobium sp. IRBG74	1	0.003%
Streptococcus ferus	1	0.003%
Methylovulum miyakonense	1	0.003%
Streptomyces venezuelae ATCC 10712	1	0.003%
Verrucomicrobia bacterium IMCC26134	1	0.003%
Thioalkalivibrio sulfidiphilus	1	0.003%
Bacillus cereus VD140	1	0.003%
Tsukamurella paurometabola	1	0.003%
Lepidochelys olivacea	1	0.003%
Staphylococcus simulans	1	0.003%
Paraprevotella xylaniphila YIT 11841	1	0.003%
Ensifer sojae	1	0.003%
Acanthamoeba culbertsoni	1	0.003%
Pseudoalteromonas sp. NW 4327	1	0.003%
Oscillibacter valericigenes	1	0.003%
Agrococcus pavilionensis RW1	1	0.003%
Leptospirillum ferrooxidans C2-3	1	0.003%
Cardiobacterium valvarum	1	0.003%
Bartonella alsatica IBS 382	1	0.003%
Nodularia spumigena CCY9414	1	0.003%
Hydrogenophaga intermedia	1	0.003%
Corynebacterium imitans	1	0.003%
Cupriavidus gilardii CR3	1	0.003%

Species	#BLAST Top-Hits	Percentage
Pseudomonas sp. 20_BN	1	0.003%
Prevotella bryantii B14	1	0.003%
Paenibacillus panacisoli	1	0.003%
Microbacterium testaceum	1	0.003%
Helicobacter pylori Hp H-42	1	0.003%
Clostridium ultunense	1	0.003%
Mycobacterium abscessus 21	1	0.003%
Afipia sp. P52-10	1	0.003%
Clostridium aceticum	1	0.003%
Fusarium langsethiae	1	0.003%
Pleomorphomonas oryzae	1	0.003%
Stanieria cyanosphaera PCC 7437	1	0.003%
Acidiphilium cryptum JF-5	1	0.003%
Halobacillus halophilus DSM 2266	1	0.003%
Mycoplasma sp. CAG:472	1	0.003%
Tetragenococcus muriaticus 3MR10-3	1	0.003%
Histophilus somni	1	0.003%
Rubritalea marina	1	0.003%
Burkholderia nodosa	1	0.003%
Candidatus Tachikawaea gelatinosa	1	0.003%
marine gamma proteobacterium HTCC2207	1	0.003%
Geobacter lovleyi	1	0.003%
Drosophila sp. n. 'large spots'	1	0.003%
Atopobium sp. oral taxon 810	1	0.003%
Aureococcus anophagefferens	1	0.003%
Dokdonia sp. PRO95	1	0.003%
Rickettsia hoogstraalii str. RCCE3	1	0.003%
Dehalobacter sp. CF	1	0.003%
Sphingobium quisquiliarum	1	0.003%
Bradyrhizobium sp. Ec3.3	1	0.003%
Sporolactobacillus terrae	1	0.003%
Chloracidobacterium thermophilum	1	0.003%
Tyzzerella nexilis DSM 1787	1	0.003%
Entamoeba histolytica KU27	1	0.003%
Desulfobulbus sp. Tol-SR	1	0.003%
Bacillus nealsonii	1	0.003%
Hungatella hathewayi WAL-18680	1	0.003%
Paracoccus aminophilus	1	0.003%
Bacteroides finegoldii	1	0.003%
Mesotoga infera	1	0.003%
Curtobacterium sp. B18	1	0.003%
Effusibacillus pohliae	1	0.003%
Leifsonia sp. 109	1	0.003%
Desulfocapsa sulfexigens	1	0.003%
Marinococcus halotolerans	1	0.003%
Rhodanobacter denitrificans	1	0.003%
Kyrpidia tusciae	1	0.003%
Pusillimonas noertemannii	1	0.003%
Nocardiopsis dassonvillei	1	0.003%
Pseudomonas fluorescens EGD-AQ6	1	0.003%
Corynebacterium sputi	1	0.003%
Ochrobactrum sp. CDB2	1	0.003%
Pectobacterium carotovorum subsp. brasiliensis ICMP 19477	1	0.003%

Species	#BLAST Top-Hits	Percentage
Bacteroides reticulotermitis	1	0.003%
Tistrella mobilis	1	0.003%
Alcanivorax dieselolei	1	0.003%
Serratia fonticola	1	0.003%
Halorubrum coriense DSM 10284	1	0.003%
Arcobacter sp. L	1	0.003%
Rhodopirellula maiorica	1	0.003%
Methylocystis sp. SC2	1	0.003%
Thermococcus kodakarensis KOD1	1	0.003%
Fischerella muscicola	1	0.003%
Dysgonomonas gadei	1	0.003%
Acetobacter tropicalis	1	0.003%
Novosphingobium tardaugens	1	0.003%
Pantoea stewartii	1	0.003%
Bacillus sp. 123MFChir2	1	0.003%
Toxoplasma gondii VEG	1	0.003%
Erwinia pyrifoliae	1	0.003%
Coccidioides immitis RMSCC 3703	1	0.003%
Nocardia asiatica	1	0.003%
Mycobacterium ulcerans Agy99	1	0.003%
Streptomyces sp. ATexAB-D23	1	0.003%
Helicobacter muridarum	1	0.003%
Acinetobacter sp. P8-3-8	1	0.003%
Clostridium] termitidis	1	0.003%
Lactobacillus delbrueckii subsp. bulgaricus PB2003/044-T3-4	1	0.003%
Stanieria cyanosphaera	1	0.003%
Chlamydophila felis	1	0.003%
Mycobacterium sp. UNC410CL29Cvi84	1	0.003%
Coxiella endosymbiont of Amblyomma americanum	1	0.003%
Clostridium leptum CAG:27	1	0.003%
Akkermansia muciniphila	1	0.003%
actinobacterium SCGC AAA027-L06	1	0.003%
Oscillibacter sp. CAG:241	1	0.003%
Microbotryum violaceum p1A1 Lamole	1	0.003%
Flavobacterium sp. KMS	1	0.003%
Acinetobacter oleivorans DR1	1	0.003%
Pseudogymnoascus pannorum VKM F-103	1	0.003%
Anaeromyxobacter dehalogenans 2CP-C	1	0.003%
Rhodopseudomonas sp. B29	1	0.003%
Actinosynnema mirum	1	0.003%
Candida albicans P78042	1	0.003%
Bacillus okuhidensis	1	0.003%
Bacteroides salyersiae WAL 10018 = DSM 18765 = JCM 12988	1	0.003%
Trichophyton rubrum D6	1	0.003%
Giardia lamblia P15	1	0.003%
Rhizobium gallicum bv. gallicum R602	1	0.003%
Niastella koreensis	1	0.003%
Candidatus Jettenia caeni	1	0.003%
Escherichia fergusonii	1	0.003%
Kordia jejudonensis	1	0.003%
Paenibacillus dendritiformis C454	1	0.003%
Nocardioides sp. J54	1	0.003%
Bacteroides salyersiae CL02T12C01	1	0.003%

Species	#BLAST Top-Hits	Percentage
Fusarium pseudograminearum CS5834	1	0.003%
Blackberry vein banding associated virus	1	0.003%
Streptomyces sp. NRRL F-525	1	0.003%
Pseudomonas aeruginosa PA38182	1	0.003%
Thioflavicoccus mobilis 8321	1	0.003%
Pseudomonas sp. ES3-33	1	0.003%
Megavirus courdo7	1	0.003%
Fusobacterium sp. CAG:815	1	0.003%
Oscillospiraceae bacterium VE202-24	1	0.003%
Yersinia intermedia	1	0.003%
Starkeya novella DSM 506	1	0.003%
Paenibacillus terrae HPL-003	1	0.003%
Butyrivibrio fibrisolvens 16/4	1	0.003%
Flavobacterium columnare	1	0.003%
Pasteurella multocida subsp. multocida	1	0.003%
Halorubrum ezzemoulense	1	0.003%
Nematocida sp. 1 ERTm6	1	0.003%
Ideonella sp. B508-1	1	0.003%
Halomonas sp. TD01	1	0.003%
Thioalkalivibrio sp. ALJ7	1	0.003%
Legionella longbeachae D-4968	1	0.003%
uncultured bacterium HF130_12L15	1	0.003%
Mycoplasma bovoculi M165/69	1	0.003%
Segniliparus rotundus	1	0.003%
Mephitis mephitis	1	0.003%
Clostridium] clostridioforme 90A7	1	0.003%
Bradyrhizobium	1	0.003%
Dorea longicatena DSM 13814	1	0.003%
Clostridium homopropionicum	1	0.003%
Bacillus cereus ATCC 10876	1	0.003%
Escherichia coli O104:H4 str. Ec11-5604	1	0.003%
Actinoplanes missouriensis 431	1	0.003%
Thiorhodospira sibirica ATCC 700588	1	0.003%
Arenibacter certesii	1	0.003%
Chitinivibrio alkaliphilus	1	0.003%
Leptospira interrogans str. HAI1594	1	0.003%
Virgibacillus alimentarius	1	0.003%
Pseudomonas aeruginosa C48	1	0.003%
Methanobacterium sp. Maddingley MBC34	1	0.003%
Butyrivibrio proteoclasticus B316	1	0.003%
Neisseria subflava NJ9703	1	0.003%
Loktanella vestfoldensis SKA53	1	0.003%
Gardnerella vaginalis 75712	1	0.003%
Methylobacterium nodulans ORS 2060	1	0.003%
Leifsonia xyli subsp. cynodontis DSM 46306	1	0.003%
Treponema pallidum subsp. pallidum str. Chicago	1	0.003%
Bordetella bronchiseptica	1	0.003%
Sodalis praecaptivus	1	0.003%
Euglena gracilis	1	0.003%
Pedobacter sp. R20-19	1	0.003%
uncultured gamma proteobacterium EB000_65A11	1	0.003%
Fischerella sp. PCC 9605	1	0.003%
Synechococcus sp. CC9311	1	0.003%

Species	#BLAST Top-Hits	Percentage
Blastomyces dermatitidis SLH14081	1	0.003%
Mycobacterium goodii	1	0.003%
Streptomyces sp. WM4235	1	0.003%
Streptomyces sp. NRRL S-1813	1	0.003%
Campylobacter showae CSUNSWCD	1	0.003%
Clostridium sp. L2-50	1	0.003%
Clostridium sp. CAG:62	1	0.003%
Streptomyces sp. NRRL B-1347	1	0.003%
Paraglaciecola polaris LMG 21857	1	0.003%
marine gamma proteobacterium HTCC2080	1	0.003%
Legionella fallonii LLAP-10	1	0.003%
Alicycliphilus denitrificans	1	0.003%
Mobiluncus curtisii	1	0.003%
Amycolatopsis thermoflava	1	0.003%
Acidithiobacillus sp. GGI-221	1	0.003%
Clostridium] clostridioforme	1	0.003%
Clostridium homopropionicum DSM 5847	1	0.003%
Chryseobacterium sp. YR460	1	0.003%
Pleomorphomonas koreensis	1	0.003%
Gardnerella vaginalis JCP8481B	1	0.003%
Gardnerella vaginalis JCP8481A	1	0.003%
Clostridium sp. CAG:75	1	0.003%
Agrobacterium radiobacter K84	1	0.003%
Ruegeria sp. ANG-S4	1	0.003%
Clostridiales bacterium NK3B98	1	0.003%
Cryptococcus gattii WM276	1	0.003%
Johnsonella ignava	1	0.003%
Pseudomonas extremaustralis	1	0.003%
Butyrivibrio sp. MC2013	1	0.003%
Roseobacter denitrificans OCh 114	1	0.003%
Brevibacillus sp. BC25	1	0.003%
Pseudomonas stutzeri KOS6	1	0.003%
Komagataeibacter medellinensis NBRC 3288	1	0.003%
Deinococcus marmoris	1	0.003%
Acidiphilium cryptum	1	0.003%
Thermobacillus composti KWC4	1	0.003%
Nitrococcus mobilis Nb-231	1	0.003%
Butyrivibrio sp. MC2021	1	0.003%
Marichromatium purpuratum	1	0.003%
Thalassospira profundimaris WP0211	1	0.003%
Algibacter lectus	1	0.003%
Comamonadaceae bacterium H1	1	0.003%
Austrelaps labialis	1	0.003%
Hepatitis C virus	1	0.003%
Lactobacillus nodensis	1	0.003%
Escherichia coli 2-156-04_S1_C1	1	0.003%
Brevundimonas sp. EAKA	1	0.003%
Acanthamoeba castellanii str. Neff	1	0.003%
Alcanivorax sp. P2S70	1	0.003%
Escherichia coli 2-156-04_S1_C2	1	0.003%
Atopobium sp. oral taxon 810 str. F0209	1	0.003%
Dorea sp. 5-2	1	0.003%
Flavobacterium	1	0.003%

Species	#BLAST Top-Hits	Percentage
Weissella koreensis	1	0.003%
Acetobacter syzygii 9H-2	1	0.003%
Limnohabitans planktonicus	1	0.003%
Lachnospiraceae bacterium 5_1_57FAA	1	0.003%
Sporosarcina newyorkensis	1	0.003%
Marinobacter algicola DG893	1	0.003%
Staphylococcus arlettae CVD059	1	0.003%
Paenibacillus sabinae T27	1	0.003%
Pseudomonas bauzanensis	1	0.003%
Serratia multitudinisentens RB-25	1	0.003%
Toxoplasma gondii GAB2-2007-GAL-DOM2	1	0.003%
Arthrobacter sp. Hiyo4	1	0.003%
Ahrensia sp. R2A130	1	0.003%
Peptococcaceae bacterium CEB3	1	0.003%
Catellicoccus marimammalium	1	0.003%
Lentisphaera araneosa HTCC2155	1	0.003%
Causus defilippii	1	0.003%
Burkholderia sprentiae	1	0.003%
Shinella sp. GWS1	1	0.003%
Erysipelotrichaceae bacterium NK3D112	1	0.003%
Bacillus massiliogorillae	1	0.003%
Bacteroides uniformis dnLKV2	1	0.003%
Treponema pallidum subsp. pallidum str. Mexico A	1	0.003%
Shewanella pealeana	1	0.003%
Pseudomonas alcaliphila	1	0.003%
Balneola vulgaris	1	0.003%
Amycolatopsis benzoatilytica	1	0.003%
Prevotella buccae D17	1	0.003%
Streptomyces sp. NRRL S-1824	1	0.003%
Streptococcus equi subsp. zooepidemicus Sz57	1	0.003%
Rhizophagus irregularis DAOM 181602	1	0.003%
Methylosinus trichosporium	1	0.003%
Triticum aestivum	1	0.003%
Thiorhodococcus drewsii AZ1	1	0.003%
Bifidobacterium dentium	1	0.003%
Mycobacterium abscessus 5S-0921	1	0.003%
Streptomyces sp. NRRL S-237	1	0.003%
Treponema pallidum subsp. pallidum SS14	1	0.003%
Clostridium saccharoperbutylacetonicum N1-4(HMT)	1	0.003%
Chitinibacter tainanensis	1	0.003%
Clostridium bolteae CAG:59	1	0.003%
Sinorhizobium sp. CCBAU 05631	1	0.003%
Bacillus fordii	1	0.003%
Halomicrobium katesii	1	0.003%
Gordonia shandongensis	1	0.003%
Salinibacillus aidingensis	1	0.003%
Ardenticatena maritima	1	0.003%
Xanthomonas arboricola pv. celebensis	1	0.003%
Mycobacterium cosmeticum	1	0.003%
Streptococcus equi subsp. zooepidemicus Sz35	1	0.003%
Lautropia mirabilis ATCC 51599	1	0.003%
Aquitalea magnusonii	1	0.003%
Hydrogenophaga sp. PBC	1	0.003%

Species	#BLAST Top-Hits	Percentage
Alistipes finegoldii CAG:68	1	0.003%
Caloplaca variabilis	1	0.003%
Bacteroides pyogenes F0041	1	0.003%
Chthonomonas calidirosea	1	0.003%
Novosphingobium sp. P6W	1	0.003%
Acinetobacter bohemicus ANC 3994	1	0.003%
halophilic archaeon DL31	1	0.003%
Paramecium bursaria chlorella virus MT325	1	0.003%
Enterococcus faecium E1039	1	0.003%
Nitrobacter hamburgensis	1	0.003%
Synechococcus sp. PCC 6312	1	0.003%
Desulfobacter postgatei	1	0.003%
Methanoculleus sp. CAG:1088	1	0.003%
Komagataeibacter intermedius AF2	1	0.003%
Geobacillus sp. WSUCF1	1	0.003%
Fimbriimonas ginsengisoli Gsoil 348	1	0.003%
Ralstonia sp. PBA	1	0.003%
Streptococcus mitis	1	0.003%
Rhizoctonia solani AG-3 Rhs1AP	1	0.003%
Escherichia coli UMEA 4076-1	1	0.003%
Foot-and-mouth disease virus - type C	1	0.003%
Burkholderia sp. MR1	1	0.003%
Agromyces italicus	1	0.003%
Rothia dentocariosa	1	0.003%
Propionicicella superfundia	1	0.003%
Microcystis aeruginosa PCC 9432	1	0.003%
Streptomyces roseochromogenes subsp. oscitans DS 12.976	1	0.003%
Halomonas sp. TG39a	1	0.003%
Bacillus cellulosilyticus	1	0.003%
Novosphingobium tardaugens NBRC 16725	1	0.003%
Trichophyton rubrum CBS 289.86	1	0.003%
Asaia platycodi SF2.1	1	0.003%
Thermanaerovibrio velox	1	0.003%
Wolbachia endosymbiont of Glossina morsitans morsitans	1	0.003%
Geobacter sp. M18	1	0.003%
Rhodococcus sp. UNC363MFTsu5.1	1	0.003%
Providencia alcalifaciens R90-1475	1	0.003%
Isosphaera pallida	1	0.003%
Pseudomonas alkylphenolia	1	0.003%
beta proteobacterium CB	1	0.003%
Aureobasidium namibiae CBS 147.97	1	0.003%
Vanderwaltozyma polyspora DSM 70294	1	0.003%
Rhodopseudomonas palustris BisB18	1	0.003%
Pontibacillus chungwhensis	1	0.003%
Photobacterium aphoticum	1	0.003%
Haemophilus haemolyticus M21639	1	0.003%
Megasphaera sp. NM10	1	0.003%
Azohydromonas australica	1	0.003%
Prevotella melaninogenica	1	0.003%
Rhizobium sp. MGL06	1	0.003%
Maribacter thermophilus	1	0.003%
Escherichia coli UCI 65	1	0.003%
Botrytis cinerea T4	1	0.003%

Species	#BLAST Top-Hits	Percentage
Mogibacterium timidum ATCC 33093	1	0.003%
Alkalilimnicola ehrlichii MLHE-1	1	0.003%
Thermus sp. 2.9	1	0.003%
Spirosoma spitsbergense	1	0.003%
Wuchereria bancrofti	1	0.003%
Listeria booriae	1	0.003%
Nocardia vulneris	1	0.003%
Candida tenuis ATCC 10573	1	0.003%
Pontibacter korlensis	1	0.003%
Komagataeibacter intermedius	1	0.003%
Actinobacteria	1	0.003%
Collinsella sp. CAG:166	1	0.003%
Hippopotamus amphibius	1	0.003%
Massilia alkalitolerans	1	0.003%
Parascardovia denticolens	1	0.003%
Acidovorax sp. NO-1	1	0.003%
Catenovulum agarivorans DS-2	1	0.003%
Parcubacteria (Giovannonibacteria) bacterium GW2011_GWA2_44_13b	1	0.003%
Chromohalobacter salexigens	1	0.003%
Thalassobacter stenotrophicus	1	0.003%
Chryseobacterium sp. BLS98	1	0.003%
Sphingobium sp. AP49	1	0.003%
Citrobacter sp. CIP 55.13	1	0.003%
Rhodobacter capsulatus	1	0.003%
Paenibacillus elgii	1	0.003%
Capnocytophaga ochracea DSM 7271	1	0.003%
Rhodococcus defluvii	1	0.003%
Paenibacillus mucilaginosus K02	1	0.003%
Thermococcus litoralis DSM 5473	1	0.003%
Bacillus sp. FJAT-14578	1	0.003%
Arabis alpina	1	0.003%
Paenibacillus sanguinis	1	0.003%
Burkholderia phytofirmans	1	0.003%
Oceanibaculum indicum	1	0.003%
Streptomyces avicenniae	1	0.003%
Haemophilus haemolyticus	1	0.003%
Fibrobacter succinogenes subsp. succinogenes S85	1	0.003%
Paenibacillus sp. FSL H8-457	1	0.003%
Lacerta agilis	1	0.003%
Terrabacter sp. 28	1	0.003%
Pseudomonas sp. QTF5	1	0.003%
Diplosphaera colitermitum	1	0.003%
Nocardia sp. 348MFTsu5.1	1	0.003%
Kibdelosporangium aridum	1	0.003%
Marinomonas posidonica	1	0.003%
Allochromatium vinosum	1	0.003%
Mycobacterium gilvum PYR-GCK	1	0.003%
Micromonospora aurantiaca	1	0.003%
Yersinia mollaretii	1	0.003%
Histoplasma capsulatum H88	1	0.003%
Cronobacter phage Dev-CD-23823	1	0.003%
Escherichia coli 2-177-06_S4_C3	1	0.003%
Fusobacterium varium	1	0.003%

Species	#BLAST Top-Hits	Percentage
Capnocytophaga gingivalis	1	0.003%
Thalassobacter	1	0.003%
Candidatus Arthromitus	1	0.003%
Alistipes obesi	1	0.003%
cyanobacterium PCC 7702	1	0.003%
Aeromonas enteropelogenes	1	0.003%
Clostridium novyi A str. NCTC 538	1	0.003%
Sulfurospirillum barnesii SES-3	1	0.003%
Oceanospirillum beijerinckii	1	0.003%
Acidocella	1	0.003%
Catla catla	1	0.003%
Coprococcus sp. ART55/1	1	0.003%
Enterobacter sp. GN02548	1	0.003%
Streptomyces sp. XY152	1	0.003%
Aspergillus clavatus NRRL 1	1	0.003%
Anaeromyxobacter sp. PSR-1	1	0.003%
Burkholderia sp. KJ006	1	0.003%
Leptospira meyeri serovar Semaranga str. Veldrot Semarang 173	1	0.003%
Bacillus halodurans C-125	1	0.003%
Colletotrichum graminicola M1.001	1	0.003%
Coriobacterium glomerans	1	0.003%
Paramecium bursaria Chlorella virus AP110A	1	0.003%
Gordonia terrae NBRC 100016	1	0.003%
Paraprevotella xylaniphila	1	0.003%
Geobacter sp. M21	1	0.003%
Agrobacterium tumefaciens CCNWGS0286	1	0.003%
Skermanella aerolata KACC 11604	1	0.003%
Candidatus Burkholderia verschuerenii	1	0.003%
Enterococcus faecium	1	0.003%
Yersinia enterocolitica IP 10393	1	0.003%
Propionibacterium thoenii	1	0.003%
Flavobacterium sp. JRM	1	0.003%
Polaromonas naphthalenivorans CJ2	1	0.003%
Rhodococcus equi ATCC 33707	1	0.003%
Rhodopirellula sallentina	1	0.003%
Streptomyces coelicolor A3(2)	1	0.003%
Laribacter hongkongensis HLHK9	1	0.003%
Methylibium sp. T29-B	1	0.003%
Roseomonas	1	0.003%
Rhizobium mesoamericanum STM3625	1	0.003%
Saccharomonospora viridis	1	0.003%
Citrobacter amalonaticus	1	0.003%
Oscillatoria] sp. PCC 6506	1	0.003%
Nocardia asteroides NBRC 15531	1	0.003%
Holdemania filiformis	1	0.003%
Ponticoccus sp. UMTAT08	1	0.003%
Dickeya sp. 2B12	1	0.003%
Roseobacter denitrificans	1	0.003%
Propionibacterium sp. KPL2005	1	0.003%
Methylotenera sp. L2L1	1	0.003%
Toscana virus	1	0.003%
Propionibacterium sp. KPL2000	1	0.003%
Aeromonas rivuli	1	0.003%

Species	#BLAST Top-Hits	Percentage
gamma proteobacterium IMCC3088	1	0.003%
Spirosoma linguale DSM 74	1	0.003%
Burkholderia sp. WSM4176	1	0.003%
Roseovarius mucosus DSM 17069	1	0.003%
Roseovarius sp. MCTG156(2b)	1	0.003%
Rhizobium phaseoli	1	0.003%
Sneathia sp. Sn35	1	0.003%
Candidatus Photodesmus katoptron	1	0.003%
Paenibacillus borealis	1	0.003%
Blastochloris viridis	1	0.003%
Pseudomonas alcaligenes NBRC 14159	1	0.003%
Sutterella wadsworthensis	1	0.003%
Terriglobus sp. TAA 43	1	0.003%
Microgenomates (Levybacteria) bacterium GW2011_GWA2_40_8	1	0.003%
Geobacter pickeringii	1	0.003%
Photorhabdus asymbiotica	1	0.003%
Ruegeria conchae	1	0.003%
Rhantus andinus	1	0.003%
Streptomyces yerevanensis	1	0.003%
Scytonema tolypothrichoides	1	0.003%
Pseudooceanicola atlanticus	1	0.003%
Chloroherpeton thalassium	1	0.003%
Parabacteroides goldsteinii	1	0.003%
Azonexus hydrophilus	1	0.003%
Diaporthe ampelina	1	0.003%
Clostridium sulfidigenes	1	0.003%
Thiolapillus brandeum	1	0.003%
Bacteroides sp. CAG:443	1	0.003%
Bactrocera dorsalis	1	0.003%
Porphyrobacter sp. HL-46	1	0.003%
Psychroserpens mesophilus	1	0.003%
Campylobacter showae RM3277	1	0.003%
Sphingomonas sanxanigenens DSM 19645 = NX02	1	0.003%
Streptomyces incarnatus	1	0.003%
Trichophyton soudanense CBS 452.61	1	0.003%
Thioalkalivibrio sp. AKL7	1	0.003%
Capnocytophaga gingivalis ATCC 33624	1	0.003%
Lyngbya aestuarii	1	0.003%
Yarrowia lipolytica	1	0.003%
Pyrobaculum islandicum	1	0.003%
Xenorhabdus griffiniae	1	0.003%
Bacillus psychrosaccharolyticus	1	0.003%
Pedobacter arcticus	1	0.003%
Sulfuricella denitrificans	1	0.003%
Thermococcus eurythermalis	1	0.003%
Actinomyces sp. HPA0247	1	0.003%
Acidocella aminolytica 101 = DSM 11237	1	0.003%
Streptomyces xiamenensis	1	0.003%
Legionella massiliensis	1	0.003%
Pseudomonas syringae pv. actinidiae ICMP 19079	1	0.003%
Lactobacillus johnsonii ATCC 33200	1	0.003%
Flexistipes sinusarabici DSM 4947	1	0.003%
Paenibacillus mucilaginosus 3016	1	0.003%

Species	#BLAST Top-Hits	Percentage
Pseudomonas syringae pv. actinidiae ICMP 19073	1	0.003%
Pseudomonas syringae pv. actinidiae ICMP 19072	1	0.003%
Pseudomonas syringae pv. actinidiae ICMP 19071	1	0.003%
Pseudomonas syringae pv. actinidiae ICMP 19070	1	0.003%
Mycobacterium elephantis	1	0.003%
Enterobacteriaceae bacterium B14	1	0.003%
Pseudomonas syringae pv. syringae	1	0.003%
Escherichia coli RN587/1	1	0.003%
Nitrospirillum amazonense	1	0.003%
Anoplopoma fimbria	1	0.003%
Pseudomonas syringae pv. actinidiae ICMP 19068	1	0.003%
uncultured marine group II/III euryarchaeote KM3_83_G03	1	0.003%
Paenibacillus sp. J14	1	0.003%
Pediococcus claussenii ATCC BAA-344	1	0.003%
Peptoclostridium difficile F501	1	0.003%
Knoellia aerolata	1	0.003%
Geoalkalibacter subterraneus	1	0.003%
Streptomyces galbus	1	0.003%
candidate division TM6 bacterium GW2011_GWF2_36_131	1	0.003%
Cellulosimicrobium funkei	1	0.003%
Leishmania major	1	0.003%
Chryseobacterium sp. OV259	1	0.003%
Streptomyces lactacystinaeus	1	0.003%
Pseudomonas syringae pv. actinidiae ICMP 19097	1	0.003%
Streptomyces violaceusniger Tu 4113	1	0.003%
Saccharomonospora halophila	1	0.003%
Aureispira sp. CCB-QB1	1	0.003%
Actinobacillus minor	1	0.003%
Streptomyces sp. NRRL F-2890	1	0.003%
Clostridium hydrogeniformans	1	0.003%
Sciscionella sp. SE31	1	0.003%
Prevotella sp. CAG:5226	1	0.003%
Porphyrobacter sp. AAP82	1	0.003%
Rhodococcus wratislaviensis IFP 2016	1	0.003%
Rhodococcus triatomae	1	0.003%
Arcanobacterium sp. S3PF19	1	0.003%
Cylindrospermum stagnale PCC 7417	1	0.003%
Verrucomicrobia bacterium L21-Fru-AB	1	0.003%
Vibrio ichthyoenteri ATCC 700023	1	0.003%
Pseudomonas syringae pv. actinidiae ICMP 9855	1	0.003%
Staphylococcus vitulinus	1	0.003%
Clostridium sp. CAG:590	1	0.003%
Brugia malayi	1	0.003%
Abies koreana	1	0.003%
Pseudomonas putida KT2440	1	0.003%
Natronorubrum bangense	1	0.003%
Plasmodium falciparum MaliPS096_E11	1	0.003%
Bacteroides helcogenes P 36-108	1	0.003%
Actinopolymorpha alba	1	0.003%
Janthinobacterium sp. CG23_2	1	0.003%
Rhodococcus pyridinivorans	1	0.003%
Nostoc punctiforme PCC 73102	1	0.003%
Saccharomonospora marina XMU15	1	0.003%

Species	#BLAST Top-Hits	Percentage
Plasmodium falciparum HB3	1	0.003%
Paenibacillus ehimensis	1	0.003%
Pseudomonas protegens	1	0.003%
Thermoactinomyces vulgaris	1	0.003%
Escherichia coli 3003	1	0.003%
Hippotragus niger	1	0.003%
Pleurocapsa minor	1	0.003%
Mycobacterium asiaticum	1	0.003%
Thioclava sp. 13D2W-2	1	0.003%
Sphingomonas sp. ATCC 31555	1	0.003%
Rhodococcus equi	1	0.003%
Aquamicrobium defluvii	1	0.003%
Arthrobacter sp. SPG23	1	0.003%
candidate division WWE3 bacterium GW2011_GWC2_44_9	1	0.003%
Thauera terpenica	1	0.003%
Lyngbya aestuarii BL J	1	0.003%
Cryptococcus neoformans var. neoformans B-3501A	1	0.003%
Clostridium pasteurianum NRRL B-598	1	0.003%
Streptomyces aureus	1	0.003%
Pleodorina starrii	1	0.003%
Olsenella uli	1	0.003%
Crocosphaera watsonii WH 0402	1	0.003%
Bacillus sp. ZYK	1	0.003%
Thermanaerovibrio velox DSM 12556	1	0.003%
Pseudomonas mendocina	1	0.003%
Agkistrodon piscivorus piscivorus	1	0.003%
Amycolicicoccus subflavus DQS3-9A1	1	0.003%
Porphyromonas macacae	1	0.003%
Streptomyces sp. NRRL B-3648	1	0.003%
Prevotella nanceiensis	1	0.003%
Encephalitozoon intestinalis ATCC 50506	1	0.003%
Natronorubrum bangense JCM 10635	1	0.003%
Desulfovibrio vulgaris str. Hildenborough	1	0.003%
Brucella ceti str. Cudo	1	0.003%
Streptomyces tsukubaensis NRRL18488	1	0.003%
Pseudomonas coronafaciens pv. oryzae	1	0.003%
Nitrosospira lacus	1	0.003%
Candidatus Microthrix parvicella	1	0.003%
Flavobacterium frigidarium	1	0.003%
Leptotrichia goodfellowii F0264	1	0.003%
Methanolinea tarda	1	0.003%
Rhodococcus sp. AW25M09	1	0.003%
Tetragenococcus muriaticus	1	0.003%
Pseudomonas mediterranea CFBP 5447	1	0.003%
Granulicella tundricola	1	0.003%
Parascardovia denticolens IPLA 20019	1	0.003%
Pseudomonas sp. RIT-PI-a	1	0.003%
Erythrobacter sp. JL475	1	0.003%
Bacteroides ovatus str. 3725 D9 iii	1	0.003%
Alteromonadaceae bacterium Bs02	1	0.003%
Thiomonas sp. FB-6	1	0.003%
Escherichia coli KTE9	1	0.003%
Bartonella rattaustraliani	1	0.003%

Species	#BLAST Top-Hits	Percentage
Dermacoccus sp. PE3	1	0.003%
Trichophyton rubrum CBS 735.88	1	0.003%
Pleurocapsa sp. PCC 7319	1	0.003%
Ruminococcus albus 7 = DSM 20455	1	0.003%
Saccharothrix syringae	1	0.003%
Butyrivibrio sp. CAG:318	1	0.003%
Thermomonospora curvata DSM 43183	1	0.003%
Agrobacterium larrymoorei	1	0.003%
Crotalus atrox	1	0.003%
Desulfobacula toluolica Tol2	1	0.003%
Desulfovibrio piezophilus	1	0.003%
Pleurocapsa sp. PCC 7327	1	0.003%
Sphingobium japonicum	1	0.003%
Corynebacterium atypicum	1	0.003%
Streptomyces purpureus	1	0.003%
Syntrophobacter fumaroxidans MPOB	1	0.003%
Propionibacterium sp. oral taxon 192	1	0.003%
Streptomyces purpurogeneiscleroticus	1	0.003%
Crotalus durissus terrificus	1	0.003%
Streptomyces sp. TAA040	1	0.003%
Corynebacterium jeikeium	1	0.003%
Thermotoga profunda	1	0.003%
Geobacillus	1	0.003%
Bacillus vietnamensis	1	0.003%
Veillonella sp. AS16	1	0.003%
Capronia semiimmersa	1	0.003%
Methylobacterium nodulans	1	0.003%
Halorubrum coriense	1	0.003%
Nitrosococcus halophilus	1	0.003%
Clostridium sp. DMHC 10	1	0.003%
Prevotella sp. P4-76	1	0.003%
Nocardia sp. NRRL WC-3656	1	0.003%
Paenibacillus alvei DSM 29	1	0.003%
Acidovorax avenae	1	0.003%
Mycoplasma columbinum	1	0.003%
Dehalobacter	1	0.003%
Idiomarina sp. MCCC 1A10513	1	0.003%
Pinus resinosa	1	0.003%
Streptomyces sp. NRRL S-350	1	0.003%
Bordetella parapertussis	1	0.003%
Streptomyces flavochromogenes	1	0.003%
Mucilaginibacter paludis	1	0.003%
Nocardia sp. NRRL S-836	1	0.003%
Lactobacillus coleohominis 101-4-CHN	1	0.003%
Microgenomates (Woesebacteria) bacterium GW2011_GWB1_43_14	1	0.003%
uncultured marine alpha proteobacterium HOT2C01	1	0.003%
Geobacter lovleyi SZ	1	0.003%
Rhizobium sullae	1	0.003%
Thalassospira profundimaris	1	0.003%
Runella slithyformis DSM 19594	1	0.003%
Plasmodium falciparum Palo Alto/Uganda	1	0.003%
Cupriavidus metallidurans	1	0.003%
Streptomyces tsukubaensis	1	0.003%

Species	#BLAST Top-Hits	Percentage
haloalkaliphilic bacterium ACht6-1	1	0.003%
Leucothrix mucor	1	0.003%
Rhodococcus sp. P27	1	0.003%
Clostridium colicanis 209318	1	0.003%
Thiomonas sp. CB2	1	0.003%
Acinetobacter gerneri DSM 14967 = CIP 107464	1	0.003%
marine actinobacterium MedAcidi-G1	1	0.003%
Streptomyces sp. NRRL S-337	1	0.003%
Arthrospira platensis C1	1	0.003%
Pelobacter propionicus DSM 2379	1	0.003%
Sporisorium reilianum SRZ2	1	0.003%
Capra falconeri	1	0.003%
Legionella shakespearei	1	0.003%
Kocuria marina	1	0.003%
Boiga kraepelini	1	0.003%
Geobacillus thermoleovorans B23	1	0.003%
Riemerella anatipestifer Yb2	1	0.003%
Dickeya chrysanthemi	1	0.003%
Sphaerochaeta coccoides DSM 17374	1	0.003%
Sphingobacterium spiritivorum ATCC 33300	1	0.003%
Aquimarina megaterium	1	0.003%
Streptomyces peruviensis	1	0.003%
Pectobacterium carotovorum subsp. brasiliense	1	0.003%
alpha proteobacterium BAL199	1	0.003%
Novosphingobium barchaimii LL02	1	0.003%
Desulfosporosinus acidiphilus	1	0.003%
Mucilaginibacter paludis DSM 18603	1	0.003%
Rhodobacter sp. AKP1	1	0.003%
unidentified phage	1	0.003%
Panonychus citri	1	0.003%
Streptococcus uberis	1	0.003%
Nodosilinea nodulosa	1	0.003%
Salinisphaera hydrothermalis	1	0.003%
Gloeobacter violaceus	1	0.003%
Dyadobacter tibetensis	1	0.003%
Campylobacter concisus UNSW2	1	0.003%
Actinomadura rifamycini	1	0.003%
Teredinibacter turnerae T7901	1	0.003%
Erwinia iniecta	1	0.003%
Ralstonia solanacearum UW551	1	0.003%
Spirillospora albida	1	0.003%
Leptospira interrogans serovar Copenhageni str. M20	1	0.003%
Burkholderia pseudomallei MSHR1043	1	0.003%
Deinococcus sp. YIM 77859	1	0.003%
Synechococcus sp. RS9916	1	0.003%
Waddlia chondrophila 2032/99	1	0.003%
Synechococcus sp. RS9917	1	0.003%
Erwinia piriflorinigrans CFBP 5888	1	0.003%
Escherichia coli O104:H4 str. Ec12-0465	1	0.003%
Bacillus sp. EGD-AK10	1	0.003%
Thiobacillus prosperus	1	0.003%
Xanthomonas oryzae	1	0.003%
Ralstonia solanacearum K60-1	1	0.003%

Species	#BLAST Top-Hits	Percentage
Microgenomates (Amesbacteria) bacterium GW2011_GWC1_46_24	1	0.003%
Komagataella pastoris GS115	1	0.003%
Mesorhizobium sp. LNHC229A00	1	0.003%
Sequoiadendron giganteum	1	0.003%
Brevibacillus massiliensis	1	0.003%
Candidatus Filomicrobium marinum	1	0.003%
Escherichia coli LAU-EC10	1	0.003%
uncultured Thiohalocapsa sp. PB-PSB1	1	0.003%
Lutibaculum baratangense AMV1	1	0.003%
Roseovarius mucosus	1	0.003%
Selenomonas ruminantium	1	0.003%
Lactococcus raffinolactis 4877	1	0.003%
Chlorogloeopsis fritschii	1	0.003%
Gordonia araii NBRC 100433	1	0.003%
Hippea alviniae	1	0.003%
Acinetobacter oleivorans CIP 110421	1	0.003%
Xanthomonas vesicatoria	1	0.003%
Streptomyces sp. NRRL F-5135	1	0.003%
Bacteroides intestinalis DSM 17393	1	0.003%
Veillonella ratti ACS-216-V-Col6b	1	0.003%
Corynebacterium argentoratense	1	0.003%
Catellicoccus marimammalium M35/04/3	1	0.003%
Candidatus Acidianus copahuensis	1	0.003%
Synechococcus sp. PCC 7335	1	0.003%
Entamoeba nuttalli P19	1	0.003%
Alistipes sp. CAG:435	1	0.003%
Corynebacterium freneyi DNF00450	1	0.003%
Vibrio sp. DCR 1-4-2	1	0.003%
Rahnella aquatilis HX2	1	0.003%
Fusarium acuminatum CS5907	1	0.003%
Deinococcus apachensis	1	0.003%
Salmonella enterica subsp. enterica serovar Typhi	1	0.003%
Mycobacterium ulcerans	1	0.003%
Xanthomonas sp. GPE 39	1	0.003%
Desulfovibrio aminophilus	1	0.003%
Escherichia coli UMEA 3163-1	1	0.003%
Enterococcus	1	0.003%
Treponema caldarium	1	0.003%
Chlamydia psittaci 08-2626_L3	1	0.003%
Bacteriovorax sp. BSW11_IV	1	0.003%
Chloracidobacterium thermophilum B	1	0.003%
Butyrivibrio sp. LB2008	1	0.003%
Prochlorococcus sp. scB243_498A3	1	0.003%
Helicobacter bilis	1	0.003%
Pseudogymnoascus pannorum VKM F-4516 (FW-969)	1	0.003%
Parcubacteria (Adlerbacteria) bacterium GW2011_GWA1_54_10	1	0.003%
Corynebacterium xerosis	1	0.003%
Achromobacter sp. LC458	1	0.003%
Leishmania braziliensis MHOM/BR/75/M2904	1	0.003%
Sphingobium baderi LL03	1	0.003%
Neisseria sp. oral taxon 014 str. F0314	1	0.003%
Ehrlichia muris AS145	1	0.003%
Borrelia coriaceae	1	0.003%

Species	#BLAST Top-Hits	Percentage
Aminobacter sp. J41	1	0.003%
Streptacidiphilus jiangxiensis	1	0.003%
Mycobacterium abscessus V06705	1	0.003%
Chloroherpeton thalassium ATCC 35110	1	0.003%
Helicosporidium sp. ATCC 50920	1	0.003%
Escherichia coli P0299917.5	1	0.003%
Citromicrobium sp. JLT1363	1	0.003%
Streptomyces somaliensis	1	0.003%
Entamoeba histolytica HM-1:IMSS-A	1	0.003%
Entamoeba histolytica HM-1:IMSS-B	1	0.003%
Streptomyces sp. CMAA 1322	1	0.003%
Streptomyces showdoensis	1	0.003%
Escherichia coli KTE186	1	0.003%
Roseivivax halodurans	1	0.003%
Paenibacillus sp. JCM 10914	1	0.003%
Cupriavidus sp. SK-4	1	0.003%
Vavraia culicis subsp. floridensis	1	0.003%
Trichophyton rubrum CBS 100081	1	0.003%
Mannheimia succiniciproducens MBEL55E	1	0.003%
Escherichia coli HVH 41 (4-2677849)	1	0.003%
Paraprevotella clara YIT 11840	1	0.003%
Azospirillum sp. B506	1	0.003%
Salinimonas chungwhensis	1	0.003%
Rubrivivax gelatinosus	1	0.003%
Methanolacinia paynteri	1	0.003%
Vibrio sp. DCR 1-4-12	1	0.003%
Butyrivibrio sp. VCB2006	1	0.003%
Rhodopirellula sp. SWK7	1	0.003%
Fervidobacterium islandicum	1	0.003%
Collinsella sp. GD3	1	0.003%
Arthrospira platensis	1	0.003%
Azospirillum sp. B510	1	0.003%
Catenovulum agarivorans	1	0.003%
Acetobacter nitrogenifigens	1	0.003%
Devosia sp. 17-2-E-8	1	0.003%
Dickeya sp. DW 0440	1	0.003%
Ralstonia solanacearum PSI07	1	0.003%
Snodgrassella alvi SCGC AB-598-P14	1	0.003%
Lactobacillus rossiae	1	0.003%
Escherichia coli KTE121	1	0.003%
Maricaulis maris	1	0.003%
Pseudomonas putida KG-4	1	0.003%
Ruminococcus gnavus ATCC 29149	1	0.003%
Selenomonas noxia ATCC 43541	1	0.003%
Lachnospira multipara	1	0.003%
Holospora obtusa	1	0.003%
Marinobacter similis	1	0.003%
Rhodosporidium toruloides	1	0.003%
Desulfotomaculum alkaliphilum	1	0.003%
Propionibacterium granulosum DSM 20700	1	0.003%
Paenibacillus fonticola	1	0.003%
Achromobacter sp. DH1f	1	0.003%
Promicromonosporaceae bacterium W15	1	0.003%

Species	#BLAST Top-Hits	Percentage
Ruminococcus sp. CAG:330	1	0.003%
Pseudomonas entomophila L48	1	0.003%
Escherichia coli KTE147	1	0.003%
Zobellia uliginosa	1	0.003%
Agrobacterium albertimagni AOL15	1	0.003%
Frischella perrara	1	0.003%
Streptomyces griseolus	1	0.003%
Sandarakinorhabdus sp. AAP62	1	0.003%
Bacillus safensis	1	0.003%
Lachnoclostridium phytofermentans ISDg	1	0.003%
Blumeria graminis f. sp. tritici 96224	1	0.003%
Nitrospira defluvii	1	0.003%
Zymomonas mobilis subsp. mobilis ZM4 = ATCC 31821	1	0.003%
Aspergillus ochraceoroseus	1	0.003%
Arthrobacter sp. H20	1	0.003%
Ensifer sp. USDA 6670	1	0.003%
Desulfitobacterium hafniense DCB-2	1	0.003%
Nocardia brevicatena	1	0.003%
Sulfolobus islandicus L.D.8.5	1	0.003%
Phytomonas sp. isolate Hart1	1	0.003%
Eggerthella sp. CAG:368	1	0.003%
Leucobacter sp. CBX130	1	0.003%
Ehrlichia mineirensis	1	0.003%
Coturnix japonica	1	0.003%
Marinilabilia salmonicolor	1	0.003%
Hylobates moloch	1	0.003%
Streptomyces lydicus A02	1	0.003%
Simplicispira psychrophila	1	0.003%
Clostridium] leptum	1	0.003%
Pedobacter heparinus DSM 2366	1	0.003%
Candidatus Methylomirabilis oxyfera	1	0.003%
Streptomyces durhamensis	1	0.003%
Acidobacteriaceae bacterium KBS 83	1	0.003%
Bothrops moojeni	1	0.003%
Parcubacteria bacterium GW2011_GWF2_45_11	1	0.003%
Methylacidiphilum infernorum	1	0.003%
Holospora obtusa F1	1	0.003%
Clostridium] symbiosum WAL-14163	1	0.003%
Entodinium caudatum	1	0.003%
Gluconacetobacter sp. SXCC-1	1	0.003%
Knoellia subterranea KCTC 19937	1	0.003%
Domibacillus indicus	1	0.003%
Paralichthys olivaceus	1	0.003%
Domibacillus enclensis	1	0.003%
Planococcus sp. PAMC 21323	1	0.003%
Jeotgalicoccus marinus	1	0.003%
Pan troglodytes verus	1	0.003%
Pelosinus sp. UFO1	1	0.003%
Akkermansia muciniphila ATCC BAA-835	1	0.003%
Dialister invisus CAG:218	1	0.003%
Pyrobaculum sp. WP30	1	0.003%
Methanothermobacter thermautotrophicus	1	0.003%
Prevotella sp. CAG:255	1	0.003%

Species	#BLAST Top-Hits	Percentage
Hylemonella gracilis	1	0.003%
Ottowia thiooxydans	1	0.003%
Tamias umbrinus	1	0.003%
Parcubacteria (OD1-vii) bacterium GW2011_GWA2_38_13b	1	0.003%
Streptomyces sp. NRRL F-4711	1	0.003%
Geniostoma tinifolium	1	0.003%
Leclercia adecarboxylata	1	0.003%
Burkholderia acidipaludis	1	0.003%
Methylophaga aminisulfidivorans	1	0.003%
Dongia sp. URHE0060	1	0.003%
Desulfosporosinus orientis DSM 765	1	0.003%
Cronobacter universalis NCTC 9529	1	0.003%
Thermus tengchongensis	1	0.003%
Butyrivibrio sp. AE3006	1	0.003%
Butyrivibrio sp. AE3004	1	0.003%
Sphingomonas sp. Ag1	1	0.003%
Stenotrophomonas maltophilia AU12-09	1	0.003%
Actinoplanes subtropicus	1	0.003%
Geobacter sulfurreducens	1	0.003%
Veillonella ratti	1	0.003%
Myxococcus stipitatus DSM 14675	1	0.003%
Segniliparus rotundus DSM 44985	1	0.003%
Actinomyces massiliensis	1	0.003%
Nocardia sp. CNY236	1	0.003%
Citricoccus sp. CH26A	1	0.003%
Corynebacterium striatum	1	0.003%
Bacillus cellulosilyticus DSM 2522	1	0.003%
Leucobacter sp. CBX152	1	0.003%
Roseiflexus sp. RS-1	1	0.003%
Spirochaeta sp. JC202	1	0.003%
Bacillus isronensis B3W22	1	0.003%
Vibrio caribbeanicus ATCC BAA-2122	1	0.003%
Pasteurella dagmatis	1	0.003%
Mycobacterium sp. EPa45	1	0.003%
Shewanella putrefaciens 200	1	0.003%
Dialister micraerophilus	1	0.003%
Bacillus panaciterrae	1	0.003%
Sphingomonas sp. PAMC 26605	1	0.003%
Marine benthic group B archaeon SG8-8-1	1	0.003%
Hyphomicrobium sp. 99	1	0.003%
Granulicatella elegans	1	0.003%
Leptospira kirschneri serovar Valbuzzi str. 200702274	1	0.003%
Candidatus Magnetomorum sp. HK-1	1	0.003%
Pseudomonas sp. DSM 28140	1	0.003%
Aspergillus flavus NRRL3357	1	0.003%
Agrococcus lahaulensis	1	0.003%
Nitrosomonas europaea ATCC 19718	1	0.003%
Nitrosospira sp. NpAV	1	0.003%
Arthrobacter arilaitensis	1	0.003%
Ruminobacter sp. RM87	1	0.003%
Frankia sp. BMG5.12	1	0.003%
Staphylococcus arlettae	1	0.003%
Dielma fastidiosa	1	0.003%

Species	#BLAST Top-Hits	Percentage
Planomicrobium glaciei	1	0.003%
Pyrinomonas methylaliphatogenes	1	0.003%
Listeria fleischmannii FSL S10-1203	1	0.003%
Oribacterium sp. P6A1	1	0.003%
Clostridium] termitidis CT1112	1	0.003%
Enterobacteriaceae bacterium LSJC7	1	0.003%
Citrobacter koseri	1	0.003%
Bacteroides stercorirosoris	1	0.003%
Acinetobacter bohemicus	1	0.003%
Leptospira fainei serovar Hurstbridge str. BUT 6	1	0.003%
Cecembia lonarensis	1	0.003%
Methylotenera mobilis	1	0.003%
Paenibacillus sp. FSL P4-0081	1	0.003%
Pseudomonas aeruginosa VRFPA04	1	0.003%
Bacteroides fragilis str. 3725 D9 ii	1	0.003%
Gordonia otitidis NBRC 100426	1	0.003%
Pantoea ananatis AJ13355	1	0.003%
Burkholderia mimosarum	1	0.003%
Bradyrhizobium sp. Cp5.3	1	0.003%
Tanticharoenia sakaeratensis NBRC 103193	1	0.003%
Mucor ambiguus	1	0.003%
Cellvibrio] gilvus	1	0.003%
Sphingobium japonicum BiD32	1	0.003%
Streptomyces sp. DpondAA-B6	1	0.003%
Choristoneura occidentalis granulovirus	1	0.003%
Streptomyces pyridomyceticus	1	0.003%
Enterococcus faecalis TX1341	1	0.003%
Eimeria mitis	1	0.003%
Rhodopirellula baltica WH47	1	0.003%
Sporolactobacillus vineae	1	0.003%
Streptococcus infantis	1	0.003%
Ewingella americana ATCC 33852	1	0.003%
Trypanosoma rangeli	1	0.003%
Escherichia coli 6-175-07_S1_C3	1	0.003%
Glarea lozoyensis ATCC 20868	1	0.003%
Escherichia coli 6-175-07_S1_C2	1	0.003%
Halococcus salifodinae DSM 8989	1	0.003%
Azospirillum halopraeferens	1	0.003%
Myroides injenensis	1	0.003%
Eikenella corrodens	1	0.003%
Frankia sp. Allo2	1	0.003%
Myxococcus xanthus	1	0.003%
Salisaeta longa	1	0.003%
Marinobacter excellens	1	0.003%
uncultured marine crenarchaeote HF4000_ANIW93H17	1	0.003%
Pseudomonas extremaustralis 14-3 substr. 14-3b	1	0.003%
Leptospirillum ferrooxidans	1	0.003%
Nematocida parisii ERTm3	1	0.003%
Catenuloplanes japonicus	1	0.003%
miscellaneous Crenarchaeota group-15 archaeon DG-45	1	0.003%
Yersinia enterocolitica LC20	1	0.003%
Trichuris trichiura	1	0.003%
Candidatus Arthromitus sp. SFB-mouse-NYU	1	0.003%

Species	#BLAST Top-Hits	Percentage
Halogranum salarium B-1	1	0.003%
Eggerthella lenta 1_1_60AFAA	1	0.003%
Streptococcus equi subsp. zooepidemicus	1	0.003%
Corynebacterium genitalium	1	0.003%
Leptolyngbya sp. JSC-1	1	0.003%
Puma concolor	1	0.003%
Vitreoscilla stercoraria	1	0.003%
Phyllobacterium sp. YR531	1	0.003%
Haloferax alexandrinus	1	0.003%
Bacteroides fragilis str. S6L5	1	0.003%
Clostridium sp. ASF502	1	0.003%
Rhodococcus qingshengii	1	0.003%
Streptococcus porci	1	0.003%
Porphyromonas asaccharolytica PR426713P-I	1	0.003%
Pseudanabaena sp. PCC 6802	1	0.003%
Algoriphagus mannitolivorans	1	0.003%
Nitrospina gracilis	1	0.003%
Knoellia subterranea	1	0.003%
Botrytis cinerea B05.10	1	0.003%
Leptolyngbya sp. PCC 7376	1	0.003%
Escherichia coli p0305293.10	1	0.003%
Thiorhodococcus sp. AK35	1	0.003%
Arthrobacter sp. 11W110_air	1	0.003%
Leptospira meyeri	1	0.003%
Microgenomates (Woesebacteria) bacterium GW2011_GWB1_40_12	1	0.003%
Prevotella sp. HUN102	1	0.003%
Fusobacterium nucleatum	1	0.003%
Methanobrevibacter wolinii	1	0.003%
Kangiella aquimarina	1	0.003%
Clostridium saccharoperbutylacetonicum	1	0.003%
Asaia sp. SF2.1	1	0.003%
Streptomyces floridae	1	0.003%
Citrobacter freundii	1	0.003%
Mesorhizobium sp. ORS3359	1	0.003%
Blastocystis sp. ST4	1	0.003%
Thioflavicoccus mobilis	1	0.003%
Calothrix sp. PCC 7507	1	0.003%
Terriglobus saanensis	1	0.003%
Streptococcus infantis SK1076	1	0.003%
Microgenomates (Woesebacteria) bacterium GW2011_GWB1_38_8	1	0.003%
Thiorhodovibrio sp. 970	1	0.003%
Leptospira interrogans serovar Copenhageni str. LT2050	1	0.003%
Bacteroides uniformis CL03T12C37	1	0.003%
Sphingobium sp. SYK-6	1	0.003%
Acetobacter okinawensis	1	0.003%
Microcystis aeruginosa NIES-843	1	0.003%
Streptomyces megasporus	1	0.003%
Candidatus Accumulibacter sp. SK-02	1	0.003%
Arthrobacter sp. PAO19	1	0.003%
Streptomyces cyaneogriseus subsp. noncyanogenus	1	0.003%
Gluconobacter frateurii NBRC 101659	1	0.003%
Prevotella sp. P6B4	1	0.003%
Lactobacillus malefermentans	1	0.003%

Species	#BLAST Top-Hits	Percentage
Mesorhizobium alhagi CCNWXJ12-2	1	0.003%
Carnobacterium alterfunditum	1	0.003%
Chitinimonas koreensis	1	0.003%
Hyphomonas neptunium ATCC 15444	1	0.003%
Alcanivorax dieselolei B5	1	0.003%
Hyphomonas atlantica	1	0.003%
Methylobacterium sp. WSM2598	1	0.003%
Lactobacillus ruminis	1	0.003%
uncultured marine thaumarchaeote KM3_173_D12	1	0.003%
Escherichia coli KTE199	1	0.003%
Thauera terpenica 58Eu	1	0.003%
Python regius	1	0.003%
Pseudomonas nitroreducens	1	0.003%
Corynebacterium freneyi	1	0.003%
Escherichia coli ARS4.2123	1	0.003%
Plasmodium falciparum	1	0.003%
Streptomyces sp. TP-A0356	1	0.003%
Aliihoeflea sp. 2WW	1	0.003%
Burkholderia pyrrocinia	1	0.003%
Alcanivorax hongdengensis	1	0.003%
Halostagnicola larsenii	1	0.003%
Rhodococcus erythropolis DN1	1	0.003%
Hyphomonas hirschiana VP5	1	0.003%
Pedobacter heparinus	1	0.003%
Selenomonas noxia	1	0.003%
Desulfovibrio vulgaris RCH1	1	0.003%
Shewanella sediminis	1	0.003%
Bifidobacterium boum	1	0.003%
Streptomyces sp. NRRL S-444	1	0.003%
gamma proteobacterium IMCC1989	1	0.003%
Pseudomonas sp. GM17	1	0.003%
Streptomyces seoulensis	1	0.003%
Microgenomates (Amesbacteria) bacterium GW2011_GWB1_47_19	1	0.003%
Aspergillus flavus AF70	1	0.003%
Paenibacillus durus	1	0.003%
Subdoligranulum sp. 4_3_54A2FAA	1	0.003%
Bordetella bronchiseptica M435/02/3	1	0.003%
Candidatus Photodesmus katoptron Akat1	1	0.003%
Naja naja	1	0.003%
Vibrio nigripulchritudo	1	0.003%
Catenulispora acidiphila	1	0.003%
Bacillus timonensis	1	0.003%
Arthrobacter phenanthrenivorans	1	0.003%
alpha proteobacterium symbiont of Oscarella lobularis	1	0.003%
Bacteroides uniformis CL03T00C23	1	0.003%
Picea sitchensis	1	0.003%
Burkholderia pseudomallei 1655	1	0.003%
Amycolatopsis mediterranei	1	0.003%
Massilia sp. BSC265	1	0.003%
Candidatus Accumulibacter sp. SK-11	1	0.003%
Helicobacter canadensis	1	0.003%
Pantoea sp. SM3	1	0.003%
Desulfovibrio hydrothermalis AM13 = DSM 14728	1	0.003%

Species	#BLAST Top-Hits	Percentage
Cycloclasticus	1	0.003%
Thauera linaloolentis 47Lol = DSM 12138	1	0.003%
Leeuwenhoekiella sp. MAR_2009_132	1	0.003%
Anabaena sp. 90	1	0.003%
Thermus scotoductus	1	0.003%
Pantoea sp. BL1	1	0.003%
Methylophilaceae bacterium 11	1	0.003%
Mesorhizobium alhagi	1	0.003%
Candidatus Stoquefichus massiliensis	1	0.003%
Leptospira borgpetersenii str. 200901122	1	0.003%
Cylindrophis ruffus	1	0.003%
Enterococcus faecalis TX1302	1	0.003%
Eremothecium cymbalariae DBVPG#7215	1	0.003%
Phlebiopsis gigantea 11061_1 CR5-6	1	0.003%
Proteobacteria	1	0.003%
Aminobacterium colombiense	1	0.003%
Rubinisphaera brasiliensis	1	0.003%
Pseudomonas sp. 5	1	0.003%
Sulfitobacter geojensis	1	0.003%
Zavarzinella formosa	1	0.003%
Mycobacterium marinum M	1	0.003%
Sinomonas sp. MUSC 117	1	0.003%
Escherichia coli 2-052-05_S4_C1	1	0.003%
Zymomonas mobilis	1	0.003%
Treponema pallidum	1	0.003%
Comamonas testosteroni ATCC 11996	1	0.003%
Mycoplasma alvi	1	0.003%
Actinomyces gerencseriae	1	0.003%
Cystobacter violaceus	1	0.003%
Streptomyces roseosporus NRRL 15998	1	0.003%
Salmonella enterica subsp. enterica serovar Typhimurium str. 14028S	1	0.003%
Cedecea neteri	1	0.003%
Marmoricola aequoreus	1	0.003%
Tyzzerella nexilis	1	0.003%
Paenisporosarcina sp. TG-14	1	0.003%
Pseudomonas aeruginosa MTB-1	1	0.003%
Prevotella melaninogenica ATCC 25845	1	0.003%
Aeromonas salmonicida subsp. salmonicida	1	0.003%
Clostridium sp. CAG:729	1	0.003%
Bacillus sp. SG-1	1	0.003%
Sphingobacterium sp. Ag1	1	0.003%
Mycoplasma sturni	1	0.003%
Xanthomonas oryzae pv. oryzae KACC 10331	1	0.003%
Providencia alcalifaciens F90-2004	1	0.003%
Rhodococcus aetherivorans	1	0.003%
Millerozyma farinosa	1	0.003%
Ruminococcus sp. CAG:254	1	0.003%
Desulfotomaculum gibsoniae	1	0.003%
Photorhabdus temperata subsp. temperata M1021	1	0.003%
Exiguobacterium acetylicum	1	0.003%
Taylorella equigenitalis	1	0.003%
Zunongwangia profunda SM-A87	1	0.003%
Streptomyces puniceus	1	0.003%

Species	#BLAST Top-Hits	Percentage
Desulfatibacillum alkenivorans	1	0.003%
Methylibium sp. CF059	1	0.003%
Roseivivax halodurans JCM 10272	1	0.003%
Streptomyces platensis	1	0.003%
Ensifer sp. BR816	1	0.003%
Escherichia coli KTE236	1	0.003%
Escherichia coli KTE237	1	0.003%
Microbacterium ginsengisoli	1	0.003%
Rahnella sp. WP5	1	0.003%
Pseudoalteromonas sp. BSi20480	1	0.003%
Leisingera daeponensis	1	0.003%
Serratia multitudinisentens	1	0.003%
Ferroplasma sp. Type II	1	0.003%
Yersinia pseudotuberculosis complex	1	0.003%
Helicobasidium mompa endornavirus 1	1	0.003%
Bifidobacterium longum CAG:69	1	0.003%
Paenibacillus sp. IHBB 10380	1	0.003%
Aestuariimicrobium kwangyangense	1	0.003%
Prochlorococcus marinus str. MIT 9301	1	0.003%
Leptospira borgpetersenii serovar Mini str. 200901116	1	0.003%
Micromonospora sp. NRRL B-16802	1	0.003%
Eggerthella sp. CAG:298	1	0.003%
Trichophyton rubrum CBS 202.88	1	0.003%
Halorhabdus tiamatea SARL4B	1	0.003%
Thermomicrobium roseum DSM 5159	1	0.003%
Rudanella lutea	1	0.003%
Comamonadaceae bacterium BICA1-1	1	0.003%
Pseudomonas sp. CF161	1	0.003%
Streptococcus suis	1	0.003%
Flavobacterium akiainvivens	1	0.003%
Clostridium sp. CAG:762	1	0.003%
Leptospira kirschneri str. H1	1	0.003%
Mycobacterium abscessus subsp. bolletii 50594	1	0.003%
Hyphomicrobium	1	0.003%
Candidatus Hamiltonella defensa (Bemisia tabaci)	1	0.003%
Pseudomonas sp. GM79	1	0.003%
Lachnospiraceae bacterium 10-1	1	0.003%
Pseudomonas sp. GM74	1	0.003%
Enterobacter sp. GN02730	1	0.003%
Nocardiopsis valliformis	1	0.003%
Lactobacillus pentosus	1	0.003%
Clostridium autoethanogenum DSM 10061	1	0.003%
Helicobacter sp. MIT 05-5293	1	0.003%
Pantoea ananatis LMG 20103	1	0.003%
Lactobacillus ultunensis	1	0.003%
Haemophilus parasuis ST4-1	1	0.003%
Thiobacillus thioparus	1	0.003%
Streptomyces sp. AS58	1	0.003%
Ralstonia eutropha H16	1	0.003%
Varibaculum cambriense	1	0.003%
bacterium OL-1	1	0.003%
Xanthomonas axonopodis pv. phaseoli	1	0.003%
Blastococcus saxobsidens DD2	1	0.003%

Species	#BLAST Top-Hits	Percentage
Desulfovibrio sp. X2	1	0.003%
Bipolaris zeicola 26-R-13	1	0.003%
Marinimicrobium sp. LS-A18	1	0.003%
Halomonas halocynthiae	1	0.003%
Porphyromonas somerae	1	0.003%
Sorangium cellulosum So0157-2	1	0.003%
actinobacterium LLX17	1	0.003%
Aeromonas phage phiAS7	1	0.003%
Desulfuromonas acetoxidans	1	0.003%
Agrobacterium sp. H13-3	1	0.003%
Bacteriovorax sp. Seq25_V	1	0.003%
Pseudanabaena sp. PCC 7367	1	0.003%
Bacillus nealsonii AAU1	1	0.003%
Bartonella birtlesii	1	0.003%
Acidithrix ferrooxidans	1	0.003%
Peregrinibacteria bacterium GW2011_GWC2_54_8	1	0.003%
Holdemania filiformis DSM 12042	1	0.003%
Glycomyces arizonensis	1	0.003%
Bacillus thuringiensis str. Al Hakam	1	0.003%
Clostridium sp. HMP27	1	0.003%
Variovorax paradoxus	1	0.003%
Virgibacillus pantothenticus	1	0.003%
Prevotella oralis ATCC 33269	1	0.003%
Rhodobacteraceae bacterium PD-2	1	0.003%
Kerstersia gyiorum	1	0.003%
Rhodopseudomonas palustris DX-1	1	0.003%
Candidatus Arthromitus sp. SFB-co	1	0.003%
Planctopirus limnophila DSM 3776	1	0.003%
Pseudoalteromonas luteoviolacea	1	0.003%
Streptomyces hygroscopicus subsp. jinggangensis TL01	1	0.003%
Planomicrobium glaciei CHR43	1	0.003%
Shewanella woodyi	1	0.003%
Amorphus coralli	1	0.003%
Methanomassiliicoccus luminyensis	1	0.003%
Clostridium] cellulosi	1	0.003%
Microvirga lotononidis	1	0.003%
Ehrlichia muris	1	0.003%
Alteromonas macleodii	1	0.003%
Magnetospira sp. QH-2	1	0.003%
Rhodococcus wratislaviensis NBRC 100605	1	0.003%
Coxiella burnetii	1	0.003%
Pyrobaculum aerophilum str. IM2	1	0.003%
Firmicutes bacterium CAG:94	1	0.003%
Marchantia polymorpha	1	0.003%
Nocardiopsis salina	1	0.003%
Parcubacteria bacterium GW2011_GWA2_44_13	1	0.003%
Frankia sp. BCU110501	1	0.003%
Conchiformibius steedae	1	0.003%
Providencia alcalifaciens DSM 30120	1	0.003%
Enterococcus casseliflavus EC30	1	0.003%
Janthinobacterium sp. HH01	1	0.003%
Candida dubliniensis CD36	1	0.003%
Weissella koreensis KCTC 3621	1	0.003%

Species	#BLAST Top-Hits	Percentage
Actinoplanes sp. N902-109	1	0.003%
Paenibacillus sp. FJAT-22460	1	0.003%
Aneurinibacillus migulanus	1	0.003%
Bifidobacterium longum NCC2705	1	0.003%
Eubacterium xylanophilum	1	0.003%
Pseudoxanthomonas suwonensis	1	0.003%
Bacillus okhensis	1	0.003%
Lachnospiraceae bacterium 7_1_58FAA	1	0.003%
Prevotella bergensis DSM 17361	1	0.003%
Yersinia pekkanenii	1	0.003%
Brevibacillus formosus	1	0.003%
Streptomyces prunicolor	1	0.003%
Ralstonia pickettii	1	0.003%
Pseudomonas syringae pv. actinidiae ICMP 9617	1	0.003%
Nocardiopsis sp. SBT366	1	0.003%
Lactobacillus farciminis	1	0.003%
Saccharicrinis fermentans DSM 9555 = JCM 21142	1	0.003%
Xanthomonas arboricola	1	0.003%
Vibrio sp. VPAP30	1	0.003%
Bacillus sp. JCM 19045	1	0.003%
Streptomyces hygroscopicus subsp. jinggangensis 5008	1	0.003%
Lactobacillus delbrueckii subsp. lactis	1	0.003%
Bacillus sp. JCM 19046	1	0.003%
Demetria terragena	1	0.003%
Clostridium celatum	1	0.003%
Teredinibacter sp. 1162T.S.0a.05	1	0.003%
Synbranchus marmoratus	1	0.003%
Paludibacter propionicigenes WB4	1	0.003%
Ralstonia sp. A12	1	0.003%
Nostoc punctiforme	1	0.003%
Enterococcus casseliflavus EC10	1	0.003%
Firmicutes bacterium CAG:65	1	0.003%
Prevotella sp. oral taxon 317	1	0.003%
Bacillus cereus HuA2-1	1	0.003%
Dictyostelium purpureum	1	0.003%
Bacillus cereus HuA2-9	1	0.003%
Dorea longicatena	1	0.003%
Hydrogenophaga	1	0.003%
Curvibacter gracilis	1	0.003%
Prevotella disiens FB035-09AN	1	0.003%
Paenibacillus sp. 1-18	1	0.003%
Plasmodium yoelii	1	0.003%
Acidovorax oryzae	1	0.003%
Xanthomonadaceae	1	0.003%
Thiomonas sp. FB-Cd	1	0.003%
Saccharomyces cerevisiae YJM1479	1	0.003%
Lactobacillus fuchuensis	1	0.003%
Kluyveromyces marxianus	1	0.003%
alpha proteobacterium D323	1	0.003%
Trichosporon asahii var. asahii CBS 8904	1	0.003%
Bacillus sp. SB49	1	0.003%
Leptospira fainei	1	0.003%
Megasphaera sp. BL7	1	0.003%

Species	#BLAST Top-Hits	Percentage
Gardnerella vaginalis 409-05	1	0.003%
Porphyromonas catoniae	1	0.003%
Bacillus methanolicus MGA3	1	0.003%
Skermanella aerolata	1	0.003%
Eubacterium sp. AB3007	1	0.003%
Mesorhizobium metallidurans STM 2683	1	0.003%
Rhodopirellula baltica SH 1	1	0.003%
Ewingella americana	1	0.003%
Sulfolobus acidocaldarius SUSAZ	1	0.003%
Clostridiales	1	0.003%
Acinetobacter pittii ANC 4050	1	0.003%
Cobetia amphilecti	1	0.003%
Azoarcus sp. PA01	1	0.003%
alpha proteobacterium HIMB59	1	0.003%
Microscilla marina ATCC 23134	1	0.003%
Desulfobulbaceae bacterium BRH_c16a	1	0.003%
Epulopiscium sp. 'N.t. morphotype B'	1	0.003%
Streptomyces sp. HmicA12	1	0.003%
Bacteroides faecis CAG:32	1	0.003%
Gordonia malaquae	1	0.003%
Pedobacter sp. V48	1	0.003%
Rahnella	1	0.003%
Pannonibacter phragmitetus	1	0.003%
Bovine leukemia virus (JAPANESE ISOLATE BLV-1)	1	0.003%
Aquincola tertiaricarbonis	1	0.003%
Leptospira sp. B5-022	1	0.003%
Firmicutes bacterium CAG:170	1	0.003%
Meiothermus ruber	1	0.003%
Treponema sp. C6A8	1	0.003%
Bacillus simplex	1	0.003%
Bacteroides uniformis CAG:3	1	0.003%
Beijerinckia indica	1	0.003%
Lactobacillus plantarum	1	0.003%
Niastella koreensis GR20-10	1	0.003%
Neisseria lactamica Y92-1009	1	0.003%
Shewanella	1	0.003%
Cylindrospermum stagnale	1	0.003%
Saccharomonospora sp. CNQ490	1	0.003%
Microcystis aeruginosa PCC 9701	1	0.003%
Elioraea tepidiphila	1	0.003%
Mycobacterium sinense	1	0.003%
Enterococcus casseliflavus	1	0.003%
Thermincola ferriacetica	1	0.003%
Bacteroides sp. CAG:754	1	0.003%
Methanosarcina barkeri 3	1	0.003%
Brevundimonas nasdae	1	0.003%
Methylomonas sp. LW13	1	0.003%
Planktothrix	1	0.003%
Thermococcus litoralis	1	0.003%
Nocardia testacea	1	0.003%
Streptomyces sp. NRRL F-6602	1	0.003%
Rhodanobacter spathiphylli	1	0.003%
Thermodesulfovibrio yellowstonii DSM 11347	1	0.003%

Species	#BLAST Top-Hits	Percentage
Magnetospirillum magneticum	1	0.003%
Oribacterium sp. oral taxon 108 str. F0425	1	0.003%
Streptomyces olindensis	1	0.003%
Brevibacillus agri	1	0.003%
Acinetobacter sp. CIP 56.2	1	0.003%
Azoarcus sp. CIB	1	0.003%
Rhizobium sp. CNPSO 671	1	0.003%
Pseudogymnoascus destructans 20631-21	1	0.003%
Rickettsia endosymbiont of Ixodes pacificus	1	0.003%
Magnetospirillum gryphiswaldense	1	0.003%
Leptospira broomii serovar Hurstbridge str. 5399	1	0.003%
Cryptococcus neoformans var. grubii H99	1	0.003%
Bacillus cereus MSX-D12	1	0.003%
Desulfovibrio sp. 6_1_46AFAA	1	0.003%
Dyadobacter beijingensis	1	0.003%
Streptomyces lydicus	1	0.003%
Paracoccus denitrificans PD1222	1	0.003%
Lysinibacillus sp. LK3	1	0.003%
Chromobacterium vaccinii	1	0.003%
Microcystis aeruginosa PCC 9717	1	0.003%
Chromobacterium violaceum	1	0.003%
Firmicutes bacterium CAG:124	1	0.003%
Sporomusa sp. An4	1	0.003%
Streptomyces sp. WM6378	1	0.003%
Dermabacter hominis	1	0.003%
Phaeodactylibacter xiamenensis	1	0.003%
Prevotella buccae	1	0.003%
Nocardia seriolae	1	0.003%
Corynebacterium doosanense	1	0.003%
Luteimonas sp. J29	1	0.003%
Streptomyces avermitilis	1	0.003%
Cryptococcus gattii CA1014	1	0.003%
Megasphaera genomosp. type_1 str. 28L	1	0.003%
Microbacterium testaceum StLB037	1	0.003%
Pseudomonas sp. PTA1	1	0.003%
Streptomyces sp. WM6372	1	0.003%
Streptomyces lividans 1326	1	0.003%
Chlamydia psittaci C1/97	1	0.003%
Fusobacterium ulcerans 12-1B	1	0.003%
Treponema pallidum subsp. pallidum str. Sea 81-4	1	0.003%
Paenibacillus riograndensis SBR5	1	0.003%
Actinoplanes globisporus	1	0.003%
Aspergillus fumigatus Z5	1	0.003%
Ovibos moschatus	1	0.003%
Xanthomonas	1	0.003%
Marinobacterium jannaschii	1	0.003%
Labrenzia alexandrii DFL-11	1	0.003%
Halorhabdus tiamatea	1	0.003%
Lytechinus variegatus	1	0.003%
Pseudomonas tuomuerensis	1	0.003%
Pseudomonas sp. GM41(2012)	1	0.003%
Streptomyces ghanaensis ATCC 14672	1	0.003%
Desulfovibrio hydrothermalis	1	0.003%

Species	#BLAST Top-Hits	Percentage
Mycobacterium marinum E11	1	0.003%
Syntrophothermus lipocalidus DSM 12680	1	0.003%
Aminobacterium colombiense DSM 12261	1	0.003%
Deinococcus frigens	1	0.003%
Bacteroides faecis	1	0.003%
Salinibacter ruber	1	0.003%
Trachipleistophora hominis	1	0.003%
Actinobaculum sp. oral taxon 183 str. F0552	1	0.003%
Clostridium butyricum 5521	1	0.003%
Pedobacter agri	1	0.003%
Hart Park virus	1	0.003%
Streptomyces sp. XY431	1	0.003%
Streptomyces sp. WM6391	1	0.003%
Desulfovibrio magneticus	1	0.003%
Pseudacidovorax intermedius	1	0.003%
Halosimplex carlsbadense	1	0.003%
Agkistrodon contortrix contortrix	1	0.003%
Hungatella hathewayi DSM 13479	1	0.003%
Fusobacterium varium ATCC 27725	1	0.003%
Caulobacter vibrioides	1	0.003%
Weissella cibaria	1	0.003%
Escherichia coli UMEA 3033-1	1	0.003%
Desulfotomaculum acetoxidans DSM 771	1	0.003%
Desulfatibacillum	1	0.003%
Robinsoniella peoriensis	1	0.003%
Leptospira interrogans str. MMD3731	1	0.003%
Trichodesmium erythraeum IMS101	1	0.003%
Fimbriimonas ginsengisoli	1	0.003%
Streptacidiphilus rugosus	1	0.003%
Castellaniella defragrans 65Phen	1	0.003%
Parcubacteria (Nomurabacteria) bacterium GW2011_GWF2_35_66	1	0.003%
Starkeya novella	1	0.003%
Providencia stuartii	1	0.003%
Salimicrobium jeotgali	1	0.003%
Enterovibrio calviensis	1	0.003%
Kitasatospora setae KM-6054	1	0.003%
Pseudomonas sp. RIT288	1	0.003%
Stenotrophomonas maltophilia RA8	1	0.003%
Komagataeibacter medellinensis	1	0.003%
Methylobacterium sp. GXF4	1	0.003%
Pseudogulbenkiania sp. MAI-1	1	0.003%
Marinomonas profundimaris	1	0.003%
Enterococcus mundtii CRL35	1	0.003%
Desulfatirhabdium butyrativorans	1	0.003%
Pseudoalteromonas piscicida	1	0.003%
Sanguibacter keddieii	1	0.003%
Asticcacaulis biprosthecium	1	0.003%
Sphaerochaeta pleomorpha str. Grapes	1	0.003%
Listeria welshimeri	1	0.003%
Pseudonocardia sp. P2	1	0.003%
Pseudonocardia sp. P1	1	0.003%
Flavobacterium phage 6H	1	0.003%
Streptomyces afghaniensis 772	1	0.003%

Species	#BLAST Top-Hits	Percentage
Marinobacter subterrani	1	0.003%
Leadbetterella byssophila DSM 17132	1	0.003%
Brucella sp. 83/13	1	0.003%
Longispora albida	1	0.003%
Microbacterium sp. CH12i	1	0.003%
Chlamydia sp. 'Rubis'	1	0.003%
alpha proteobacterium Mf 1.05b.01	1	0.003%
Methylophaga aminisulfidivorans MP	1	0.003%
Escherichia vulneris NBRC 102420	1	0.003%
Mesorhizobium australicum WSM2073	1	0.003%
Glaciecola nitratireducens FR1064	1	0.003%
Bacteroides sp. D20	1	0.003%
Lactobacillus rhamnosus	1	0.003%
Desulfovibrio sp. A2	1	0.003%
Ferrovum myxofaciens	1	0.003%
Gracilimonas tropica	1	0.003%
Oceanobacillus massiliensis	1	0.003%
Mannheimia] succiniciproducens	1	0.003%
Sphingomonas changbaiensis	1	0.003%
Stenotrophomonas maltophilia 5BA-I-2	1	0.003%
Paenibacillus sabinae	1	0.003%
Klebsiella pneumoniae RYC492	1	0.003%
Fusobacterium nucleatum subsp. animalis 3_1_33	1	0.003%
Paraprevotella clara	1	0.003%
Leisingera caerulea	1	0.003%
Streptomyces scabiei 87.22	1	0.003%
Helicobacter bilis ATCC 43879	1	0.003%
Bradyrhizobium sp. ORS 375	1	0.003%
Nitrosococcus halophilus Nc 4	1	0.003%
Gillisia sp. CAL575	1	0.003%
Sulfurospirillum arcachonense	1	0.003%
Aeromonas salmonicida subsp. salmonicida 01-B526	1	0.003%
Blochmannia endosymbiont of Polyrhachis (Hedomyrma) turneri	1	0.003%
Metarhizium anisopliae BRIP 53293	1	0.003%
Chryseobacterium sp. P1-3	1	0.003%
Mycobacterium	1	0.003%
Nevskia ramosa	1	0.003%
Lachesis muta	1	0.003%
Rhizobium	1	0.003%
Pantoea sp. At-9b	1	0.003%
Cellulomonas sp. HZM	1	0.003%
Nocardia farcinica	1	0.003%
Fusobacterium sp. CAG:439	1	0.003%
Candidatus Arthromitus sp. SFB-4	1	0.003%
Candidatus Arthromitus sp. SFB-1	1	0.003%
Candidatus Arthromitus sp. SFB-2	1	0.003%
Rhizobium etli bv. mimosae str. IE4771	1	0.003%
Rhizobium undicola	1	0.003%
Roseobacter sp. CCS2	1	0.003%
Flexistipes sinusarabici	1	0.003%
Candidatus Magnetoglobus multicellularis str. Araruama	1	0.003%
Burkholderia sp. A1	1	0.003%
Lentisphaera araneosa	1	0.003%

Species	#BLAST Top-Hits	Percentage
Streptomyces avermitilis MA-4680 = NBRC 14893	1	0.003%
Selenomonas noxia F0398	1	0.003%
Escovopsis weberi	1	0.003%
Nocardia rhamnosiphila	1	0.003%
Bacteroides plebeius CAG:211	1	0.003%
Mesorhizobium australicum	1	0.003%
Desulfurococcus mobilis	1	0.003%
Mycobacterium smegmatis JS623	1	0.003%
Azoarcus sp. BH72	1	0.003%
Rothia dentocariosa ATCC 17931	1	0.003%
alpha proteobacterium LLX12A	1	0.003%
Pseudoxanthomonas suwonensis 11-1	1	0.003%
Verrucomicrobium sp. BvORR106	1	0.003%
Desulfotomaculum acetoxidans	1	0.003%
Chryseobacterium soli	1	0.003%
Altererythrobacter marensis	1	0.003%
Firmicutes bacterium ASF500	1	0.003%
Rhizobium mesoamericanum	1	0.003%
Oscillochloris trichoides DG-6	1	0.003%
Halomonas lutea	1	0.003%
Sinorhizobium medicae	1	0.003%
Clostridium sp. BR72	1	0.003%
Nitrosomonas europaea	1	0.003%
Eikenella corrodens CC92I	1	0.003%
Aspergillus niger ATCC 1015	1	0.003%
Methylomonas denitrificans	1	0.003%
Bacteroides eggerthii	1	0.003%
Mycobacterium avium subsp. hominissuis A5	1	0.003%
Arthrobacter sp. M2012083	1	0.003%
Lactobacillus brevis subsp. gravesensis ATCC 27305	1	0.003%
Rhizobium lupini HPC(L)	1	0.003%
Riemerella anatipestifer RA-YM	1	0.003%
Labrenzia alexandrii	1	0.003%
Mycobacterium gilvum Spyr1	1	0.003%
Fusobacterium ulcerans	1	0.003%
Collinsella aerofaciens ATCC 25986	1	0.003%
Clostridiaceae bacterium MS3	1	0.003%
Paenibacillus sp. UNC451MF	1	0.003%
Rhizobium etli CFN 42	1	0.003%
Domibacillus sp. PAMC 80007	1	0.003%
Klebsiella sp. RIT-PI-d	1	0.003%
Clostridium colicanis	1	0.003%
Bacteroides salyersiae	1	0.003%
Kosakonia radicincitans DSM 16656	1	0.003%
Pichia kudriavzevii	1	0.003%
Methylobacterium sp. 10	1	0.003%
Burkholderia sp. K24	1	0.003%
Pyrobaculum islandicum DSM 4184	1	0.003%
Lactobacillus fabifermentans	1	0.003%
Roseobacter sp. SK209-2-6	1	0.003%
Bifidobacterium gallicum	1	0.003%
Lactobacillus pobuzihii	1	0.003%
Spiroplasma litorale	1	0.003%

Species	#BLAST Top-Hits	Percentage
Arachis hypogaea	1	0.003%
Anaeromyxobacter dehalogenans	1	0.003%
Aeromonas sp. HZM	1	0.003%
Prevotella oralis	1	0.003%
Streptomyces sp. PRh5	1	0.003%
Methylacidiphilum infernorum V4	1	0.003%
Shewanella sediminis HAW-EB3	1	0.003%
Trichophyton rubrum MR850	1	0.003%
Clostridium] litorale	1	0.003%
Zobellia galactanivorans	1	0.003%
Methanosalsum zhilinae DSM 4017	1	0.003%
Pyronema omphalodes CBS 100304	1	0.003%
Gordonia rubripertincta NBRC 101908	1	0.003%
Desulfovibrio desulfuricans ND132	1	0.003%
Clostridium butyricum 60E.3	1	0.003%
Rhodococcus erythropolis	1	0.003%
Bradyrhizobium sp. LTSP857	1	0.003%
Bacillus sp. LF1	1	0.003%
Streptomyces sp. KE1	1	0.003%
Bacteroides xylanisolvens SD CC 2a	1	0.003%
Desulfurispirillum indicum	1	0.003%
Providencia alcalifaciens 205/92	1	0.003%
Leptotrichia buccalis	1	0.003%
Porphyromonas asaccharolytica	1	0.003%
Ptyas dhumnades	1	0.003%
Carboxydothermus ferrireducens	1	0.003%
Porticoccus hydrocarbonoclasticus	1	0.003%
Silicibacter sp. TrichCH4B	1	0.003%
Microtetraspora glauca	1	0.003%
Rhodobacter capsulatus YW2	1	0.003%
Spiroplasma sabaudiense Ar-1343	1	0.003%
Hydrogenophaga sp. T4	1	0.003%
Chthoniobacter flavus Ellin428	1	0.003%
Clostridium beijerinckii	1	0.003%
Paramecium bursaria Chlorella virus NY2A	1	0.003%
Flexibacter roseolus	1	0.003%
Serratia plymuthica PRI-2C	1	0.003%
Rhodobacter sphaeroides WS8N	1	0.003%
Candidatus Entotheonella sp. TSY1	1	0.003%
Rhodococcus rhodochrous	1	0.003%
Nitratireductor aquibiodomus	1	0.003%
Sporosarcina newyorkensis 2681	1	0.003%
Prevotella sp. S7 MS 2	1	0.003%
Burkholderia	1	0.003%
Tremella mesenterica DSM 1558	1	0.003%
Coraliomargarita sp. CAG:312	1	0.003%
Sedimenticola sp. SIP-G1	1	0.003%
Megamonas rupellensis	1	0.003%
Amycolatopsis sp. ATCC 39116	1	0.003%
Nycticebus coucang	1	0.003%
Burkholderia ambifaria IOP40-10	1	0.003%
Saccharomyces cerevisiae YJM1400	1	0.003%
Thiorhodococcus drewsii	1	0.003%

Species	#BLAST Top-Hits	Percentage
Bacteroides xylanisolvens SD CC 1b	1	0.003%
Erwinia pyrifoliae Ep1/96	1	0.003%
Lymphocytic choriomeningitis mammarenavirus	1	0.003%
Serratia sp. M24T3	1	0.003%
Streptococcus oralis	1	0.003%
Bacillus cereus VDM006	1	0.003%
Sulfolobus solfataricus P2	1	0.003%
Photobacterium gaetbulicola Gung47	1	0.003%
Ruegeria pomeroyi	1	0.003%
Clostridium sp. KNHs209	1	0.003%
Streptomyces sp. Tu 6176	1	0.003%
Mobilicoccus sp. SIT2	1	0.003%
Martelella mediterranea	1	0.003%
Microgenomates (Amesbacteria) bacterium GW2011_GWA1_44_24	1	0.003%
Marinobacterium sp. AK27	1	0.003%
Methylobacterium sp. 77	1	0.003%
Streptomyces sp. PBH53	1	0.003%
Massilia sp. 9096	1	0.003%
Bacillus phage PM1	1	0.003%
Rhizobium sp. LC145	1	0.003%
Streptomyces vitaminophilus	1	0.003%
Candidatus Prevotella conceptionensis	1	0.003%
Halobiforma nitratireducens	1	0.003%
Klebsiella pneumoniae MGH 36	1	0.003%
Actinopolyspora erythraea	1	0.003%
Nitrobacter sp. Nb-311A	1	0.003%
Mycobacterium avium subsp. hominissuis 10-4249	1	0.003%
Methylomonas sp. 11b	1	0.003%
Xanthomonas fuscans	1	0.003%
Synechococcus	1	0.003%
Microgenomates (Woesebacteria) bacterium GW2011_GWA1_39_11b	1	0.003%
Leptospira interrogans serovar Copenhageni str. Fiocruz L1-130	1	0.003%
Streptomyces sp. PsTaAH-124	1	0.003%
Butyrivibrio sp. WCD2001	1	0.003%
Thermincola potens	1	0.003%
Reticuloendotheliosis virus	1	0.003%
Streptomyces niveus NCIMB 11891	1	0.003%
Streptomyces sp. CcalMP-8W	1	0.003%
Bacillus aurantiacus	1	0.003%
Clostridium sp. CAG:242	1	0.003%
Klebsiella pneumoniae MGH 64	1	0.003%
Desulfovibrio piezophilus C1TLV30	1	0.003%
Flavobacterium indicum	1	0.003%
Labilithrix luteola	1	0.003%
Mycobacterium sp. UNC280MFTsu5.1	1	0.003%
Agrobacterium albertimagni	1	0.003%
Peregrinibacteria bacterium GW2011_GWA2_44_7	1	0.003%
Mycobacterium llatzerense	1	0.003%
Limnoraphis robusta CS-951	1	0.003%
Lactobacillus ceti	1	0.003%
Bactrocera cucurbitae	1	0.003%
Acetivibrio cellulolyticus	1	0.003%
Orenia marismortui	1	0.003%

Species	#BLAST Top-Hits	Percentage
Rhodopirellula baltica	1	0.003%
Paenibacillus terrae	1	0.003%
Bacillus coahuilensis	1	0.003%
Capnocytophaga ochracea	1	0.003%
Treponema phagedenis	1	0.003%
Clostridiales bacterium VE202-09	1	0.003%
Clostridiales bacterium VE202-08	1	0.003%
Halobacillus dabanensis	1	0.003%
Lymphocystis disease virus 1	1	0.003%
Thermodesulfovibrio	1	0.003%
Enterococcus mundtii	1	0.003%
Musa acuminata AAA Group	1	0.003%
Corynebacterium uterequi	1	0.003%
Thermomonospora curvata	1	0.003%
Peptostreptococcaceae bacterium oral taxon 113 str. W5053	1	0.003%
Clostridiales bacterium VE202-07	1	0.003%
Candidatus Accumulibacter sp. BA-91	1	0.003%
Lactobacillus jensenii	1	0.003%
Anaerotruncus colihominis DSM 17241	1	0.003%
Lactobacillus gigeriorum DSM 23908 = CRBIP 24.85	1	0.003%
delta proteobacterium NaphS2	1	0.003%
Synechococcus elongatus PCC 6301	1	0.003%
Hirschia maritima	1	0.003%
Burkholderia pseudomallei MSHR3709	1	0.003%
Streptomyces sp. WMMB 714	1	0.003%
Bacteroides clarus CAG:160	1	0.003%
gamma proteobacterium HTCC2207	1	0.003%
Allochromatium vinosum DSM 180	1	0.003%
Halobacillus trueperi	1	0.003%
Scytonema tolypothrichoides VB-61278	1	0.003%
Halomonas sp. PBN3	1	0.003%
Polaromonas glacialis	1	0.003%
Acinetobacter ursingii NIPH 706	1	0.003%
Microgenomates (Pacebacteria) bacterium GW2011_GWF1_36_5	1	0.003%
Sugarcane mosaic virus	1	0.003%
Pelagibacterium halotolerans	1	0.003%
Microbispora rosea	1	0.003%
Cryptococcus gattii NT-10	1	0.003%
Rhizobium alamii	1	0.003%
Streptomyces californicus	1	0.003%
Wolbachia endosymbiont of Drosophila simulans	1	0.003%
Corynebacterium genitalium ATCC 33030	1	0.003%
Desulfocapsa sulfexigens DSM 10523	1	0.003%
Pseudovibrio sp. JE062	1	0.003%
Mesorhizobium sp. SOD10	1	0.003%
Desulfobacter postgatei 2ac9	1	0.003%
Paracoccus sp. N5	1	0.003%
Eimeria necatrix	1	0.003%
Thermochromatium tepidum	1	0.003%
Perkinsela sp. CCAP 1560/4	1	0.003%
Marivirga tractuosa	1	0.003%
Kosmotoga pacifica	1	0.003%
Xanthomonas translucens DAR61454	1	0.003%

Species	#BLAST Top-Hits	Percentage
Streptococcus parasanguinis	1	0.003%
Vibrio sp. ECSMB14105	1	0.003%
Rubinisphaera brasiliensis DSM 5305	1	0.003%
Prochlorococcus sp. W2	1	0.003%
Parcubacteria bacterium GW2011_GWC2_44_22	1	0.003%
Arthrobacter sp. MWB30	1	0.003%
Escherichia vulneris	1	0.003%
Terriglobus saanensis SP1PR4	1	0.003%
Bifidobacterium adolescentis	1	0.003%
Escherichia coli PA15	1	0.003%
Clavibacter michiganensis subsp. michiganensis NCPPB 382	1	0.003%
Bacillus acidiproducens	1	0.003%
Prevotella corporis	1	0.003%
acyl-carrier-protein	1	0.003%
Clostridium] papyrosolvens	1	0.003%
Bradyrhizobium sp. STM 3809	1	0.003%
Phytophthora infestans T30-4	1	0.003%
Microgenomates (Levybacteria) bacterium GW2011_GWB1_37_8	1	0.003%
Anabaena variabilis ATCC 29413	1	0.003%
Candidatus Micrarchaeum acidiphilum ARMAN-2	1	0.003%
Pelobacter propionicus	1	0.003%
Haloferax alexandrinus JCM 10717	1	0.003%
Escherichia coli PA31	1	0.003%
bacterium endosymbiont of Mortierella elongata FMR23-6	1	0.003%
Candidatus Brocadia fulgida	1	0.003%
Ideonella sp. 201-F6	1	0.003%
Mycobacterium vulneris	1	0.003%
Sutterella wadsworthensis 2_1_59BFAA	1	0.003%
Cryptobacterium sp. CAG:338	1	0.003%
Acinetobacter tandoii DSM 14970 = CIP 107469	1	0.003%
Streptomyces sp. XY511	1	0.003%
Dyella jiangningensis	1	0.003%
Emticicia oligotrophica	1	0.003%
Riemerella anatipestifer RA-GD	1	0.003%
Arcobacter cibarius	1	0.003%
Enterobacter sp. YD4	1	0.003%
Cupriavidus sp. HMR-1	1	0.003%
Thiomicrospira sp. Milos-T1	1	0.003%
Parcubacteria bacterium GW2011_GWA1_47_9	1	0.003%
Rhizobium phaseoli Ch24-10	1	0.003%
Nocardia brasiliensis NBRC 14402	1	0.003%
Nitrobacter hamburgensis X14	1	0.003%
Toxoplasma gondii VAND	1	0.003%
Corynebacterium pyruviciproducens	1	0.003%
Hafnia alvei ATCC 51873	1	0.003%
Paraglaciecola polaris	1	0.003%
Leptotrichia goodfellowii	1	0.003%
Erwinia pyrifoliae DSM 12163	1	0.003%
Prevotella stercorea DSM 18206	1	0.003%
Curvibacter lanceolatus	1	0.003%
Bacillus sp. OxB-1	1	0.003%
Alteromonas macleodii str. 'Deep ecotype'	1	0.003%
Pandoravirus inopinatum	1	0.003%

Species	#BLAST Top-Hits	Percentage
Saccharomyces cerevisiae AWRI796	1	0.003%
Uliginosibacterium gangwonense	1	0.003%
Comamonas testosteroni TK102	1	0.003%
Natronomonas pharaonis	1	0.003%
Desulfovibrio gigas DSM 1382 = ATCC 19364	1	0.003%
Escherichia coli KTE76	1	0.003%
Aureimonas ureilytica	1	0.003%
Vibrio campbellii	1	0.003%
Butyricimonas virosa	1	0.003%
Streptomyces sp. Tu6071	1	0.003%
Pedobacter oryzae	1	0.003%
Ehrlichia sp. HF	1	0.003%
Polaromonas naphthalenivorans	1	0.003%
Marinovum algicola	1	0.003%
Franconibacter helveticus	1	0.003%
Halostagnicola larsenii XH-48	1	0.003%
Plasmodium falciparum 3D7	1	0.003%
Microgenomates (Gottesmanbacteria) bacterium GW2011_GWA2_47_9	1	0.003%
Legionella geestiana	1	0.003%
Pseudomonas chlororaphis subsp. aurantiaca PB-St2	1	0.003%
Finegoldia magna ATCC 53516	1	0.003%
Serratia fonticola AU-P3(3)	1	0.003%
Deferrisoma camini	1	0.003%
Paenibacillus sp. FSL R7-0273	1	0.003%
Providencia stuartii ATCC 25827	1	0.003%
Diplodia seriata	1	0.003%
Saccharomyces cerevisiae Lalvin QA23	1	0.003%
Acidithiobacillus ferrivorans	1	0.003%
Pseudomonas pseudoalcaligenes CECT 5344	1	0.003%
Alteromonas macleodii str. 'Aegean Sea MED64'	1	0.003%
Small ruminant lentivirus	1	0.003%
Burkholderia sp. RPE64	1	0.003%
Herbaspirillum sp. B65	1	0.003%
Hordeum vulgare subsp. vulgare	1	0.003%
Pseudomonas cremoricolorata	1	0.003%
Mesorhizobium sp. LNHC220B00	1	0.003%
Alistipes finegoldii DSM 17242	1	0.003%
Ichthyophthirius multifiliis	1	0.003%
Serratia symbiotica	1	0.003%
Clostridium] hiranonis DSM 13275	1	0.003%
candidate division CPR2 bacterium GW2011_GWD1_39_7	1	0.003%
Runella slithyformis	1	0.003%
Aeromonas salmonicida subsp. salmonicida A449	1	0.003%
Microgenomates (Pacebacteria) bacterium GW2011_GWF2_38_9	1	0.003%
Desulfovibrio sp. L21-Syr-AB	1	0.003%
Xenorhabdus sp. GDc328	1	0.003%
Mycobacterium smegmatis	1	0.003%
Chryseobacterium sp. OV715	1	0.003%
Desulfurococcus mucosus DSM 2162	1	0.003%
Escherichia coli KTE43	1	0.003%
Raoultella terrigena	1	0.003%
Leptospira santarosai str. HAI821	1	0.003%
Methylosarcina lacus	1	0.003%

Species	#BLAST Top-Hits	Percentage
Methanohalophilus mahii	1	0.003%
Treponema azotonutricium	1	0.003%
Bacteriovorax sp. DB6_IX	1	0.003%
Athalia rosae	1	0.003%
Spiribacter salinus M19-40	1	0.003%
Bacillus sp. FJAT-21352	1	0.003%
Hyphomonas	1	0.003%
Pseudocercospora fijiensis CIRAD86	1	0.003%
Blastocystis hominis	1	0.003%
Stereum hirsutum FP-91666 SS1	1	0.003%
SAR86 cluster bacterium SAR86E	1	0.003%
Pseudomonas sp. LFM046	1	0.003%
Agrococcus pavilionensis	1	0.003%
Kamptonema	1	0.003%
Ramlibacter tataouinensis	1	0.003%
Kutzneria sp. 744	1	0.003%
Desulfovibrio bastinii	1	0.003%
Naumovozyma castellii CBS 4309	1	0.003%
Magnaporthe oryzae 70-15	1	0.003%
Thiorhodospira sibirica	1	0.003%
Actinomyces sp. oral taxon 877 str. F0543	1	0.003%
Sclerotinia borealis F-4157	1	0.003%
Actinomadura oligospora	1	0.003%
Dialister micraerophilus DSM 19965	1	0.003%
Clostridium orbiscindens 1_3_50AFAA	1	0.003%
Bacteroides cellulosilyticus	1	0.003%
Toxoplasma gondii RUB	1	0.003%
Sporomusa ovata	1	0.003%
Peptoclostridium difficile CD160	1	0.003%
Stenotrophomonas maltophilia MF89	1	0.003%
Haemophilus parasuis str. Nagasaki	1	0.003%
Flavobacterium sp. MEB061	1	0.003%
Streptomyces lividans TK24	1	0.003%
Escherichia coli 1-110-08_S4_C1	1	0.003%
Candidatus Carsonella ruddii	1	0.003%
Plasmodium falciparum NF135/5.C10	1	0.003%
Trichophyton rubrum MR1448	1	0.003%
Prevotella enoeca	1	0.003%
Bradyrhizobium sp. ORS 278	1	0.003%
Neisseria sp. oral taxon 014	1	0.003%
Roseburia sp. CAG:197	1	0.003%
Myxococcus stipitatus	1	0.003%
Entamoeba histolytica HM-1:IMSS	1	0.003%
Propionibacterium acnes	1	0.003%
Acinetobacter sp. CIP 102159	1	0.003%
Streptomyces xylophagus	1	0.003%
Yarrowia lipolytica CLIB122	1	0.003%
Babesia microti strain RI	1	0.003%
Hyphomonas chukchiensis	1	0.003%
Thermofilum pendens	1	0.003%
Emticicia oligotrophica DSM 17448	1	0.003%
Agrobacterium tumefaciens LBA4213 (Ach5)	1	0.003%
Streptomyces zinciresistens	1	0.003%

Species	#BLAST Top-Hits	Percentage
Leptospira interrogans serovar Copenhageni str. HAI0188	1	0.003%
Bacillus akibai JCM 9157	1	0.003%
Acetobacter pasteurianus	1	0.003%
Komagataella pastoris CBS 7435	1	0.003%
Sporichthya polymorpha	1	0.003%
Vibrio	1	0.003%
Oscillatoria sp. PCC 10802	1	0.003%
Actinomyces cardiffensis F0333	1	0.003%
Entamoeba invadens IP1	1	0.003%
Fusarium oxysporum Fo47	1	0.003%
Acinetobacter gerneri MTCC 9824	1	0.003%
Escherichia coli 2-005-03_S4_C3	1	0.003%
Escherichia coli 2-005-03_S4_C2	1	0.003%
Pseudomonas mandelii PD30	1	0.003%
Lactobacillus spicheri	1	0.003%
Corynebacterium maris	1	0.003%
Clostridium sp. 12(A)	1	0.003%
Micromonospora sp. ATCC 39149	1	0.003%
Plasmodium falciparum Tanzania (2000708)	1	0.003%
Pseudomonas sp. CMAA1215	1	0.003%
Frateuria aurantia	1	0.003%
Lyngbya confervoides	1	0.003%
Pseudomonas fluorescens WH6	1	0.003%
Tetrasphaera japonica	1	0.003%
Aromatoleum aromaticum	1	0.003%
Cohnella panacarvi	1	0.003%
Mycoplasma conjunctivae	1	0.003%
Megasphaera	1	0.003%
Bacillus circulans	1	0.003%
Sphingobium baderi	1	0.003%
Vibrio shilonii	1	0.003%
Sulfitobacter sp. CB2047	1	0.003%
Thermonema rossianum	1	0.003%
Aspergillus fumigatus A1163	1	0.003%
Serratia plymuthica	1	0.003%
Xenorhabdus doucetiae	1	0.003%
Oribacterium sp. oral taxon 108	1	0.003%
Gordonia malaquae NBRC 108250	1	0.003%
Roseovarius nubinhibens ISM	1	0.003%
bacterial glycogen	1	0.003%
Bifidobacterium actinocoloniiforme DSM 22766	1	0.003%
Clostridium novyi NT	1	0.003%
Tumebacillus flagellatus	1	0.003%
Eubacterium sp. ER2	1	0.003%
Azoarcus toluclasticus	1	0.003%
Microbacterium maritypicum	1	0.003%
Burkholderia mallei	1	0.003%
Burkholderia jiangsuensis	1	0.003%
Erysipelotrichaceae bacterium 3_1_53	1	0.003%
uncultured marine thaumarchaeote KM3_161_D03	1	0.003%
Lactobacillus coleohominis	1	0.003%
Desulfovibrio fructosivorans	1	0.003%
Thermomonas fusca	1	0.003%

Species	#BLAST Top-Hits	Percentage
Cupriavidus basilensis OR16	1	0.003%
Filobasidiella depauperata	1	0.003%
Oscillibacter valericigenes Sjm18-20	1	0.003%
Achromobacter xylosoxidans C54	1	0.003%
Sulfurospirillum barnesii	1	0.003%
Nocardiopsis	1	0.003%
Sulfitobacter phage pCB2047-A	1	0.003%
Selenomonas sputigena ATCC 35185	1	0.003%
Serratia sp. DD3	1	0.003%
Gekko japonicus	1	0.003%
Haemophilus pittmaniae HK 85	1	0.003%
Bacillus akibai	1	0.003%
Methylophilus	1	0.003%
Elizabethkingia sp. BM10	1	0.003%
Hyphomonas adhaerens MHS-3	1	0.003%
Glarea lozoyensis 74030	1	0.003%
Salinisphaera hydrothermalis C41B8	1	0.003%
Paenibacillus sp. HW567	1	0.003%
Rhizobium etli	1	0.003%
Crocinitomix catalasitica	1	0.003%
Butyrivibrio sp. AC2005	1	0.003%
Lactobacillus florum 8D	1	0.003%
Leuconostoc citreum	1	0.003%
candidate division TM6 bacterium GW2011_GWF2_43_87	1	0.003%
Sediminibacterium salmoneum	1	0.003%
Herbaspirillum seropedicae	1	0.003%
Ruegeria pomeroyi DSS-3	1	0.003%
Saccharomyces cerevisiae FostersO	1	0.003%
Lewinella persica	1	0.003%
Desulfosporosinus orientis	1	0.003%
Acetobacter aceti 1023	1	0.003%
Rattus sp.	1	0.003%
Riemerella anatipestifer	1	0.003%
Thermincola potens JR	1	0.003%
Leptospira sp. Fiocruz LV3954	1	0.003%
Coilia nasus	1	0.003%
gamma proteobacterium L18	1	0.003%
Microbacterium maritypicum MF109	1	0.003%
nanoarchaeote Nst1	1	0.003%
candidate division TM6 bacterium GW2011_GWE2_36_25	1	0.003%
Pseudozyma aphidis DSM 70725	1	0.003%
Paenibacillus sp. FSL H7-689	1	0.003%
Shimia sp. SK013	1	0.003%
Deveximentum hanedai	1	0.003%
Streptosporangium roseum DSM 43021	1	0.003%
Pelotomaculum thermopropionicum SI	1	0.003%
Salinibacter ruber DSM 13855	1	0.003%
Bradyrhizobium sp. ORS 285	1	0.003%
Paenibacillus harenae	1	0.003%
Potato virus Y strain O	1	0.003%
Mycobacterium phage HelDan	1	0.003%
Parcubacteria (Uhrbacteria) bacterium GW2011_GWA2_53_10	1	0.003%
Geobacillus sp. CAMR5420	1	0.003%

Species	#BLAST Top-Hits	Percentage
Lonsdalea quercina	1	0.003%
Paludibacter propionicigenes	1	0.003%
Actinoalloteichus cyanogriseus	1	0.003%
Geodermatophilaceae bacterium URHB0062	1	0.003%
Vibrio caribbeanicus	1	0.003%
Rhodopirellula sallentina SM41	1	0.003%
Leptotrichia buccalis C-1013-b	1	0.003%
Vibrio campbellii ATCC BAA-1116	1	0.003%
Lactobacillus kitasatonis	1	0.003%
Gloeocapsa sp. PCC 7428	1	0.003%
Shinella sp. SUS2	1	0.003%
Actinosynnema mirum DSM 43827	1	0.003%
Histoplasma capsulatum G186AR	1	0.003%
Oxalobacteraceae bacterium IMCC9480	1	0.003%
Janthinobacterium lividum	1	0.003%
Cenarchaeum symbiosum A	1	0.003%
Cryptophis nigrescens	1	0.003%
Bacteroides thetaiotaomicron VPI-5482	1	0.003%
Natronolimnobius innermongolicus JCM 12255	1	0.003%
Streptacidiphilus neutrinimicus	1	0.003%
Mesorhizobium sp. WSM3224	1	0.003%
Rahnella aquatilis	1	0.003%
Candidatus Microthrix parvicella RN1	1	0.003%
Microgenomates (Levybacteria) bacterium GW2011_GWA1_39_11	1	0.003%
Kutzneria albida	1	0.003%
Fusarium oxysporum f. sp. cubense race 4	1	0.003%
Bacillus sp. DW5-4	1	0.003%
Fusarium oxysporum f. sp. cubense race 1	1	0.003%
Burkholderia cepacia	1	0.003%
Lactobacillus gigeriorum	1	0.003%
Streptomyces scabrisporus	1	0.003%
Megasphaera genomosp. type_1	1	0.003%
Enterobacter lignolyticus	1	0.003%
Enterobacter lignolyticus SCF1	1	0.003%
Bacillus halodurans	1	0.003%
Microscilla marina	1	0.003%
Photobacterium leiognathi	1	0.003%
Coccidioides posadasii C735 delta SOWgp	1	0.003%
Alistipes finegoldii	1	0.003%
Beijerinckia indica subsp. indica ATCC 9039	1	0.003%
Escherichia coli IS1	1	0.003%
Trichophyton rubrum CBS 288.86	1	0.003%
Clostridium butyricum DKU-01	1	0.003%
Actinomyces sp. oral taxon 171	1	0.003%
Lactobacillus mali	1	0.003%
Sulfuricurvum kujiense DSM 16994	1	0.003%
Bacteroides nordii CL02T12C05	1	0.003%
Mycobacterium phage Che9c	1	0.003%
Comamonas thiooxydans	1	0.003%
Hassallia byssoidea	1	0.003%
Brassica rapa	1	0.003%
Rheinheimera texasensis	1	0.003%
Nitrococcus mobilis	1	0.003%

Species	#BLAST Top-Hits	Percentage
Bifidobacterium sp. 7101	1	0.003%
Aestuariibacter salexigens	1	0.003%
Mesorhizobium sp. LNHC209A00	1	0.003%
Vibrio splendidus	1	0.003%
Halobacillus kuroshimensis	1	0.003%
Ruminococcus sp. CAG:17	1	0.003%
Acetohalobium arabaticum	1	0.003%
Thioalkalivibrio sulfidiphilus HL-EbGr7	1	0.003%
Mycobacterium vanbaalenii	1	0.003%
Dermabacter hominis 1368	1	0.003%
Micromonospora parva	1	0.003%
Bradyrhizobium sp. URHD0069	1	0.003%
Olsenella uli DSM 7084	1	0.003%
Bordetella petrii	1	0.003%
Cycloclasticus sp. P1	1	0.003%
Cyanothece sp. PCC 7425	1	0.003%
Mycobacterium chubuense	1	0.003%
Faecalibacterium sp. CAG:1138	1	0.003%
Arthrobacter sp. 9MFCol3.1	1	0.003%
Candidatus Arthromitus sp. SFB-mouse-NL	1	0.003%
Cordyceps militaris CM01	1	0.003%
Clostridium ljungdahlii DSM 13528	1	0.003%
Tetrasphaera australiensis Ben110	1	0.003%
Sporothrix schenckii ATCC 58251	1	0.003%
Prevotella timonensis	1	0.003%
Clostridium acetobutylicum	1	0.003%
Methylophaga lonarensis	1	0.003%
Bifidobacterium bombi	1	0.003%
Verrucomicrobium sp. 3C	1	0.003%
Hippea jasoniae	1	0.003%
Parcubacteria bacterium GW2011_GWC2_49_9	1	0.003%
Aspergillus oryzae RIB40	1	0.003%
Legionella fallonii	1	0.003%
Roseiflexus castenholzii DSM 13941	1	0.003%
Rubidibacter lacunae	1	0.003%
Paracoccus sp. J55	1	0.003%
Streptomyces sp. CFMR 7	1	0.003%
Legionella longbeachae NSW150	1	0.003%
Finegoldia magna	1	0.003%
Nocardiopsis alkaliphila	1	0.003%
Kyrpidia tusciae DSM 2912	1	0.003%
Acinetobacter tandoii	1	0.003%
Streptococcus	1	0.003%
Prevotella sp. oral taxon 317 str. F0108	1	0.003%
Erythrobacter sp. KA37	1	0.003%
Desulfuromonas acetoxidans DSM 684	1	0.003%
Streptomyces griseoflavus Tu4000	1	0.003%
Streptomyces sp. NRRL WC-3753	1	0.003%
Corynebacterium pyruviciproducens ATCC BAA-1742	1	0.003%
Xanthomonas oryzae pv. oryzicola BLS256	1	0.003%
Rhizobium mongolense	1	0.003%
Shewanella woodyi ATCC 51908	1	0.003%
Streptomyces sp. AA4	1	0.003%

Species	#BLAST Top-Hits	Percentage
Coriobacterium glomerans PW2	1	0.003%
candidate division CPR2 bacterium GW2011_GWC2_39_35	1	0.003%
Pseudomonas syringae pv. actinidiae ICMP 18886	1	0.003%
Pseudomonas taeanensis MS-3	1	0.003%
Asticcacaulis sp. AC460	1	0.003%
Corynebacterium aurimucosum	1	0.003%
Photorhabdus temperata subsp. temperata Meg1	1	0.003%
Pseudomonas syringae pv. actinidiae ICMP 18884	1	0.003%
Geobacillus sp. WCH70	1	0.003%
Parcubacteria bacterium GW2011_GWB1_35_5	1	0.003%
Lactobacillus fermentum	1	0.003%
Lachnospiraceae bacterium 3_1_57FAA_CT1	1	0.003%
Streptomyces sp. NRRL WC-3744	1	0.003%
Blautia sp. KLE 1732	1	0.003%
Devosia soli	1	0.003%
Paracoccus yeei	1	0.003%
Aeromonas popoffii	1	0.003%
Sphaerochaeta pleomorpha	1	0.003%
Thermoproteus tenax	1	0.003%
Bifidobacterium bifidum CAG:234	1	0.003%
Bartonella birtlesii LL-WM9	1	0.003%
Advenella kashmirensis W13003	1	0.003%
Anoxybacillus sp. SK3-4	1	0.003%
Desulfatiglans anilini	1	0.003%
Helicobacter pametensis	1	0.003%
Shinella	1	0.003%
Marinomonas posidonica IVIA-Po-181	1	0.003%
Pirellula staleyi DSM 6068	1	0.003%
Sphingomonas sp. LH128	1	0.003%
Candidatus Arthromitus sp. SFB-mouse-SU	1	0.003%
Schizophyllum commune	1	0.003%
Ceratitis capitata	1	0.003%
Acinetobacter sp. C15	1	0.003%
Leptospira sp. Fiocruz LV4135	1	0.003%
Roseibium sp. TrichSKD4	1	0.003%
Treponema pallidum subsp. pallidum str. Nichols	1	0.003%
Megasphaera sp. UPII 135-E	1	0.003%
Smaragdicoccus niigatensis	1	0.003%
Fusarium pseudograminearum CS3096	1	0.003%
Diaphus metopoclampus	1	0.003%
Microbacterium sp. B24	1	0.003%
Paenibacillus pini JCM 16418	1	0.003%
Acidobacteriaceae bacterium S15	1	0.003%
Plasmodium reichenowi	1	0.003%
Paucisalibacillus globulus	1	0.003%
Alcanivorax hongdengensis A-11-3	1	0.003%
Campylobacter jejuni K1	1	0.003%
Bacillus thermotolerans	1	0.003%
Meiothermus ruber H328	1	0.003%
Staphylococcus simulans ACS-120-V-Sch1	1	0.003%
Dehalobacter sp. DCA	1	0.003%
bacterium UASB270	1	0.003%
Enterobacter cloacae complex	1	0.003%

Species	#BLAST Top-Hits	Percentage
Ferrimonas kyonanensis	1	0.003%
Periplaneta fuliginosa	1	0.003%
Actinoplanes friuliensis	1	0.003%
Pediococcus claussenii	1	0.003%
Methyloversatilis thermotolerans	1	0.003%
Polypterus ornatipinnis	1	0.003%
Yersinia similis	1	0.003%
Streptococcus equi	1	0.003%
Massilia sp. WF1	1	0.003%
Sulfuricella denitrificans skB26	1	0.003%
Pseudochrobactrum sp. AO18b	1	0.003%
Leisingera sp. ANG1	1	0.003%
Leptospira noguchii str. 2001034031	1	0.003%
Sphingomonas jaspsi	1	0.003%
Lewinella cohaerens	1	0.003%
Aeromonas media WS	1	0.003%
Bacillus bogoriensis	1	0.003%
Odoribacter splanchnicus DSM 20712	1	0.003%
Arthrobacter sp. 31Y	1	0.003%
Desulfobacca acetoxidans DSM 11109	1	0.003%
Desulfovibrio desulfuricans	1	0.003%
Campylobacter concisus ATCC 51562	1	0.003%
Asticcacaulis sp. AC402	1	0.003%
Ruegeria mobilis	1	0.003%
Proteus phage PM 93	1	0.003%
Bacillus methanolicus	1	0.003%
Pseudomonas taeanensis	1	0.003%
Idiomarina abyssalis	1	0.003%
Peptococcaceae bacterium BRH_c4b	1	0.003%
Xanthomonas fuscans subsp. aurantifolii str. ICPB 10535	1	0.003%
Vibrio sp. 100512A	1	0.003%
Bacteroides ovatus	1	0.003%
Rozella allomycis CSF55	1	0.003%
Parachlamydiaceae bacterium HS-T3	1	0.003%
Bacteroides thetaiotaomicron	1	0.003%
Agrobacterium rhizogenes	1	0.003%
Azospirillum lipoferum 4B	1	0.003%
Amycolatopsis balhimycina	1	0.003%
Mycobacterium obuense	1	0.003%
Bacteroides fragilis	1	0.003%
Clostridium] saccharogumia	1	0.003%
Massilia timonae CCUG 45783	1	0.003%
Serratia proteamaculans 568	1	0.003%
Halobacillus karajensis	1	0.003%
Neptunomonas japonica	1	0.003%
Pseudomonas mendocina EGD-AQ5	1	0.003%
Peptococcaceae bacterium BICA1-7	1	0.003%
Agkistrodon contortrix laticinctus	1	0.003%
Agrobacterium tumefaciens GW4	1	0.003%
Aeromonas piscicola	1	0.003%
Actinobaculum massiliense ACS-171-V-Col2	1	0.003%
Phytophthora parasitica P1976	1	0.003%
Acetobacter malorum	1	0.003%

Species	#BLAST Top-Hits	Percentage
Photobacterium aquae	1	0.003%
Flavobacterium sp. ABG	1	0.003%
Streptomyces chartreusis	1	0.003%
Bacillus sp. 5B6	1	0.003%
Acinetobacter oleivorans	1	0.003%
Nodularia spumigena	1	0.003%
Actinobacillus capsulatus	1	0.003%
Alloactinosynnema sp. L-07	1	0.003%
Mobiluncus curtisii subsp. curtisii ATCC 35241	1	0.003%
Methanohalophilus mahii DSM 5219	1	0.003%
Photobacterium swingsii	1	0.003%
Isosphaera pallida ATCC 43644	1	0.003%
Acetobacteraceae bacterium AT-5844	1	0.003%
Streptomyces bingchenggensis	1	0.003%
unclassified Clostridiales (miscellaneous)	1	0.003%
Providencia stuartii MRSN 2154	1	0.003%
Bartonella sp. AR 15-3	1	0.003%
Eubacterium sp. CAG:248	1	0.003%
Lyngbya sp. PCC 8106	1	0.003%
Streptomyces sp. SBT349	1	0.003%
Pseudozyma antarctica	1	0.003%
Peregrinibacteria bacterium GW2011_GWF2_39_17	1	0.003%
Escherichia coli 2-005-03_S3_C3	1	0.003%
Eggerthella sp. CAG:1427	1	0.003%
Anaerotruncus sp. G3(2012)	1	0.003%
Escherichia coli 2-005-03_S3_C1	1	0.003%
Microbacterium sp. SUBG005	1	0.003%
Capnocytophaga granulosa ATCC 51502	1	0.003%
Methylobacter luteus	1	0.003%
Borrelia coriaceae Co53	1	0.003%
Bacteroides cellulosilyticus DSM 14838	1	0.003%
Granulicatella elegans ATCC 700633	1	0.003%
Shewanella baltica OS223	1	0.003%
Bacillus sp. FF3	1	0.003%
Bacillus cereus group	1	0.003%
Leptospira broomii	1	0.003%
Bacteroides acidifaciens	1	0.003%
Snodgrassella alvi	1	0.003%
Mogibacterium timidum	1	0.003%
Penicillium rubens Wisconsin 54-1255	1	0.003%
Actinomyces sp. S6-Spd3	1	0.003%
Leptospira borgpetersenii	1	0.003%
Pelistega sp. HM-7	1	0.003%
Pseudoalteromonas atlantica	1	0.003%
Amycolatopsis mediterranei RB	1	0.003%
Marinobacter lipolyticus SM19	1	0.003%
Thermus filiformis	1	0.003%
Peptostreptococcaceae bacterium CM2	1	0.003%
Sphingomonas elodea	1	0.003%
Frankia symbiont of Datisca glomerata	1	0.003%
Sphaerotilus natans	1	0.003%
Haematobacter missouriensis	1	0.003%
Toxoplasma gondii RH	1	0.003%

Species	#BLAST Top-Hits	Percentage
Ruminococcus albus	1	0.003%
Gloydius saxatilis	1	0.003%
Johnsonella ignava ATCC 51276	1	0.003%
Helicobacter canadensis MIT 98-5491	1	0.003%
Dehalococcoides mccartyi CBDB1	1	0.003%
Streptomyces viridochromogenes DSM 40736	1	0.003%
Actinomyces cardiffensis	1	0.003%
Desulfitobacterium sp. PCE1	1	0.003%
Bacillus sp. FJAT-26652	1	0.003%
unclassified Lachnospiraceae	1	0.003%
Peregrinibacteria bacterium GW2011_GWE2_39_6	1	0.003%
Sedimenticola selenatireducens	1	0.003%
Laribacter hongkongensis	1	0.003%
Paenibacillus beijingensis	1	0.003%
Desulfotignum balticum	1	0.003%
Sphingomonas sp. Ant20	1	0.003%
Thermobispora bispora	1	0.003%
Halomonas sp. GFAJ-1	1	0.003%
Corynebacterium doosanense CAU 212 = DSM 45436	1	0.003%
Sulfolobus acidocaldarius	1	0.003%
Plasmodium falciparum FCH/4	1	0.003%
Oceanibaculum indicum P24	1	0.003%
Turnip ringspot virus	1	0.003%
Pseudomonas sp. URMO17WK12:I8	1	0.003%
Clostridiaceae bacterium BRH_c20a	1	0.003%
Pantoea ananatis	1	0.003%
Chitinivibrio alkaliphilus ACht1	1	0.003%
Pseudomonas mediterranea	1	0.003%
Fischerella sp. JSC-11	1	0.003%
Tilletiaria anomala UBC 951	1	0.003%
Leptospira interrogans	1	0.003%
Tetrasphaera japonica T1-X7	1	0.003%
Microgenomates (Woesebacteria) bacterium GW2011_GWC1_42_9	1	0.003%
Streptomyces chattanoogensis	1	0.003%
Streptococcus parauberis	1	0.003%
Mesorhizobium loti MAFF303099	1	0.003%
Herbinix sp. SD1D	1	0.003%
Maribacter sp. Hel_I_7	1	0.003%
Parcubacteria bacterium GW2011_GWA2_43_17	1	0.003%
Trimeresurus gramineus	1	0.003%
Trypanosoma brucei brucei TREU927	1	0.003%
Hafnia alvei	1	0.003%
Balneatrix alpica	1	0.003%
Nostoc sp. PCC 7524	1	0.003%
Cryptomonas paramecium	1	0.003%
Nocardiopsis dassonvillei subsp. dassonvillei DSM 43111	1	0.003%
Kazachstania naganishii CBS 8797	1	0.003%
Clostridium] leptum DSM 753	1	0.003%
Nocardiopsis ganjiahuensis	1	0.003%
Streptomyces roseus	1	0.003%
Zymobacter palmae	1	0.003%
Acidithiobacillus	1	0.003%
Weissella halotolerans	1	0.003%

Species	#BLAST Top-Hits	Percentage
Devosia	1	0.003%
Amycolatopsis methanolica	1	0.003%
Tachyglossus aculeatus	1	0.003%
Streptomyces sp. NRRL B-3229	1	0.003%
Eustrephus latifolius	1	0.003%
Streptomyces zinciresistens K42	1	0.003%
Rhodococcus opacus PD630	1	0.003%
Spiribacter salinus	1	0.003%
Bacteroides finegoldii DSM 17565	1	0.003%
Kosakonia radicincitans	1	0.003%
Novosphingobium acidiphilum	1	0.003%
Pantoea rodasii	1	0.003%
Neorickettsia sp. SF agent	1	0.003%
Capnocytophaga granulosa	1	0.003%
Listeria welshimeri serovar 6b str. SLCC5334	1	0.003%
Clostridium sp. CAG:452	1	0.003%
Tatumella saanichensis	1	0.003%
Brucella	1	0.003%
Rubrivivax benzoatilyticus	1	0.003%
Sediminibacterium sp. OR53	1	0.003%
Methyloversatilis universalis FAM5	1	0.003%
Bacteroides helcogenes	1	0.003%
Nosema ceranae	1	0.003%
Halogranum salarium	1	0.003%
Enhydris plumbea	1	0.003%
Ornithinibacillus californiensis	1	0.003%
Mycobacterium sp. UNCCL9	1	0.003%
Clostridium] sporosphaeroides	1	0.003%
Pseudomonas stutzeri A1501	1	0.003%
Propionibacterium acnes HL201PA1	1	0.003%
Methylophilus sp. Q8	1	0.003%
Ruminococcus gnavus CC55_001C	1	0.003%
Escherichia coli IS25	1	0.003%
Prevotella maculosa	1	0.003%
Halosimplex carlsbadense 2-9-1	1	0.003%
Nocardiopsis sp. CNS639	1	0.003%
Arthrobacter sp. L77	1	0.003%
Methylacidiphilum kamchatkense	1	0.003%
Providencia alcalifaciens RIMD 1656011	1	0.003%
Caldanaerobacter subterraneus subsp. pacificus DSM 12653	1	0.003%
Gloeobacter violaceus PCC 7421	1	0.003%
Acanthocystis turfacea Chlorella virus MN0810.1	1	0.003%
Drechslerella stenobrocha 248	1	0.003%
Porphyromonas catoniae F0037	1	0.003%
Pseudobutyrivibrio ruminis	1	0.003%
Ruminococcus sp. HUN007	1	0.003%
Treponema caldarium DSM 7334	1	0.003%
Streptomyces halstedii	1	0.003%
Nereida ignava	1	0.003%
Caldicellulosiruptor kronotskyensis	1	0.003%
Acinetobacter sp. NIPH 298	1	0.003%
Rhizobium leguminosarum bv. trifolii WSM1325	1	0.003%
Kribbella flavida DSM 17836	1	0.003%

Species	#BLAST Top-Hits	Percentage
Clostridiaceae bacterium GM1	1	0.003%
Halorhodospira halophila SL1	1	0.003%
Leptospira interrogans serovar Icterohaemorrhagiae str. Verdun LP	1	0.003%
Burkholderia rhizoxinica HKI 454	1	0.003%
Rhizobium/Agrobacterium group	1	0.003%
Ensifer sp. TW10	1	0.003%
Ralstonia mannitolilytica	1	0.003%
Meiothermus ruber DSM 1279	1	0.003%
Pseudomonas pelagia	1	0.003%
Catenulispora acidiphila DSM 44928	1	0.003%
Shewanella pealeana ATCC 700345	1	0.003%
Bacteroides gallinarum	1	0.003%
Patulibacter minatonensis	1	0.003%
Anaeroarcus burkinensis	1	0.003%
Staphylothermus hellenicus DSM 12710	1	0.003%
Bradyrhizobium sp. CCGE-LA001	1	0.003%
Halomonas meridiana	1	0.003%
Anguilla japonica	1	0.003%
Novosphingobium malaysiense	1	0.003%
Neochlamydia sp. S13	1	0.003%
Brettanomyces bruxellensis AWRI1499	1	0.003%
Xanthomonas sp. M97	1	0.003%
Sphaerochaeta coccoides	1	0.003%
Candidatus Magnetobacterium casensis	1	0.003%
Candidatus Endolissoclinum faulkneri L5	1	0.003%
Francisella guangzhouensis	1	0.003%
Meiothermus cerbereus	1	0.003%
Paenibacillus alvei	1	0.003%
Cycloclasticus zancles 78-ME	1	0.003%
Burkholderia terrae	1	0.003%
Aromatoleum aromaticum EbN1	1	0.003%
Nocardia nova SH22a	1	0.003%
Pseudomonas syringae pv. actinidiae str. M302091	1	0.003%
Parcubacteria bacterium GW2011_GWB1_41_4	1	0.003%
Prevotella sp. MA2016	1	0.003%
Fodinicurvata fenggangensis	1	0.003%
Fibrisoma limi BUZ 3	1	0.003%
Cycloclasticus sp. PY97M	1	0.003%
Staphylococcus simulans UMC-CNS-990	1	0.003%
Prevotella buccae ATCC 33574	1	0.003%
Psychromonas sp. SP041	1	0.003%
Treponema pedis str. T A4	1	0.003%
Eggerthella lenta DSM 2243	1	0.003%
Cystobacter violaceus Cb vi76	1	0.003%
Agrobacterium tumefaciens 5A	1	0.003%
Dethiobacter alkaliphilus	1	0.003%
Advenella kashmirensis	1	0.003%
Clostridium sp. CAG:299	1	0.003%
Corynebacterium lubricantis	1	0.003%
Rhizobium leucaenae	1	0.003%
Oceanobacillus picturae	1	0.003%
Cryobacterium roopkundense	1	0.003%
Mizuhopecten yessoensis	1	0.003%

Species	#BLAST Top-Hits	Percentage
Streptomyces roseochromogenus	1	0.003%
Thorsellia sp. T2.1	1	0.003%
Pseudonocardia dioxanivorans	1	0.003%
Cupriavidus taiwanensis	1	0.003%
Terasakiella pusilla	1	0.003%
Desulfotomaculum nigrificans	1	0.003%
Propionibacterium sp. KPL1838	1	0.003%
uncultured Flavobacteriia bacterium	1	0.003%
cattle. brain. Peptide. 193 aa	1	0.003%
Bifidobacterium longum subsp. longum 35B	1	0.003%
Protobothrops cornutus	1	0.003%
Sphingomonas sp. SKA58	1	0.003%
Porphyromonas sp. COT-290 OH860	1	0.003%
Methylibium sp. T29	1	0.003%
Corynebacterium halotolerans	1	0.003%
Nocardia pneumoniae	1	0.003%
Bacillus isronensis	1	0.003%
Firmicutes bacterium CAG:272	1	0.003%
Rubrivivax gelatinosus IL144	1	0.003%
Streptomyces sp. NRRL F-5630	1	0.003%
Sphingobium quisquiliarum P25	1	0.003%
Osedax symbiont Rs1	1	0.003%
Gloeophyllum trabeum ATCC 11539	1	0.003%
Streptacidiphilus carbonis	1	0.003%
Curtobacterium sp. S6	1	0.003%
Candidatus Acetothermus autotrophicum	1	0.003%
Peptostreptococcaceae bacterium VA2	1	0.003%
Bifidobacterium actinocoloniiforme	1	0.003%
Lactobacillus mali KCTC 3596 = DSM 20444	1	0.003%
Paenibacillus dendritiformis	1	0.003%
Mesorhizobium sp. LSJC255A00	1	0.003%
Piscirickettsia salmonis	1	0.003%
Methylophilus sp. TWE2	1	0.003%
Dysgonomonas sp. BGC7	1	0.003%
Treponema pallidum subsp. pallidum DAL-1	1	0.003%
Klebsiella phage 0507-KN2-1	1	0.003%
Marivirga tractuosa DSM 4126	1	0.003%
Paracoccus aminophilus JCM 7686	1	0.003%
Ehrlichia ruminantium	1	0.003%
Tistrella mobilis KA081020-065	1	0.003%
Metarhizium majus ARSEF 297	1	0.003%
Legionella norrlandica	1	0.003%
Naja kaouthia	1	0.003%
Rouxiella chamberiensis	1	0.003%
Vibrio ezurae NBRC 102218	1	0.003%
Actinotalea fermentans	1	0.003%
Magnetospirillum gryphiswaldense MSR-1 v2	1	0.003%
Solobacterium moorei	1	0.003%
Sphingobacterium spiritivorum ATCC 33861	1	0.003%
Rhodanobacter	1	0.003%
Burkholderia ginsengisoli	1	0.003%
Propionibacterium sp. KPL1852	1	0.003%
Bacillus aryabhattai	1	0.003%

Species	#BLAST Top-Hits	Percentage
Ruminococcus sp. CAG:624	1	0.003%
Xanthomonas albilineans GPE PC73	1	0.003%
Saccharomyces bayanus	1	0.003%
Acidocella sp. MX-AZ02	1	0.003%
Pseudomonas putida HB3267	1	0.003%
Cardiobacterium valvarum F0432	1	0.003%
Prevotella bergensis	1	0.003%
Firmicutes bacterium CAG:240	1	0.003%
Amycolatopsis mediterranei U32	1	0.003%
Pseudomonas amygdali pv. mori str. 301020	1	0.003%
Wallemia ichthyophaga EXF-994	1	0.003%
Streptomyces griseus group	1	0.003%
Pasteurella dagmatis ATCC 43325	1	0.003%
Stenotrophomonas sp. RIT309	1	0.003%
Oribacterium sinus	1	0.003%
Nevskia soli	1	0.003%
Erwinia piriflorinigrans	1	0.003%
Clostridium] symbiosum	1	0.003%
candidate division TM6 bacterium GW2011_GWF2_28_16	1	0.003%
Streptomyces sp. NRRL WC-3626	1	0.003%
Lactobacillus fabifermentans T30PCM01	1	0.003%
Desulfobacter vibrioformis	1	0.003%
Actinobaculum massiliense	1	0.003%
Burkholderia andropogonis	1	0.003%
Rahnella sp. Y9602	1	0.003%
Sphingomonas sanxanigenens	1	0.003%
Roseiflexus castenholzii	1	0.003%
Streptomyces bingchenggensis BCW-1	1	0.003%
Dasania marina	1	0.003%
Pandoraea	1	0.003%
Lysinibacillus sinduriensis BLB-1 = JCM 15800	1	0.003%
Pseudoalteromonas rubra DSM 6842	1	0.003%
Herbaspirillum frisingense	1	0.003%
Dehalococcoides mccartyi	1	0.003%
Lactobacillus vini	1	0.003%
Exiguobacterium oxidotolerans	1	0.003%
Opitutaceae bacterium TAV5	1	0.003%
Odoribacter splanchnicus	1	0.003%
Sinorhizobium fredii	1	0.003%
Pseudomonas syringae pv. syringae B728a	1	0.003%
Butyrivibrio sp. NC2002	1	0.003%
Cladophialophora carrionii CBS 160.54	1	0.003%
Ruminococcaceae bacterium AB4001	1	0.003%
Pedobacter kyungheensis	1	0.003%
Fluoribacter dumoffii	1	0.003%
Xanthomonas axonopodis	1	0.003%
Synechococcus sp. NKBG042902	1	0.003%
Aquimarina macrocephali	1	0.003%
Photobacterium gaetbulicola	1	0.003%
Pasteurella multocida	1	0.003%
Streptomyces sp. URHA0041	1	0.003%
Brucella sp. NF 2653	1	0.003%
Sphingomonas astaxanthinifaciens	1	0.003%

Species	#BLAST Top-Hits	Percentage
Prunus dulcis x Prunus persica	1	0.003%
Promicromonospora sukumoe	1	0.003%
Moraxella caprae	1	0.003%
Natrinema pellirubrum	1	0.003%
Robinsoniella sp. RHS	1	0.003%
Cellvibrio] gilvus ATCC 13127	1	0.003%
Actinobacillus minor 202	1	0.003%
Arthrobacter alpinus	1	0.003%
Sulfolobus islandicus	1	0.003%
Acinetobacter gerneri	1	0.003%
Treponema azotonutricium ZAS-9	1	0.003%
Toxoplasma gondii GT1	1	0.003%
Tanticharoenia sakaeratensis	1	0.003%
Bacteroides fragilis str. 3996 N(B) 6	1	0.003%
Desulfovibrio fructosivorans JJ	1	0.003%
Micromonospora aurantiaca ATCC 27029	1	0.003%
Halobiforma nitratireducens JCM 10879	1	0.003%
Sciscionella marina	1	0.003%
Anaerostipes hadrus	1	0.003%
candidate division CPR2 bacterium GW2011_GWC1_41_48	1	0.003%
Prevotella intermedia	1	0.003%
Varibaculum cambriense DORA_20	1	0.003%
Streptosporangium amethystogenes	1	0.003%
Propionibacterium acidifaciens F0233	1	0.003%
Eimeria tenella	1	0.003%
Lachnoclostridium phytofermentans	1	0.003%
Clostridium butyricum E4 str. BoNT E BL5262	1	0.003%
Pseudoalteromonas rubra	1	0.003%
Clostridium bartlettii CAG:1329	1	0.003%
uncultured sulfate-reducing bacterium	1	0.003%
Escherichia coli O81:NM str. 02-3012	1	0.003%
Psychromonas arctica	1	0.003%
Bacteroidaceae bacterium MS4	1	0.003%
Paenibacillus popilliae ATCC 14706	1	0.003%
Glaciecola nitratireducens	1	0.003%
Boaedon fuliginosus	1	0.003%
Leeia oryzae	1	0.003%
Bifidobacterium dentium ATCC 27679	1	0.003%
Fusarium oxysporum f. sp. cubense tropical race 4 54006	1	0.003%
Aminobacterium mobile	1	0.003%
Pandoraea sp. SD6-2	1	0.003%
Ponticaulis koreensis	1	0.003%
Acinetobacter pittii	1	0.003%
Abiotrophia defectiva ATCC 49176	1	0.003%
Lactobacillus equi	1	0.003%
Siccibacter turicensis 564	1	0.003%
Saccharomonospora saliphila	1	0.003%
Fictibacillus gelatini	1	0.003%
Jiangella gansuensis	1	0.003%
Pseudomonas syringae pv. actinidiae ICMP 18801	1	0.003%
Rhizobium etli bv. mimosae str. Mim1	1	0.003%
Verrucomicrobiae bacterium DG1235	1	0.003%
Rhodanobacter sp. 115	1	0.003%

Species	#BLAST Top-Hits	Percentage
Yaniella halotolerans	1	0.003%
Aspergillus fumigatus var. RP-2014	1	0.003%
Halanaerobium hydrogeniformans	1	0.003%
Sediminimonas qiaohouensis	1	0.003%
Riemerella anatipestifer RA-CH-2	1	0.003%
Sciscionella	1	0.003%
Natrinema versiforme	1	0.003%
Nocardia nova	1	0.003%
Streptomyces sp. HPH0547	1	0.003%
Candidatus Carsonella ruddii HT isolate Thao2000	1	0.003%
Peptoclostridium difficile	1	0.003%
Saccharomonospora marina	1	0.003%
Burkholderia phenoliruptrix	1	0.003%
Mycobacterium chubuense NBB4	1	0.003%
Clostridium nexile CAG:348	1	0.003%
Gordonia rubripertincta	1	0.003%
Pseudoalteromonas sp. SCSIO_11900	1	0.003%
Bacillus chagannorensis	1	0.003%
Aliivibrio logei	1	0.003%
Alcanivorax sp. PN-3	1	0.003%
Monascus pilosus	1	0.003%
Brevibacillus agri BAB-2500	1	0.003%
Pinus strobus	1	0.003%
ATP	1	0.003%
Escherichia coli O104:H4 str. Ec11-9941	1	0.003%
Ogataea parapolymorpha DL-1	1	0.003%
Hyphomicrobium sp. MC1	1	0.003%
Pseudogymnoascus pannorum VKM F-3557	1	0.003%
Streptomyces ghanaensis	1	0.003%
Thermococcus kodakarensis	1	0.003%
Corynebacterium maris DSM 45190	1	0.003%
Microgenomates (Roizmanbacteria) bacterium GW2011_GWA2_35_19	1	0.003%
Prevotella disiens	1	0.003%
Gordonia otitidis	1	0.003%
Corynebacterium nuruki	1	0.003%
Sphingobium chlorophenolicum L-1	1	0.003%
Cryptococcus gattii EJB2	1	0.003%
Flavobacterium sp. EM1308	1	0.003%
Synechococcus sp. GFB01	1	0.003%
Marinovum algicola DG 898	1	0.003%
Flammeovirga pacifica	1	0.003%
Bacteroides	1	0.003%
Pseudonocardia dioxanivorans CB1190	1	0.003%
Sinorhizobium fredii USDA 257	1	0.003%
Psychrobacter lutiphocae	1	0.003%
Desulfobacula toluolica	1	0.003%
Dickeya	1	0.003%
Chryseobacterium daeguense	1	0.003%
Bacteroides ovatus CL02T12C04	1	0.003%
Natronomonas pharaonis DSM 2160	1	0.003%
Desulfurococcus mucosus	1	0.003%
Bacteroides pyogenes JCM 10003	1	0.003%
Rhodococcus fascians	1	0.003%

Species	#BLAST Top-Hits	Percentage
Enterococcus avium ATCC 14025	1	0.003%
Ochrobactrum	1	0.003%
Thermoproteus tenax Kra 1	1	0.003%
Asticcacaulis sp. YBE204	1	0.003%
Afipia felis	1	0.003%
Mycobacterium chelonae	1	0.003%
Dysgonomonas gadei ATCC BAA-286	1	0.003%
Flavobacterium sp. Fl	1	0.003%
Mangrovibacter sp. MFB070	1	0.003%
Microgenomates (Collierbacteria) bacterium GW2011_GWA1_44_12	1	0.003%
Listeria fleischmannii subsp. coloradonensis	1	0.003%
Gloeocapsa sp. PCC 73106	1	0.003%
Rubellimicrobium mesophilum	1	0.003%
Lactobacillus ruminis ATCC 27782	1	0.003%
Bifidobacterium bombi DSM 19703	1	0.003%
Aquimarina agarilytica	1	0.003%
Plasmodium vinckei petteri	1	0.003%
Pseudomonas agarici	1	0.003%
Afipia	1	0.003%
Clostridium sp. CAG:306	1	0.003%
Piscirickettsia salmonis LF-89 = ATCC VR-1361	1	0.003%
Petrotoga mobilis SJ95	1	0.003%
Fusarium oxysporum f. sp. pisi HDV247	1	0.003%
Geobacillus sp. JF8	1	0.003%
Duganella zoogloeoides	1	0.003%
Clostridium novyi	1	0.003%
Syntrophobacter fumaroxidans	1	0.003%
Bacillus sp. FJAT-21945	1	0.003%
Cecembia lonarensis LW9	1	0.003%
Janthinobacterium sp. CG3	1	0.003%
Paenibacillus pini	1	0.003%
Methanocella arvoryzae	1	0.003%
Oribacterium sp. FC2011	1	0.003%
Zunongwangia profunda	1	0.003%
Commensalibacter sp. MX01	1	0.003%
Thalassobacter sp. 16PALIMAR09	1	0.003%
Thiomonas intermedia K12	1	0.003%
Dethiobacter alkaliphilus AHT 1	1	0.003%
Actinomycetospora chiangmaiensis	1	0.003%
Streptomyces niger	1	0.003%
Legionella longbeachae	1	0.003%
Drosophila virilis	1	0.003%
Paenibacillus sp. JDR-2	1	0.003%
Bacteroides sp. CAG:661	1	0.003%
Alcanivorax	1	0.003%
Salinibacterium sp. PAMC 21357	1	0.003%
Streptomyces griseoaurantiacus	1	0.003%
Pantoea rwandensis	1	0.003%
Bordetella bronchiseptica OSU054	1	0.003%
Xenophilus azovorans	1	0.003%
Geomicrobium sp. JCM 19038	1	0.003%
Sphingomonas sp. MM-1	1	0.003%
Burkholderia ubonensis	1	0.003%

Species	#BLAST Top-Hits	Percentage
Ensifer sp. WSM1721	1	0.003%
Desulfosporosinus sp. OT	1	0.003%
Pseudomonas fluorescens SBW25	1	0.003%
Phyllostachys edulis	1	0.003%
Myxococcus fulvus HW-1	1	0.003%
Thiocystis violascens	1	0.003%
Staphylothermus hellenicus	1	0.003%
Marinomonas ushuaiensis	1	0.003%
Ambystoma tigrinum	1	0.003%
Proteus vulgaris	1	0.003%
Mycobacterium avium 10-5581	1	0.003%
Bacteroides uniformis	1	0.003%
Clostridium sp. CAG:349	1	0.003%
Desulfatibacillum aliphaticivorans	1	0.003%
Corynebacterium caspium	1	0.003%
Frankia sp. Iso899	1	0.003%
Allokutzneria albata	1	0.003%
Paenibacillus sp. FSL R7-277	1	0.003%
Petrotoga mobilis	1	0.003%
Candidatus Sulcia muelleri CARI	1	0.003%
Eggerthella lenta	1	0.003%
Streptomyces sviceus ATCC 29083	1	0.003%
Synechococcus sp. WH 8103	1	0.003%
Clostridium sp. CAG:343	1	0.003%
Acetobacter syzygii	1	0.003%
Roseovarius nubinhibens	1	0.003%
Candidatus Paracaedibacter symbiosus	1	0.003%
Vibrio breoganii	1	0.003%
Gregarina niphandrodes	1	0.003%
Prochlorococcus sp. scB245a_518O7	1	0.003%
Caldisericum exile AZM16c01	1	0.003%
Prochlorothrix hollandica	1	0.003%
Gammaproteobacteria	1	0.003%
Pseudomonas sp. LAMO17WK12:I2	1	0.003%
Tetrasphaera australiensis	1	0.003%
Gymnocephalus cernua	1	0.003%
Gordonia araii	1	0.003%
Kuraishia capsulata CBS 1993	1	0.003%
Thalassospira lucentensis	1	0.003%
Lachnospiraceae bacterium 2_1_58FAA	1	0.003%
Natrinema versiforme JCM 10478	1	0.003%
Fodinicurvata sediminis	1	0.003%
Kitasatospora sp. MY 5-36	1	0.003%
Streptococcus parasanguinis F0449	1	0.003%
Rheinheimera sp. KL1	1	0.003%
Roseobacter litoralis	1	0.003%
Pseudozyma hubeiensis SY62	1	0.003%
Mitsuokella jalaludinii	1	0.003%
Paenibacillus sp. FSL R7-269	1	0.003%
halophilic archaeon J07HX5	1	0.003%
Phytophthora parasitica CJ01A1	1	0.003%
Acetohalobium arabaticum DSM 5501	1	0.003%
Mesorhizobium sp. LSJC264A00	1	0.003%

Species	#BLAST Top-Hits	Percentage
Bradyrhizobium sp. WSM1253	1	0.003%
Arthrobacter arilaitensis Re117	1	0.003%
Thermofilum pendens Hrk 5	1	0.003%
Leptospirillum sp. Group II '5-way CG'	1	0.003%
Gluconobacter frateurii M-2	1	0.003%
Klebsiella pneumoniae	1	0.003%
TOTAL	36023	100.000%
