# Supplementary material for: In-depth transcriptome reveals the potential biotechnological application of Bothrops jararaca venom gland
Source: J Venom Anim Toxins Incl Trop Dis. 2020 Oct 21;26:e20190058. doi: 10.1590/1678-9199-JVATITD-2019-0058 (PMC7579844; doi:10.1590/1678-9199-JVATITD-2019-0058)
Supplement: Additional file 12. [file 1678-9199-jvatitd-26-e20190058-s12.pdf]

J Venom Anim Toxins incl Trop Dis

https://doi.org/10.1590/1678-9199-JVATITD-2019-0058

Supplementary Material to "In-depth transcriptome reveals the potential biotechnological application of *Bothrops jararaca* venom gland"

Additional file 12. Animal toxins encoded in the *Bothrops jararaca* transcriptome identified in the Animal Toxin Annotation Project.

Sequence Name	Sequence Description	Blast Top Hit Accession	Blast Top Hit Score
**DN34028_c0_g1**	VCO3_CROAD Venom factor OS=Crotalus adamanteus OX=8729 PE=2 SV=1	J3S836	3343,9
**DN32044_c0_g1**	PDE1_CROAD Venom phosphodiesterase 1 OS=Crotalus adamanteus OX=8729 PE=1 SV=2	J3SEZ3	1686
**DN34028_c0_g1**	VCO3_CROAD Venom factor OS=Crotalus adamanteus OX=8729 PE=2 SV=1	J3S836	1559,66
**DN29580_c0_g1**	V5NTD_CROAD Snake venom 5'-nucleotidase OS=Crotalus adamanteus OX=8729 PE=1 SV=2	F8S0Z7	1125,54
**DN31772_c0_g1**	PLB_CROAD Phospholipase B OS=Crotalus adamanteus OX=8729 PE=1 SV=1	F8S101	1117,45
**DN34088_c1_g1**	VM3BP_BOTJA Zinc metalloproteinase-disintegrin-like bothropasin OS=Bothrops jararaca OX=8724 PE=1 SV=2	O93523	1109,36
**DN34049_c16_g1**	VM36A_BOTIN Zinc metalloproteinase-disintegrin-like BITM06A OS=Bothrops insularis OX=8723 PE=2 SV=1	Q8QG88	1085,09
**DN31322_c0_g1**	OXLA_BOTPAL-amino-acid oxidase (Fragment) OS=Bothrops pauloensis OX=1042543 PE=1 SV=1	B5AR80	1038,1
**DN32234_c0_g1**	OXLA_BOTPA L-amino-acid oxidase (Fragment) OS=Bothrops pauloensis OX=1042543 PE=1 SV=1	B5AR80	1035,4
**DN34028_c0_g1**	VCO3_CROAD Venom factor OS=Crotalus adamanteus OX=8729 PE=2 SV=1	J3S836	1025,77
**DN34088_c1_g1**	VM2J2_BOTJA Zinc metalloproteinase/disintegrin OS=Bothrops jararaca OX=8724 PE=1 SV=1	Q98SP2	925,235
**DN29659_c0_g1**	HYAL_CROAD Hyaluronidase OS=Crotalus adamanteus OX=8729 PE=1 SV=1	J3S820	902,894
**DN31047_c0_g1**	LIPE_CROAD Putative endothelial lipase OS=Crotalus adamanteus OX=8729 PE=2 SV=1	J3RZ81	869,381
**DN34088_c1_g1**	VM2J2_BOTJA Zinc metalloproteinase/disintegrin OS=Bothrops jararaca OX=8724 PE=1 SV=1	Q98SP2	851,662
**DN34049_c16_g1**	VM3JA_BOTJA Zinc metalloproteinase-disintegrin-like jararhagin (Fragment) OS=Bothrops jararaca OX=8724 PE=1 SV=1	P30431	825,469
**DN11926_c0_g1**	LICH_CROAD Putative lysosomal acid lipase/cholesteryl ester hydrolase OS=Crotalus adamanteus OX=8729 PE=2 SV=1	J3SDX8	797,734
**DN32861_c0_g1**	QPCT_BOTJA Glutaminyl-peptide cyclotransferase OS=Bothrops jararaca OX=8724 GN=QPCT PE=2 SV=1	Q9YIB5	764,607
**DN34049_c16_g1**	VM3JA_BOTJA Zinc metalloproteinase-disintegrin-like jararhagin (Fragment) OS=Bothrops jararaca OX=8724 PE=1 SV=1	P30431	749,199
**DN34028_c0_g1**	VCO3_CROAD Venom factor OS=Crotalus adamanteus OX=8729 PE=2 SV=1	J3S836	739,184
**DN34088_c1_g1**	VM2IA_BOTIN Zinc metalloproteinase/disintegrin OS=Bothrops insularis OX=8723 PE=1 SV=1	Q5XUW8	657,907
**DN28767_c0_g1**	RCN2V_CROAD Reticulocalbin-2 OS=Crotalus adamanteus OX=8729 PE=1 SV=1	J3S9D9	590,112
**DN33554_c8_g1**	VM3H3_BOTJA Zinc metalloproteinase-disintegrin-like HF3 OS=Bothrops jararaca OX=8724 PE=1 SV=3	Q98UF9	549,28
**DN33554_c8_g1**	VM3H3_BOTJA Zinc metalloproteinase-disintegrin-like HF3 OS=Bothrops jararaca OX=8724 PE=1 SV=3	Q98UF9	535,413
**DN33554_c8_g1**	VM38_CROAD Zinc metalloproteinase-disintegrin-like 8 OS=Crotalus adamanteus OX=8729 PE=1 SV=1	J3SDW8	514,227
**DN33554_c8_g1**	VM38_CROAD Zinc metalloproteinase-disintegrin-like 8 OS=Crotalus adamanteus OX=8729 PE=1 SV=1	J3SDW8	508,064
**DN54672_c0_g1**	NGFV_BOTJR Venom nerve growth factor OS=Bothrops jararacussu OX=8726 GN=NGF PE=2 SV=1	Q90W38	502,286
**DN33554_c8_g1**	VM31_BOTAT Zinc metalloproteinase-disintegrin-like batroxstatin-1 OS=Bothrops atrox OX=8725 PE=2 SV=1	C5H5D2	501,901
**DN34088_c1_g2**	VM3JA_BOTJA Zinc metalloproteinase-disintegrin-like jararhagin (Fragment) OS=Bothrops jararaca OX=8724 PE=1 SV=1	P30431	484,952
**DN32463_c0_g1**	VCO3_CROAD Venom factor OS=Crotalus adamanteus OX=8729 PE=2 SV=1	J3S836	482,256
**DN33554_c8_g1**	VM31_BOTAT Zinc metalloproteinase-disintegrin-like batroxstatin-1 OS=Bothrops atrox OX=8725 PE=2 SV=1	C5H5D2	482,256
**DN33554_c8_g1**	VM3H3_BOTJA Zinc metalloproteinase-disintegrin-like HF3 OS=Bothrops jararaca OX=8724 PE=1 SV=3	Q98UF9	466,463
**DN33554_c8_g1**	VM31_BOTAT Zinc metalloproteinase-disintegrin-like batroxstatin-1 OS=Bothrops atrox OX=8725 PE=2 SV=1	C5H5D2	464,537
**DN8104_c0_g1**	VCP_APIME Venom serine carboxypeptidase OS=Apis mellifera OX=7460 PE=2 SV=1	C9WMM5	460,685
**DN34088_c1_g2**	VM3JA_BOTJA Zinc metalloproteinase-disintegrin-like jararhagin (Fragment) OS=Bothrops jararaca OX=8724 PE=1 SV=1	P30431	459,144
**DN33554_c8_g1**	VM3H3_BOTJAZinc metalloproteinase-disintegrin-like HF3 OS=Bothrops jararaca OX=8724 PE=1 SV=3	Q98UF9	458,373
**DN33554_c8_g1**	VM31_BOTAT Zinc metalloproteinase-disintegrin-like batroxstatin-1 OS=Bothrops atrox OX=8725 PE=2 SV=1	C5H5D2	447,203
**DN19402_c0_g1**	CRVP_AGKPI Cysteine-rich venom protein piscivorin OS=Agkistrodon piscivorus piscivorus OX=8716 PE=1 SV=1	Q7ZTA0	445,277
**DN33061_c11_g1**	VDPP4_APIME Venom dipeptidyl peptidase 4 OS=Apis mellifera OX=7460 PE=1 SV=1	B2D0J4	445,277
**DN21958_c0_g1**	CRVP_AGKPI Cysteine-rich venom protein piscivorin OS=Agkistrodon piscivorus piscivorus OX=8716 PE=1 SV=1	Q7ZTA0	444,121
**DN33312_c0_g1**	FA5V_OXYMI Venom prothrombin activator omicarin-C non-catalytic subunit OS=Oxyuranus microlepidotus OX=111177 PE=2 SV=1	Q58L90	442,195
**DN33312_c0_g1**	FA5V_OXYMI Venom prothrombin activator omicarin-C non-catalytic subunit OS=Oxyuranus microlepidotus OX=111177 PE=2 SV=1	Q58L90	441,81
**DN33023_c0_g1**	VEGFA_PROFL Vascular endothelial growth factor A OS=Protobothrops flavoviridis OX=88087 PE=1 SV=1	P67860	439,113
**DN33554_c8_g1**	VM31_BOTAT Zinc metalloproteinase-disintegrin-like batroxstatin-1 OS=Bothrops atrox OX=8725 PE=2 SV=1	C5H5D2	430,254
**DN31826_c0_g1**	VSP04_TRIST Snake venom serine protease KN4 homolog OS=Trimeresurus stejnegeri OX=39682 PE=2 SV=1	Q71QJ4	429,483
**DN31826_c0_g1**	VSP04_TRIST Snake venom serine protease KN4 homolog OS=Trimeresurus stejnegeri OX=39682 PE=2 SV=1	Q71QJ4	428,328
**DN28629_c0_g1**	NEP_TRILK Neprilysin-1 OS=Trittame loki OX=1295018 PE=1 SV=1	W4VS99	424,476
**DN30499_c0_g1**	VKT_AUSLA Putative Kunitz-type serine protease inhibitor OS=Austrelaps labialis OX=471292 PE=2 SV=1	B2BS84	408,297
**DN28629_c0_g1**	NEP_TRILK Neprilysin-1 OS=Trittame loki OX=1295018 PE=1 SV=1	W4VS99	402,134
**DN34088_c1_g2**	VM3JA_BOTJA Zinc metalloproteinase-disintegrin-like jararhagin (Fragment) OS=Bothrops jararaca OX=8724 PE=1 SV=1	P30431	402,134
**DN33023_c0_g1**	VEGFA_AGKPI Vascular endothelial growth factor A OS=Agkistrodon piscivorus piscivorus OX=8716 PE=2 SV=1	C0K3N4	390,193
**DN33554_c8_g1**	VM3H3_BOTJA Zinc metalloproteinase-disintegrin-like HF3 OS=Bothrops jararaca OX=8724 PE=1 SV=3	Q98UF9	388,267
**DN28278_c2_g1**	HYAL1_BITAR Hyaluronidase-1 OS=Bitis arietans OX=8692 PE=2 SV=1	A3QVN9	385,571
**DN31772_c0_g1**	PLB_CROAD Phospholipase B OS=Crotalus adamanteus OX=8729 PE=1 SV=1	F8S101	384,8
**DN36408_c0_g1**	PDE2_CROAD Venom phosphodiesterase 2 OS=Crotalus adamanteus OX=8729 PE=1 SV=1	J3SBP3	384,03
**DN19402_c0_g1**	CRVP_AGKPI Cysteine-rich venom protein piscivorin OS=Agkistrodon piscivorus piscivorus OX=8716 PE=1 SV=1	Q7ZTA0	380,563
**DN34049_c16_g1**	VM32_LACMR Zinc metalloproteinase-disintegrin-like lachestatin-2 OS=Lachesis muta rhombeata OX=60219 PE=2 SV=1	C5H5D6	379,793
**DN33554_c8_g1**	VM3AA_CROAT Zinc metalloproteinase-disintegrin-like atrolysin-A (Fragment) OS=Crotalus atrox OX=8730 PE=1 SV=1	Q92043	379,407
**DN33554_c8_g1**	VM3H3_BOTJA Zinc metalloproteinase-disintegrin-like HF3 OS=Bothrops jararaca OX=8724 PE=1 SV=3	Q98UF9	376,326
**DN24099_c0_g2**	VM3_NAJAT Zinc metalloproteinase-disintegrin-like NaMP OS=Naja atra OX=8656 PE=2 SV=1	A8QL59	375,17
**DN31365_c0_g1**	BNP_BOTIN Bradykinin-potentiating and C-type natriuretic peptides OS=Bothrops insularis OX=8723 PE=1 SV=2	P68515	359,377
**DN27551_c0_g1**	TCTP_CROHD Translationally-controlled tumor protein homolog OS=Crotalus horridus OX=35024 PE=2 SV=1	T1DKS4	352,058
**DN33592_c1_g1**	QPCT_GLOBL Glutaminyl-peptide cyclotransferase OS=Gloydius blomhoffii OX=242054 GN=QPCT PE=2 SV=1	Q90YA8	351,673
**DN32888_c0_g1**	VCO3_CROAD Venom factor OS=Crotalus adamanteus OX=8729 PE=2 SV=1	J3S836	351,288
**DN30958_c0_g1**	NEP_TRILK Neprilysin-1 OS=Trittame loki OX=1295018 PE=1 SV=1	W4VS99	347,821
**DN31826_c0_g1**	VSPA_BOTJA Thrombin-like enzyme bothrombin OS=Bothrops jararaca OX=8724 PE=1 SV=1	P81661	339,347
**DN33554_c8_g1**	VM31_BOTAT Zinc metalloproteinase-disintegrin-like batroxstatin-1 OS=Bothrops atrox OX=8725 PE=2 SV=1	C5H5D2	332,413
**DN33554_c8_g1**	VM31_BOTAT Zinc metalloproteinase-disintegrin-like batroxstatin-1 OS=Bothrops atrox OX=8725 PE=2 SV=1	C5H5D2	331,257

Sequence Name	Sequence Description	Blast Top Hit Accession	Blast Top Hit Score
DN7769_c0_g1	VCO31_AUSSU A.superbus venom factor 1 OS=Austrelaps superbus OX=29156 PE=1 SV=1	Q0ZZJ6	327,791
DN62927_c0_g1	LECG_BOTIN C-type lectin BiL OS=Bothrops insularis OX=8723 PE=1 SV=1	Q6QX33	325,479
DN21889_c0_g1	CALGL_BOTIN Calglandulin OS=Bothrops insularis OX=8723 PE=2 SV=1	Q8AY75	316,62
DN32310_c0_g1	VSP_BOTIN Snake venom serine protease BITS01A OS=Bothrops insularis OX=8723 PE=2 SV=1	Q8QG86	313,538
DN32310_c0_g1	VSP1_BOTJA Platelet-aggregating proteinase PA-BJ (Fragment) OS=Bothrops jararaca OX=8724 PE=1 SV=2	P81824	313,153
DN32310_c0_g1	VSPA_BOTJA Thrombin-like enzyme bothrombin OS=Bothrops jararaca OX=8724 PE=1 SV=1	P81661	313,153
DN33554_c8_g1	VM2V2_CROAT Zinc metalloproteinase-disintegrin VMP-II OS=Crotalus atrox OX=8730 PE=2 SV=1	C9E1R7	310,842
DN60211_c0_g1	NGFV_CRODU Venom nerve growth factor OS=Crotalus durissus terrificus OX=8732 PE=2 SV=1	Q9DEZ9	310,071
DN33554_c8_g1	VM2V2_CROAT Zinc metalloproteinase-disintegrin VMP-II OS=Crotalus atrox OX=8730 PE=2 SV=1	C9E1R7	309,301
DN29729_c1_g1	VSPB_GLOBL Beta-fibrinogenase brevinase OS=Gloydius blomhoffii OX=242054 PE=1 SV=1	Q9PT51	309,301
DN32310_c0_g1	VSPA_BOTJA Thrombin-like enzyme bothrombin OS=Bothrops jararaca OX=8724 PE=1 SV=1	P81661	306,605
DN33554_c8_g1	VM34_CROAD Zinc metalloproteinase-disintegrin-like 4a OS=Crotalus adamanteus OX=8729 PE=1 SV=1	F8S108	302,753
DN39558_c0_g1	PDE2_CROAD Venom phosphodiesterase 2 OS=Crotalus adamanteus OX=8729 PE=1 SV=1	J3SBP3	302,368
DN32310_c0_g1	VSPTL_BOTAL Snake venom serine protease BthaTL OS=Bothrops alternatus OX=64174 PE=2 SV=1	Q6IWF1	301,982
DN23222_c0_g1	TXVE_BOTJA Snake venom vascular endothelial growth factor toxin OS=Bothrops jararaca OX=8724 PE=1 SV=1	Q90X23	301,212
DN32310_c0_g1	VSPTL_BOTAL Snake venom serine protease BthaTL OS=Bothrops alternatus OX=64174 PE=2 SV=1	Q6IWF1	300,442
DN34152_c6_g1	VSP14_BOTJA Snake venom serine protease HS114 OS=Bothrops jararaca OX=8724 PE=1 SV=1	Q5W959	296,975
DN32310_c0_g1	VSPTL_BOTAL Snake venom serine protease BthaTL OS=Bothrops alternatus OX=64174 PE=2 SV=1	Q6IWF1	296,59
DN33554_c8_g1	VM2V2_CROAT Zinc metalloproteinase-disintegrin VMP-II OS=Crotalus atrox OX=8730 PE=2 SV=1	C9E1R7	294,278
DN34152_c6_g1	VSP14_BOTJA Snake venom serine protease HS114 OS=Bothrops jararaca OX=8724 PE=1 SV=1	Q5W959	294,278
DN33113_c2_g1	VM2IA_BOTIN Zinc metalloproteinase/disintegrin OS=Bothrops insularis OX=8723 PE=1 SV=1	Q5XUW8	293,893
DN64527_c0_g1	ACES_BUNFA Acetylcholinesterase OS=Bungarus fasciatus OX=8613 GN=ACHE PE=1 SV=2	Q92035	292,352
DN12020_c0_g1	FA5V_PSETE Venom prothrombin activator pseutarin-C non-catalytic subunit OS=Pseudonaja textilis OX=8673 PE=1 SV=1	Q7SZN0	292,352
DN32310_c0_g1	VSP_LACST Snake venom serine protease OS=Lachesis stenophrys OX=88085 PE=2 SV=1	Q072L7	292,352
DN32310_c0_g1	VSP_LACST Snake venom serine protease OS=Lachesis stenophrys OX=88085 PE=2 SV=1	Q072L7	292,352
DN33023_c0_g1	VEGFA_PROFL Vascular endothelial growth factor A OS=Protobothrops flavoviridis OX=88087 PE=1 SV=1	P67860	290,812
DN33023_c0_g1	VEGFA_PROFL Vascular endothelial growth factor A OS=Protobothrops flavoviridis OX=88087 PE=1 SV=1	P67860	290,041
DN34088_c1_g1	VM1B_BOTIN Snake venom metalloproteinase BITM02A OS=Bothrops insularis OX=8723 PE=2 SV=1	Q8QG89	289,271
DN29729_c1_g1	VSPB_GLOBL Beta-fibrinogenase brevinase OS=Gloydius blomhoffii OX=242054 PE=1 SV=1	Q9PT51	287,345
DN32061_c4_g1	SL1A_BOTJA Snaclec GPIB-binding protein subunit alpha OS=Bothrops jararaca OX=8724 PE=1 SV=1	Q9PSM6	285,804
DN34152_c6_g1	VSP14_BOTJA Snake venom serine protease HS114 OS=Bothrops jararaca OX=8724 PE=1 SV=1	Q5W959	283,878
DN31365_c0_g1	BNP1_BOTJA Bradykinin-potentiating and C-type natriuretic peptides OS=Bothrops jararaca OX=8724 PE=1 SV=1	Q6LEM5	283,108
DN33554_c8_g1	VM34_CROAD Zinc metalloproteinase-disintegrin-like 4a OS=Crotalus adamanteus OX=8729 PE=1 SV=1	F8S108	281,952
DN18834_c0_g1	FCNV1_VARKO Veficolin-1 (Fragment) OS=Varanus komodoensis OX=61221 PE=2 SV=1	E2IYB3	281,567
DN11376_c0_g1	FCNV3_CERRY Ryncolin-3 OS=Cerberus rynchops OX=46267 PE=1 SV=1	D8VNS9	280,796
DN32310_c0_g1	VSP1_BOTJA Platelet-aggregating proteinase PA-BJ (Fragment) OS=Bothrops jararaca OX=8724 PE=1 SV=2	P81824	278,1
DN33311_c1_g1	PA2B1_BOTJR Basic phospholipase A2 homolog bothropstoxin-1 OS=Bothrops jararacussu OX=8726 PE=1 SV=3	Q90249	277,33
DN44891_c0_g1	FA5_PSETE Coagulation factor V OS=Pseudonaja textilis OX=8673 GN=F5 PE=1 SV=1	Q593B6	275,789
DN33023_c0_g1	VEGFA_PROFL Vascular endothelial growth factor A OS=Protobothrops flavoviridis OX=88087 PE=1 SV=1	P67860	273,092
DN25855_c0_g1	VM34_CROADZinc metalloproteinase-disintegrin-like 4a OS=Crotalus adamanteus OX=8729 PE=1 SV=1	F8S108	273,092
DN33554_c8_g1	VM3LB_BOTLC Zinc metalloproteinase leucurolysin-B (Fragment) OS=Bothrops leucurus OX=157295 PE=1 SV=1	P86092	271,937
DN21512_c0_g1	FA5V_PSETE Venom prothrombin activator pseutarin-C non-catalytic subunit OS=Pseudonaja textilis OX=8673 PE=1 SV=1	Q7SZN0	270,781
DN31826_c0_g1	VSP14_BOTJA Snake venom serine protease HS114 OS=Bothrops jararaca OX=8724 PE=1 SV=1	Q5W959	270,011
DN31826_c0_g1	VSP14_BOTJA Snake venom serine protease HS114 OS=Bothrops jararaca OX=8724 PE=1 SV=1	Q5W959	268,855
DN32310_c0_g1	VSP_LACST Snake venom serine protease OS=Lachesis stenophrys OX=88085 PE=2 SV=1	Q072L7	268,47
DN33311_c1_g1	PA2A_BOTER Acidic phospholipase A2 BE-I-PLA2 OS=Bothrops erythromelas OX=44710 PE=1 SV=1	Q2HZ28	267,314
DN18627_c0_g1	PDE2_CROAD Venom phosphodiesterase 2 OS=Crotalus adamanteus OX=8729 PE=1 SV=1	J3SBP3	266,544
DN33554_c8_g1	VM34_CROAD Zinc metalloproteinase-disintegrin-like 4a OS=Crotalus adamanteus OX=8729 PE=1 SV=1	F8S108	266,544
DN31371_c0_g1	PA2A_BOTER Acidic phospholipase A2 BE-I-PLA2 OS=Bothrops erythromelas OX=44710 PE=1 SV=1	Q2HZ28	264,618
DN26435_c0_g1	DNA2_ACAPL Plancitoxin-1 OS=Acanthaster planci OX=133434 PE=1 SV=1	Q75WF2	263,848
DN31826_c0_g1	VSP_BOTIN Snake venom serine protease BITS01A OS=Bothrops insularis OX=8723 PE=2 SV=1	Q8QG86	261,922
DN32061_c4_g1	SL1B_BOTJA Snaclec GPIB-binding protein subunit beta OS=Bothrops jararaca OX=8724 PE=1 SV=1	Q9PSM5	261,536
DN34152_c6_g1	VSP14_BOTJA Snake venom serine protease HS114 OS=Bothrops jararaca OX=8724 PE=1 SV=1	Q5W959	261,536
DN26435_c0_g1	DNA2_ACAPL Plancitoxin-1 OS=Acanthaster planci OX=133434 PE=1 SV=1	Q75WF2	261,151
DN34270_c0_g1	ARMT1_PIMH Y-glutamate O-methyltransferase OS=Pimpla hypochondriaca OX=135724 GN=vpr2 PE=1 SV=1	Q8MMH3	260,766
DN32061_c4_g1	SLAB_BOTJA Snaclec bothrojaracin subunit beta OS=Bothrops jararaca OX=8724 PE=1 SV=1	Q56EB0	258,455
DN33219_c0_g1	SL1B_BOTJA Snaclec GPIB-binding protein subunit beta OS=Bothrops jararaca OX=8724 PE=1 SV=1	Q9PSM5	258,07
DN33315_c0_g1	VM3_OPHHA Zinc metalloproteinase-disintegrin-like ohanin OS=Ophiophagus hannah OX=8665 PE=1 SV=1	A3R0T9	258,07
DN34049_c13_g1	VM2JT_PROJR Zinc metalloproteinase-disintegrin jerdonitin OS=Protobothrops jerdonii OX=242841 PE=1 SV=1	P83912	256,529
DN32061_c4_g1	SL1B_BOTJA Snaclec GPIB-binding protein subunit beta OS=Bothrops jararaca OX=8724 PE=1 SV=1	Q9PSM5	255,758
DN33311_c1_g1	PA2A_BOTER Acidic phospholipase A2 BE-I-PLA2 OS=Bothrops erythromelas OX=44710 PE=1 SV=1	Q2HZ28	255,373
DN19574_c0_g1	VM33_BOTATZinc metalloproteinase-disintegrin-like batroxstatin-3 (Fragment) OS=Bothrops atrox OX=8725 PE=2 SV=1	C5H5D4	255,373
DN212_c0_g1	SL_DEIAC Snaclec clone 2100755 OS=Deinagkistrodon acutus OX=36307 PE=2 SV=1	Q8JIV8	253,832
DN33554_c7_g1	VM31_BOTAT Zinc metalloproteinase-disintegrin-like batroxstatin-1 OS=Bothrops atrox OX=8725 PE=2 SV=1	C5H5D2	253,447
DN34152_c5_g1	VSP14_BOTJA Snake venom serine protease HS114 OS=Bothrops jararaca OX=8724 PE=1 SV=1	Q5W959	253,447
DN31371_c0_g1	PA2A_BOTER Acidic phospholipase A2 BE-I-PLA2 OS=Bothrops erythromelas OX=44710 PE=1 SV=1	Q2HZ28	253,062
DN33219_c0_g1	SLAB_DEIAC Snaclec anticoagulant protein subunit B OS=Deinagkistrodon acutus OX=36307 PE=1 SV=1	Q9DEF8	252,677
DN14583_c0_g1	TCTP_CROHD Translationally-controlled tumor protein homolog OS=Crotalus horridus OX=35024 PE=2 SV=1	T1DKS4	251,906
DN34152_c5_g1	VSP14_BOTJA Snake venom serine protease HS114 OS=Bothrops jararaca OX=8724 PE=1 SV=1	Q5W959	251,906
DN33311_c1_g1	PA2A_BOTER Acidic phospholipase A2 BE-I-PLA2 OS=Bothrops erythromelas OX=44710 PE=1 SV=1	Q2HZ28	251,136
DN33311_c1_g1	PA2A_BOTIN Acidic phospholipase A2 BITP01A OS=Bothrops insularis OX=8723 PE=1 SV=1	Q8QG87	249,98
DN33311_c1_g1	PA2A_BOTIN Acidic phospholipase A2 BITP01A OS=Bothrops insularis OX=8723 PE=1 SV=1	Q8QG87	248,825
DN33641_c0_g1	PDE2_CROAD Venom phosphodiesterase 2 OS=Crotalus adamanteus OX=8729 PE=1 SV=1	J3SBP3	248,054
DN32061_c4_g1	SL1A_BOTJA Snaclec GPIB-binding protein subunit alpha OS=Bothrops jararaca OX=8724 PE=1 SV=1	Q9PSM6	244,588
DN32061_c4_g1	SLAB_DEIAC Snaclec anticoagulant protein subunit B OS=Deinagkistrodon acutus OX=36307 PE=1 SV=1	Q9DEF8	242,662
DN33219_c0_g1	SLAB_DEIAC Snaclec anticoagulant protein subunit B OS=Deinagkistrodon acutus OX=36307 PE=1 SV=1	Q9DEF8	242,276
DN32406_c0_g1	CYT2_CROAD Cystatin-2 OS=Crotalus adamanteus OX=8729 PE=2 SV=1	J3SE80	239,195
DN4812_c0_g1	VDDP4_VESVU Venom dipeptidyl peptidase 4 OS=Vespula vulgaris OX=7454 PE=1 SV=1	B1A4F7	238,81
DN33311_c1_g1	PA2A_BOTIN Acidic phospholipase A2 BITP01A OS=Bothrops insularis OX=8723 PE=1 SV=1	Q8QG87	238,424
DN32061_c4_g1	SL1A_BOTJA Snaclec GPIB-binding protein subunit alpha OS=Bothrops jararaca OX=8724 PE=1 SV=1	Q9PSM6	238,424

Sequence Name	Sequence Description	Blast Top Hit Accession	Blast Top Hit Score
DN33554_c8_g1	VM2V2_CROAT Zinc metalloproteinase-disintegrin VMP-II OS=Crotalus atrox OX=8730 PE=2 SV=1	C9E1R7	238,039
DN34049_c13_g1	VM28_CROAD Zinc metalloproteinase-disintegrin 8 OS=Crotalus adamanteus OX=8729 PE=1 SV=1	J3SBP9	236,498
DN33554_c8_g1	VM31_CRODCZinc metalloproteinase-disintegrin-like crotastatin (Fragment) OS=Crotalus durissus cascavella OX=184540 PE=2 SV=1	C5H5D1	236,113
DN34040_c1_g1	VSPH_BOTJR Snake venom serine protease homolog OS=Bothrops jararacussu OX=8726 PE=1 SV=1	Q7T229	235,728
DN30554_c0_g1	PA2B1_BOTJR Basic phospholipase A2 homolog bothropstoxin-1 OS=Bothrops jararacussu OX=8726 PE=1 SV=3	Q90249	233,417
DN22973_c0_g1	CYT1_CROAD Cystatin-1 OS=Crotalus adamanteus OX=8729 PE=2 SV=1	J3RYX9	231,106
DN1659_c0_g1	VA3_SOLIN Venom allergen 3 OS=Solenopsis invicta OX=13686 PE=1 SV=2	P35778	229,565
DN32310_c0_g1	VSP_BOTIN Snake venom serine protease BITS01A OS=Bothrops insularis OX=8723 PE=2 SV=1	Q8QG86	228,409
DN28929_c0_g1	PLB_CROAD Phospholipase B OS=Crotalus adamanteus OX=8729 PE=1 SV=1	F8S101	227,639
DN32061_c4_g1	SLAB_DEIAC Snaclec anticoagulant protein subunit B OS=Deinagkistrodon acutus OX=36307 PE=1 SV=1	Q9DEF8	227,639
DN17389_c0_g1	VM2SA_GLOSA Zinc metalloproteinase/disintegrin OS=Gloydius saxatilis OX=92067 PE=2 SV=1	Q7SZE0	227,639
DN8141_c0_g1	VM3BE_BOTER Zinc metalloproteinase-disintegrin-like berythractivase OS=Bothrops erythromelas OX=44710 PE=1 SV=1	Q8UVG0	227,254
DN27478_c0_g1	FA5V_OXYSUVenom prothrombin activator oscutarin-C non-catalytic subunit OS=Oxyuranus scutellatus OX=8668 PE=1 SV=1	Q58L91	226,868
DN26435_c0_g1	DNA2_ACAPL Plancitoxin-1 OS=Acanthaster planci OX=133434 PE=1 SV=1	Q75WF2	223,402
DN34049_c16_g1	VM32_LACMR Zinc metalloproteinase-disintegrin-like lachestatin-2 OS=Lachesis muta rhombeata OX=60219 PE=2 SV=1	C5H5D6	223,016
DN18185_c0_g1	RCN2V_CROAD Reticulocalbin-2 OS=Crotalus adamanteus OX=8729 PE=1 SV=1	J3S9D9	222,246
DN33614_c0_g1	VCO3_CROAD Venom factor OS=Crotalus adamanteus OX=8729 PE=2 SV=1	J3S836	221,861
DN33554_c8_g1	VM3B2_BOTJA Zinc metalloproteinase-disintegrin-like bothrojarin-2 (Fragment) OS=Bothrops jararaca OX=8724 PE=1 SV=1	Q0NZX9	220,705
DN18185_c0_g1	RCN2V_CROAD Reticulocalbin-2 OS=Crotalus adamanteus OX=8729 PE=1 SV=1	J3S9D9	220,32
DN33554_c8_g1	VM32_LACMRZinc metalloproteinase-disintegrin-like lachestatin-2 OS=Lachesis muta rhombeata OX=60219 PE=2 SV=1	C5H5D6	219,935
DN25680_c0_g1	VM3BE_BOTER Zinc metalloproteinase-disintegrin-like berythractivase OS=Bothrops erythromelas OX=44710 PE=1 SV=1	Q8UVG0	219,55
DN33554_c8_g1	VM3B2_BOTJA Zinc metalloproteinase-disintegrin-like bothrojarin-2 (Fragment) OS=Bothrops jararaca OX=8724 PE=1 SV=1	Q0NZX9	218,394
DN30385_c1_g1	VM33_BOTAT Zinc metalloproteinase-disintegrin-like batroxstatin-3 (Fragment) OS=Bothrops atrox OX=8725 PE=2 SV=1	C5H5D4	218,009
DN25855_c0_g1	VM31_BOTAT Zinc metalloproteinase-disintegrin-like batroxstatin-1 OS=Bothrops atrox OX=8725 PE=2 SV=1	C5H5D2	217,624
DN33554_c8_g1	VM3B2_BOTJA Zinc metalloproteinase-disintegrin-like bothrojarin-2 (Fragment) OS=Bothrops jararaca OX=8724 PE=1 SV=1	Q0NZX9	217,624
DN32061_c4_g1	SLAB_DEIAC Snaclec anticoagulant protein subunit B OS=Deinagkistrodon acutus OX=36307 PE=1 SV=1	Q9DEF8	216,853
DN41645_c0_g1	FA5_PSETE Coagulation factor V OS=Pseudonaja textilis OX=8673 GN=F5 PE=1 SV=1	Q593B6	216,468
DN21739_c0_g1	VM31_BOTAT Zinc metalloproteinase-disintegrin-like batroxstatin-1 OS=Bothrops atrox OX=8725 PE=2 SV=1	C5H5D2	215,698
DN33554_c7_g1	VM2IA_BOTIN Zinc metalloproteinase/disintegrin OS=Bothrops insularis OX=8723 PE=1 SV=1	Q5XUW8	213,772
DN26273_c0_g1	VM32B_GLOBR Zinc metalloproteinase-disintegrin-like brevilysin H2b OS=Gloydius brevicaudus OX=259325 PE=1 SV=1	P0DM90	210,305
DN19230_c0_g1	RCN2V_CROAD Reticulocalbin-2 OS=Crotalus adamanteus OX=8729 PE=1 SV=1	J3S9D9	209,149
DN27340_c0_g1	VSPUI_GLOUS Snake venom serine protease ussurin OS=Gloydius ussuriensis OX=35671 PE=2 SV=2	Q8UUJ2	208,379
DN30385_c1_g1	VM33_BOTAT Zinc metalloproteinase-disintegrin-like batroxstatin-3 (Fragment) OS=Bothrops atrox OX=8725 PE=2 SV=1	C5H5D4	206,453
DN32061_c4_g1	SLAB_BOTJA Snaclec bothrojaracin subunit beta OS=Bothrops jararaca OX=8724 PE=1 SV=1	Q56EB0	206,068
DN32310_c0_g1	VSP1_BOTJA Platelet-aggregating proteinase PA-BJ (Fragment) OS=Bothrops jararaca OX=8724 PE=1 SV=2	P81824	203,371
DN32587_c0_g1	RCN2V_CROAD Reticulocalbin-2 OS=Crotalus adamanteus OX=8729 PE=1 SV=1	J3S9D9	202,601
DN33554_c7_g1	VM2MD_GLOBR Zinc metalloproteinase/disintegrin OS=Gloydius brevicaudus OX=259325 PE=2 SV=1	Q9PVK9	202,216
DN32310_c0_g1	VSP1_BOTJA Platelet-aggregating proteinase PA-BJ (Fragment) OS=Bothrops jararaca OX=8724 PE=1 SV=2	P81824	202,216
DN20896_c0_g1	VM3V3_AGKPL Zinc metalloproteinase-disintegrin-like VMP-III OS=Agkistrodon piscivorus leucostoma OX=459671 PE=2 SV=1	C9E1S0	201,83
DN30385_c1_g1	VM3AA_CROAT Zinc metalloproteinase-disintegrin-like atrolysin-A (Fragment) OS=Crotalus atrox OX=8730 PE=1 SV=1	Q92043	201,445
DN24099_c0_g1	VM3_NAJAT Zinc metalloproteinase-disintegrin-like NaMP OS=Naja atra OX=8656 PE=2 SV=1	A8QL59	198,749
DN33554_c7_g1	VM3G1_TRIGA Zinc metalloproteinase/disintegrin (Fragment) OS=Trimeresurus gramineus OX=8767 PE=1 SV=1	P0C6E8	198,364
DN30385_c1_g1	VM3AA_CROAT Zinc metalloproteinase-disintegrin-like atrolysin-A (Fragment) OS=Crotalus atrox OX=8730 PE=1 SV=1	Q92043	197,208
DN30385_c1_g1	VM3H3_BOTJA Zinc metalloproteinase-disintegrin-like HF3 OS=Bothrops jararaca OX=8724 PE=1 SV=3	Q98UF9	195,282
DN19230_c0_g1	RCN2V_CROAD Reticulocalbin-2 OS=Crotalus adamanteus OX=8729 PE=1 SV=1	J3S9D9	193,356
DN48162_c0_g1	SL_DEIAC Snaclec clone 2100755 OS=Deinagkistrodon acutus OX=36307 PE=2 SV=1	Q8JIV8	191,815
DN32061_c4_g1	SLAA_BOTJA Snaclec bothrojaracin subunit alpha OS=Bothrops jararaca OX=8724 PE=1 SV=1	Q56EB1	188,348
DN33130_c0_g1	FCNV1_CERRY Ryncolin-1 OS=Cerberus rynchops OX=46267 PE=1 SV=1	D8VNS7	187,193
DN33554_c8_g1	VM3H1_CRORU Snake venom metalloproteinase HT-1 (Fragment) OS=Crotalus ruber ruber OX=8736 PE=1 SV=1	Q9PSN7	185,652
DN33130_c0_g1	FCNV1_CERRY Ryncolin-1 OS=Cerberus rynchops OX=46267 PE=1 SV=1	D8VNS7	184,882
DN20299_c0_g1	WAP1_PHIOL Waprin-Phi1 OS=Philodryas olfersii OX=120305 PE=2 SV=1	A7X4K1	182,956
DN71199_c0_g1	ACES_BUNFA Acetylcholinesterase OS=Bungarus fasciatus OX=8613 GN=ACHE PE=1 SV=2	Q92035	182,185
DN29632_c0_g1	PDE2_CROAD Venom phosphodiesterase 2 OS=Crotalus adamanteus OX=8729 PE=1 SV=1	J3SBP3	181,03
DN24576_c0_g1	VCO3_CROAD Venom factor OS=Crotalus adamanteus OX=8729 PE=2 SV=1	J3S836	179,874
DN63578_c0_g1	LECM_THRJA C-type lectin lectoxin-Thr1 OS=Thrasops jacksonii OX=186611 PE=2 SV=1	A7X3Z0	178,718
DN24778_c0_g1	LIPE_CROAD Putative endothelial lipase OS=Crotalus adamanteus OX=8729 PE=2 SV=1	J3RZ81	177,178
DN32587_c0_g1	RCN2V_CROAD Reticulocalbin-2 OS=Crotalus adamanteus OX=8729 PE=1 SV=1	J3S9D9	175,637
DN33130_c0_g1	FCNV1_CERRY Ryncolin-1 OS=Cerberus rynchops OX=46267 PE=1 SV=1	D8VNS7	175,252
DN30385_c1_g1	VM33_BOTAT Zinc metalloproteinase-disintegrin-like batroxstatin-3 (Fragment) OS=Bothrops atrox OX=8725 PE=2 SV=1	C5H5D4	174,866
DN33113_c2_g1	VM2IA_BOTIN Zinc metalloproteinase/disintegrin OS=Bothrops insularis OX=8723 PE=1 SV=1	Q5XUW8	174,481
DN27838_c0_g1	VM1B_BOTIN Snake venom metalloproteinase BITM02A OS=Bothrops insularis OX=8723 PE=2 SV=1	Q8QG89	171,014
DN27838_c0_g1	VM2MD_GLOBR Zinc metalloproteinase/disintegrin OS=Gloydius brevicaudus OX=259325 PE=2 SV=1	Q9PVK9	170,629
DN32661_c0_g1	LCTA_LATTR Alpha-latrocrustotoxin-Lt1a (Fragment) OS=Latrodectus tredecimguttatus OX=6925 PE=2 SV=2	Q9XZC0	169,474
DN17557_c0_g1	BNP1_BOTJA Bradykinin-potentiating and C-type natriuretic peptides OS=Bothrops jararaca OX=8724 PE=1 SV=1	Q6LEM5	169,088
DN32661_c0_g1	LCTA_LATTR Alpha-latrocrustotoxin-Lt1a (Fragment) OS=Latrodectus tredecimguttatus OX=6925 PE=2 SV=2	Q9XZC0	169,088
DN32661_c0_g1	LCTA_LATTR Alpha-latrocrustotoxin-Lt1a (Fragment) OS=Latrodectus tredecimguttatus OX=6925 PE=2 SV=2	Q9XZC0	168,703
DN27838_c0_g1	VM2AB_AGKCO Zinc metalloproteinase/disintegrin OS=Agkistrodon contortrix contortrix OX=8713 PE=1 SV=1	Q805F6	168,703
DN27838_c0_g1	VM2MC_GLOBR Zinc metalloproteinase/disintegrin OS=Gloydius brevicaudus OX=259325 PE=1 SV=1	Q9YI19	168,703
DN27838_c0_g1	VM2MD_GLOBR Zinc metalloproteinase/disintegrin OS=Gloydius brevicaudus OX=259325 PE=2 SV=1	Q9PVK9	168,318
DN27838_c0_g1	VM2MC_GLOBR Zinc metalloproteinase/disintegrin OS=Gloydius brevicaudus OX=259325 PE=1 SV=1	Q9YI19	167,548
DN27838_c0_g1	VM2MD_GLOBR Zinc metalloproteinase/disintegrin OS=Gloydius brevicaudus OX=259325 PE=2 SV=1	Q9PVK9	167,162
DN33311_c1_g1	PA2A_BOTMO Acidic phospholipase A2 BmooPLA2 OS=Bothrops moojeni OX=98334 PE=1 SV=1	G3DT18	166,007
DN27838_c0_g1	VM2MC_GLOBR Zinc metalloproteinase/disintegrin OS=Gloydius brevicaudus OX=259325 PE=1 SV=1	Q9YI19	166,007
DN43182_c0_g1	VM3_NAJAT Zinc metalloproteinase-disintegrin-like NaMP OS=Naja atra OX=8656 PE=2 SV=1	A8QL59	166,007
DN34088_c1_g1	VM2J2_BOTJA Zinc metalloproteinase/disintegrin OS=Bothrops jararaca OX=8724 PE=1 SV=1	Q98SP2	164,466
DN34088_c1_g1	VM2V2_CROVV Zinc metalloproteinase/disintegrin VMP-II OS=Crotalus viridis viridis OX=8742 PE=2 SV=1	C9E1R9	164,081
DN34088_c1_g1	VM2J2_BOTJAZinc metalloproteinase/disintegrin OS=Bothrops jararaca OX=8724 PE=1 SV=1	Q98SP2	163,696
DN33311_c1_g1	PA2A_BOTIN Acidic phospholipase A2 BITP01A OS=Bothrops insularis OX=8723 PE=1 SV=1	Q8QG87	160,999

Sequence Name	Sequence Description	Blast Top Hit Accession	Blast Top Hit Score
DN3083_c0_g1	TREA_PIMHY Trehalase OS=Pimpla hypochondriaca OX=135724 GN=tre1 PE=1 SV=1	Q8MMG9	160,999
DN15795_c0_g1	VCO32_AUSSU A.superbus venom factor 2 OS=Austrelaps superbus OX=29156 PE=2 SV=1	A0RZC6	159,073
DN21921_c0_g2	FCNV4_CERRY Ryncolin-4 OS=Cerberus rynchops OX=46267 PE=1 SV=1	D8VNT0	157,532
DN32863_c0_g1	VDPP4_APIME Venom dipeptidyl peptidase 4 OS=Apis mellifera OX=7460 PE=1 SV=1	B2D0J4	157,147
DN12952_c2_g1	VM3AD_AGKCL Zinc metalloproteinase-disintegrin-like ACLD OS=Agkistrodon contortrix laticinctus OX=37195 PE=2 SV=1	O42138	156,762
DN31063_c0_g1	LITD_LATTR Delta-latroinsectotoxin-Lt1a OS=Latrodectus tredecimguttatus OX=6925 PE=1 SV=1	Q25338	155,606
DN28191_c0_g1	SLA_PROFL Snaclec coagulation factor IX-binding protein subunit A OS=Protobothrops flavoviridis OX=88087 PE=1 SV=1	Q7LZ71	155,606
DN29230_c0_g1	BLTX_BLABR Blarina toxin OS=Blarina brevicauda OX=9387 GN=BTX PE=1 SV=1	Q76B45	153,68
DN25382_c0_g1	BNP_BOTIN Bradykinin-potentiating and C-type natriuretic peptides OS=Bothrops insularis OX=8723 PE=1 SV=2	P68515	152,525
DN33378_c0_g1	FAXD2_NOTSC Venom prothrombin activator notecarin-D2 OS=Notechis scutatus scutatus OX=70142 PE=1 SV=1	Q58L94	152,14
DN32661_c0_g1	LCTA_LATTR Alpha-latrocrustotoxin-Lt1a (Fragment) OS=Latrodectus tredecimguttatus OX=6925 PE=2 SV=2	Q9XZC0	150,214
DN32661_c0_g1	LCTA_LATTR Alpha-latrocrustotoxin-Lt1a (Fragment) OS=Latrodectus tredecimguttatus OX=6925 PE=2 SV=2	Q9XZC0	150,214
DN32661_c0_g1	LCTA_LATTR Alpha-latrocrustotoxin-Lt1a (Fragment) OS=Latrodectus tredecimguttatus OX=6925 PE=2 SV=2	Q9XZC0	150,214
DN67117_c0_g1	ACES_BUNFA Acetylcholinesterase OS=Bungarus fasciatus OX=8613 GN=ACHE PE=1 SV=2	Q92035	149,058
DN33075_c1_g1	PA23_HELSU Phospholipase A2 isozymes PA3A/PA3B/PA5 OS=Heloderma suspectum OX=8554 PE=1 SV=3	P16354	149,058
DN32376_c0_g1	VDPP4_APIME Venom dipeptidyl peptidase 4 OS=Apis mellifera OX=7460 PE=1 SV=1	B2D0J4	149,058
DN31959_c1_g1	VM2V2_CROVV Zinc metalloproteinase/disintegrin VMP-II OS=Crotalus viridis viridis OX=8742 PE=2 SV=1	C9E1R9	149,058
DN32376_c0_g1	VDPP4_APIME Venom dipeptidyl peptidase 4 OS=Apis mellifera OX=7460 PE=1 SV=1	B2D0J4	148,673
DN31959_c1_g1	VM1A_AGKCL Snake venom metalloproteinase ACLF OS=Agkistrodon contortrix laticinctus OX=37195 GN=ACLPREF PE=1 SV=1	Q92031	148,673
DN25382_c0_g1	BNP1_BOTJA Bradykinin-potentiating and C-type natriuretic peptides OS=Bothrops jararaca OX=8724 PE=1 SV=1	Q6LEM5	147,132
DN33980_c0_g3	CYT_NAJKA Cystatin OS=Naja kaouthia OX=8649 PE=2 SV=1	E3P6P4	147,132
DN36382_c0_g1	FCNV4_CERRYRyncolin-4 OS=Cerberus rynchops OX=46267 PE=1 SV=1	D8VNT0	147,132
DN31959_c1_g1	VM3SB_TRIST Zinc metalloproteinase-disintegrin-like stejnihagin-B OS=Trimeresurus stejnegeri OX=39682 PE=2 SV=1	Q3HTN2	146,747
DN31959_c1_g1	VM2V2_CROVV Zinc metalloproteinase/disintegrin VMP-II OS=Crotalus viridis viridis OX=8742 PE=2 SV=1	C9E1R9	145,206
DN52466_c0_g1	PDE1_CROAD Venom phosphodiesterase 1 OS=Crotalus adamanteus OX=8729 PE=1 SV=2	J3SEZ3	144,436
DN31959_c1_g1	VM3HB_PROFL Zinc metalloproteinase-disintegrin-like HR1b OS=Protobothrops flavoviridis OX=88087 PE=1 SV=4	P20164	144,436
DN44012_c0_g1	TCTP_LOXIN Translationally-controlled tumor protein homolog OS=Loxosceles intermedia OX=58218 PE=2 SV=1	G3LU44	143,28
DN32376_c0_g1	VDPP4_APIME Venom dipeptidyl peptidase 4 OS=Apis mellifera OX=7460 PE=1 SV=1	B2D0J4	139,813
DN32855_c0_g1	FA10_TROCA Coagulation factor X OS=Tropidechis carinatus OX=100989 GN=F10 PE=2 SV=1	Q4QXT9	139,043
DN50260_c0_g1	FCNV4_CERRY Ryncolin-4 OS=Cerberus rynchops OX=46267 PE=1 SV=1	D8VNT0	137,117
DN33641_c0_g1	PDE1_CROAD Venom phosphodiesterase 1 OS=Crotalus adamanteus OX=8729 PE=1 SV=2	J3SEZ3	136,732
DN5343_c0_g1	VA3_SOLIN Venom allergen 3 OS=Solenopsis invicta OX=13686 PE=1 SV=2	P35778	136,346
DN30398_c0_g1	ACPH1_APIME Venom acid phosphatase Acph-1 OS=Apis mellifera OX=7460 PE=1 SV=1	Q5BLY5	134,806
DN32855_c0_g1	ISOHC_AGEAP Venom peptide isomerase heavy chain OS=Agelenopsis aperta OX=6908 PE=1 SV=1	Q9TXD8	133,65
DN32855_c0_g1	ISOHC_AGEAP Venom peptide isomerase heavy chain OS=Agelenopsis aperta OX=6908 PE=1 SV=1	Q9TXD8	133,265
DN12144_c0_g1	LATA_LATTR Alpha-latrotoxin-Lt1a OS=Latrodectus tredecimguttatus OX=6925 PE=1 SV=2	P23631	133,265
DN34747_c0_g1	LCTA_LATTR Alpha-latrocrustotoxin-Lt1a (Fragment) OS=Latrodectus tredecimguttatus OX=6925 PE=2 SV=2	Q9XZC0	132,88
DN26118_c0_g1	SL_DEIAC Snaclec clone 2100755 OS=Deinagkistrodon acutus OX=36307 PE=2 SV=1	Q8JIV8	132,88
DN33075_c1_g1	PA23_HELSU Phospholipase A2 isozymes PA3A/PA3B/PA5 OS=Heloderma suspectum OX=8554 PE=1 SV=3	P16354	132,494
DN20749_c0_g1	ISOHC_AGEAP Venom peptide isomerase heavy chain OS=Agelenopsis aperta OX=6908 PE=1 SV=1	Q9TXD8	132,109
DN40083_c0_g1	LECG_BOTIN C-type lectin BiL OS=Bothrops insularis OX=8723 PE=1 SV=1	Q6QX33	131,724
DN30445_c0_g1	VCP_APIME Venom serine carboxypeptidase OS=Apis mellifera OX=7460 PE=2 SV=1	C9WMM5	131,724
DN35389_c0_g1	LCTA_LATTR Alpha-latrocrustotoxin-Lt1a (Fragment) OS=Latrodectus tredecimguttatus OX=6925 PE=2 SV=2	Q9XZC0	130,954
DN71617_c0_g1	CALGL_TROCA Calglandulin OS=Tropidechis carinatus OX=100989 PE=2 SV=1	Q3SB11	130,568
DN25722_c0_g1	CALGL_TROCA Calglandulin OS=Tropidechis carinatus OX=100989 PE=2 SV=1	Q3SB11	130,568
DN47777_c0_g1	LITD_LATTR Delta-latroinsectotoxin-Lt1a OS=Latrodectus tredecimguttatus OX=6925 PE=1 SV=1	Q25338	129,798
DN33219_c0_g1	SL1A_BOTJA Snaclec GPIB-binding protein subunit alpha OS=Bothrops jararaca OX=8724 PE=1 SV=1	Q9PSM6	129,798
DN32602_c0_g1	VM3_BUNFA Zinc metalloproteinase-disintegrin-like BfMP (Fragment) OS=Bungarus fasciatus OX=8613 PE=2 SV=1	A8QL48	129,413
DN28901_c0_g1	FAXD2_DEMVE Venom prothrombin activator vestarin-D2 OS=Demansia vestigiata OX=412038 PE=1 SV=1	A6MFK8	129,028
DN34086_c4_g3	ISOHC_AGEAP Venom peptide isomerase heavy chain OS=Agelenopsis aperta OX=6908 PE=1 SV=1	Q9TXD8	129,028
DN33015_c0_g1	LITA_LATTR Alpha-latroinsectotoxin-Lt1a (Fragment) OS=Latrodectus tredecimguttatus OX=6925 PE=1 SV=1	Q02989	127,872
DN49754_c0_g1	VM3M1_NAJMO Snake venom metalloproteinase-disintegrin-like mocarhagin OS=Naja mossambica OX=8644 PE=1 SV=3	Q10749	127,102
DN29657_c0_g1	LITA_LATTR Alpha-latroinsectotoxin-Lt1a (Fragment) OS=Latrodectus tredecimguttatus OX=6925 PE=1 SV=1	Q02989	126,331
DN56183_c0_g1	STXB_SYNVE Neoverrucotoxin subunit beta OS=Synanceia verrucosa OX=51996 PE=1 SV=1	A0ZSK4	123,635
DN19282_c0_g1	FA5V_PSETE Venom prothrombin activator pseutarin-C non-catalytic subunit OS=Pseudonaja textilis OX=8673 PE=1 SV=1	Q7SZN0	122,865
DN26118_c0_g1	LECM2_ERYPO C-type lectin lectoxin-Lio2 OS=Erythrolamprus poecilogyrus OX=338838 PE=2 SV=1	A7X3Z7	122,865
DN17557_c0_g1	BNP_BOTJR Bradykinin-potentiating and C-type natriuretic peptides isoform 2 (Fragment) OS=Bothrops jararacussu OX=8726 PE=1 SV=1	Q7T1M3	122,094
DN35238_c0_g1	VM34_CROAD Zinc metalloproteinase-disintegrin-like 4a OS=Crotalus adamanteus OX=8729 PE=1 SV=1	F8S108	120,553
DN5077_c0_g1	SLTB_PROMU Snaclec trimecetin subunit beta OS=Protobothrops mucrosquamatus OX=103944 PE=1 SV=1	Q5FZI5	120,168
DN32855_c0_g1	FAXD2_DEMVE Venom prothrombin activator vestarin-D2 OS=Demansia vestigiata OX=412038 PE=1 SV=1	A6MFK8	119,783
DN48458_c0_g1	LATA_LATTR Alpha-latrotoxin-Lt1a OS=Latrodectus tredecimguttatus OX=6925 PE=1 SV=2	P23631	119,398
DN23379_c0_g1	STXA_SYNHO Stonustoxin subunit alpha OS=Synanceia horrida OX=13279 PE=1 SV=3	Q98989	119,398
DN30250_c0_g1	FAXD2_NOTSC Venom prothrombin activator notecarin-D2 OS=Notechis scutatus scutatus OX=70142 PE=1 SV=1	Q58L94	117,857
DN8935_c0_g1	TREA_PIMHY Trehalase OS=Pimpla hypochondriaca OX=135724 GN=tre1 PE=1 SV=1	Q8MMG9	116,701
DN58758_c0_g1	ACES_BUNFA Acetylcholinesterase OS=Bungarus fasciatus OX=8613 GN=ACHE PE=1 SV=2	Q92035	115,546
DN32967_c0_g1	VKT_AUSLA Putative Kunitz-type serine protease inhibitor OS=Austrelaps labialis OX=471292 PE=2 SV=1	B2BS84	114,775
DN19445_c0_g1	CALGL_BOTIN Calglandulin OS=Bothrops insularis OX=8723 PE=2 SV=1	Q8AY75	114,39
DN28629_c0_g1	NEP_TRILK Neprilysin-1 OS=Trittame loki OX=1295018 PE=1 SV=1	W4VS99	114,005
DN30546_c0_g1	FAXC_OXYMIVenom prothrombin activator omicarin-C catalytic subunit OS=Oxyuranus microlepidotus OX=111177 PE=2 SV=1	Q58L95	112,849
DN41491_c0_g1	FCNV3_CERRY Ryncolin-3 OS=Cerberus rynchops OX=46267 PE=1 SV=1	D8VNS9	112,849
DN18788_c0_g1	LATA_LATHE Alpha-latrotoxin-Lhe1a OS=Latrodectus hesperus OX=256737 PE=1 SV=2	P0DJE3	112,464
DN39236_c0_g1	IGFBP_CUPSA Insulin-like growth factor-binding protein-related protein 1 OS=Cupiennius salei OX=6928 PE=2 SV=1	G4V4G1	112,079
DN21627_c0_g1	PA2_APICC Phospholipase A2 OS=Apis cerana cerana OX=94128 PE=2 SV=1	Q9BMK4	112,079
DN32855_c0_g1	FAXD2_DEMVE Venom prothrombin activator vestarin-D2 OS=Demansia vestigiata OX=412038 PE=1 SV=1	A6MFK8	111,694
DN71808_c0_g1	VCP_APIME Venom serine carboxypeptidase OS=Apis mellifera OX=7460 PE=2 SV=1	C9WMM5	111,694
DN33569_c0_g1	LCTA_LATTR Alpha-latrocrustotoxin-Lt1a (Fragment) OS=Latrodectus tredecimguttatus OX=6925 PE=2 SV=2	Q9XZC0	110,538
DN28218_c0_g1	VM3H6_GLOBR Zinc metalloproteinase-disintegrin-like brevilysin H6 OS=Gloydius brevicaudus OX=259325 PE=1 SV=2	P0C7B0	109,768
DN30459_c0_g1	RCN2V_CROAD Reticulocalbin-2 OS=Crotalus adamanteus OX=8729 PE=1 SV=1	J3S9D9	109,383

Sequence Name	Sequence Description	Blast Top Hit Accession	Blast Top Hit Score
DN30106_c0_g1	ISOHC_AGEAP Venom peptide isomerase heavy chain OS=Agelenopsis aperta OX=6908 PE=1 SV=1	Q9TXD8	108,997
DN48052_c0_g1	LATA_LATHE Alpha-latrotoxin-Lhe1a OS=Latrodectus hesperus OX=256737 PE=1 SV=2	P0DJE3	108,997
DN4528_c0_g1	FA5V_OXYMI Venom prothrombin activator omicarin-C non-catalytic subunit OS=Oxyuranus microlepidotus OX=111177 PE=2 SV=1	Q58L90	106,686
DN33930_c0_g1	OXLA_DEMVE L-amino-acid oxidase OS=Demansia vestigiata OX=412038 PE=2 SV=1	A6MFL0	106,301
DN21737_c0_g1	CALGL_TROCA Calglandulin OS=Tropidechis carinatus OX=100989 PE=2 SV=1	Q3SB11	105,916
DN24798_c0_g1	LITD_LATTR Delta-latroinsectotoxin-Lt1a OS=Latrodectus tredecimguttatus OX=6925 PE=1 SV=1	Q25338	105,916
DN39723_c0_g1	NGFV1_NAJSP Venom nerve growth factor 1 OS=Naja sputatrix OX=33626 PE=1 SV=1	Q5YF90	105,916
DN18796_c0_g1	LATA_LATHA Alpha-latrotoxin-Lh1a (Fragment) OS=Latrodectus hasseltii OX=256736 PE=1 SV=2	G0LXV8	105,145
DN55702_c0_g1	VMPA5_LOXGA Astacin-like metalloprotease toxin 5 (Fragment) OS=Loxosceles gaucho OX=58216 PE=2 SV=1	P0DM62	104,76
DN33130_c0_g1	FCNV1_CERRY Ryncolin-1 OS=Cerberus rynchops OX=46267 PE=1 SV=1	D8VNS7	104,375
DN4812_c0_g1	VDDP4_VESVU Venom dipeptidyl peptidase 4 OS=Vespula vulgaris OX=7454 PE=1 SV=1	B1A4F7	103,99
DN31055_c0_g1	VKT3_BITGA Kunitz-type serine protease inhibitor bitisilin-3 (Fragment) OS=Bitis gabonica OX=8694 PE=2 SV=1	Q6T269	103,605
DN65532_c0_g1	LITD_LATTR Delta-latroinsectotoxin-Lt1a OS=Latrodectus tredecimguttatus OX=6925 PE=1 SV=1	Q25338	102,834
DN30193_c0_g1	LCTA_LATTR Alpha-latrocrustotoxin-Lt1a (Fragment) OS=Latrodectus tredecimguttatus OX=6925 PE=2 SV=2	Q9XZC0	102,064
DN32847_c1_g1	FA5V_PSETE Venom prothrombin activator pseutarin-C non-catalytic subunit OS=Pseudonaja textilis OX=8673 PE=1 SV=1	Q7SZN0	100,908
DN15502_c0_g1	VKT1_TRILK Kunitz-type U19-barytoxin-Tl1a OS=Trittame loki OX=1295018 PE=2 SV=2	W4VSH9	100,908
DN71079_c0_g1	LCTA_LATTR Alpha-latrocrustotoxin-Lt1a (Fragment) OS=Latrodectus tredecimguttatus OX=6925 PE=2 SV=2	Q9XZC0	100,523
DN9764_c0_g1	PDE1_CROAD Venom phosphodiesterase 1 OS=Crotalus adamanteus OX=8729 PE=1 SV=2	J3SEZ3	100,523
DN35176_c0_g1	TREA_PIMHY Trehalase OS=Pimpla hypochondriaca OX=135724 GN=tre1 PE=1 SV=1	Q8MMG9	100,523
DN54675_c0_g1	VM3_BUNFA Zinc metalloproteinase-disintegrin-like BfMP (Fragment) OS=Bungarus fasciatus OX=8613 PE=2 SV=1	A8QL48	100,523
DN25112_c0_g1	LCTA_LATTR Alpha-latrocrustotoxin-Lt1a (Fragment) OS=Latrodectus tredecimguttatus OX=6925 PE=2 SV=2	Q9XZC0	100,138
DN2414_c0_g1	FA5V_PSETE Venom prothrombin activator pseutarin-C non-catalytic subunit OS=Pseudonaja textilis OX=8673 PE=1 SV=1	Q7SZN0	99,3673
DN27134_c0_g1	LATA_LATTR Alpha-latrotoxin-Lt1a OS=Latrodectus tredecimguttatus OX=6925 PE=1 SV=2	P23631	99,3673
DN25112_c0_g1	LCTA_LATTR Alpha-latrocrustotoxin-Lt1a (Fragment) OS=Latrodectus tredecimguttatus OX=6925 PE=2 SV=2	Q9XZC0	98,9821
DN30651_c0_g1	PA2BN_CROVV Basic phospholipase A2 Cvv-N6 OS=Crotalus viridis viridis OX=8742 PE=1 SV=1	Q71QE8	97,4413
DN25112_c0_g1	LCTA_LATTR Alpha-latrocrustotoxin-Lt1a (Fragment) OS=Latrodectus tredecimguttatus OX=6925 PE=2 SV=2	Q9XZC0	97,0561
DN15620_c0_g1	LECM1_HYDHA C-type lectin 1 OS=Hydrophis hardwickii OX=8781 PE=2 SV=1	A3FM55	97,0561
DN4060_c0_g1	SP4_BOMPE Venom protease OS=Bombus pensylvanicus OX=28643 PE=1 SV=1	Q7M4I3	96,6709
DN30651_c0_g1	PA2BN_CROVV Basic phospholipase A2 Cvv-N6 OS=Crotalus viridis viridis OX=8742 PE=1 SV=1	Q71QE8	96,2857
DN27922_c0_g1	VESP_LACMU Ohanin-like protein OS=Lachesis muta muta OX=8753 PE=2 SV=1	Q27J48	94,3597
DN27922_c0_g1	VESP_LACMU Ohanin-like protein OS=Lachesis muta muta OX=8753 PE=2 SV=1	Q27J48	94,3597
DN26656_c0_g1	VESP_POGBA Vespryn (Fragment) OS=Pogona barbata OX=52202 PE=2 SV=1	Q2XXL4	94,3597
DN11007_c0_g1	LCTA_LATTR Alpha-latrocrustotoxin-Lt1a (Fragment) OS=Latrodectus tredecimguttatus OX=6925 PE=2 SV=2	Q9XZC0	93,5893
DN34734_c0_g1	LCTA_LATTR Alpha-latrocrustotoxin-Lt1a (Fragment) OS=Latrodectus tredecimguttatus OX=6925 PE=2 SV=2	Q9XZC0	93,2041
DN12235_c0_g1	LITA_LATTR Alpha-latroinsectotoxin-Lt1a (Fragment) OS=Latrodectus tredecimguttatus OX=6925 PE=1 SV=1	Q02989	92,4337
DN76371_c0_g1	CALGL_TROCA Calglandulin OS=Tropidechis carinatus OX=100989 PE=2 SV=1	Q3SB11	92,0485
DN68378_c0_g1	FCNV4_CERRY Ryncolin-4 OS=Cerberus rynchops OX=46267 PE=1 SV=1	D8VNT0	90,8929
DN16806_c0_g1	VM39_DRYCN Zinc metalloproteinase-disintegrin-like MTP9 OS=Drysdalia coronoides OX=66186 PE=1 SV=1	F8RKV9	90,8929
DN19880_c0_g1	FAXD1_NOTSC Venom prothrombin activator notecarin-D1 OS=Notechis scutatus scutatus OX=70142 PE=1 SV=2	P82807	90,5077
DN9214_c0_g1	LCTA_LATTR Alpha-latrocrustotoxin-Lt1a (Fragment) OS=Latrodectus tredecimguttatus OX=6925 PE=2 SV=2	Q9XZC0	89,7373
DN10635_c0_g1	LITA_LATTR Alpha-latroinsectotoxin-Lt1a (Fragment) OS=Latrodectus tredecimguttatus OX=6925 PE=1 SV=1	Q02989	88,5817
DN32651_c0_g1	VESP_LACMU Ohanin-like protein OS=Lachesis muta muta OX=8753 PE=2 SV=1	Q27J48	88,1965
DN32651_c0_g1	VESP_LACMU Ohanin-like protein OS=Lachesis muta muta OX=8753 PE=2 SV=1	Q27J48	88,1965
DN31446_c0_g1	LATA_LATHA Alpha-latrotoxin-Lh1a (Fragment) OS=Latrodectus hasseltii OX=256736 PE=1 SV=2	G0LXV8	87,8113
DN32651_c0_g1	VESP_LACMU Ohanin-like protein OS=Lachesis muta muta OX=8753 PE=2 SV=1	Q27J48	87,8113
DN27635_c0_g1	CALGL_BOTINCalglandulin OS=Bothrops insularis OX=8723 PE=2 SV=1	Q8AY75	87,4261
DN31446_c0_g1	LATA_LATHA Alpha-latrotoxin-Lh1a (Fragment) OS=Latrodectus hasseltii OX=256736 PE=1 SV=2	G0LXV8	87,4261
DN31446_c0_g1	LITA_LATTR Alpha-latroinsectotoxin-Lt1a (Fragment) OS=Latrodectus tredecimguttatus OX=6925 PE=1 SV=1	Q02989	87,4261
DN33573_c0_g1	STXA_SYNHO Stonustoxin subunit alpha OS=Synanceia horrida OX=13279 PE=1 SV=3	Q98989	87,4261
DN13692_c0_g1	FA102_PSETE Coagulation factor X isoform 2 OS=Pseudonaja textilis OX=8673 GN=F10 PE=1 SV=1	Q1L658	87,0409
DN13765_c0_g1	SP4_BOMPE Venom protease OS=Bombus pensylvanicus OX=28643 PE=1 SV=1	Q7M4I3	87,0409
DN35331_c0_g1	VEGFA_BITGA Vascular endothelial growth factor A OS=Bitis gabonica OX=8694 PE=1 SV=1	P83906	86,2705
DN21358_c0_g1	LATA_LATTR Alpha-latrotoxin-Lt1a OS=Latrodectus tredecimguttatus OX=6925 PE=1 SV=2	P23631	85,8853
DN21358_c0_g1	LCTA_LATTR Alpha-latrocrustotoxin-Lt1a (Fragment) OS=Latrodectus tredecimguttatus OX=6925 PE=2 SV=2	Q9XZC0	85,8853
DN29791_c0_g1	VESP_POGBA Vespryn (Fragment) OS=Pogona barbata OX=52202 PE=2 SV=1	Q2XXL4	85,8853
DN28972_c0_g1	LATA_LATHA Alpha-latrotoxin-Lh1a (Fragment) OS=Latrodectus hasseltii OX=256736 PE=1 SV=2	G0LXV8	85,1149
DN21474_c0_g1	CALGL_BOTIN Calglandulin OS=Bothrops insularis OX=8723 PE=2 SV=1	Q8AY75	84,7297
DN28835_c0_g1	FA5V_PSETE Venom prothrombin activator pseutarin-C non-catalytic subunit OS=Pseudonaja textilis OX=8673 PE=1 SV=1	Q7SZN0	84,7297
DN33417_c0_g1	LCTA_LATTR Alpha-latrocrustotoxin-Lt1a (Fragment) OS=Latrodectus tredecimguttatus OX=6925 PE=2 SV=2	Q9XZC0	83,9593
DN13363_c0_g1	FAXC_PSETE Venom prothrombin activator pseutarin-C catalytic subunit OS=Pseudonaja textilis OX=8673 PE=1 SV=2	Q56VR3	83,5741
DN42187_c0_g1	LATA_LATTR Alpha-latrotoxin-Lt1a OS=Latrodectus tredecimguttatus OX=6925 PE=1 SV=2	P23631	83,5741
DN51683_c0_g1	SP4_BOMPE Venom protease OS=Bombus pensylvanicus OX=28643 PE=1 SV=1	Q7M4I3	83,5741
DN33573_c0_g1	STXA_SYNVE Neoverrucotoxin subunit alpha OS=Synanceia verrucosa OX=51996 PE=1 SV=1	A0ZSK3	83,5741
DN13881_c0_g1	VESP_POGBA Vespryn (Fragment) OS=Pogona barbata OX=52202 PE=2 SV=1	Q2XXL4	83,1889
DN57971_c0_g1	LATA_LATHA Alpha-latrotoxin-Lh1a (Fragment) OS=Latrodectus hasseltii OX=256736 PE=1 SV=2	G0LXV8	82,8037
DN34103_c0_g1	VESP_DRYCNVespryn-21 OS=Drysdalia coronoides OX=66186 PE=1 SV=1	F8RKW2	82,8037
DN33234_c1_g1	VSP_BOMIG Venom serine protease Bi-VSP OS=Bombus ignitus OX=130704 PE=1 SV=1	B5U2W0	82,8037
DN19677_c0_g1	LATA_LATTR Alpha-latrotoxin-Lt1a OS=Latrodectus tredecimguttatus OX=6925 PE=1 SV=2	P23631	82,4185
DN28578_c0_g1	VKT3_BITGA Kunitz-type serine protease inhibitor bitisilin-3 (Fragment) OS=Bitis gabonica OX=8694 PE=2 SV=1	Q6T269	81,6481
DN55258_c0_g1	IGFBP_CUPSA Insulin-like growth factor-binding protein-related protein 1 OS=Cupiennius salei OX=6928 PE=2 SV=1	G4V4G1	80,8777
DN33666_c0_g1	LCTA_LATTR Alpha-latrocrustotoxin-Lt1a (Fragment) OS=Latrodectus tredecimguttatus OX=6925 PE=2 SV=2	Q9XZC0	80,8777
DN33666_c0_g1	LCTA_LATTR Alpha-latrocrustotoxin-Lt1a (Fragment) OS=Latrodectus tredecimguttatus OX=6925 PE=2 SV=2	Q9XZC0	80,8777
DN9195_c0_g1	LECM4_PSEPL C-type lectin lectoxin-Enh4 OS=Pseudoferania polylepis OX=338839 PE=2 SV=1	A7X3X3	80,8777
DN6327_c0_g1	PA1_SOLIN Phospholipase A1 OS=Solenopsis invicta OX=13686 PE=1 SV=1	Q68KK0	80,8777
DN68249_c0_g1	CALGL_TROCA Calglandulin OS=Tropidechis carinatus OX=100989 PE=2 SV=1	Q3SB11	80,1073
DN18229_c0_g1	LECG_PSEAU C-type lectin galactose-binding isoform OS=Pseudechis australis OX=8670 PE=1 SV=1	D2YVI2	79,7221
DN34011_c0_g3	LCTA_LATTR Alpha-latrocrustotoxin-Lt1a (Fragment) OS=Latrodectus tredecimguttatus OX=6925 PE=2 SV=2	Q9XZC0	78,9518
DN26008_c0_g1	ISOHC_AGEAP Venom peptide isomerase heavy chain OS=Agelenopsis aperta OX=6908 PE=1 SV=1	Q9TXD8	78,5666
DN34011_c0_g3	LCTA_LATTR Alpha-latrocrustotoxin-Lt1a (Fragment) OS=Latrodectus tredecimguttatus OX=6925 PE=2 SV=2	Q9XZC0	78,5666

Sequence Name	Sequence Description	Blast Top Hit Accession	Blast Top Hit Score
DN56728_c0_g1	VCO31_AUSSU A.superbus venom factor 1 OS=Austrelaps superbus OX=29156 PE=1 SV=1	Q0ZZJ6	78,1814
DN33084_c0_g1	VESP_DRYCN Vespryn-21 OS=Drysdalia coronoides OX=66186 PE=1 SV=1	F8RKW2	78,1814
DN28080_c0_g1	VM2SA_GLOSA Zinc metalloproteinase/disintegrin OS=Gloydius saxatilis OX=92067 PE=2 SV=1	Q7SZE0	78,1814
DN33084_c0_g1	VESP_DRYCN Vespryn-21 OS=Drysdalia coronoides OX=66186 PE=1 SV=1	F8RKW2	77,7962
DN33084_c0_g1	VESP_DRYCN Vespryn-21 OS=Drysdalia coronoides OX=66186 PE=1 SV=1	F8RKW2	77,7962
DN39423_c0_g1	LITA_LATTR Alpha-latroinsectotoxin-Lt1a (Fragment) OS=Latrodectus tredecimguttatus OX=6925 PE=1 SV=1	Q02989	77,411
DN32186_c0_g1	LITD_LATTR Delta-latroinsectotoxin-Lt1a OS=Latrodectus tredecimguttatus OX=6925 PE=1 SV=1	Q25338	77,0258
DN27715_c0_g1	LITA_LATTR Alpha-latroinsectotoxin-Lt1a (Fragment) OS=Latrodectus tredecimguttatus OX=6925 PE=1 SV=1	Q02989	76,6406
DN41116_c0_g1	HYAL_CROAD Hyaluronidase OS=Crotalus adamanteus OX=8729 PE=1 SV=1	J3S820	76,2554
DN28984_c0_g1	LITA_LATTR Alpha-latroinsectotoxin-Lt1a (Fragment) OS=Latrodectus tredecimguttatus OX=6925 PE=1 SV=1	Q02989	75,8702
DN34103_c0_g1	VESP_OPHHA Ohanin OS=Ophiophagus hannah OX=8665 PE=1 SV=2	P83234	75,8702
DN34103_c0_g1	VESP_OPHHA Ohanin OS=Ophiophagus hannah OX=8665 PE=1 SV=2	P83234	75,485
DN31108_c0_g1	VKT21_HETCR Kunitz-type serine protease inhibitor HCRG21 OS=Heteractis crispa OX=175771 PE=3 SV=1	P0DL86	75,485
DN45218_c0_g1	FA5V_PSETEVenom prothrombin activator pseutarin-C non-catalytic subunit OS=Pseudonaja textilis OX=8673 PE=1 SV=1	Q7SZN0	75,0998
DN31981_c0_g1	VESP_OPHHA Ohanin OS=Ophiophagus hannah OX=8665 PE=1 SV=2	P83234	74,3294
DN30432_c0_g1	VKT3A_ACTEQ PI-actitoxin-Aeq3a OS=Actinia equina OX=6106 PE=1 SV=1	P0DMW6	74,3294
DN30432_c0_g1	VKT3A_ACTEQ PI-actitoxin-Aeq3a OS=Actinia equina OX=6106 PE=1 SV=1	P0DMW6	74,3294
DN33666_c0_g1	LITA_LATTR Alpha-latroinsectotoxin-Lt1a (Fragment) OS=Latrodectus tredecimguttatus OX=6925 PE=1 SV=1	Q02989	73,9442
DN48244_c0_g1	ISOHC_AGEAP Venom peptide isomerase heavy chain OS=Agelenopsis aperta OX=6908 PE=1 SV=1	Q9TXD8	73,559
DN33288_c1_g1	LCTA_LATTR Alpha-latrocrustotoxin-Lt1a (Fragment) OS=Latrodectus tredecimguttatus OX=6925 PE=2 SV=2	Q9XZC0	73,559
DN30294_c0_g1	LITA_LATTR Alpha-latroinsectotoxin-Lt1a (Fragment) OS=Latrodectus tredecimguttatus OX=6925 PE=1 SV=1	Q02989	73,559
DN30294_c0_g1	LITA_LATTR Alpha-latroinsectotoxin-Lt1a (Fragment) OS=Latrodectus tredecimguttatus OX=6925 PE=1 SV=1	Q02989	73,559
DN4108_c0_g1	CALGL_BOTIN Calglandulin OS=Bothrops insularis OX=8723 PE=2 SV=1	Q8AY75	73,1738
DN23171_c0_g1	VKT3_BITGA Kunitz-type serine protease inhibitor bitisilin-3 (Fragment) OS=Bitis gabonica OX=8694 PE=2 SV=1	Q6T269	73,1738
DN15225_c0_g1	FAXC_PSETE Venom prothrombin activator pseutarin-C catalytic subunit OS=Pseudonaja textilis OX=8673 PE=1 SV=2	Q56VR3	72,7886
DN33288_c1_g1	LCTA_LATTR Alpha-latrocrustotoxin-Lt1a (Fragment) OS=Latrodectus tredecimguttatus OX=6925 PE=2 SV=2	Q9XZC0	72,7886
DN34080_c0_g1	LCTA_LATTR Alpha-latrocrustotoxin-Lt1a (Fragment) OS=Latrodectus tredecimguttatus OX=6925 PE=2 SV=2	Q9XZC0	72,7886
DN31981_c0_g1	VESP_POGBA Vespryn (Fragment) OS=Pogona barbata OX=52202 PE=2 SV=1	Q2XXL4	72,7886
DN17645_c0_g1	LATA_LATTR Alpha-latrotoxin-Lt1a OS=Latrodectus tredecimguttatus OX=6925 PE=1 SV=2	P23631	72,4034
DN17497_c1_g1	PRDX4_CROAT Peroxiredoxin-4 (Fragments) OS=Crotalus atrox OX=8730 PE=1 SV=1	P0CV91	72,4034
DN14187_c0_g1	VCO3_NAJKA Cobra venom factor OS=Naja kaouthia OX=8649 PE=1 SV=1	Q91132	72,4034
DN31981_c0_g1	VESP_POGBA Vespryn (Fragment) OS=Pogona barbata OX=52202 PE=2 SV=1	Q2XXL4	72,4034
DN22327_c0_g1	VESP_POGBA Vespryn (Fragment) OS=Pogona barbata OX=52202 PE=2 SV=1	Q2XXL4	72,4034
DN15522_c0_g1	CALGL_BOTIN Calglandulin OS=Bothrops insularis OX=8723 PE=2 SV=1	Q8AY75	72,0182
DN33075_c1_g1	PA23_HELSU Phospholipase A2 isozymes PA3A/PA3B/PA5 OS=Heloderma suspectum OX=8554 PE=1 SV=3	P16354	72,0182
DN22327_c0_g1	VESP_POGBA Vespryn (Fragment) OS=Pogona barbata OX=52202 PE=2 SV=1	Q2XXL4	72,0182
DN20394_c0_g1	VM31_BOTAT Zinc metalloproteinase-disintegrin-like batroxstatin-1 OS=Bothrops atrox OX=8725 PE=2 SV=1	C5H5D2	72,0182
DN36548_c0_g1	FCNV1_VARKO Veficolin-1 (Fragment) OS=Varanus komodoensis OX=61221 PE=2 SV=1	E2IYB3	71,633
DN26356_c0_g1	LITD_LATTR Delta-latroinsectotoxin-Lt1a OS=Latrodectus tredecimguttatus OX=6925 PE=1 SV=1	Q25338	71,633
DN2933_c0_g1	VCO31_AUSSU A.superbus venom factor 1 OS=Austrelaps superbus OX=29156 PE=1 SV=1	Q0ZZJ6	71,633
DN31664_c0_g1	VKT3_ANESU KappaPI-actitoxin-Avd3d OS=Anemonia sulcata OX=6108 PE=1 SV=1	Q9TWF8	71,633
DN34083_c2_g1	VESP_LACMU Ohanin-like protein OS=Lachesis muta muta OX=8753 PE=2 SV=1	Q27J48	71,2478
DN29695_c0_g1	VESP_LACMU Ohanin-like protein OS=Lachesis muta muta OX=8753 PE=2 SV=1	Q27J48	71,2478
DN34011_c0_g3	LCTA_LATTR Alpha-latrocrustotoxin-Lt1a (Fragment) OS=Latrodectus tredecimguttatus OX=6925 PE=2 SV=2	Q9XZC0	70,8626
DN31152_c1_g1	STXA_SYNVE Neoverrucotoxin subunit alpha OS=Synanceia verrucosa OX=51996 PE=1 SV=1	A0ZSK3	70,4774
DN6214_c0_g1	CALGL_BOTIN Calglandulin OS=Bothrops insularis OX=8723 PE=2 SV=1	Q8AY75	70,0922
DN27315_c0_g1	FAXD1_DEMVE Venom prothrombin activator vestarin-D1 OS=Demansia vestigiata OX=412038 PE=1 SV=1	A6MFK7	70,0922
DN58795_c0_g1	LATA_LATTR Alpha-latrotoxin-Lt1a OS=Latrodectus tredecimguttatus OX=6925 PE=1 SV=2	P23631	69,3218
DN34115_c0_g1	LECG1_BUNFA C-type lectin BfL-1 OS=Bungarus fasciatus OX=8613 PE=2 SV=1	Q90WI8	69,3218
DN29313_c0_g1	LCTA_LATTR Alpha-latrocrustotoxin-Lt1a (Fragment) OS=Latrodectus tredecimguttatus OX=6925 PE=2 SV=2	Q9XZC0	68,5514
DN30947_c0_g1	LCTA_LATTR Alpha-latrocrustotoxin-Lt1a (Fragment) OS=Latrodectus tredecimguttatus OX=6925 PE=2 SV=2	Q9XZC0	68,1662
DN8520_c0_g1	LITA_LATTR Alpha-latroinsectotoxin-Lt1a (Fragment) OS=Latrodectus tredecimguttatus OX=6925 PE=1 SV=1	Q02989	68,1662
DN47426_c0_g1	LITD_LATTR Delta-latroinsectotoxin-Lt1a OS=Latrodectus tredecimguttatus OX=6925 PE=1 SV=1	Q25338	68,1662
DN21474_c0_g1	CALGL_BOTIN Calglandulin OS=Bothrops insularis OX=8723 PE=2 SV=1	Q8AY75	67,3958
DN33916_c0_g1	CALGL_TROCA Calglandulin OS=Tropidechis carinatus OX=100989 PE=2 SV=1	Q3SB11	67,0106
DN73304_c0_g1	LCTA_LATTR Alpha-latrocrustotoxin-Lt1a (Fragment) OS=Latrodectus tredecimguttatus OX=6925 PE=2 SV=2	Q9XZC0	67,0106
DN34103_c0_g1	VESP_CROAD Vespryn OS=Crotalus adamanteus OX=8729 PE=1 SV=1	F8S122	67,0106
DN41039_c0_g1	NEP_TRILK Neprilysin-1 OS=Trittame loki OX=1295018 PE=1 SV=1	W4VS99	66,6254
DN31824_c0_g1	FAXD2_NOTSCVenom prothrombin activator notecarin-D2 OS=Notechis scutatus scutatus OX=70142 PE=1 SV=1	Q58L94	66,2402
DN14716_c0_g1	PRDX4_CROAT Peroxiredoxin-4 (Fragments) OS=Crotalus atrox OX=8730 PE=1 SV=1	P0CV91	66,2402
DN24675_c0_g1	PRDX4_CROAT Peroxiredoxin-4 (Fragments) OS=Crotalus atrox OX=8730 PE=1 SV=1	P0CV91	66,2402
DN27899_c0_g1	LATA_LATHA Alpha-latrotoxin-Lh1a (Fragment) OS=Latrodectus hasseltii OX=256736 PE=1 SV=2	G0LXV8	65,855
DN36389_c0_g1	LITD_LATTR Delta-latroinsectotoxin-Lt1a OS=Latrodectus tredecimguttatus OX=6925 PE=1 SV=1	Q25338	65,855
DN33916_c0_g1	CALGL_TROCA Calglandulin OS=Tropidechis carinatus OX=100989 PE=2 SV=1	Q3SB11	65,4698
DN128_c0_g1	LATA_LATHE Alpha-latrotoxin-Lhe1a OS=Latrodectus hesperus OX=256737 PE=1 SV=2	P0DJE3	65,0846
DN76440_c0_g1	LCTA_LATTR Alpha-latrocrustotoxin-Lt1a (Fragment) OS=Latrodectus tredecimguttatus OX=6925 PE=2 SV=2	Q9XZC0	64,6994
DN24249_c0_g1	CRVP5_VARAC Cysteine-rich venom protein VAR5 (Fragment) OS=Varanus acanthurus OX=62035 PE=2 SV=1	Q2XXR0	64,3142
DN9747_c0_g1	LATA_LATTR Alpha-latrotoxin-Lt1a OS=Latrodectus tredecimguttatus OX=6925 PE=1 SV=2	P23631	64,3142
DN34103_c0_g1	VESP_CROAD Vespryn OS=Crotalus adamanteus OX=8729 PE=1 SV=1	F8S122	64,3142
DN445_c0_g1	FA10_TROCA Coagulation factor X OS=Tropidechis carinatus OX=100989 GN=F10 PE=2 SV=1	Q4QXT9	63,929
DN17073_c0_g1	LECM1_ERYPO C-type lectin lectoxin-Lio1 OS=Erythrolamprus poecilogyrus OX=338838 PE=2 SV=1	A7X3Z4	63,929
DN73585_c0_g1	FCNV1_VARKO Veficolin-1 (Fragment) OS=Varanus komodoensis OX=61221 PE=2 SV=1	E2IYB3	63,5438
DN13979_c0_g1	LCTA_LATTR Alpha-latrocrustotoxin-Lt1a (Fragment) OS=Latrodectus tredecimguttatus OX=6925 PE=2 SV=2	Q9XZC0	63,5438
DN33776_c0_g1	LITD_LATTR Delta-latroinsectotoxin-Lt1a OS=Latrodectus tredecimguttatus OX=6925 PE=1 SV=1	Q25338	63,5438
DN33776_c0_g1	LITD_LATTR Delta-latroinsectotoxin-Lt1a OS=Latrodectus tredecimguttatus OX=6925 PE=1 SV=1	Q25338	63,5438
DN40332_c0_g1	VEGFA_BITGA Vascular endothelial growth factor A OS=Bitis gabonica OX=8694 PE=1 SV=1	P83906	63,5438
DN15502_c0_g1	VKT3_DEMVE Kunitz-type serine protease inhibitor vestiginin-3 OS=Demansia vestigiata OX=412038 PE=1 SV=1	A6MFL3	62,7734
DN33417_c0_g1	LATA_LATHE Alpha-latrotoxin-Lhe1a OS=Latrodectus hesperus OX=256737 PE=1 SV=2	P0DJE3	62,3882
DN427_c0_g1	LITA_LATTR Alpha-latroinsectotoxin-Lt1a (Fragment) OS=Latrodectus tredecimguttatus OX=6925 PE=1 SV=1	Q02989	62,3882
DN29001_c0_g1	OXLA_SISCA L-amino-acid oxidase OS=Sistrurus catenatus edwardsii OX=8762 PE=2 SV=1	B0VXW0	62,3882

Sequence Name	Sequence Description	Blast Top Hit Accession	Blast Top Hit Score
**DN37088_c0_g1**	SP4_BOMPE Venom protease OS=Bombus pensylvanicus OX=28643 PE=1 SV=1	Q7M4I3	62,3882
**DN14211_c0_g1**	VKT1_ARAVE Kunitz-type U1-aranetoxin-Av1a OS=Araneus ventricosus OX=182803 PE=2 SV=1	Q8T3S7	62,3882
**DN55846_c0_g1**	VKT2_BITGA Kunitz-type serine protease inhibitor bitisilin-2 OS=Bitis gabonica OX=8694 PE=1 SV=1	Q6T6S5	62,3882
**DN44810_c0_g1**	FA5V_OXYMI Venom prothrombin activator omicarin-C non-catalytic subunit OS=Oxyuranus microlepidotus OX=111177 PE=2 SV=1	Q58L90	62,003
**DN56886_c0_g1**	LATA_LATHA Alpha-latrotoxin-Lh1a (Fragment) OS=Latrodectus hasseltii OX=256736 PE=1 SV=2	G0LXV8	62,003
**DN33776_c0_g1**	LITD_LATTR Delta-latroinsectotoxin-Lt1a OS=Latrodectus tredecimguttatus OX=6925 PE=1 SV=1	Q25338	62,003
**DN40861_c0_g1**	CRVP_TRIBI Cysteine-rich venom protein TRI1 (Fragment) OS=Trimorphodon biscutatus OX=338818 PE=2 SV=1	Q2XXP4	61,6178
**DN32439_c0_g1**	LCTA_LATTR Alpha-latrocrustotoxin-Lt1a (Fragment) OS=Latrodectus tredecimguttatus OX=6925 PE=2 SV=2	Q9XZC0	61,6178
**DN65187_c0_g1**	LITD_LATTR Delta-latroinsectotoxin-Lt1a OS=Latrodectus tredecimguttatus OX=6925 PE=1 SV=1	Q25338	60,8474
**DN27424_c0_g1**	LCTA_LATTR Alpha-latrocrustotoxin-Lt1a (Fragment) OS=Latrodectus tredecimguttatus OX=6925 PE=2 SV=2	Q9XZC0	60,4622
**DN57025_c0_g1**	LITD_LATTR Delta-latroinsectotoxin-Lt1a OS=Latrodectus tredecimguttatus OX=6925 PE=1 SV=1	Q25338	60,4622
**DN66608_c0_g1**	AMY_TITSE Alpha-amylase (Fragment) OS=Tityus serrulatus OX=6887 PE=1 SV=1	P85843	60,077
**DN43893_c0_g1**	LCTA_LATTR Alpha-latrocrustotoxin-Lt1a (Fragment) OS=Latrodectus tredecimguttatus OX=6925 PE=2 SV=2	Q9XZC0	60,077
**DN27424_c0_g1**	LCTA_LATTR Alpha-latrocrustotoxin-Lt1a (Fragment) OS=Latrodectus tredecimguttatus OX=6925 PE=2 SV=2	Q9XZC0	60,077
**DN33352_c0_g1**	LCTA_LATTR Alpha-latrocrustotoxin-Lt1a (Fragment) OS=Latrodectus tredecimguttatus OX=6925 PE=2 SV=2	Q9XZC0	60,077
**DN7147_c0_g1**	CALGL_BOTIN Calglandulin OS=Bothrops insularis OX=8723 PE=2 SV=1	Q8AY75	59,6918
**DN67051_c0_g1**	FA5_PSETE Coagulation factor V OS=Pseudonaja textilis OX=8673 GN=F5 PE=1 SV=1	Q593B6	59,6918
**DN32924_c0_g1**	LCTA_LATTR Alpha-latrocrustotoxin-Lt1a (Fragment) OS=Latrodectus tredecimguttatus OX=6925 PE=2 SV=2	Q9XZC0	59,6918
**DN2883_c0_g1**	ACPH1_APIME Venom acid phosphatase Acph-1 OS=Apis mellifera OX=7460 PE=1 SV=1	Q5BLY5	59,3066
**DN50504_c0_g1**	SL1_ECHPL Snaclec 1 OS=Echis pyramidum leakeyi OX=38415 PE=2 SV=1	Q6X5S3	59,3066
**DN74762_c0_g1**	SLCA_DEIAC Snaclec agkicetin-C subunit alpha OS=Deinagkistrodon acutus OX=36307 PE=1 SV=1	Q9DEA2	59,3066
**DN9143_c0_g1**	STXA_SYNVE Neoverrucotoxin subunit alpha OS=Synanceia verrucosa OX=51996 PE=1 SV=1	A0ZSK3	59,3066
**DN34719_c0_g1**	STXA_SYNHO Stonustoxin subunit alpha OS=Synanceia horrida OX=13279 PE=1 SV=3	Q98989	58,9214
**DN5321_c0_g1**	LCTA_LATTR Alpha-latrocrustotoxin-Lt1a (Fragment) OS=Latrodectus tredecimguttatus OX=6925 PE=2 SV=2	Q9XZC0	58,5362
**DN33352_c0_g1**	LCTA_LATTR Alpha-latrocrustotoxin-Lt1a (Fragment) OS=Latrodectus tredecimguttatus OX=6925 PE=2 SV=2	Q9XZC0	58,5362
**DN39198_c0_g1**	LITD_LATTR Delta-latroinsectotoxin-Lt1a OS=Latrodectus tredecimguttatus OX=6925 PE=1 SV=1	Q25338	58,5362
**DN33916_c0_g1**	CALGL_TROCA Calglandulin OS=Tropidechis carinatus OX=100989 PE=2 SV=1	Q3SB11	58,151
**DN40889_c0_g1**	LITD_LATTR Delta-latroinsectotoxin-Lt1a OS=Latrodectus tredecimguttatus OX=6925 PE=1 SV=1	Q25338	58,151
**DN71017_c0_g1**	CALGL_TROCA Calglandulin OS=Tropidechis carinatus OX=100989 PE=2 SV=1	Q3SB11	57,7658
**DN26514_c0_g1**	CYT_BITGA Bitiscystatin OS=Bitis gabonica OX=8694 PE=1 SV=1	Q6T6T4	57,7658
**DN67205_c0_g1**	LATA_LATTR Alpha-latrotoxin-Lt1a OS=Latrodectus tredecimguttatus OX=6925 PE=1 SV=2	P23631	57,7658
**DN26065_c0_g1**	LATA_LATHE Alpha-latrotoxin-Lhe1a OS=Latrodectus hesperus OX=256737 PE=1 SV=2	P0DJE3	57,3806
**DN26065_c0_g1**	LITD_LATTR Delta-latroinsectotoxin-Lt1a OS=Latrodectus tredecimguttatus OX=6925 PE=1 SV=1	Q25338	57,3806
**DN64331_c0_g1**	OXLA_CRODM L-amino acid oxidase Cdc18 (Fragment) OS=Crotalus durissus cumanensis OX=184542 PE=1 SV=1	K9N7B7	57,3806
**DN5633_c0_g1**	RCN2V_CROAD Reticulocalbin-2 OS=Crotalus adamanteus OX=8729 PE=1 SV=1	J3S9D9	57,3806
**DN7956_c0_g1**	VCP_APIME Venom serine carboxypeptidase OS=Apis mellifera OX=7460 PE=2 SV=1	C9WMM5	57,3806
**DN72632_c0_g1**	LCTA_LATTR Alpha-latrocrustotoxin-Lt1a (Fragment) OS=Latrodectus tredecimguttatus OX=6925 PE=2 SV=2	Q9XZC0	56,9954
**DN76600_c0_g1**	LITD_LATTR Delta-latroinsectotoxin-Lt1a OS=Latrodectus tredecimguttatus OX=6925 PE=1 SV=1	Q25338	56,9954
**DN41311_c0_g1**	CALGL_BOTIN Calglandulin OS=Bothrops insularis OX=8723 PE=2 SV=1	Q8AY75	56,6102
**DN42514_c0_g1**	LCTA_LATTR Alpha-latrocrustotoxin-Lt1a (Fragment) OS=Latrodectus tredecimguttatus OX=6925 PE=2 SV=2	Q9XZC0	56,6102
**DN33352_c0_g1**	LCTA_LATTR Alpha-latrocrustotoxin-Lt1a (Fragment) OS=Latrodectus tredecimguttatus OX=6925 PE=2 SV=2	Q9XZC0	56,6102
**DN29853_c0_g1**	LITD_LATTR Delta-latroinsectotoxin-Lt1a OS=Latrodectus tredecimguttatus OX=6925 PE=1 SV=1	Q25338	56,6102
**DN34083_c2_g1**	VESP_LACMU Ohanin-like protein OS=Lachesis muta muta OX=8753 PE=2 SV=1	Q27J48	56,6102
**DN34083_c2_g1**	VESP_LACMU Ohanin-like protein OS=Lachesis muta muta OX=8753 PE=2 SV=1	Q27J48	56,6102
**DN26141_c0_g1**	LITA_LATTR Alpha-latroinsectotoxin-Lt1a (Fragment) OS=Latrodectus tredecimguttatus OX=6925 PE=1 SV=1	Q02989	56,225
**DN31827_c0_g1**	LITD_LATTR Delta-latroinsectotoxin-Lt1a OS=Latrodectus tredecimguttatus OX=6925 PE=1 SV=1	Q25338	56,225
**DN4964_c0_g1**	FA5V_OXYSU Venom prothrombin activator oscutarin-C non-catalytic subunit OS=Oxyuranus scutellatus OX=8668 PE=1 SV=1	Q58L91	55,4546
**DN4964_c0_g1**	FA5V_OXYSU Venom prothrombin activator oscutarin-C non-catalytic subunit OS=Oxyuranus scutellatus OX=8668 PE=1 SV=1	Q58L91	55,4546
**DN16567_c0_g1**	LCTA_LATTR Alpha-latrocrustotoxin-Lt1a (Fragment) OS=Latrodectus tredecimguttatus OX=6925 PE=2 SV=2	Q9XZC0	55,4546
**DN15855_c0_g1**	3SX3_SISCA Three-finger toxin 3 OS=Sistrurus catenatus edwardsii OX=8762 PE=3 SV=1	A5X2W8	55,0694
**DN34009_c1_g1**	LITA_LATTR Alpha-latroinsectotoxin-Lt1a (Fragment) OS=Latrodectus tredecimguttatus OX=6925 PE=1 SV=1	Q02989	55,0694
**DN25445_c0_g1**	PRDX4_CROAT Peroxiredoxin-4 (Fragments) OS=Crotalus atrox OX=8730 PE=1 SV=1	P0CV91	55,0694
**DN15206_c0_g1**	STXA_SYNHO Stonustoxin subunit alpha OS=Synanceia horrida OX=13279 PE=1 SV=3	Q98989	55,0694
**DN19329_c0_g1**	VM3TM_TRIST Zinc metalloproteinase-disintegrin-like TSV-DM OS=Trimeresurus stejnegeri OX=39682 PE=1 SV=1	Q2LD49	55,0694
**DN7164_c0_g1**	CRVP_TELDH Cysteine-rich venom protein TEL1 (Fragment) OS=Telescopus dhara OX=338837 PE=2 SV=1	Q2XXP5	54,6842
**DN56777_c0_g1**	FAXC_OXYSU Venom prothrombin activator oscutarin-C catalytic subunit OS=Oxyuranus scutellatus OX=8668 PE=1 SV=1	Q58L96	54,6842
**DN33478_c0_g1**	LCTA_LATTR Alpha-latrocrustotoxin-Lt1a (Fragment) OS=Latrodectus tredecimguttatus OX=6925 PE=2 SV=2	Q9XZC0	54,6842
**DN25445_c0_g1**	PRDX4_CROAT Peroxiredoxin-4 (Fragments) OS=Crotalus atrox OX=8730 PE=1 SV=1	P0CV91	54,6842
**DN71440_c0_g1**	SRTXL_ATRMM Long-sarafotoxin (Fragment) OS=Atractaspis microlepidota microlepidota OX=172021 PE=1 SV=1	Q6RY98	54,6842
**DN36623_c0_g1**	VM2A2_DEIAC Zinc metalloproteinase/disintegrin OS=Deinagkistrodon acutus OX=36307 GN=wbfib4 PE=1 SV=1	Q9PWJ0	54,6842
**DN26008_c0_g1**	ISOHC_AGEAP Venom peptide isomerase heavy chain OS=Agelenopsis aperta OX=6908 PE=1 SV=1	Q9TXD8	54,299
**DN65829_c0_g1**	LITA_LATTR Alpha-latroinsectotoxin-Lt1a (Fragment) OS=Latrodectus tredecimguttatus OX=6925 PE=1 SV=1	Q02989	54,299
**DN34103_c0_g1**	VESP_CROAD Vespryn OS=Crotalus adamanteus OX=8729 PE=1 SV=1	F8S122	54,299
**DN32855_c0_g1**	VSPB_GLOBL Beta-fibrinogenase brevinase OS=Gloydius blomhoffii OX=242054 PE=1 SV=1	Q9PT51	54,299
**DN75220_c0_g1**	CALGL_BOTIN Calglandulin OS=Bothrops insularis OX=8723 PE=2 SV=1	Q8AY75	53,9138
**DN36568_c0_g1**	LCTA_LATTR Alpha-latrocrustotoxin-Lt1a (Fragment) OS=Latrodectus tredecimguttatus OX=6925 PE=2 SV=2	Q9XZC0	53,9138
**DN56996_c0_g1**	LECM3_ERYPO C-type lectin lectoxin-Lio3 OS=Erythrolamprus poecilogyrus OX=338838 PE=2 SV=1	A7X413	53,9138
**DN7885_c0_g1**	LITD_LATTR Delta-latroinsectotoxin-Lt1a OS=Latrodectus tredecimguttatus OX=6925 PE=1 SV=1	Q25338	53,9138
**DN32855_c0_g1**	ISOHC_AGEAP Venom peptide isomerase heavy chain OS=Agelenopsis aperta OX=6908 PE=1 SV=1	Q9TXD8	53,5286
**DN10731_c0_g1**	VEGFA_AGKPI Vascular endothelial growth factor A OS=Agkistrodon piscivorus piscivorus OX=8716 PE=2 SV=1	C0K3N4	53,5286
**DN42779_c0_g1**	WAPA_TROCA Carwaprin-a OS=Tropidechis carinatus OX=100989 PE=2 SV=1	B5G6H2	53,5286
**DN32855_c0_g1**	ISOHC_AGEAP Venom peptide isomerase heavy chain OS=Agelenopsis aperta OX=6908 PE=1 SV=1	Q9TXD8	53,1434
**DN33478_c0_g1**	LCTA_LATTR Alpha-latrocrustotoxin-Lt1a (Fragment) OS=Latrodectus tredecimguttatus OX=6925 PE=2 SV=2	Q9XZC0	53,1434
**DN33542_c3_g1**	LCTA_LATTR Alpha-latrocrustotoxin-Lt1a (Fragment) OS=Latrodectus tredecimguttatus OX=6925 PE=2 SV=2	Q9XZC0	53,1434
**DN47247_c0_g1**	LITA_LATTR Alpha-latroinsectotoxin-Lt1a (Fragment) OS=Latrodectus tredecimguttatus OX=6925 PE=1 SV=1	Q02989	53,1434
**DN54668_c0_g1**	PN47_PHONI U21-ctenitoxin-Pn1a OS=Phoneutria nigriventer OX=6918 PE=1 SV=2	P84033	52,7582
**DN33385_c0_g1**	LCTA_LATTR Alpha-latrocrustotoxin-Lt1a (Fragment) OS=Latrodectus tredecimguttatus OX=6925 PE=2 SV=2	Q9XZC0	52,373
**DN34009_c1_g1**	LITA_LATTR Alpha-latroinsectotoxin-Lt1a (Fragment) OS=Latrodectus tredecimguttatus OX=6925 PE=1 SV=1	Q02989	52,373

Sequence Name	Sequence Description	Blast Top Hit Accession	Blast Top Hit Score
DN62912_c0_g1	LITA_LATTR Alpha-latroinsectotoxin-Lt1a (Fragment) OS=Latrodectus tredecimguttatus OX=6925 PE=1 SV=1	Q02989	52,373
DN30193_c0_g1	LITD_LATTR Delta-latroinsectotoxin-Lt1a OS=Latrodectus tredecimguttatus OX=6925 PE=1 SV=1	Q25338	52,373
DN33666_c0_g1	FAXC_OXYMI Venom prothrombin activator omicarin-C catalytic subunit OS=Oxyuranus microlepidotus OX=111177 PE=2 SV=1	Q58L95	51,6026
DN24032_c0_g1	LCTA_LATTR Alpha-latrocrustotoxin-Lt1a (Fragment) OS=Latrodectus tredecimguttatus OX=6925 PE=2 SV=2	Q9XZC0	51,6026
DN23823_c0_g1	LCTA_LATTR Alpha-latrocrustotoxin-Lt1a (Fragment) OS=Latrodectus tredecimguttatus OX=6925 PE=2 SV=2	Q9XZC0	51,6026
DN46057_c0_g1	LCTA_LATTR Alpha-latrocrustotoxin-Lt1a (Fragment) OS=Latrodectus tredecimguttatus OX=6925 PE=2 SV=2	Q9XZC0	51,6026
DN34009_c1_g1	LITA_LATTR Alpha-latroinsectotoxin-Lt1a (Fragment) OS=Latrodectus tredecimguttatus OX=6925 PE=1 SV=1	Q02989	51,6026
DN16818_c0_g1	VESP_POGBA Vespryn (Fragment) OS=Pogona barbata OX=52202 PE=2 SV=1	Q2XXL4	51,6026
DN11890_c0_g1	VM3HB_PROFL Zinc metalloproteinase-disintegrin-like HR1b OS=Protobothrops flavoviridis OX=88087 PE=1 SV=4	P20164	51,6026
DN27420_c0_g1	FCNV1_VARKO Veficolin-1 (Fragment) OS=Varanus komodoensis OX=61221 PE=2 SV=1	E2IYB3	51,2174
DN23823_c0_g1	LCTA_LATTR Alpha-latrocrustotoxin-Lt1a (Fragment) OS=Latrodectus tredecimguttatus OX=6925 PE=2 SV=2	Q9XZC0	51,2174
DN34103_c0_g1	VESP_OPHHA Ohanin OS=Ophiophagus hannah OX=8665 PE=1 SV=2	P83234	51,2174
DN25123_c0_g1	CALGL_BOTIN Calglandulin OS=Bothrops insularis OX=8723 PE=2 SV=1	Q8AY75	50,8322
DN36965_c0_g1	FA5_PSETE Coagulation factor V OS=Pseudonaja textilis OX=8673 GN=F5 PE=1 SV=1	Q593B6	50,8322
DN17595_c0_g1	FA5V_OXYMI Venom prothrombin activator omicarin-C non-catalytic subunit OS=Oxyuranus microlepidotus OX=111177 PE=2 SV=1	Q58L90	50,8322
DN47689_c0_g1	FCNV1_VARKO Veficolin-1 (Fragment) OS=Varanus komodoensis OX=61221 PE=2 SV=1	E2IYB3	50,8322
DN29700_c0_g1	CALGL_BOTIN Calglandulin OS=Bothrops insularis OX=8723 PE=2 SV=1	Q8AY75	50,447
DN64419_c0_g1	LATA_LATTR Alpha-latrotoxin-Lt1a OS=Latrodectus tredecimguttatus OX=6925 PE=1 SV=2	P23631	49,6766
DN34011_c0_g3	LCTA_LATTR Alpha-latrocrustotoxin-Lt1a (Fragment) OS=Latrodectus tredecimguttatus OX=6925 PE=2 SV=2	Q9XZC0	49,6766
DN33475_c0_g1	LITD_LATTR Delta-latroinsectotoxin-Lt1a OS=Latrodectus tredecimguttatus OX=6925 PE=1 SV=1	Q25338	49,6766
DN26930_c0_g1	OXLA_PSEAU L-amino-acid oxidase OS=Pseudechis australis OX=8670 PE=1 SV=1	Q4JHE1	49,6766
DN34115_c0_g1	SLUB_DEIAC Snaclec agkisacutacin subunit B OS=Deinagkistrodon acutus OX=36307 PE=1 SV=2	Q8JIW1	49,6766
DN30026_c0_g1	CALGL_BOTIN Calglandulin OS=Bothrops insularis OX=8723 PE=2 SV=1	Q8AY75	49,2914
DN20516_c0_g1	FCNV1_VARKO Veficolin-1 (Fragment) OS=Varanus komodoensis OX=61221 PE=2 SV=1	E2IYB3	49,2914
DN20516_c0_g1	FCNV1_VARKO Veficolin-1 (Fragment) OS=Varanus komodoensis OX=61221 PE=2 SV=1	E2IYB3	49,2914
DN29867_c0_g1	LATA_LATHA Alpha-latrotoxin-Lh1a (Fragment) OS=Latrodectus hasseltii OX=256736 PE=1 SV=2	G0LXV8	49,2914
DN32384_c0_g1	LCTA_LATTR Alpha-latrocrustotoxin-Lt1a (Fragment) OS=Latrodectus tredecimguttatus OX=6925 PE=2 SV=2	Q9XZC0	49,2914
DN20651_c0_g1	VESP_POGBA Vespryn (Fragment) OS=Pogona barbata OX=52202 PE=2 SV=1	Q2XXL4	49,2914
DN57368_c0_g1	CALGL_BOTIN Calglandulin OS=Bothrops insularis OX=8723 PE=2 SV=1	Q8AY75	48,9062
DN25510_c0_g1	LECG_THANI Galactose-specific lectin nattectin OS=Thalassophryne nattereri OX=289382 PE=1 SV=1	Q66S03	48,9062
DN74161_c0_g1	LITD_LATTR Delta-latroinsectotoxin-Lt1a OS=Latrodectus tredecimguttatus OX=6925 PE=1 SV=1	Q25338	48,9062
DN34083_c2_g1	VESP_LACMU Ohanin-like protein OS=Lachesis muta muta OX=8753 PE=2 SV=1	Q27J48	48,9062
DN26949_c0_g1	LCTA_LATTR Alpha-latrocrustotoxin-Lt1a (Fragment) OS=Latrodectus tredecimguttatus OX=6925 PE=2 SV=2	Q9XZC0	48,521
DN31078_c0_g1	CALGL_TROCA Calglandulin OS=Tropidechis carinatus OX=100989 PE=2 SV=1	Q3SB11	48,1358
DN38916_c0_g1	LITD_LATTR Delta-latroinsectotoxin-Lt1a OS=Latrodectus tredecimguttatus OX=6925 PE=1 SV=1	Q25338	48,1358
DN74482_c0_g1	OXLA_BUNMU L-amino-acid oxidase OS=Bungarus multicinctus OX=8616 PE=2 SV=1	A8QL51	48,1358
DN33930_c0_g1	OXLA_DEMVE L-amino-acid oxidase OS=Demansia vestigiata OX=412038 PE=2 SV=1	A6MFL0	48,1358
DN29129_c0_g1	TXVE_DABRR Snake venom vascular endothelial growth factor toxin VR-1 OS=Daboia russelii OX=8707 PE=1 SV=2	P67861	48,1358
DN21765_c0_g1	CALGL_PSEPO Calglandulin OS=Pseudechis porphyriacus OX=8671 PE=2 SV=1	Q3SB08	47,7506
DN17784_c0_g1	CALGL_TROCA Calglandulin OS=Tropidechis carinatus OX=100989 PE=2 SV=1	Q3SB11	47,7506
DN47355_c0_g1	FAXD2_DEMVE Venom prothrombin activator vestarin-D2 OS=Demansia vestigiata OX=412038 PE=1 SV=1	A6MFK8	47,7506
DN34011_c0_g3	LCTA_LATTR Alpha-latrocrustotoxin-Lt1a (Fragment) OS=Latrodectus tredecimguttatus OX=6925 PE=2 SV=2	Q9XZC0	47,7506
DN32941_c0_g1	SLA_BITAR Snaclec bitiscetin subunit alpha OS=Bitis arietans OX=8692 PE=1 SV=1	Q7LZK5	47,7506
DN34076_c13_g1	LCTA_LATTR Alpha-latrocrustotoxin-Lt1a (Fragment) OS=Latrodectus tredecimguttatus OX=6925 PE=2 SV=2	Q9XZC0	47,3654
DN10885_c0_g1	LITA_LATTR Alpha-latroinsectotoxin-Lt1a (Fragment) OS=Latrodectus tredecimguttatus OX=6925 PE=1 SV=1	Q02989	47,3654
DN62980_c0_g1	VSP12_BOTJA Snake venom serine protease HS112 OS=Bothrops jararaca OX=8724 PE=2 SV=1	Q5W960	47,3654
DN19787_c0_g1	FCNV1_VARKO Veficolin-1 (Fragment) OS=Varanus komodoensis OX=61221 PE=2 SV=1	E2IYB3	46,9802
DN32885_c0_g1	IGFBP_CUPSA Insulin-like growth factor-binding protein-related protein 1 OS=Cupiennius salei OX=6928 PE=2 SV=1	G4V4G1	46,9802
DN71749_c0_g1	LATA_LATHA Alpha-latrotoxin-Lh1a (Fragment) OS=Latrodectus hasseltii OX=256736 PE=1 SV=2	G0LXV8	46,9802
DN37884_c0_g1	LECM2_BUNFA C-type lectin BfL-2 OS=Bungarus fasciatus OX=8613 PE=2 SV=1	Q90WI7	46,9802
DN30192_c0_g1	LIPE_CROAD Putative endothelial lipase OS=Crotalus adamanteus OX=8729 PE=2 SV=1	J3RZ81	46,9802
DN60249_c0_g1	FAXD1_DEMVE Venom prothrombin activator vestarin-D1 OS=Demansia vestigiata OX=412038 PE=1 SV=1	A6MFK7	46,595
DN33288_c1_g1	LCTA_LATTR Alpha-latrocrustotoxin-Lt1a (Fragment) OS=Latrodectus tredecimguttatus OX=6925 PE=2 SV=2	Q9XZC0	46,595
DN40675_c0_g1	VESP_POGBA Vespryn (Fragment) OS=Pogona barbata OX=52202 PE=2 SV=1	Q2XXL4	46,595
DN23976_c0_g1	FA10_TROCA Coagulation factor X OS=Tropidechis carinatus OX=100989 GN=F10 PE=2 SV=1	Q4QXT9	46,2098
DN32010_c0_g1	FCNV2_CERRY Ryncolin-2 OS=Cerberus rynchops OX=46267 PE=1 SV=1	D8VNS8	46,2098
DN32010_c0_g1	FCNV2_CERRY Ryncolin-2 OS=Cerberus rynchops OX=46267 PE=1 SV=1	D8VNS8	46,2098
DN32010_c0_g1	FCNV3_CERRYRyncolin-3 OS=Cerberus rynchops OX=46267 PE=1 SV=1	D8VNS9	46,2098
DN32010_c0_g1	FCNV3_CERRY Ryncolin-3 OS=Cerberus rynchops OX=46267 PE=1 SV=1	D8VNS9	46,2098
DN32502_c2_g1	IGFBP_CUPSA Insulin-like growth factor-binding protein-related protein 1 OS=Cupiennius salei OX=6928 PE=2 SV=1	G4V4G1	46,2098
DN32502_c2_g1	IGFBP_CUPSA Insulin-like growth factor-binding protein-related protein 1 OS=Cupiennius salei OX=6928 PE=2 SV=1	G4V4G1	46,2098
DN32502_c2_g1	IGFBP_CUPSA Insulin-like growth factor-binding protein-related protein 1 OS=Cupiennius salei OX=6928 PE=2 SV=1	G4V4G1	46,2098
DN32502_c2_g1	IGFBP_CUPSA Insulin-like growth factor-binding protein-related protein 1 OS=Cupiennius salei OX=6928 PE=2 SV=1	G4V4G1	46,2098
DN63492_c0_g1	ISOHC_AGEAP Venom peptide isomerase heavy chain OS=Agelenopsis aperta OX=6908 PE=1 SV=1	Q9TXD8	46,2098
DN31235_c0_g1	LITA_LATTR Alpha-latroinsectotoxin-Lt1a (Fragment) OS=Latrodectus tredecimguttatus OX=6925 PE=1 SV=1	Q02989	46,2098
DN20957_c0_g1	LITA_LATTR Alpha-latroinsectotoxin-Lt1a (Fragment) OS=Latrodectus tredecimguttatus OX=6925 PE=1 SV=1	Q02989	46,2098
DN19787_c0_g1	FCNV1_VARKO Veficolin-1 (Fragment) OS=Varanus komodoensis OX=61221 PE=2 SV=1	E2IYB3	45,8246
DN33473_c0_g1	VDDP4_VESVU Venom dipeptidyl peptidase 4 OS=Vespula vulgaris OX=7454 PE=1 SV=1	B1A4F7	45,8246
DN31981_c0_g1	VESP_OPHHA Ohanin OS=Ophiophagus hannah OX=8665 PE=1 SV=2	P83234	45,8246
DN30845_c0_g1	SLG_CROAD C-type lectin 16 OS=Crotalus adamanteus OX=8729 PE=1 SV=1	J3S3U6	45,4394
DN26519_c0_g1	TU91_IOTCI Turripeptide Ici9.1 (Fragment) OS=Iotyrris cingulifera OX=553733 PE=2 SV=1	P0DKM8	45,4394
DN33473_c0_g1	VDDP4_VESVU Venom dipeptidyl peptidase 4 OS=Vespula vulgaris OX=7454 PE=1 SV=1	B1A4F7	45,4394
DN31088_c0_g1	VDPP4_APIME Venom dipeptidyl peptidase 4 OS=Apis mellifera OX=7460 PE=1 SV=1	B2D0J4	45,4394
DN31088_c0_g1	VDPP4_APIME Venom dipeptidyl peptidase 4 OS=Apis mellifera OX=7460 PE=1 SV=1	B2D0J4	45,4394
DN31078_c0_g1	CALGL_TROCA Calglandulin OS=Tropidechis carinatus OX=100989 PE=2 SV=1	Q3SB11	45,0542
DN26398_c0_g1	LITA_LATTR Alpha-latroinsectotoxin-Lt1a (Fragment) OS=Latrodectus tredecimguttatus OX=6925 PE=1 SV=1	Q02989	45,0542
DN36284_c0_g1	LITA_LATTR Alpha-latroinsectotoxin-Lt1a (Fragment) OS=Latrodectus tredecimguttatus OX=6925 PE=1 SV=1	Q02989	45,0542
DN8180_c0_g1	FA10_TROCA Coagulation factor X OS=Tropidechis carinatus OX=100989 GN=F10 PE=2 SV=1	Q4QXT9	44,669

Sequence Name	Sequence Description	Blast Top Hit Accession	Blast Top Hit Score
DN42019_c0_g1	FCNV1_VARKO Veficolin-1 (Fragment) OS=Varanus komodoensis OX=61221 PE=2 SV=1	E2IYB3	44,669
DN21525_c0_g1	FCNV1_VARKO Veficolin-1 (Fragment) OS=Varanus komodoensis OX=61221 PE=2 SV=1	E2IYB3	44,669
DN20014_c0_g1	LITD_LATTR Delta-latroinsectotoxin-Lt1a OS=Latrodectus tredecimguttatus OX=6925 PE=1 SV=1	Q25338	44,669
DN28153_c0_g1	ACES_TRILK Acetylcholinesterase-1 OS=Trittame loki OX=1295018 PE=1 SV=1	W4VSJ0	44,2838
DN68349_c0_g1	CTX_SEPES SE-cephalotoxin OS=Sepia esculenta OX=31210 PE=1 SV=1	B2DCR8	44,2838
DN22691_c0_g1	FAXC_OXYSUVenom prothrombin activator oscutarin-C catalytic subunit OS=Oxyuranus scutellatus OX=8668 PE=1 SV=1	Q58L96	44,2838
DN30193_c0_g1	LCTA_LATTR Alpha-latrocrustotoxin-Lt1a (Fragment) OS=Latrodectus tredecimguttatus OX=6925 PE=2 SV=2	Q9XZC0	44,2838
DN33241_c0_g1	FCNV1_VARKO Veficolin-1 (Fragment) OS=Varanus komodoensis OX=61221 PE=2 SV=1	E2IYB3	43,8986
DN17930_c0_g1	FCNV1_VARKO Veficolin-1 (Fragment) OS=Varanus komodoensis OX=61221 PE=2 SV=1	E2IYB3	43,8986
DN5661_c0_g1	LCTA_LATTR Alpha-latrocrustotoxin-Lt1a (Fragment) OS=Latrodectus tredecimguttatus OX=6925 PE=2 SV=2	Q9XZC0	43,8986
DN48704_c0_g1	TU11_LOPAL Turripeptide OL11-like (Fragment) OS=Lophiotoma albina OX=1525239 PE=2 SV=1	P0DKM9	43,8986
DN10438_c0_g1	CALGL_BOTIN Calglandulin OS=Bothrops insularis OX=8723 PE=2 SV=1	Q8AY75	43,5134
DN21474_c0_g1	CALGL_BOTIN Calglandulin OS=Bothrops insularis OX=8723 PE=2 SV=1	Q8AY75	43,5134
DN47993_c0_g1	FA10_TROCA Coagulation factor X OS=Tropidechis carinatus OX=100989 GN=F10 PE=2 SV=1	Q4QXT9	43,5134
DN22708_c0_g1	FAXC_OXYSU Venom prothrombin activator oscutarin-C catalytic subunit OS=Oxyuranus scutellatus OX=8668 PE=1 SV=1	Q58L96	43,5134
DN7638_c0_g1	FAXC_PSETE Venom prothrombin activator pseutarin-C catalytic subunit OS=Pseudonaja textilis OX=8673 PE=1 SV=2	Q56VR3	43,5134
DN23422_c0_g1	FAXD2_DEMVE Venom prothrombin activator vestarin-D2 OS=Demansia vestigiata OX=412038 PE=1 SV=1	A6MFK8	43,5134
DN33259_c0_g1	FCNV1_VARKO Veficolin-1 (Fragment) OS=Varanus komodoensis OX=61221 PE=2 SV=1	E2IYB3	43,5134
DN33259_c0_g1	FCNV1_VARKO Veficolin-1 (Fragment) OS=Varanus komodoensis OX=61221 PE=2 SV=1	E2IYB3	43,5134
DN17930_c0_g1	FCNV1_VARKO Veficolin-1 (Fragment) OS=Varanus komodoensis OX=61221 PE=2 SV=1	E2IYB3	43,5134
DN33721_c0_g1	LCTA_LATTR Alpha-latrocrustotoxin-Lt1a (Fragment) OS=Latrodectus tredecimguttatus OX=6925 PE=2 SV=2	Q9XZC0	43,5134
DN14581_c0_g1	FA10_TROCA Coagulation factor X OS=Tropidechis carinatus OX=100989 GN=F10 PE=2 SV=1	Q4QXT9	43,1282
DN14967_c0_g1	FAXC_PSETE Venom prothrombin activator pseutarin-C catalytic subunit OS=Pseudonaja textilis OX=8673 PE=1 SV=2	Q56VR3	43,1282
DN33259_c0_g1	FCNV1_VARKO Veficolin-1 (Fragment) OS=Varanus komodoensis OX=61221 PE=2 SV=1	E2IYB3	43,1282
DN33259_c0_g1	FCNV1_VARKO Veficolin-1 (Fragment) OS=Varanus komodoensis OX=61221 PE=2 SV=1	E2IYB3	43,1282
DN4939_c0_g1	FCNV1_VARKO Veficolin-1 (Fragment) OS=Varanus komodoensis OX=61221 PE=2 SV=1	E2IYB3	43,1282
DN73218_c0_g1	LCTA_LATTR Alpha-latrocrustotoxin-Lt1a (Fragment) OS=Latrodectus tredecimguttatus OX=6925 PE=2 SV=2	Q9XZC0	43,1282
DN72689_c0_g1	SL5_BITAR Snaclec 5 OS=Bitis arietans OX=8692 PE=1 SV=1	Q6X5T3	43,1282
DN10729_c0_g1	FAXD_PSEPO Venom prothrombin activator porpharin-D OS=Pseudechis porphyriacus OX=8671 PE=2 SV=1	Q58L93	42,743
DN29902_c0_g1	FCNV1_VARKO Veficolin-1 (Fragment) OS=Varanus komodoensis OX=61221 PE=2 SV=1	E2IYB3	42,743
DN33288_c1_g1	LCTA_LATTR Alpha-latrocrustotoxin-Lt1a (Fragment) OS=Latrodectus tredecimguttatus OX=6925 PE=2 SV=2	Q9XZC0	42,743
DN31787_c0_g1	LCTA_LATTR Alpha-latrocrustotoxin-Lt1a (Fragment) OS=Latrodectus tredecimguttatus OX=6925 PE=2 SV=2	Q9XZC0	42,743
DN37356_c0_g1	LITA_LATTR Alpha-latroinsectotoxin-Lt1a (Fragment) OS=Latrodectus tredecimguttatus OX=6925 PE=1 SV=1	Q02989	42,743
DN22335_c0_g1	CALGL_BOTIN Calglandulin OS=Bothrops insularis OX=8723 PE=2 SV=1	Q8AY75	42,3578
DN59592_c0_g1	CTX_SEPES SE-cephalotoxin OS=Sepia esculenta OX=31210 PE=1 SV=1	B2DCR8	42,3578
DN25693_c0_g1	FCNV1_VARKO Veficolin-1 (Fragment) OS=Varanus komodoensis OX=61221 PE=2 SV=1	E2IYB3	42,3578
DN23464_c0_g1	IGFBP_CUPSA Insulin-like growth factor-binding protein-related protein 1 OS=Cupiennius salei OX=6928 PE=2 SV=1	G4V4G1	42,3578
DN33473_c0_g1	VDDP4_VESVU Venom dipeptidyl peptidase 4 OS=Vespula vulgaris OX=7454 PE=1 SV=1	B1A4F7	42,3578
DN33740_c5_g1	3NO2_OPHHA Weak neurotoxin OH-72 (Fragment) OS=Ophiophagus hannah OX=8665 PE=2 SV=1	Q53B61	41,9726
DN13652_c0_g1	FAXD_PSEPO Venom prothrombin activator porpharin-D OS=Pseudechis porphyriacus OX=8671 PE=2 SV=1	Q58L93	41,9726
DN47659_c0_g1	LATA_LATTR Alpha-latrotoxin-Lt1a OS=Latrodectus tredecimguttatus OX=6925 PE=1 SV=2	P23631	41,9726
DN24258_c0_g1	LCTA_LATTR Alpha-latrocrustotoxin-Lt1a (Fragment) OS=Latrodectus tredecimguttatus OX=6925 PE=2 SV=2	Q9XZC0	41,9726
DN24258_c0_g1	LCTA_LATTR Alpha-latrocrustotoxin-Lt1a (Fragment) OS=Latrodectus tredecimguttatus OX=6925 PE=2 SV=2	Q9XZC0	41,9726
DN34115_c0_g1	OXLA_BUNFA L-amino-acid oxidase OS=Bungarus fasciatus OX=8613 PE=1 SV=1	A8QL52	41,9726
DN5655_c0_g1	QPCT_BOIDE Glutaminyl-peptide cyclotransferase OS=Boiga dendrophila OX=46286 GN=QPCT PE=2 SV=1	A7ISW2	41,9726
DN17444_c0_g1	FCNV1_VARKO Veficolin-1 (Fragment) OS=Varanus komodoensis OX=61221 PE=2 SV=1	E2IYB3	41,5874
DN33759_c0_g1	FCNV1_VARKO Veficolin-1 (Fragment) OS=Varanus komodoensis OX=61221 PE=2 SV=1	E2IYB3	41,5874
DN42355_c0_g1	LATA_LATHA Alpha-latrotoxin-Lh1a (Fragment) OS=Latrodectus hasseltii OX=256736 PE=1 SV=2	G0LXV8	41,5874
DN9994_c0_g1	LATA_LATTR Alpha-latrotoxin-Lt1a OS=Latrodectus tredecimguttatus OX=6925 PE=1 SV=2	P23631	41,5874
DN17598_c0_g1	PV22_POMCA Perivitellin-2 31 kDa subunit OS=Pomacea canaliculata OX=400727 PE=1 SV=2	P0C8G7	41,5874
DN38203_c0_g1	FAXD1_DEMVEVenom prothrombin activator vestarin-D1 OS=Demansia vestigiata OX=412038 PE=1 SV=1	A6MFK7	41,2022
DN9496_c0_g1	FAXD1_DEMVE Venom prothrombin activator vestarin-D1 OS=Demansia vestigiata OX=412038 PE=1 SV=1	A6MFK7	41,2022
DN33202_c0_g1	FCNV1_VARKO Veficolin-1 (Fragment) OS=Varanus komodoensis OX=61221 PE=2 SV=1	E2IYB3	41,2022
DN10092_c0_g1	FCNV1_VARKO Veficolin-1 (Fragment) OS=Varanus komodoensis OX=61221 PE=2 SV=1	E2IYB3	41,2022
DN38449_c0_g1	FCNV1_VARKO Veficolin-1 (Fragment) OS=Varanus komodoensis OX=61221 PE=2 SV=1	E2IYB3	41,2022
DN13471_c0_g1	FCNV1_VARKO Veficolin-1 (Fragment) OS=Varanus komodoensis OX=61221 PE=2 SV=1	E2IYB3	41,2022
DN30769_c0_g1	GIG1_STIGIO MEGA-stichotoxin-Sgt1a OS=Stichodactyla gigantea OX=230562 PE=1 SV=1	Q76CA1	41,2022
DN24097_c0_g1	IGFBP_CUPSA Insulin-like growth factor-binding protein-related protein 1 OS=Cupiennius salei OX=6928 PE=2 SV=1	G4V4G1	41,2022
DN27174_c0_g1	LECM3_ERYPO C-type lectin lectoxin-Lio3 OS=Erythrolamprus poecilogyrus OX=338838 PE=2 SV=1	A7X413	41,2022
DN29654_c0_g1	LITA_LATTR Alpha-latroinsectotoxin-Lt1a (Fragment) OS=Latrodectus tredecimguttatus OX=6925 PE=1 SV=1	Q02989	41,2022
DN30829_c1_g1	OXLA_BOTJR L-amino-acid oxidase (Fragment) OS=Bothrops jararacussu OX=8726 PE=1 SV=1	Q6TGQ9	41,2022
DN33870_c1_g1	CTX_SEPES SE-cephalotoxin OS=Sepia esculenta OX=31210 PE=1 SV=1	B2DCR8	40,817
DN43528_c0_g1	FAXD_PSEPO Venom prothrombin activator porpharin-D OS=Pseudechis porphyriacus OX=8671 PE=2 SV=1	Q58L93	40,817
DN35224_c0_g1	IGFBP_CUPSA Insulin-like growth factor-binding protein-related protein 1 OS=Cupiennius salei OX=6928 PE=2 SV=1	G4V4G1	40,817
DN54765_c0_g1	LITA_LATTR Alpha-latroinsectotoxin-Lt1a (Fragment) OS=Latrodectus tredecimguttatus OX=6925 PE=1 SV=1	Q02989	40,817
DN21146_c0_g1	VPI1_SCOJE Venom peptide SjAPI OS=Scorpiops jendeki OX=587368 PE=2 SV=1	P0DM55	40,817
DN33067_c0_g1	FAXD2_NOTSC Venom prothrombin activator notecarin-D2 OS=Notechis scutatus scutatus OX=70142 PE=1 SV=1	Q58L94	40,4318
DN32231_c0_g1	HUGAA_VESVU Hyaluronidase A OS=Vespula vulgaris OX=7454 PE=1 SV=1	P49370	40,4318
DN14674_c0_g1	FAXC_OXYMI Venom prothrombin activator omicarin-C catalytic subunit OS=Oxyuranus microlepidotus OX=111177 PE=2 SV=1	Q58L95	40,0466
DN32231_c0_g1	HUGAA_VESVU Hyaluronidase A OS=Vespula vulgaris OX=7454 PE=1 SV=1	P49370	40,0466
DN39579_c0_g1	IGFBP_CUPSA Insulin-like growth factor-binding protein-related protein 1 OS=Cupiennius salei OX=6928 PE=2 SV=1	G4V4G1	40,0466
DN29081_c0_g1	LITA_LATTR Alpha-latroinsectotoxin-Lt1a (Fragment) OS=Latrodectus tredecimguttatus OX=6925 PE=1 SV=1	Q02989	40,0466
DN22179_c0_g1	FAXD1_DEMVE Venom prothrombin activator vestarin-D1 OS=Demansia vestigiata OX=412038 PE=1 SV=1	A6MFK7	39,6614
DN33202_c0_g1	FCNV1_VARKO Veficolin-1 (Fragment) OS=Varanus komodoensis OX=61221 PE=2 SV=1	E2IYB3	39,6614
DN33202_c0_g1	FCNV1_VARKO Veficolin-1 (Fragment) OS=Varanus komodoensis OX=61221 PE=2 SV=1	E2IYB3	39,6614
DN39320_c0_g1	FCNV1_VARKO Veficolin-1 (Fragment) OS=Varanus komodoensis OX=61221 PE=2 SV=1	E2IYB3	39,6614
DN15519_c0_g1	OXLA_TRIST L-amino-acid oxidase OS=Trimeresurus stejnegeri OX=39682 PE=1 SV=1	Q6WP39	39,6614
DN26117_c1_g1	STXB_SYNHO Stonustoxin subunit beta OS=Synanceia horrida OX=13279 PE=1 SV=3	Q91453	39,6614
DN26117_c1_g1	STXB_SYNHO Stonustoxin subunit beta OS=Synanceia horrida OX=13279 PE=1 SV=3	Q91453	39,6614

Sequence Name	Sequence Description	Blast Top Hit Accession	Blast Top Hit Score
DN14242_c0_g1	FCNV1_VARKO Veficolin-1 (Fragment) OS=Varanus komodoensis OX=61221 PE=2 SV=1	E2IYB3	39,2762
DN70945_c0_g1	FCNV1_VARKO Veficolin-1 (Fragment) OS=Varanus komodoensis OX=61221 PE=2 SV=1	E2IYB3	39,2762
DN55055_c0_g1	FCNV1_VARKO Veficolin-1 (Fragment) OS=Varanus komodoensis OX=61221 PE=2 SV=1	E2IYB3	39,2762
DN36993_c0_g1	FCNV1_VARKO Veficolin-1 (Fragment) OS=Varanus komodoensis OX=61221 PE=2 SV=1	E2IYB3	39,2762
DN14338_c0_g1	FCNV1_VARKO Veficolin-1 (Fragment) OS=Varanus komodoensis OX=61221 PE=2 SV=1	E2IYB3	39,2762
DN31350_c0_g1	FCNV3_CERRY Ryncolin-3 OS=Cerberus rynchops OX=46267 PE=1 SV=1	D8VNS9	39,2762
DN31350_c0_g1	FCNV3_CERRY Ryncolin-3 OS=Cerberus rynchops OX=46267 PE=1 SV=1	D8VNS9	39,2762
DN72046_c0_g1	LCTA_LATTR Alpha-latrocrustotoxin-Lt1a (Fragment) OS=Latrodectus tredecimguttatus OX=6925 PE=2 SV=2	Q9XZC0	39,2762
DN22548_c0_g1	RCN2V_CROAD Reticulocalbin-2 OS=Crotalus adamanteus OX=8729 PE=1 SV=1	J3S9D9	39,2762
DN11860_c0_g1	VCO3_CROAD Venom factor OS=Crotalus adamanteus OX=8729 PE=2 SV=1	J3S836	39,2762
DN34427_c0_g1	BLTX_BLABR Blarina toxin OS=Blarina brevicauda OX=9387 GN=BTX PE=1 SV=1	Q76B45	38,891
DN68011_c0_g1	CTX_SEPES SE-cephalotoxin OS=Sepia esculenta OX=31210 PE=1 SV=1	B2DCR8	38,891
DN33202_c0_g1	FCNV1_VARKO Veficolin-1 (Fragment) OS=Varanus komodoensis OX=61221 PE=2 SV=1	E2IYB3	38,891
DN14754_c0_g1	FCNV1_VARKO Veficolin-1 (Fragment) OS=Varanus komodoensis OX=61221 PE=2 SV=1	E2IYB3	38,891
DN58994_c0_g1	FCNV1_VARKO Veficolin-1 (Fragment) OS=Varanus komodoensis OX=61221 PE=2 SV=1	E2IYB3	38,891
DN32501_c0_g1	FCNV1_VARKO Veficolin-1 (Fragment) OS=Varanus komodoensis OX=61221 PE=2 SV=1	E2IYB3	38,891
DN48811_c0_g1	LATA_LATHA Alpha-latrotoxin-Lh1a (Fragment) OS=Latrodectus hasseltii OX=256736 PE=1 SV=2	G0LXV8	38,891
DN21846_c0_g1	LITD_LATTR Delta-latroinsectotoxin-Lt1a OS=Latrodectus tredecimguttatus OX=6925 PE=1 SV=1	Q25338	38,891
DN32633_c1_g1	OXLA_BUNMUL-amino-acid oxidase OS=Bungarus multicinctus OX=8616 PE=2 SV=1	A8QL51	38,891
DN3086_c0_g1	PV21_POMCA Perivitellin-2 67 kDa subunit OS=Pomacea canaliculata OX=400727 PE=1 SV=2	P0C8G6	38,891
DN27081_c0_g1	CALGL_BOTIN Calglandulin OS=Bothrops insularis OX=8723 PE=2 SV=1	Q8AY75	38,5058
DN34648_c0_g1	SLB_CROHD Snaclec CHH-B subunit beta OS=Crotalus horridus OX=35024 PE=1 SV=1	P81509	38,5058
DN1850_c0_g1	STXA_SYNVE Neoverrucotoxin subunit alpha OS=Synanceia verrucosa OX=51996 PE=1 SV=1	A0ZSK3	38,5058
DN17444_c0_g1	FCNV1_VARKO Veficolin-1 (Fragment) OS=Varanus komodoensis OX=61221 PE=2 SV=1	E2IYB3	38,1206
DN59154_c0_g1	FCNV1_VARKO Veficolin-1 (Fragment) OS=Varanus komodoensis OX=61221 PE=2 SV=1	E2IYB3	38,1206
DN45300_c0_g1	FCNV1_VARKO Veficolin-1 (Fragment) OS=Varanus komodoensis OX=61221 PE=2 SV=1	E2IYB3	38,1206
DN433_c0_g2	FCNV3_CERRY Ryncolin-3 OS=Cerberus rynchops OX=46267 PE=1 SV=1	D8VNS9	38,1206
DN31121_c0_g1	PA2H2_BOTAS Basic phospholipase A2 homolog 2 OS=Bothrops asper OX=8722 PE=1 SV=3	P24605	38,1206
DN31121_c0_g1	PA2H2_BOTAS Basic phospholipase A2 homolog 2 OS=Bothrops asper OX=8722 PE=1 SV=3	P24605	38,1206
DN32650_c0_g1	CALGL_BOTIN Calglandulin OS=Bothrops insularis OX=8723 PE=2 SV=1	Q8AY75	37,7354
DN29060_c0_g1	CALGL_PSEPO Calglandulin OS=Pseudechis porphyriacus OX=8671 PE=2 SV=1	Q3SB08	37,7354
DN29060_c0_g1	CALGL_TROCA Calglandulin OS=Tropidechis carinatus OX=100989 PE=2 SV=1	Q3SB11	37,7354
DN15802_c0_g1	FAXD2_DEMVE Venom prothrombin activator vestarin-D2 OS=Demansia vestigiata OX=412038 PE=1 SV=1	A6MFK8	37,7354
DN60844_c0_g1	IGFBP_CUPSA Insulin-like growth factor-binding protein-related protein 1 OS=Cupiennius salei OX=6928 PE=2 SV=1	G4V4G1	37,7354
DN36949_c0_g1	LECG_THANI Galactose-specific lectin nattectin OS=Thalassophryne nattereri OX=289382 PE=1 SV=1	Q66S03	37,7354
DN34126_c1_g5	LIPE_CROAD Putative endothelial lipase OS=Crotalus adamanteus OX=8729 PE=2 SV=1	J3RZ81	37,7354
DN32986_c0_g1	LIPE_CROAD Putative endothelial lipase OS=Crotalus adamanteus OX=8729 PE=2 SV=1	J3RZ81	37,7354
DN31121_c0_g1	PA2H2_BOTAS Basic phospholipase A2 homolog 2 OS=Bothrops asper OX=8722 PE=1 SV=3	P24605	37,7354
DN1850_c0_g1	STXA_SYNVE Neoverrucotoxin subunit alpha OS=Synanceia verrucosa OX=51996 PE=1 SV=1	A0ZSK3	37,7354
DN71109_c0_g1	TU91_IOTCI Turripeptide Ici9.1 (Fragment) OS=Iotyrris cingulifera OX=553733 PE=2 SV=1	P0DKM8	37,7354
DN51329_c0_g1	VCO32_AUSSU A.superbus venom factor 2 OS=Austrelaps superbus OX=29156 PE=2 SV=1	A0RZC6	37,7354
DN48370_c0_g1	ACPH1_APIME Venom acid phosphatase Acph-1 OS=Apis mellifera OX=7460 PE=1 SV=1	Q5BLY5	37,3502
DN57153_c0_g1	FCNV1_VARKO Veficolin-1 (Fragment) OS=Varanus komodoensis OX=61221 PE=2 SV=1	E2IYB3	37,3502
DN4605_c0_g1	FCNV2_CERRY Ryncolin-2 OS=Cerberus rynchops OX=46267 PE=1 SV=1	D8VNS8	37,3502
DN68985_c0_g1	IGFBP_CUPSA Insulin-like growth factor-binding protein-related protein 1 OS=Cupiennius salei OX=6928 PE=2 SV=1	G4V4G1	37,3502
DN72015_c0_g1	IGFBP_CUPSA Insulin-like growth factor-binding protein-related protein 1 OS=Cupiennius salei OX=6928 PE=2 SV=1	G4V4G1	37,3502
DN32633_c1_g1	OXLA_BUNMU L-amino-acid oxidase OS=Bungarus multicinctus OX=8616 PE=2 SV=1	A8QL51	37,3502
DN28300_c0_g1	PA2_ANUPH Phospholipase A2 phaiodactylipin OS=Anuroctonus phaiodactylus OX=246982 PE=1 SV=2	Q6PXP0	37,3502
DN65171_c0_g1	A1LC_LOXAR Phospholipase D LarSicTox-alphaIB2c (Fragment) OS=Loxosceles arizonica OX=196454 PE=2 SV=1	C0JAU6	36,965
DN26947_c0_g1	CALGL_BOTIN Calglandulin OS=Bothrops insularis OX=8723 PE=2 SV=1	Q8AY75	36,965
DN60576_c0_g1	FAXD1_DEMVE Venom prothrombin activator vestarin-D1 OS=Demansia vestigiata OX=412038 PE=1 SV=1	A6MFK7	36,965
DN73547_c0_g1	FCNV1_VARKO Veficolin-1 (Fragment) OS=Varanus komodoensis OX=61221 PE=2 SV=1	E2IYB3	36,965
DN48792_c0_g1	VM2T3_PROMU Zinc metalloproteinase/disintegrin OS=Protobothrops mucrosquamatus OX=103944 PE=1 SV=1	O57413	36,965
DN61519_c0_g1	ALL6_APIME Allergen Api m 6 OS=Apis mellifera OX=7460 PE=1 SV=1	P83563	36,5798
DN40868_c0_g1	B1H1_LOXIN Phospholipase D LiSicTox-betaIA1i OS=Loxosceles intermedia OX=58218 PE=2 SV=1	Q2XQ09	36,5798
DN23632_c0_g1	CALGL_PSEPO Calglandulin OS=Pseudechis porphyriacus OX=8671 PE=2 SV=1	Q3SB08	36,5798
DN33898_c1_g5	CALGL_TROCA Calglandulin OS=Tropidechis carinatus OX=100989 PE=2 SV=1	Q3SB11	36,5798
DN15515_c0_g1	FAXC_PSETE Venom prothrombin activator pseutarin-C catalytic subunit OS=Pseudonaja textilis OX=8673 PE=1 SV=2	Q56VR3	36,5798
DN62654_c0_g1	FCNV1_VARKO Veficolin-1 (Fragment) OS=Varanus komodoensis OX=61221 PE=2 SV=1	E2IYB3	36,5798
DN54740_c0_g1	FCNV1_VARKO Veficolin-1 (Fragment) OS=Varanus komodoensis OX=61221 PE=2 SV=1	E2IYB3	36,5798
DN20014_c0_g1	LATA_LATTR Alpha-latrotoxin-Lt1a OS=Latrodectus tredecimguttatus OX=6925 PE=1 SV=2	P23631	36,5798
DN31085_c0_g1	LECM2_PSEPL C-type lectin lectoxin-Enh2 OS=Pseudoferania polylepis OX=338839 PE=2 SV=1	A7X3W6	36,5798
DN32785_c0_g1	NGFV1_TROCA Venom nerve growth factor 1 OS=Tropidechis carinatus OX=100989 PE=2 SV=1	Q3HXX8	36,5798
DN35066_c0_g1	TO1D_HADMO Omega-hexatoxin-Hmo1d OS=Hadronyche modesta OX=1337084 PE=3 SV=1	P0DMQ3	36,5798
DN34936_c0_g1	VCP_APIME Venom serine carboxypeptidase OS=Apis mellifera OX=7460 PE=2 SV=1	C9WMM5	36,5798
DN32271_c0_g1	VEGFA_BITGA Vascular endothelial growth factor A OS=Bitis gabonica OX=8694 PE=1 SV=1	P83906	36,5798
DN28831_c0_g1	3NO21_BUNCA Weak toxin 1 OS=Bungarus candidus OX=92438 PE=1 SV=1	Q8AY51	36,1946
DN40595_c0_g1	ACES_TRILK Acetylcholinesterase-1 OS=Trittame loki OX=1295018 PE=1 SV=1	W4VSJ0	36,1946
DN23632_c0_g1	CALGL_PSEPOCalglandulin OS=Pseudechis porphyriacus OX=8671 PE=2 SV=1	Q3SB08	36,1946
DN56147_c0_g1	CVP6_PIMHY Cysteine-rich venom protein 6 OS=Pimpla hypochondriaca OX=135724 PE=1 SV=1	Q8T0W0	36,1946
DN40439_c0_g1	LCTA_LATTR Alpha-latrocrustotoxin-Lt1a (Fragment) OS=Latrodectus tredecimguttatus OX=6925 PE=2 SV=2	Q9XZC0	36,1946
DN26730_c0_g1	OXLA_OPHHA L-amino-acid oxidase OS=Ophiophagus hannah OX=8665 PE=1 SV=3	P81383	36,1946
DN4715_c0_g1	CT0C_CONAE Conotoxin ArMLCL-022 OS=Conus arenatus OX=89451 PE=2 SV=1	Q9BPD2	35,8094
DN64019_c0_g1	FAXD2_DEMVEVenom prothrombin activator vestarin-D2 OS=Demansia vestigiata OX=412038 PE=1 SV=1	A6MFK8	35,8094
DN52333_c0_g1	FCNV1_VARKO Veficolin-1 (Fragment) OS=Varanus komodoensis OX=61221 PE=2 SV=1	E2IYB3	35,8094
DN14038_c0_g1	FCNV1_VARKO Veficolin-1 (Fragment) OS=Varanus komodoensis OX=61221 PE=2 SV=1	E2IYB3	35,8094
DN57657_c0_g1	FCNV1_VARKO Veficolin-1 (Fragment) OS=Varanus komodoensis OX=61221 PE=2 SV=1	E2IYB3	35,8094
DN55027_c0_g1	GIG1_STIGIO MEGA-stichotoxin-Sgt1a OS=Stichodactyla gigantea OX=230562 PE=1 SV=1	Q76CA1	35,8094
DN21352_c0_g1	PDE1_CROAD Venom phosphodiesterase 1 OS=Crotalus adamanteus OX=8729 PE=1 SV=2	J3SEZ3	35,8094
DN34096_c1_g1	SL1A_BOTJA Snaclec GPIB-binding protein subunit alpha OS=Bothrops jararaca OX=8724 PE=1 SV=1	Q9PSM6	35,8094

Sequence Name	Sequence Description	Blast Top Hit Accession	Blast Top Hit Score
**DN14593_c0_g1**	TX150_BUNCI Toxin Bcs III 15.09 (Fragment) OS=Bunodosoma caissarum OX=31165 PE=1 SV=1	P86468	35,8094
**DN67138_c0_g1**	VM2TA_TRIGA Zinc metalloproteinase/disintegrin OS=Trimeresurus gramineus OX=8767 PE=1 SV=3	P15503	35,8094
**DN31058_c0_g1**	ACR2_ACTE QU-actitoxin-Aeq6a OS=Actinia equina OX=6106 PE=1 SV=1	Q3C256	35,4242
**DN31058_c0_g1**	ACR2_ACTE QU-actitoxin-Aeq6a OS=Actinia equina OX=6106 PE=1 SV=1	Q3C256	35,4242
**DN31058_c0_g1**	ACR2A_ACTE QU-actitoxin-Aeq6b OS=Actinia equina OX=6106 PE=1 SV=1	Q3C255	35,4242
**DN31058_c0_g1**	ACR2A_ACTE QU-actitoxin-Aeq6b OS=Actinia equina OX=6106 PE=1 SV=1	Q3C255	35,4242
**DN32642_c0_g1**	CA14_CONPL Alpha-conotoxin-like Pu1.4 OS=Conus pulicarius OX=93154 PE=3 SV=1	P0C8U8	35,4242
**DN1966_c0_g1**	CVP6_PIMHY Cysteine-rich venom protein 6 OS=Pimpla hypochondriaca OX=135724 PE=1 SV=1	Q8T0W0	35,4242
**DN64399_c0_g1**	IGFBP_CUPSA Insulin-like growth factor-binding protein-related protein 1 OS=Cupiennius salei OX=6928 PE=2 SV=1	G4V4G1	35,4242
**DN39200_c0_g1**	LATA_LATHE Alpha-latrotoxin-Lhe1a OS=Latrodectus hesperus OX=256737 PE=1 SV=2	P0DJE3	35,4242
**DN31704_c0_g1**	NF3_CONMR Conotoxin Mr15.3 OS=Conus marmoreus OX=42752 PE=1 SV=1	P0DM20	35,4242
**DN23217_c0_g1**	TX150_BUNCI Toxin Bcs III 15.09 (Fragment) OS=Bunodosoma caissarum OX=31165 PE=1 SV=1	P86468	35,4242
**DN66943_c0_g1**	VM2H1_BOTLA Zinc metalloproteinase-disintegrin BlatH1 OS=Bothriechis lateralis OX=44727 PE=1 SV=1	U5PZ28	35,4242
**DN4270_c0_g1**	FAXC_PSETE Venom prothrombin activator pseutarin-C catalytic subunit OS=Pseudonaja textilis OX=8673 PE=1 SV=2	Q56VR3	35,039
**DN35978_c0_g1**	FCNV1_VARKO Veficolin-1 (Fragment) OS=Varanus komodoensis OX=61221 PE=2 SV=1	E2IYB3	35,039
**DN23304_c0_g1**	I3BB_CONCB Conotoxin Ca11b OS=Conus caracteristicus OX=89440 PE=1 SV=1	D2DGD4	35,039
**DN33674_c1_g1**	LIPE_CROAD Putative endothelial lipase OS=Crotalus adamanteus OX=8729 PE=2 SV=1	J3RZ81	35,039
**DN23217_c0_g1**	TX150_BUNCI Toxin Bcs III 15.09 (Fragment) OS=Bunodosoma caissarum OX=31165 PE=1 SV=1	P86468	35,039
**DN3888_c0_g1**	TX150_BUNCI Toxin Bcs III 15.09 (Fragment) OS=Bunodosoma caissarum OX=31165 PE=1 SV=1	P86468	35,039
**DN68043_c0_g1**	VDDP4_VESVU Venom dipeptidyl peptidase 4 OS=Vespula vulgaris OX=7454 PE=1 SV=1	B1A4F7	35,039
**DN45071_c0_g1**	FCNV1_VARKO Veficolin-1 (Fragment) OS=Varanus komodoensis OX=61221 PE=2 SV=1	E2IYB3	34,6538
**DN35254_c0_g1**	LCTA_LATTR Alpha-latrocrustotoxin-Lt1a (Fragment) OS=Latrodectus tredecimguttatus OX=6925 PE=2 SV=2	Q9XZC0	34,6538
**DN17294_c0_g1**	SL7_ECHPL Snaclec 7 OS=Echis pyramidum leakeyi OX=38415 PE=2 SV=1	Q6X5S0	34,6538
**DN67041_c0_g1**	STXB_SYNHO Stonustoxin subunit beta OS=Synanceia horrida OX=13279 PE=1 SV=3	Q91453	34,6538
**DN39613_c0_g1**	VM3_BUNMU Zinc metalloproteinase-disintegrin-like BmMP OS=Bungarus multicinctus OX=8616 PE=1 SV=1	A8QL49	34,6538
**DN28598_c0_g1**	VM3_CERRY Zinc metalloproteinase-disintegrin-like OS=Cerberus rynchops OX=46267 PE=1 SV=1	D8VNS0	34,6538
**DN52355_c0_g1**	CTX_SEPES SE-cephalotoxin OS=Sepia esculenta OX=31210 PE=1 SV=1	B2DCR8	34,2686
**DN14212_c0_g1**	CTX2_CHIFL Toxin CfTX-2 OS=Chironex fleckeri OX=45396 PE=1 SV=1	A7L036	34,2686
**DN4821_c0_g1**	FAXD_HOPST Venom prothrombin activator hopsarin-D OS=Hoplocephalus stephensii OX=196418 PE=1 SV=2	P83370	34,2686
**DN33563_c9_g2**	LIPE_CROAD Putative endothelial lipase OS=Crotalus adamanteus OX=8729 PE=2 SV=1	J3RZ81	34,2686
**DN33679_c0_g1**	LITA_LATTR Alpha-latroinsectotoxin-Lt1a (Fragment) OS=Latrodectus tredecimguttatus OX=6925 PE=1 SV=1	Q02989	34,2686
**DN5238_c0_g1**	O1611_CONVE Conotoxin VnMKLT1-01121 OS=Conus ventricosus OX=117992 PE=2 SV=1	Q9BPA2	34,2686
**DN33900_c1_g2**	3NX1_ERYPO Probable weak neurotoxin 3FTx-Lio1 OS=Erythrolamprus poecilogyrus OX=338838 PE=3 SV=1	A7X3M9	33,8834
**DN74150_c0_g1**	B31_LOXSN Phospholipase D LspiSicTox-betaIII1 (Fragment) OS=Loxosceles spinulosa OX=571532 PE=2 SV=1	C0JB92	33,8834
**DN66071_c0_g1**	FAXD1_DEMVE Venom prothrombin activator vestarin-D1 OS=Demansia vestigiata OX=412038 PE=1 SV=1	A6MFK7	33,8834
**DN9176_c0_g1**	FCNV3_CERRY Ryncolin-3 OS=Cerberus rynchops OX=46267 PE=1 SV=1	D8VNS9	33,8834
**DN33468_c4_g1**	LIPE_CROAD Putative endothelial lipase OS=Crotalus adamanteus OX=8729 PE=2 SV=1	J3RZ81	33,8834
**DN23217_c0_g1**	TX150_BUNCI Toxin Bcs III 15.09 (Fragment) OS=Bunodosoma caissarum OX=31165 PE=1 SV=1	P86468	33,8834
**DN72083_c0_g1**	FCNV1_VARKO Veficolin-1 (Fragment) OS=Varanus komodoensis OX=61221 PE=2 SV=1	E2IYB3	33,4982
**DN60823_c0_g1**	FCNV3_CERRY Ryncolin-3 OS=Cerberus rynchops OX=46267 PE=1 SV=1	D8VNS9	33,4982
**DN18810_c0_g1**	KBX22_LYCMC Neurotoxin beta-KTx 12 OS=Lychas mucronatus OX=172552 PE=2 SV=1	D9U2B1	33,4982
**DN23217_c0_g1**	TX150_BUNCI Toxin Bcs III 15.09 (Fragment) OS=Bunodosoma caissarum OX=31165 PE=1 SV=1	P86468	33,4982
**DN32453_c0_g1**	VM2L2_MACLB Zinc metalloproteinase/disintegrin OS=Macrovipera lebetina OX=8709 PE=1 SV=1	Q98995	33,4982
**DN14949_c0_g1**	CALGL_BOTIN Calglandulin OS=Bothrops insularis OX=8723 PE=2 SV=1	Q8AY75	33,113
**DN26386_c0_g1**	MASOD_ORADR Eumenine mastoparan-OD OS=Orancistrocerus drewseni OX=529024 GN=VP1 PE=1 SV=2	P86146	33,113
**DN31121_c0_g1**	PA2A_BOTJA Acidic phospholipase A2 OS=Bothrops jararaca OX=8724 PE=1 SV=1	P81243	33,113
**DN31121_c0_g1**	PA2A_BOTJA Acidic phospholipase A2 OS=Bothrops jararaca OX=8724 PE=1 SV=1	P81243	33,113
**DN56103_c0_g1**	TAG2Z_AGEOR U2-agatoxin-Ao1z OS=Agelena orientalis OX=293813 PE=2 SV=1	Q5Y4W2	33,113
**DN47735_c0_g1**	GIG1_STIGIO MEGA-stichotoxin-Sgt1a OS=Stichodactyla gigantea OX=230562 PE=1 SV=1	Q76CA1	32,7278
**DN31121_c0_g1**	PA2A_BOTJAAcidic phospholipase A2 OS=Bothrops jararaca OX=8724 PE=1 SV=1	P81243	32,7278
**DN31121_c0_g1**	PA2A_BOTJA Acidic phospholipase A2 OS=Bothrops jararaca OX=8724 PE=1 SV=1	P81243	32,7278
**DN31121_c0_g1**	PA2A_BOTJA Acidic phospholipase A2 OS=Bothrops jararaca OX=8724 PE=1 SV=1	P81243	32,7278
**DN29723_c0_g1**	3SOIK_BUNFA Neurotoxin 3FTx-LK OS=Bungarus fasciatus OX=8613 PE=1 SV=1	P0C552	32,3426
