# Supplementary material for: In-depth transcriptome reveals the potential biotechnological application of Bothrops jararaca venom gland
Source: J Venom Anim Toxins Incl Trop Dis. 2020 Oct 21;26:e20190058. doi: 10.1590/1678-9199-JVATITD-2019-0058 (PMC7579844; doi:10.1590/1678-9199-JVATITD-2019-0058)
Supplement: Additional file 13. [file 1678-9199-jvatitd-26-e20190058-s13.pdf]

J Venom Anim Toxins incl Trop Dis

https://doi.org/10.1590/1678-9199-JVATITD-2019-0058

Supplementary Material to "In-depth transcriptome reveals the potential biotechnological application of *Bothrops jararaca* venom gland"

Additional file 13. Animal toxins encoded in the *Bothrops jararaca* transcriptome, identified in both Animal Toxin Annotation Project and NCBI non-redundant (NR) database.

Sequence name	Sequence desc.	Hit ACC	E-Value
**DN33641_c0_g1**	Ectonucleotide pyrophosphatase/phosphodiesterase family member 4	ETE60172	0
**DN18627_c0_g1**	ectonucleotide pyrophosphatase/phosphodiesterase family member 2 isoform X1	AEJ31981	0
**DN36408_c0_g1**	ectonucleotide pyrophosphatase/phosphodiesterase family member 2 isoform X2	XP_013929717	0
**DN32044_c0_g1**	ectonucleotide pyrophosphatase/phosphodiesterase family member 3	J3SEZ3, AEJ31980	0.00E+00
**DN29632_c0_g1**	ectonucleotide pyrophosphatase/phosphodiesterase family member 6	XP_013917463	0
**DN33935_c2_g1**	5'-nucleotidase domain-containing protein 2	XP_007432442	0
**DN25704_c0_g1**	5'-nucleotidase domain-containing protein 2-like	XP_008102124	0
**DN29580_c0_g1**	5'-nucleotidase isoform X1	BAP39925	0
**DN11038_c0_g1**	cytosolic purine 5'-nucleotidase	ABK63558	0
**DN24760_c0_g1**	Cytosolic 5'-nucleotidase 1B	ETE70174	0
**DN31509_c0_g1**	5'-nucleotidase domain-containing protein 1	XP_013908865	0
**DN31791_c0_g1**	Soluble calcium-activated nucleotidase 1	ETE65318	0
**DN24778_c0_g1**	phospholipase A1 member A	XP_013922948	0
**DN25276_c0_g1**	phospholipase A-2-activating protein	XP_013910549	0
**DN29385_c0_g1**	cytosolic phospholipase A2 gamma-like	XP_007442450	0
**DN23375_c0_g1**	cytosolic phospholipase A2	XP_013922893	0
**DN29094_c0_g1**	calcium-independent phospholipase A2-gamma isoform X1	XP_013921974	0
**DN33608_c0_g1**	patatin-like phospholipase domain-containing protein 7	XP_007420656	0
**DN31772_c0_g1**	Phospholipase B-like 1	F8S101, AEJ31983	0
**DN28929_c0_g1**	putative phospholipase B-like 2	XP_007436772	0
**DN9413_c0_g1**	N-acyl-phosphatidylethanolamine-hydrolyzing phospholipase D	XP_013932002	0
**DN33180_c0_g1**	phosphatidylinositol-glycan-specific phospholipase D	XP_007422003	0
**DN22201_c0_g1**	phospholipase D2	XP_013931058, XP_013931065, XP_013931072	0
**DN31240_c0_g1**	phospholipase D2	XP_013931058, XP_013931065, XP_013931072	0
**DN25048_c0_g1**	phospholipase D3	XP_007433844, XP_007433845	0
**DN20509_c0_g1**	phospholipase DDHD1	XP_013921948	0
**DN33930_c0_g1**	amine oxidase [flavin-containing] A	XP_008105637	0
**DN32234_c0_g1**	L-amino acid oxidase	B5AR80, ACG55578	0
**DN31322_c0_g1**	L-amino acid oxidase	B5AR80, ACG55578	0
**DN28080_c0_g1**	A disintegrin and metalloproteinase with thrombospondin motifs 1	ETE67283	0
**DN32602_c0_g1**	Disintegrin and metalloproteinase domain-containing protein 10	XP_013914208	0
**DN28218_c0_g1**	disintegrin and metalloproteinase domain-containing protein 17	XP_007439283	0
**DN34088_c1_g1**	metalloproteinase precursor	Q5XUW8, AAU47334	0
**DN34088_c1_g1**	metalloproteinase VMP-III precursor	O93523, AAC61986	0
**DN34049_c16_g1**	metalloproteinase VMP-III precursor	Q8QG88, AAM09693	0
**DN34028_c0_g1**	complement C3-like	J3S836	0
**DN34028_c0_g1**	complement C3-like	J3S836	0
**DN34028_c0_g1**	complement C3-like	J3S836	0
**DN34028_c0_g1**	complement C3-like	J3S836	0
**DN33061_c11_g1**	venom dipeptidylpeptidase IV	BAP39988	0
**DN33061_c11_g1**	venom dipeptidylpeptidase IV	BAP39988	0
**DN32861_c0_g1**	glutaminyl cyclase	Q9YIB5, BAA34290	0
**DN29659_c0_g1**	hyaluronidase	BAN89379	0
**DN2883_c0_g1**	lysophosphatidic acid phosphatase type 6	XP_013932094	0
**DN30398_c0_g1**	Lysosomal acid phosphatase	ETE72159	0
**DN27255_c0_g1**	acid phosphatase-like protein 2	XP_013919847	0
**DN32197_c1_g1**	iron/zinc purple acid phosphatase-like protein	XP_008120993	0
**DN33067_c0_g1**	serine protease 23	XP_013912593	0
**DN20749_c0_g1**	serine protease hepsin	XP_007432268, XP_007432269	0
**DN22875_c0_g1**	Cathepsin L1	ETE62885	0
**DN19230_c0_g1**	Reticulocalbin-1	XP_013921149	0
**DN32587_c0_g1**	Reticulocalbin-1	ETE73341	0
**DN34049_c16_g1**	metalloproteinase VMP-III precursor	P30431, CAA48323	0
**DN34049_c16_g1**	metalloproteinase VMP-III precursor	P30431, CAA48323	0
**DN34088_c1_g1**	Disintegrin bothrostatin; Short=D-BTT; Flags: Precursor	Q98SP2, AAK15542	0
**DN34088_c1_g1**	metalloproteinase VMP-III precursor	Q98SP2, AAK15542	0
**DN29499_c1_g1**	Insulin-like growth factor 2 mRNA-binding protein 2	XP_007420695	0
**DN34009_c1_g1**	relA-associated inhibitor	XP_013918408	0
**DN34009_c1_g1**	relA-associated inhibitor	XP_013918408	0
**DN34009_c1_g1**	relA-associated inhibitor	XP_013918408	0
**DN3215_c0_g1**	plasma protease C1 inhibitor	XP_013930568	0
**DN31756_c0_g1**	Ribonuclease inhibitor	XP_013916345	0
**DN31756_c0_g1**	Ribonuclease inhibitor	ETE73461	0
**DN32967_c0_g1**	kunitz-type protease inhibitor 1	XP_013916935	0
**DN30106_c0_g1**	Complement factor I	ETE65087	0
**DN30250_c0_g1**	complement C1r subcomponent	XP_013927599	0
**DN24671_c0_g1**	Complement component C7	XP_013920644	0
**DN23293_c0_g1**	3'(2'),5'-bisphosphate nucleotidase 1	ETE66665	2.58E-174
**DN47228_c0_g1**	Serine protease HTRA1	ETE61161	6.20E-174

Sequence name	Sequence desc.	Hit ACC	E-Value
**DN34139_c2_g1**	Cathepsin O	XP_007437935	1.00E-166
**DN31240_c0_g1**	phospholipase D2	XP_013931058, XP_013931065, XP_013931072	1.68E-166
**DN3729_c0_g1**	7-methylguanosine phosphate-specific 5'-nucleotidase	XP_013908174	1.5604E-165
**DN32587_c0_g1**	Reticulocalbin-1	ETE73341	9.29E-162
**DN33592_c1_g1**	glutaminyl-peptide cyclotransferase-like protein	XP_007440518	4.596E-160
**DN39558_c0_g1**	ectonucleotide pyrophosphatase/phosphodiesterase family member 1	XP_007430580	3.52E-159
**DN39558_c0_g1**	ectonucleotide pyrophosphatase/phosphodiesterase family member 1	XP_007430580	3.52E-159
**DN32541_c0_g1**	Phospholipase A2 inhibitor	O93233, BAA31994	5.2162E-159
**DN28767_c0_g1**	reticulocalbin-2	J3S9D9	5.41E-158
**DN33075_c1_g1**	group 3 secretory phospholipase A2	XP_007440602	2.4974E-155
**DN17497_c1_g1**	peroxiredoxin-4	XP_007436716	9.5003E-153
**DN32541_c0_g1**	Phospholipase A2 inhibitor	O93233, BAA31994	4.6252E-152
**DN54825_c0_g1**	85/88 kDa calcium-independent phospholipase A2	XP_013910428, XP_013910429	3.68E-147
**DN11376_c0_g1**	ficolin-2-like	XP_013909778	5.06E-144
**DN25704_c0_g1**	5'-nucleotidase domain-containing protein 2-like	XP_007420586	5.8314E-143
**DN32197_c1_g1**	Iron/zinc purple acid phosphatase-like protein	ETE61889	3.13E-140
**DN33554_8c_g1**	Vascular apoptosis-inducing protein-like; Short=VAP-like	C5H5D2, ACI02287	1.38E-139
**DN32197_c1_g1**	iron/zinc purple acid phosphatase-like protein	XP_008120993	3.37E-138
**DN33554_c8_g1**	Vascular apoptosis-inducing protein-like; Short=VAP-like	C5H5D2, ACI02287	1.379E-136
**DN49418_c0_g1**	phospholipase D3-like	XP_007442263	3.2729E-136
**DN28080_c0_g1**	A disintegrin and metalloproteinase with thrombospondin motifs 1	ETE67283	4.547E-136
**DN29385_c0_g1**	cytosolic phospholipase A2 gamma-like	XP_007442450	2.32E-135
**DN40332_c0_g1**	vascular endothelial growth factor C	XP_013912093	4.411E-135
**DN34088_c1_g2**	metalloproteinase VMP-III precursor	P30431, CAA48323	1.35E-134
**DN32197_c1_g1**	iron/zinc purple acid phosphatase-like protein	XP_008120993	1.22E-130
**DN62957_c0_g1**	Lysophospholipase-like protein 1	ETE66670	6.69E-130
**DN34088_c1_g2**	metalloproteinase VMP-III precursor	P30431, CAA48323	8.39E-127
**DN32351_c0_g1**	metalloproteinase inhibitor 2	XP_007431028	3.27E-126
**DN33554_c8_g1**	Vascular apoptosis-inducing protein-like; Short=VAP-like	C5H5D2, ACI02287	1.971E-125
**DN29230_c0_g1**	complement factor D	XP_013914195	6.5136E-125
**DN22046_c0_g1**	mannan-binding lectin serine protease 2	XP_013920883	6.88E-123
**DN33075_c1_g1**	group 3 secretory phospholipase A2	XP_007440602	1.0228E-121
**DN13298_c0_g1**	ADAMTS-like protein 2	XP_013928722	2.43E-121
**DN12386_c0_g1**	Metalloproteinase inhibitor 3	ETE71398	4.92E-121
**DN64527_c0_g1**	cholinesterase isoform X1	ETE63748	1.83E-119
**DN13198_c0_g1**	group XV phospholipase A2	XP_007438398	8.0608E-117
**DN26220_c0_g1**	tumor necrosis factor ligand superfamily member 10-like	XP_015687894.1	9.87E-117
**DN13735_c0_g1**	peroxiredoxin-6	XP_007425204	3.5468E-116
**DN32197_c1_g1**	iron/zinc purple acid phosphatase-like protein	XP_013928633	5.89E-116
**DN33053_c0_g1**	phospholipase A2 inhibitor-like	ETE56837	1.19E-113
**DN66608_c0_g1**	pancreatic alpha-amylase-like	XP_007424834	6.2551E-113
**DN33180_c0_g1**	phosphatidylinositol-glycan-specific phospholipase D	XP_007422003	2.77E-112
**DN30499_c0_g1**	kunitz-type protease inhibitor 2	B2BS84, ABW90603	2.5023E-111
**DN31772_c0_g1**	phospholipase b	BAN82156	6.1447E-111
**DN24675_c0_g1**	peroxiredoxin-1	XP_013919294	7.7562E-110
**DN34088_c1_g2**	metalloproteinase VMP-III precursor	P30431, CAA48323	8.24E-110
**DN66057_c0_g1**	ADAMTS-like protein 2	XP_003229626	8.30E-109
**DN33641_c0_g1**	Ectonucleotide pyrophosphatase/phosphodiesterase family member 4	ETE60172	1.27E-107
**DN33023_c0_g1**	vascular endothelial growth factor	BAP39940	5.0286E-107
**DN24249_c0_g1**	Cysteine-rich secretory protein LCCL domain-containing 2	ETE66074	1.1249E-104
**DN28300_c0_g1**	group XIIA secretory phospholipase A2	XP_007437449	3.2626E-104
**DN29873_c0_g1**	5'(3')-deoxyribonucleotidase, cytosolic type	XP_013926019	4.10E-104
**DN34049_c16_g1**	metalloproteinase VMP-III precursor	P30431, CAA48323	1.36E-103
**DN40072_c0_g1**	reversion-inducing cysteine-rich protein with Kazal motifs	XP_007438838	1.50E-100
**DN13298_c0_g1**	ADAMTS-like protein 2	XP_013928721	1.72E-100
**DN48293_c0_g1**	cytosolic 5'-nucleotidase 3A isoform X1	XP_003222308	2.86E-100
**DN14716_c0_g1**	peroxiredoxin-1 isoform X2	XP_013928980	4.1373E-100
**DN71172_c0_g1**	tumor necrosis factor receptor superfamily member 21	XP_026534872.1	1.76E-99
**DN33935_c2_g1**	5'-nucleotidase domain-containing protein 2	XP_007432442	8.14E-99
**DN39954_c0_g1**	Phospholipase D1	ETE64712	7.25643E-98
**DN39236_c0_g1**	insulin-like growth factor-binding protein 7	XP_013917918	1.11499E-97
**DN16294_c0_g1**	ADAMTS-like protein 4	XP_007443702	3.59E-97
**DN34216_c0_g1**	metalloproteinase inhibitor 1	XP_013928204	6.82E-97
**DN39723_c0_g1**	Neurotrophin-3	ETE60908	1.29022E-96
**DN54668_c0_g1**	Mannan-binding lectin serine protease 2	ETE69340	2.93E-96
**DN22046_c0_g1**	Mannan-binding lectin serine protease 2	ETE69340	3.33E-95
**DN63420_c0_g1**	Cytosolic phospholipase A2 zeta	XP_007428661	1.57E-93
**DN47901_c0_g1**	Cytosolic phospholipase A2 zeta	ETE66890	1.10E-92
**DN65784_c0_g1**	phospholipase DDHD2	XP_013920187	7.19719E-92
**DN16308_c0_g1**	patatin-like phospholipase domain-containing protein 7	XP_007420655	8.76E-92
**DN16437_c0_g1**	alpha-phospholipase A2 inhibitor precursor	B1A4N8, ABZ82329, ABZ82330, ABZ82331	1.55054E-91
**DN72338_c0_g1**	patatin-like phospholipase domain-containing protein 2	XP_013926144	5.15E-90
**DN74197_c0_g1**	phospholipase DDHD1	XP_013921948	1.05122E-88
**DN3083_c0_g1**	trehalase	XP_007424325	1.78588E-88
**DN71199_c0_g1**	cholinesterase	XP_013929768	1.82E-87
**DN34088_c1_g1**	metalloprotease P-IIa 1	BAP39948	5.51957E-86
**DN28182_c0_g1**	peroxiredoxin-5, mitochondrial	XP_013924410	3.94964E-85
**DN21889_c0_g1**	calglandulin-like protein	Q8AY75, AAN37910	3.30016E-84
**DN21415_c0_g1**	Patatin-like phospholipase domain-containing protein 2	ETE73136	9.04E-84
**DN33554_c8_g1**	MP_IIb1 SVMP precursor	ADO21506	2.1283E-83
**DN32310_c0_g1**	serine protease	Q8QG86, AAM09695	2.71577E-83

Sequence name	Sequence desc.	Hit ACC	E-Value
DN32310_c0_g1	serine protease	P81824	3.29209E-83
DN55601_c0_g1	pancreatic alpha-amylase	XP_007424834	9.06E-82
DN56775_c0_g1	cysteine-rich protein 2	XP_003219925	2.57E-81
DN32197_c1_g1	iron/zinc purple acid phosphatase-like protein-like	XP_007445495	5.95E-81
DN33023_c0_g1	vascular endothelial growth factor	BAD38846	6.72017E-80
DN13358_c0_g1	patatin-like phospholipase domain-containing protein 2	XP_007431121	5.05E-79
DN44012_c0_g1	translationally-controlled tumor protein homolog	XP_011641383	1.01527E-78
DN34152_c6_g1	serine protease	Q5W959, BAD66928	2.6537E-78
DN32310_c0_g1	serine protease	Q6IWF1, AAT40141	3.08127E-78
DN36603_c0_g1	cytosolic 5'-nucleotidase 3A isoform X1	XP_007431653	1.17E-77
DN34152_c6_g1	serine protease	Q5W959, BAD66928	1.49096E-77
DN20156_c0_g1	Calcium-independent phospholipase A2-gamma	XP_013917729, XP_013917737	9.15E-77
DN67500_c0_g1	cytosolic 5'-nucleotidase 1A-like	XP_013923131	3.21E-76
DN9764_c0_g1	ectonucleotide pyrophosphatase/phosphodiesterase family member 2	XP_013929717	1.93E-75
DN9764_c0_g1	ectonucleotide pyrophosphatase/phosphodiesterase family member 2	XP_013929717	1.93E-75
DN40861_c0_g1	Golgi-associated plant pathogenesis-related protein 1	XP_013908306	1.9553E-74
DN40861_c0_g1	Golgi-associated plant pathogenesis-related protein 1	XP_013908306	1.9553E-74
DN34152_c6_g1	Snake venom serine protease HS114; Short=SVSP; Flags: Precursor	Q5W959, BAD66928	2.39076E-74
DN33930_c0_g1	amine oxidase [flavin-containing] B	XP_013923711	2.62658E-74
DN33554_c8_g1	Snake venom metalloproteinase 7; Short=SVMP; Flags: Precursor	F8S108, AEJ31990	8.40727E-74
DN32310_c0_g1	serine proteinase isoform 5	ABG26971	4.13709E-72
DN4528_c0_g1	Coagulation factor VIII	ETE61422	8.72972E-72
DN33311_c1_g1	phospholipase A2	AFJ79208	2.93796E-71
DN25276_c0_g1	phospholipase A-2-activating protein	XP_013910549	3.67554E-71
DN33023_c0_g1	vascular endothelial growth factor	BAP39940	5.42723E-71
DN39396_c0_g1	Phospholipase DDHD2	ETE67929	1.05004E-70
DN31371_c0_g1	phospholipase A2	AFJ79208	1.82415E-70
DN75690_c0_g1	Calcium-independent phospholipase A2-gamma	XP_013917729, XP_013917737	2.28E-70
DN18379_c0_g1	patatin-like phospholipase domain-containing protein 2	XP_013913162	8.39E-70
DN55258_c0_g1	insulin-like growth factor-binding protein-like 1	XP_007421622	1.64971E-69
DN35176_c0_g1	trehalase	XP_013911319	2.69283E-68
DN67117_c0_g1	cholinesterase isoform X1	BAN89409	6.37E-68
DN33311_c1_g1	phospholipase A2	AFJ79208	9.69627E-68
DN34152_c6_g1	serine protease	Q5W959, BAD66928	1.09E-67
DN33554_c7_g1	metalloprotease BOJUMET II	AAP78951	2.55469E-67
DN40740_c0_g1	reversion-inducing cysteine-rich protein with Kazal motifs	XP_013908307	3.66E-67
DN31371_c0_g1	phospholipase A2	AFJ79208	4.68717E-67
DN8954_c0_g1	ADAMTS-like protein 4	XP_005293859	8.95E-67
DN34049_c13_g1	P-III metalloprotease	ADO21506	1.35275E-66
DN33311_c1_g1	phospholipase A2	AFJ79208	2.07978E-66
DN33219_c0_g1	c-type lectin	AAM22789	3.37816E-66
DN34152_c5_g1	serine protease	Q5W959, BAD66928	3.15801E-65
DN6844_c0_g1	85/88 kDa calcium-independent phospholipase A2	ETE71158	7.78E-65
DN34152_c5_g1	serine protease	Q5W959, BAD66928	8.78021E-65
DN40805_c0_g1	5'(3')-deoxyribonucleotidase, mitochondrial	XP_007433712	1.26E-64
DN8935_c0_g1	trehalase isoform X1	XP_013911319	2.4806E-64
DN33311_c1_g1	phospholipase A2	Q8QG87, AAM09694	3.421E-64
DN33978_c2_g1	Tumor rejection antigen	ETE71763	7.27E-64
DN33311_c1_g1	phospholipase A2	Q8QG87, AAM09694	7.81437E-64
DN30935_c0_g1	protein phosphatase inhibitor 2	XP_013925190	1.80E-63
DN32061_c4_g1	c-type lectin	AAM22789	2.04648E-63
DN33554_c8_g1	MP_IIb1 SVMP precursor	ADO21506	3.92389E-63
DN33219_c0_g1	c-type lectin	AAM22789	4.04605E-63
DN28578_c0_g1	BPTI/Kunitz domain-containing protein-like	XP_007443753	1.7083E-62
DN5074_c0_g1	tumor necrosis factor ligand superfamily member 10	XP_015670419.1	6.72E-62
DN63049_c0_g1	phospholipase A2 inhibitor and Ly6/PLAUR domain-containing protein	XP_013920904	8.12638E-61
DN33311_c1_g1	phospholipase A2	Q8QG87, AAM09694	1.05614E-60
DN32197_c1_g1	iron/zinc purple acid phosphatase-like protein-like	XP_007445495	1.39E-60
DN52466_c0_g1	ectonucleotide pyrophosphatase/phosphodiesterase family member 1	XP_007430580	8.30E-57
DN52466_c0_g1	ectonucleotide pyrophosphatase/phosphodiesterase family member 1	XP_007430580	8.30E-57
DN29873_c0_g1	5'(3')-deoxyribonucleotidase, cytosolic type	XP_013926019	1.49E-56
DN30568_c0_g1	phospholipase A2 inhibitor subunit gamma B-like	XP_007436184, XP_007436185	2.25E-55
DN30568_c0_g1	phospholipase A2 inhibitor subunit gamma B-like	XP_007436184, XP_007436185	2.38719E-55
DN73461_c0_g1	ADAMTS-like protein 4	XP_013910940	2.47E-55
DN30385_c1_g1	metalloprotease P-III 5	BAK64383	3.62528E-55
DN41645_c0_g1	Coagulation factor V	XP_007429194	2.44531E-54
DN33852_c0_g1	phosphatidylinositol-glycan-specific phospholipase D	XP_002937970	2.64E-54
DN20299_c0_g1	WAP four-disulfide core domain protein 2	ETE59165	3.96954E-54
DN71373_c0_g1	reversion-inducing cysteine-rich protein with Kazal motifs	XP_013908307	1.16E-53
DN23718_c0_g1	phospholipase DDHD1 isoform X2	XP_007424172	1.22277E-53
DN63914_c0_g1	ADAMTS-like protein 2	ETE63039	1.29E-53
DN70049_c0_g1	patatin-like phospholipase domain-containing protein 2	XP_003214822	3.15E-53
DN76849_c0_g1	thymus-specific serine protease-like	XP_013928370	6.68E-53
DN33075_c1_g1	group 3 secretory phospholipase A2	XP_007440602	6.82629E-52
DN21627_c0_g1	phospholipase A2-like	XP_011641088	1.15734E-51
DN24249_c0_g1	Cysteine-rich secretory protein LCCL domain-containing 2	ETE66074	1.34E-51
DN31664_c0_g1	kunitz-type protease inhibitor 4	XP_007442544	3.56303E-51
DN34088_c1_g1	metalloprotease P-IIa 1	BAP39948	4.97417E-51
DN39288_c0_g1	cytosolic phospholipase A2 zeta	XP_013920477	6.12E-51
DN39288_c0_g1	cytosolic phospholipase A2 zeta	XP_013920477	6.12E-51
DN33937_c0_g1	Tumor rejection antigen	ETE71763	2.21E-50

Sequence name	Sequence desc.	Hit ACC	E-Value
DN25445_c0_g1	Peroxiredoxin 3	ETE70551	2.67484E-50
DN34088_c1_g1	metalloprotease P-IIa 1	BAP39948	3.30512E-50
DN21921_c0_g2	veficolin-1-like isoform X1	XP_007443670	7.60E-50
DN32310_c0_g1	serine protease	P81824	9.71766E-50
DN32310_c0_g1	serine protease	P81824	9.72E-50
DN74057_c0_g1	phospholipase D1 isoform X1	XP_007421638	9.7338E-50
DN34088_c1_g1	metalloprotease P-IIa 1	BAP39948	1.11202E-49
DN73846_c0_g1	cytosolic 5'-nucleotidase 1A-like	ETE58393	2.42E-49
DN33112_c0_g1	Tumor rejection antigen	XP_013922044	2.42E-49
DN48543_c0_g1	phospholipase D1 isoform X2	XP_007421638	1.44245E-48
DN33978_c2_g1	Tumor rejection antigen	ETE71763	3.92E-46
DN31728_c0_g1	Tumor rejection antigen	ETE71763	3.40E-45
DN58758_c0_g1	cholinesterase-like	ETE70564	2.99E-43
DN29873_c0_g1	5'(3')-deoxyribonucleotidase, cytosolic type	XP_013926019	3.00E-43
DN71009_c0_g1	ATPase inhibitor, mitochondrial	XP_007434913	1.04E-42
DN31728_c0_g1	Tumor rejection antigen	ETE71763	6.37E-42
DN33978_c2_g1	Tumor rejection antigen	ETE71763	1.20E-41
DN56183_c0_g1	stonustoxin subunit alpha-like	ETE58515	1.39199E-41
DN29385_c0_g1	cytosolic phospholipase A2 gamma-like	XP_007442450	5.49E-41
DN68442_c0_g1	Phospholipase D3	ETE57698	1.35041E-40
DN32380_c0_g1	Tumor rejection antigen	ETE71763	1.92E-40
DN33311_c1_g1	phospholipase A2	G3DT18, ADQ08654	7.77679E-39
DN33440_c1_g3	Tumor rejection antigen	XP_013913595	1.42E-37
DN34177_c4_g1	Tumor rejection antigen	ETE71763	1.47E-37
DN35680_c0_g1	Cysteine-rich protein 1	ETE59024	1.50E-37
DN33937_c0_g1	Tumor rejection antigen	ETE71763	1.54E-37
DN33311_c1_g1	phospholipase A2	Q8QG87, AAM09694	1.97875E-37
DN72956_c0_g1	Tumor rejection antigen	ETE71763	6.84E-37
DN34041_c3_g2	Tumor rejection antigen	ETE71763	9.37E-37
DN3943_c0_g1	Tumor rejection antigen	AAL49761	1.35E-35
DN25382_c0_g1	angiotensin converting enzyme inhibitor and C-type natriuretic peptide precursor	AAM09691	3.08958E-34
DN33087_c0_g1	Tumor rejection antigen	XP_013913595	4.43E-33
DN33087_c0_g1	Tumor rejection antigen	ETE71763	1.82E-32
DN34110_c1_g2	Tumor rejection antigen	XP_013913595	2.27E-32
DN11082_c0_g1	Tumor rejection antigen	AAL49761	2.60E-32
DN23500_c1_g1	Tumor rejection antigen	ETE71763	2.09E-30
DN32802_c0_g1	Tumor rejection antigen	XP_013927219	7.92E-30
DN68985_c0_g1	insulin-like growth factor-binding protein 7	XP_013917918	4.46842E-29
DN32802_c0_g1	Tumor rejection antigen	AAL49761	1.84E-28
DN22101_c0_g1	Tumor rejection antigen	ETE71763	1.68E-27
DN32197_c1_g1	iron/zinc purple acid phosphatase-like protein	XP_008120993	9.52E-27
DN34041_c3_g2	Tumor rejection antigen	ETE71763	9.80E-27
DN28585_c0_g1	Tumor rejection antigen	ETE71763	1.11E-26
DN65376_c0_g1	5'(3')-deoxyribonucleotidase, mitochondrial	XP_013925150	2.09E-26
DN28585_c0_g1	Tumor rejection antigen	ETE71763	6.36E-26
DN33978_c2_g3	Tumor rejection antigen	ETE71763	1.23E-25
DN33646_c2_g1	Tumor rejection antigen	ETE71763	1.65E-25
DN24753_c0_g1	Tumor rejection antigen	XP_013929687	1.74E-25
DN32690_c0_g1	phospholipase A2 inhibitor subunit gamma B-like	ETE58602	4.88397E-25
DN32690_c0_g1	phospholipase A2 inhibitor subunit gamma B-like	ETE58602	5.04972E-25
DN33937_c0_g1	Tumor rejection antigen	ETE71763	1.07E-24
DN30651_c0_g1	phospholipase A2, membrane associated	XP_003431137	1.29524E-24
DN29075_c0_g1	Tumor rejection antigen	ETE71763	4.73E-24
DN30651_c0_g1	phospholipase A2, membrane associated	XP_003431137	7.20207E-23
DN16868_c0_g1	Tumor rejection antigen	XP_013913595	9.04E-23
DN34156_c1_g1	Tumor rejection antigen	ETE71763	1.27E-22
DN34156_c1_g1	Tumor rejection antigen	ETE71763	1.31E-22
DN34156_c1_g1	Tumor rejection antigen	ETE71763	2.34E-22
DN34156_c1_g1	Tumor rejection antigen	ETE71763	2.65E-22
DN34156_c1_g1	Tumor rejection antigen	ETE71763	3.08E-22
DN17557_c0_g1	angiotensin converting enzyme inhibitor and C-type natriuretic peptide precursor	BAP39952	1.22021E-21
DN31240_c0_g1	phospholipase D2	XP_013931058, XP_013931065, XP_013931072	2.32597E-20
DN34156_c1_g1	Tumor rejection antigen	ETE71763	3.08E-20
DN6327_c0_g1	phospholipase A1-like	XP_012058882	4.22087E-20
DN36832_c0_g1	phospholipase D3-like	XP_007442263	1.72759E-19
DN20497_c0_g1	predicted protein	ETE71763	2.24E-19
DN33112_c0_g1	Tumor rejection antigen	ETE71763	7.32E-19
DN18126_c0_g1	Tumor rejection antigen	ETE71763	1.20E-18
DN34177_c4_g1	Tumor rejection antigen	AAL49761	1.45E-16
DN30213_c0_g1	Tumor rejection antigen	XP_013907088	7.08E-15
DN33995_c1_g1	Tumor rejection antigen	AAL49761	1.26E-14
DN30213_c0_g1	Tumor rejection antigen	XP_013907088	1.99E-14
DN30213_c0_g1	Tumor rejection antigen	XP_013907088	4.15E-14
DN25432_c0_g2	Tumor rejection antigen	ETE71763	1.58E-13
DN13638_c0_g1	Tumor rejection antigen	ETE71763	2.55E-13
DN33420_c4_g1	testicular acid phosphatase homolog	XP_009029349, ESN93095	1.57E-12
DN31903_c0_g1	phospholipase A2 inhibitor subunit gamma B-like	XP_003222865, XP_008111986	4.75103E-12
DN31903_c0_g1	phospholipase A2 inhibitor subunit gamma B-like	ETE58602	6.76915E-12
DN31903_c0_g1	phospholipase A2 inhibitor subunit gamma B-like	ETE58602	7.53394E-12
DN31903_c0_g1	phospholipase A2 inhibitor subunit gamma B-like	ETE58602	9.93623E-12
DN30213_c0_g1	Tumor rejection antigen	XP_013907088	2.00E-11

Sequence name	Sequence desc.	Hit ACC	E-Value
DN32061_c4_g1	C-type lectin beta subunit	BAN82151	2.45991E-11
DN31903_c0_g1	phospholipase A2 inhibitor subunit gamma B-like	ETE58602	2.68186E-11
DN31903_c0_g1	phospholipase A2 inhibitor and Ly6/PLAUR domain-containing protein-like	ETE58602	2.88068E-11
DN31903_c0_g1	phospholipase A2 inhibitor subunit gamma B-like	XP_003222865, XP_008111986	2.89E-11
DN32061_c4_g1	C-type lectin beta subunit	BAN82151	4.3621E-11
DN30213_c0_g1	Tumor rejection antigen	XP_013907088	7.51E-11
DN54998_c0_g1	Tumor rejection antigen	XP_013918912	7.65E-11
DN33219_c0_g1	C-type lectin beta subunit	BAN82151	1.37793E-10
DN31903_c0_g1	phospholipase A2 inhibitor and Ly6/PLAUR domain-containing protein-like	XP_003222865, XP_008111986	2.02003E-10
DN30213_c0_g1	Tumor rejection antigen	ETE71763	1.73E-09
DN21562_c0_g1	Tumor rejection antigen	ETE71763	2.03E-09
DN34156_c1_g1	Tumor rejection antigen	ETE71763	2.17E-09
DN42779_c0_g1	protease inhibitor 3-like	ETE57997	3.52E-09
DN28257_c0_g1	Tumor rejection antigen	XP_013907088	4.17E-09
DN34156_c1_g1	Tumor rejection antigen	ETE71763	5.22E-09
DN34156_c1_g1	Tumor rejection antigen	ETE71763	6.61E-09
DN22201_c0_g1	phospholipase D2 isoform X2	XP_009429937	8.77447E-08
DN34156_c1_g1	Tumor rejection antigen	ETE71763	6.10E-07
DN16109_c0_g1	Tumor rejection antigen	XP_013929687	1.35E-06
DN16109_c0_g1	Tumor rejection antigen	XP_013930876	2.39E-06
DN32197_c1_g1	iron/zinc purple acid phosphatase-like protein isoform X4	XP_007994940	0.000367343
DN32197_c1_g1	iron/zinc purple acid phosphatase-like protein isoform X4	XP_007994940	0.000821927
DN32197_c1_g1	iron/zinc purple acid phosphatase-like protein isoform X4	XP_007994940	0.001070313
DN33110_c0_g1	disintegrin and metalloproteinase domain-containing protein 9-like	CEF83449	0.014924943
DN31682_c0_g1	disintegrin and metalloproteinase domain-containing protein 33-like	XP_013858797	0.02279166
DN18379_c0_g1	patatin-like phospholipase domain-containing protein 2 isoform X1	XP_006718328, XP_006718329	0.027671449
DN19983_c0_g1	Tumor rejection antigen	ETE71763	0.056433853
DN32832_c0_g1	serine proteinase stubble, putative	EGV99087	0.393062574
DN23441_c0_g1	serine protease ClpP	WP_002163536, EJR18705, KMP47499, KMP70058	5.43E-01
DN17799_c1_g1	trypsin-like serine protease with C-terminal PDZ domain	WP_051868916, KEZ34766	6.02E-01
DN33621_c0_g1	multifunctional 2',3'-cyclic-nucleotide 2'-phosphodiesterase/5'-nucleotidase/3'-nucleotidase	WP_040433522	1.057994751
DN63836_c0_g1	multifunctional 2',3'-cyclic-nucleotide 2'-phosphodiesterase/5'-nucleotidase/3'-nucleotidase	WP_017823160, EYT49386	1.865713683
DN22241_c0_g1	alkaline serine protease	EFX05394	3.19E+00
DN31737_c0_g1	adenosine-3'(2'),5'-bisphosphate nucleotidase	WP_027979931	3.343254722
DN30046_c0_g1	serine protease SohB	WP_008943465, EKE78208	4.45E+00
DN48637_c0_g1	phospholipase A I-like isoform X2	XP_009363541	6.034382776
DN26269_c0_g1	vascular non-inflammatory molecule 3-like	XP_010399820	6.472637417
DN33656_c2_g1	mannan-binding lectin serine protease 1 isoform X2	XP_006765916	6.77E+00
DN33598_c8_g1	MULTISPECIES: acid phosphatase	WP_030773682	8.288596109
DN17324_c0_g1	phospholipase A2, group IIE (predicted), isoform CRA_a	NP_001100166, EDL80892, EDL80893	8.962592422
DN76658_c0_g1	A disintegrin and metalloproteinase with thrombospondin motifs 2-like	XP_012687938	0.000166509
DN52719_c0_g1	disintegrin and metalloproteinase domain-containing protein 23 isoform X5	XP_011903056	0.050367496
DN28629_c0_g1	PREDICTED: endothelin-converting enzyme 2-like [Protobothrops mucrosquamatus]	XP_015679750.1	0.0
DN28629_c0_g1	PREDICTED: endothelin-converting enzyme 2-like [Protobothrops mucrosquamatus]	XP_015679750.1	0.0
DN28629_c0_g1	PREDICTED: endothelin-converting enzyme 2-like [Protobothrops mucrosquamatus]	XP_015679750.1	0.0
DN28901_c0_g1	XP_015669515.1 complement C1s subcomponent	XP_015669515.1	0.0
DN27812_c0_g1	Insulin-like growth factor-binding protein 6	ETE67110	1.00671E-48
DN24099_c0_g1	snake venom metalloprotease	A8QL59, ABN72547	1.03728E-48
DN27838_c0_g1	metalloproteinase VMP-II precursor	Q805F6, BAC55945	1.03965E-39
DN25382_c0_g1	Bradykinin-potentiating and C-type natriuretic peptides	Q6LEM5, BAA12879	1.04175E-24
DN31826_c0_g1	serine protease	Q8QG86, AAM09695	1.04433E-67
DN32310_c0_g1	Snake venom serine protease BthaTL; Short=SVSP	Q6IWF1, AAT40141	1.05064E-79
DN33023_c0_g1	vascular endothelial growth factor A isoform X1	P67860, BAD38847, ACN22044	1.0521E-110
DN32310_c0_g1	serine protease	Q8QG86, AAM09695	1.05984E-57
DN32061_c4_g1	C-type lectin beta subunit	BAN82151	1.08244E-12
DN32061_c4_g1	Snaclec GPIB-binding protein subunit alpha; Short=GPIb-BP subunit alpha	Q9PSM6, AAB47092	1.08379E-60
DN35331_c0_g1	vascular endothelial growth factor A-like isoform X1	ETE57464	1.1023E-40
DN54675_c0_g1	disintegrin and metalloproteinase domain-containing protein 15	XP_006031054	1.11579E-52
DN34115_c0_g1	C-type lectin precursor	ACS74991	1.12468E-75
DN27838_c0_g1	metalloproteinase precursor	Q9YI19, AAD02655	1.14744E-39
DN20394_c0_g1	disintegrin and metalloproteinase domain-containing protein 9-like	XP_007420853	1.1506E-159
DN32061_c4_g1	Snaclec bothrojaracin subunit beta; Short=BJC subunit beta; Flags: Precursor	Q56EB0, AAX68504	1.15937E-66
DN73869_c0_g1	A disintegrin and metalloproteinase with thrombospondin motifs 7	XP_013920681	1.16811E-40
DN30385_c1_g1	metalloproteinase	Q98UF9, AAG48931	1.17121E-47
DN8141_c0_g1	disintegrin and metalloproteinase domain 9,transcript variant 2	CBV37338	1.18871E-05
DN26273_c0_g1	disintegrin and metalloproteinase domain-containing protein 9-like	XP_007420853	1.1975E-127
DN20896_c0_g1	Snake venom metalloproteinase; Short=SVMP; Flags: Precursor	C9E1S0, ACV83935	1.20765E-49
DN16806_c0_g1	disintegrin and metalloproteinase domain-containing protein 9-like	XP_013912113	1.2603E-112
DN33554_c8_g1	Vascular apoptosis-inducing protein-like; Short=VAP-like	C5H5D2, ACI02287	1.2708E-133
DN32061_c4_g1	Snaclec GPIB-binding protein subunit beta; Short=GPIb-BP subunit beta	Q9PSM5, AAB47093	1.28109E-67
DN5343_c0_g1	venom allergen 3-like	XP_011165202	1.28503E-39
DN32061_c4_g1	Snaclec GPIB-binding protein subunit alpha; Short=GPIb-BP subunit alpha	Q9PSM6, AAB47092	1.33439E-62
DN34719_c0_g1	Stonustoxin subunit alpha	ETE56540	1.35055E-77
DN31826_c0_g1	Snake venom serine protease HS114; Short=SVSP; Flags: Precursor	Q5W959, BAD66928	1.36158E-66
DN23222_c0_g1	vascular endothelial growth factor precursor	Q90X23, AAK52103	1.36521E-79
DN33219_c0_g1	Snaclec GPIB-binding protein subunit beta; Short=GPIb-BP subunit beta	Q9PSM5, AAB47093	1.38341E-66
DN65603_c0_g1	A disintegrin and metalloproteinase with thrombospondin motifs 7	XP_007432084	1.38471E-43
DN30385_c1_g1	Vascular apoptosis-inducing protein-like; Short=VAP-like	C5H5D4, ACI02289	1.38938E-41
DN11890_c0_g1	disintegrin and metalloproteinase domain-containing protein 9	XP_007430419	1.39962E-91
DN27838_c0_g1	metalloproteinase precursor	Q9PVK9, AAD02654	1.42055E-39

Sequence name	Sequence desc.	Hit ACC	E-Value
DN32061_c4_g1	Snaclec bothrojaracin subunit alpha; Short=BJC subunit alpha; Flags: Precursor	Q56EB1, AAX68503	1.46641E-45
DN22973_c0_g1	cystatin precursor	J3RYX9	1.52435E-58
DN5077_c0_g1	C-type lectin beta subunit	BAN82151	1.53132E-07
DN30385_c1_g1	Vascular apoptosis-inducing protein-like; Short=VAP-like	C5H5D4, ACI02289	1.54537E-54
DN47520_c0_g1	A disintegrin and metalloproteinase with thrombospondin motifs 7	XP_013920681	1.57007E-75
DN10222_c0_g1	cystatin B	ETE69273	1.58168E-52
DN31826_c0_g1	Snake venom serine protease KN4 homolog; Short=SVSP; Flags: Precursor	Q71QJ4, AAQ02893	1.7186E-117
DN31959_c1_g1	metalloproteinase precursor	ABG26980	1.7366E-38
DN32310_c0_g1	venom thrombin-like enzyme	ABG26971	1.7456E-81
DN31365_c0_g1	Bradykinin-potentiating and C-type natriuretic peptides	P68515, AAM09690	1.77553E-78
DN27838_c0_g1	metalloproteinase precursor	Q9YI19, AAD02655	1.77631E-40
DN31959_c1_g1	group III snake venom metalloproteinase	ABG26980	1.77742E-43
DN31959_c1_g1	metalloproteinase VMP-II precursor	ABG26980	1.85852E-38
DN29729_c1_g1	Beta-fibrinogenase brevinase chain B	Q9PT51, CAB65936	1.87132E-75
DN33023_c0_g1	vascular endothelial growth factor	P67860, BAD38847, ACN22044	1.94035E-76
DN19402_c0_g1	cysteine-rich venom protein	BAP39957	1.9708E-105
DN5077_c0_g1	Snaclec trimecetin subunit beta; Flags: Precursor	BAN89424	1.98442E-32
DN25855_c0_g1	Vascular apoptosis-inducing protein-like; Short=VAP-like	C5H5D2, ACI02287	2.0088E-54
DN33113_c2_g1	Snake Venom Metalloproteinase Bap1 In Complex With A Peptidomimetic: Insights Into Inhibitor Binding	Q5XUW8, AAU47334	2.05019E-41
DN32061_c4_g1	C-type lectin beta subunit	BAN82151	2.15065E-11
DN32310_c0_g1	Snake venom serine protease BthaTL; Short=SVSP	Q6IWF1, AAT40141	2.22475E-79
DN30385_c1_g1	metalloproteinase	AEJ31992	2.22684E-52
DN33113_c2_g1	Metalloproteinase Bap1 In Complex With A Peptidomimetic: Insights Into Inhibitor Binding	Q5XUW8, AAU47334	2.2427E-77
DN33311_c1_g1	Basic phospholipase A2 homolog bothropstoxin-1	Q90249, AAO27453, AAP57527	2.24442E-72
DN48792_c0_g1	A disintegrin and metalloproteinase with thrombospondin motifs 10	KFQ07275	2.32993E-96
DN13765_c0_g1	serine protease 27-like	XP_013912131	2.34935E-77
DN28191_c0_g1	C-type lectin factor IX/X binding protein A subunit	BAN81990	2.36867E-36
DN32733_c1_g1	A disintegrin and metalloproteinase with thrombospondin motifs 1	XP_005880710	2.408273821
DN24099_c0_g2	disintegrin and metalloproteinase domain-containing protein 28	ETE62122	2.4319E-131
DN27838_c0_g1	metalloproteinase precursor	Q9YI19, AAD02655	2.43499E-39
DN33554_c7_g1	metalloproteinase VMP-III precursor	Q8UVG0, AAL47169	2.45594E-49
DN29448_c0_g1	A disintegrin and metalloproteinase with thrombospondin motifs 17	XP_005984541	2.458886643
DN27838_c0_g1	metalloproteinase VMP-II precursor	Q9PVK9, AAD02654	2.48007E-40
DN32406_c0_g1	cystatin C	ETE66625	2.5961E-66
DN33554_c8_g1	Snake venom metalloproteinase; Short=SVMP; Flags: Precursor	Q98UF9, AAG48931	2.6048E-105
DN33554_c8_g1	Snake venom metalloproteinase; Short=SVMP	Q0NZX9, ABD34830	2.61581E-55
DN10731_c0_g1	vascular endothelial growth factor	XP_007435124	2.6384E-28
DN48792_c0_g1	A disintegrin and metalloproteinase with thrombospondin motifs 6 isoform X3	XP_011541427	2.64395E-06
DN60211_c0_g1	nerve growth factor	Q9DEZ9, AAG30924	2.69417E-82
DN33554_c8_g1	Snake venom metalloproteinase; Short=SVMP; Flags: Precursor	Q98UF9, AAG48931	2.73427E-47
DN15576_c0_g1	serine protease HTRA2, mitochondrial	XP_013909049	2.76632E-52
DN1850_c0_g1	neoverrucotoxin subunit beta-like	ETE57257	2.79135E-59
DN37088_c0_g1	Serine protease 27	XP_013921690	2.8377E-46
DN32599_c0_g1	Disintegrin and metalloproteinase domain-containing protein 17	ETE70641	2.8654E-82
DN27601_c0_g1	disintegrin and metalloproteinase domain-containing protein 12	XP_005882567	2.87933E-14
DN33554_c8_g1	Snake venom metalloproteinase; Short=SVMP; Flags: Precursor	J3SDW8	2.9112E-141
DN21958_c0_g1	cysteine-rich venom protein	Q7ZTA0, AAO62994	2.9225E-122
DN15855_c0_g1	three finger toxin 1	ABG27004, ABZ89716	2.92556E-17
DN32310_c0_g1	Venombin A	P81661, AAB30013	2.9538E-81
DN33554_c7_g1	Zinc metalloproteinase/disintegrin	Q5XUW8, AAU47334	3.00823E-53
DN27838_c0_g1	metalloproteinase VMP-II precursor	Q9PVK9, AAD02654	3.10158E-39
DN31826_c0_g1	Snake venom serine protease HS114; Short=SVSP; Flags: Precursor	Q5W959, BAD66928	3.21572E-66
DN212_c0_g1	C-type lectin 6	AEJ31972	3.25971E-74
DN30554_c0_g1	phospholipase A2	Q90249, AAO27453, AAP57527	3.26208E-59
DN28431_c0_g1	disintegrin and metalloproteinase domain-containing protein 29 isoform X2	WP_049183553	3.354498026
DN32310_c0_g1	Venombin A	P81661, AAB30013	3.37371E-83
DN33315_c0_g1	disintegrin and metalloproteinase domain-containing protein 28	XP_013925301	3.4429E-154
DN33554_c8_g1	metalloproteinase isoform 7	ABG26984	3.50546E-69
DN33554_c8_g1	metalloproteinase isoform 7	ABG26984	3.51076E-61
DN33219_c0_g1	C-type lectin B subunit	BAP39929	3.57314E-33
DN40083_c0_g1	C-type lectin-like	ETE61126	3.6174E-56
DN26118_c0_g1	venom C-type lectin mannose binding isoform 4	ADF50042	3.67565E-30
DN34115_c0_g1	C-type lectin precursor	ACS74991	3.68151E-38
DN18229_c0_g1	C-type lectin precursor	ABD52882	3.76597E-97
DN33554_c8_g1	snake venom metalloprotease	C5H5D2, ACI02287	3.9014E-118
DN33554_c8_g1	snake venom metalloprotease	C5H5D2, ACI02287	3.9264E-123
DN33554_c8_g1	metalloproteinase 3	AEJ31986	3.92961E-84
DN17389_c0_g1	disintegrin and metalloproteinase domain-containing protein 15	XP_007436355	3.9473E-149
DN1659_c0_g1	venom allergen 3-like	XP_011871305	3.95384E-72
DN25855_c0_g1	Snake venom metalloproteinase 7; Short=SVMP; Flags: Precursor	F8S108, AEJ31990	3.98735E-71
DN31959_c1_g1	metalloproteinase VMP-II precursor	ABG26980	3.99865E-43
DN30385_c1_g1	metalloproteinase	AEJ31992	4.00311E-51
DN26008_c0_g1	serine protease 27	XP_013912133	4.1288E-68
DN31365_c0_g1	Bradykinin-potentiating and C-type natriuretic peptides	Q6LEM5, BAA12879	4.14101E-74
DN33554_c8_g1	group III snake venom metalloproteinase	ADO21502	4.18919E-59
DN33554_c8_g1	Snake venom metalloproteinase; Short=SVMP; Flags: Precursor	J3SDW8	4.2708E-143
DN32310_c0_g1	Snake venom serine protease; Short=SVSP; Flags: Precursor	P81824	4.39861E-50
DN1850_c0_g1	neoverrucotoxin subunit beta-like	ETE57257	4.44501E-68
DN12952_c2_g1	Metalloproteinase Bap1 In Complex With A Peptidomimetic: Insights Into Inhibitor Binding	O42138, AAC18911	4.44927E-36
DN26118_c0_g1	venom C-type lectin mannose binding isoform 3	ADF50042	4.54282E-31

Sequence name	Sequence desc.	Hit ACC	E-Value
DN34049_c13_g1	P-III metalloprotease	AEJ31986	4.59168E-68
DN32310_c0_g1	serine protease	ABG26971	4.59901E-78
DN63143_c0_g1	A disintegrin and metalloproteinase with thrombospondin motifs 10	XP_007433829	4.60932E-58
DN56777_c0_g1	serine protease 27	XP_013921690	4.68447E-40
DN29729_c1_g1	Beta-fibrinogenase brevinase chain B	Q9PT51, CAB65936	4.73513E-82
DN63578_c0_g1	venom C-type lectin mannose binding isoform 1	ABP94123	4.74227E-43
DN48162_c0_g1	C-type lectin 6	AEJ31972	4.81103E-55
DN31560_c0_g1	cysteine-rich secretory protein LCCL domain-containing 2	XP_007439730	4.97244E-86
DN32310_c0_g1	venom thrombin-like enzyme	ABG26971	5.12444E-79
DN33554_c8_g1	Vascular apoptosis-inducing protein-like; Short=VAP-like	C5H5D2, ACI02287	5.15696E-89
DN19329_c0_g1	A disintegrin and metalloproteinase with thrombospondin motifs 1	XP_007430227	5.1695E-97
DN37544_c0_g1	neoverrucotoxin subunit beta-like	ETE57679	5.17864E-57
DN41116_c0_g1	hyaluronidase-2	XP_013923960, XP_013923961	5.36817E-53
DN32061_c4_g1	Snaclec bothrojaracin subunit beta; Short=BJC subunit beta; Flags: Precursor	Q56EB0, AAX68504	5.53959E-51
DN25654_c0_g1	serine protease hepsin-like	XP_006278476	5.709425604
DN25680_c0_g1	ery1; Flags: Precursor	Q8UVG0, AAL47169	5.81463E-55
DN32602_c0_g1	Disintegrin and metalloproteinase domain-containing protein 10	ETE73040	5.88426E-79
DN15576_c0_g1	serine protease HTRA2, mitochondrial	XP_013909049	5.9863E-117
DN49754_c0_g1	disintegrin and metalloproteinase domain-containing protein 23	XP_013914319	6.02656E-84
DN32061_c4_g1	Snaclec GPIB-binding protein subunit beta; Short=GPIb-BP subunit beta	Q9PSM5, AAB47093	6.04573E-66
DN33554_c8_g1	Snake venom metalloproteinase 7; Short=SVMP; Flags: Precursor	F8S108, AEJ31990	6.14915E-56
DN33554_c8_g1	Zinc metalloproteinase-disintegrin VMP-II	C9E1R7, ACV83932	6.2181E-82
DN17557_c0_g1	Bradykinin-potentiating and C-type natriuretic peptides	Q6LEM5, BAA12879	6.30378E-31
DN44891_c0_g1	Coagulation factor V	ETE68574	6.39816E-74
DN212_c0_g1	C-type lectin beta subunit	BAN82151	6.62363E-18
DN32061_c4_g1	Snaclec GPIB-binding protein subunit alpha; Short=GPIb-BP subunit alpha	Q9PSM6, AAB47092	6.68146E-75
DN31826_c0_g1	Venombin A	P81661, AAB30013	6.89336E-91
DN73610_c0_g1	A disintegrin and metalloproteinase with thrombospondin motifs 10	XP_007433829	6.97153E-89
DN62927_c0_g1	C-type lectin	Q6QX33, AAS01426	7.01751E-87
DN33980_c0_g3	cystatin precursor	ETE59443	7.26197E-46
DN27551_c0_g1	translationally-controlled tumor protein	J3SFJ3, T1DKS4	7.34446E-95
DN26008_c0_g1	serine protease 33-like	XP_013912133	7.40568E-35
DN27838_c0_g1	metalloproteinase precursor	Q9YI19, AAD02655	7.41203E-39
DN39613_c0_g1	disintegrin and metalloproteinase domain-containing protein 19-like	XP_007425464	7.46831E-69
DN43182_c0_g1	Group III snake venom metalloproteinase	A8QL59, ABN72547	7.64815E-39
DN34040_c1_g1	serine protease	Q7T229, AAP42416	7.97243E-60
DN54672_c0_g1	nerve growth factor	Q90W38, AAG12169	8.1997E-140
DN21739_c0_g1	Vascular apoptosis-inducing protein-like; Short=VAP-like	C5H5D2, ACI02287	8.20881E-54
DN31959_c1_g1	group III snake venom metalloproteinase	ABG26980	8.26123E-39
DN36623_c0_g1	A disintegrin and metalloproteinase with thrombospondin motifs 7	XP_013920681	8.2828E-118
DN19402_c0_g1	cysteine-rich venom protein	BAP39957	8.3352E-125
DN34049_c16_g1	Vascular apoptosis-inducing protein; Short=VAP; Flags: Precursor	Q8AWI5, AAN39540	8.56558E-57
DN66114_c0_g1	vascular endothelial growth factor A-like isoform X1	XP_006129708	8.621321252
DN34617_c0_g1	A disintegrin and metalloproteinase with thrombospondin motifs 7	XP_007432084	8.91835E-79
DN33554_c8_g1	metalloproteinase VMP-III precursor	C9E1S0, ACV83935	8.94186E-90
DN27340_c0_g1	Snake venom serine protease 2A homolog; Short=SVSP 2A; Flags: Precursor	O13060, BAA19980	8.95767E-52
DN8141_c0_g1	disintegrin and metalloproteinase domain-containing protein 9	XP_007430419	8.9643E-144
DN18834_c0_g1	Veficolin-1; Flags: Precursor	ETE57381	8.97121E-83
DN7164_c0_g1	cysteine-rich secretory protein LCCL domain-containing 2	XP_007439730	8.97589E-54
DN31826_c0_g1	Snake venom serine protease KN4 homolog; Short=SVSP; Flags: Precursor	Q71QJ4, AAQ02893	9.1918E-118
DN19574_c0_g1	Vascular apoptosis-inducing protein-like; Short=VAP-like	C5H5D4, ACI02289	9.25784E-66
DN33554_c7_g1	metalloproteinase VMP-II precursor	Q9PVK9, AAD02654	9.36492E-50
DN32061_c4_g1	C-type lectin beta subunit	BAN82151	9.44031E-11
DN71367_c0_g1	C-type lectin A subunit	BAP39932	9.55186E-06
DN56541_c0_g1	Insulin-like growth factor-binding protein 4	ETE68665	9.87094E-75
DN32061_c4_g1	C-type lectin beta subunit	BAN82151	9.87849E-13
DN14583_c0_g1	translationally-controlled tumor protein	J3SFJ3, T1DKS4	9.89396E-65
DN33554_c8_g1	Snake venom metalloproteinase; Short=SVMP; Flags: Precursor	Q98UF9, AAG48931	9.9068E-102
DN35238_c0_g1	disintegrin and metalloproteinase domain-containing protein 9	XP_007430419	9.91773E-80
