# Supplementary material for: In-depth transcriptome reveals the potential biotechnological application of Bothrops jararaca venom gland
Source: J Venom Anim Toxins Incl Trop Dis. 2020 Oct 21;26:e20190058. doi: 10.1590/1678-9199-JVATITD-2019-0058 (PMC7579844; doi:10.1590/1678-9199-JVATITD-2019-0058)
Supplement: Additional file 14. [file 1678-9199-jvatitd-26-e20190058-s14.pdf]

J Venom Anim Toxins incl Trop Dis

https://doi.org/10.1590/1678-9199-JVATITD-2019-0058

Supplementary Material to "In-depth transcriptome reveals the potential biotechnological application of *Bothrops jararaca* venom gland"

Additional file 14. Toxins and inhibitors identified in the predicted proteome of *B. jararaca.*

Animal Toxins identified	Proteins counts	Percentage	Peptide signal
Metalloproteinases	112	21.0%	Yes
Tumor rejection antigen	60	11.2%	Yes
Serine protease	41	7.7%	Yes
Phospholipase A2	37	6.9%	Yes
C-type lectin	25	4.7%	Yes
5'-nucleotidase	23	4.3%	Yes
Phospholipase D type	20	3.7%	Yes
Phospholipase A2 inhibitor	18	3.4%	Yes
Acid phosphatase	16	3.0%	Yes
Ectonucleotide pyrophosphatase/phosphodiesterase	12	2.2%	Yes
Vascular endothelial growth factor	11	2.1%	Yes
Cysteine-rich proteins	9	1.7%	Not
Patatin-like phospholipase domain-containing protein 2	8	1.5%	Not
Insulin-like growth factor	6	1.1%	Not
Bradykinin-potentiating and C-type natriuretic peptides	6	1.1%	Not
Venom thrombin-like enzyme	5	0.9%	Not
Snaclec trimecetin subunit beta	5	0.9%	Not
Cystatin	4	0.7%	Yes
Endothelin-converting enzyme 2	4	0.7%	Not
Phospholipase B	4	0.7%	Yes
L-amino acid oxidase	4	0.7%	Yes
Acetylcholinesterase	4	0.7%	Not
Venom factor	4	0.7%	Not
ADAMTS-like protein 2	4	0.7%	Not
Phospholipase A1	3	0.6%	Not
Translationally-controlled tumor protein	3	0.6%	Not
Reticulocalbin-1	3	0.6%	Yes
Neoverrucotoxin	3	0.6%	Not
Trehalase	3	0.6%	Yes
ADAMTS-like protein 4	3	0.6%	Not
Mannan-binding lectin serine protease 2	3	0.6%	Yes

Animal Toxins identified	Proteins counts	Percentage	Peptide signal
relA-associated inhibitor	3	0.6%	Not
Reversion-inducing cysteine-rich protein with Kazal motifs	3	0.6%	Not
Mannan-binding lectin serine protease 2	3	0.6%	Yes
Peroxiredoxin 1	2	0.4%	Not
Coagulation factor V	2	0.4%	Not
Lysophospholipase	2	0.4%	Not
Pancreatic alpha-amylase	2	0.4%	Yes
Venom dipeptidylpeptidase IV	2	0.4%	Not
Glutaminyl-peptide cyclotransferase	2	0.4%	Not
Venom allergen 3-like	2	0.4%	Yes
Fibrinogenase brevinase	2	0.4%	Not
Stonustoxin subunit alpha	2	0.4%	Not
Nerve growth factor	2	0.4%	Yes
Snake venom metalloproteinase jararafibrase-2	2	0.4%	Not
Beta-fibrinogenase brevinase	2	0.4%	Not
Ribonuclease inhibitor	2	0.4%	Not
Cystatin B	1	0.2%	Yes
Cystatin C	1	0.2%	Not
Coagulation factor VIII	1	0.2%	Not
Calglandulin-like	1	0.2%	Not
Hyaluronidase	1	0.2%	Yes
Hyaluronidase-2	1	0.2%	Not
Veficolin-1	1	0.2%	Not
Three finger toxin 1	1	0.2%	Yes
Reticulocalbin-2	1	0.2%	Yes
Peroxiredoxin 3	1	0.2%	Not
Peroxiredoxin 4	1	0.2%	Not
WAP four-disulfide core domain protein 2 (putative Waprins)	1	0.2%	Yes
Cathepsin O	1	0.2%	Yes
Cathepsin L1	1	0.2%	Not
Neurotrophin-3	1	0.2%	Yes
Ryncolin-1-like	1	0.2%	Not
Plasma protease C1 inhibitor	1	0.2%	Yes
Protein phosphatase inhibitor 2	1	0.2%	Not
kunitz-type protease inhibitor 1	1	0.2%	Yes
kunitz-type protease inhibitor 2	1	0.2%	Yes
kunitz-type protease inhibitor 4	1	0.2%	Yes
Protease inhibitor 3-like	1	0.2%	Not

Animal Toxins identified	Proteins counts	Percentage	Peptide signal
**metalloproteinase inhibitor 1**	1	0.2%	Yes
**metalloproteinase inhibitor 2**	1	0.2%	Yes
**Metalloproteinase inhibitor 3**	1	0.2%	Yes
**ATPase inhibitor, mitochondrial**	1	0.2%	Not
**Protein phosphatase inhibitor 2**	1	0.2%	Not
**Plasma protease C1 inhibitor**	1	0.2%	Yes
**Protease inhibitor 3-like**	1	0.2%	Not
**Putative protein-glutamate O-methyltransferase**	1	0.2%	Not
**Mannan-binding lectin serine protease 1**	1	0.2%	Not
**Endothelin-converting enzyme 1**	1	0.2%	Not
**Peroxiredoxin 3**	1	0.2%	Not
**Peroxiredoxin 4**	1	0.2%	Not
**Peroxiredoxin 5**	1	0.2%	Not
**Peroxiredoxin 6**	1	0.2%	Yes
**TOTAL**	**534**	**100,0%**	
