# Supplementary material for: In-depth transcriptome reveals the potential biotechnological application of Bothrops jararaca venom gland
Source: J Venom Anim Toxins Incl Trop Dis. 2020 Oct 21;26:e20190058. doi: 10.1590/1678-9199-JVATITD-2019-0058 (PMC7579844; doi:10.1590/1678-9199-JVATITD-2019-0058)
Supplement: Additional file 15. [file 1678-9199-jvatitd-26-e20190058-s15.pdf]

J Venom Anim Toxins incl Trop Dis

https://doi.org/10.1590/1678-9199-JVATITD-2019-0058

Supplementary Material to "In-depth transcriptome reveals the potential biotechnological application of *Bothrops jararaca* venom gland"

Additional file 15. Function of toxins and accessory proteins identified in the predicted proteome of *B. jararaca.*

#qseqid	sseqid	pident	length	evalue	hit_descr
**DN31772_c0_g1**	sp|F8S101|PLB_CROAD	96.38	553	0.0	Phospholipase B OS=Crotalus adamanteus OX=8729 PE=1 SV=1
**DN34088_c1_g1**	sp|O93523|VM3BP_BOTJA	86.56	610	0.0	Zinc metalloproteinase-disintegrin-like bothropasin OS=Bothrops jararaca OX=8724 PE=1 SV=2
**DN34088_c1_g1**	sp|Q98SP2|VM2J2_BOTJA	92.47	478	0.0	Zinc metalloproteinase/disintegrin OS=Bothrops jararaca OX=8724 PE=1 SV=1
**DN34028_c0_g1**	sp|Q01833|CO3_NAJNA	86.19	1651	0.0	Complement C3 OS=Naja naja OX=35670 GN=C3 PE=2 SV=1
**DN11926_c0_g1**	sp|J3SDX8|LICH_CROAD	93.75	400	0.0	Putative lysosomal acid lipase/cholesteryl ester hydrolase OS=Crotalus adamanteus OX=8729 PE=2 SV=1
**DN32044_c0_g1**	sp|J3SEZ3|PDE1_CROAD	95.31	852	0.0	Venom phosphodiesterase 1 OS=Crotalus adamanteus OX=8729 PE=1 SV=2
**DN34088_c1_g1**	sp|Q98SP2|VM2J2_BOTJA	84.20	481	0.0	Zinc metalloproteinase/disintegrin OS=Bothrops jararaca OX=8724 PE=1 SV=1
**DN29580_c0_g1**	sp|F8S0Z7|V5NTD_CROAD	95.42	590	0.0	Snake venom 5'-nucleotidase OS=Crotalus adamanteus OX=8729 PE=1 SV=2
**DN32234_c0_g1**	sp|B5AR80|OXLA_BOTPA	98.41	502	0.0	L-amino-acid oxidase (Fragment) OS=Bothrops pauloensis OX=1042543 PE=1 SV=1
**DN31322_c0_g1**	sp|B5AR80|OXLA_BOTPA	98.61	502	0.0	L-amino-acid oxidase (Fragment) OS=Bothrops pauloensis OX=1042543 PE=1 SV=1
**DN28629_c0_g1**	sp|F1N476|ECE2_BOVIN	76.43	768	0.0	Endothelin-converting enzyme 2 OS=Bos taurus OX=9913 GN=ECE2 PE=2 SV=1
**DN28629_c0_g1**	sp|F1N476|ECE2_BOVIN	73.44	768	0.0	Endothelin-converting enzyme 2 OS=Bos taurus OX=9913 GN=ECE2 PE=2 SV=1
**DN28629_c0_g1**	sp|F1N476|ECE2_BOVIN	73.44	768	0.0	Endothelin-converting enzyme 2 OS=Bos taurus OX=9913 GN=ECE2 PE=2 SV=1
**DN28629_c0_g1**	sp|F1N476|ECE2_BOVIN	76.43	768	0.0	Endothelin-converting enzyme 2 OS=Bos taurus OX=9913 GN=ECE2 PE=2 SV=1
**DN32861_c0_g1**	sp|Q9YIB5|QPCT_BOTJA	100.00	361	0.0	Glutaminyl-peptide cyclotransferase OS=Bothrops jararaca OX=8724 GN=QPCT PE=2 SV=1
**DN28218_c0_g1**	sp|P78536|ADA17_HUMAN	75.27	651	0.0	Disintegrin and metalloproteinase domain-containing protein 17 OS=Homo sapiens OX=9606 GN=ADAM17 PE=1 SV=1
**DN31047_c0_g1**	sp|J3RZ81|LIPE_CROAD	96.97	429	0.0	Putative endothelial lipase OS=Crotalus adamanteus OX=8729 PE=2 SV=1
**DN34049_c16_g1**	sp|Q8QG88|VM36A_BOTIN	98.10	526	0.0	Zinc metalloproteinase-disintegrin-like BITM06A OS=Bothrops insularis OX=8723 PE=2 SV=1
**DN30958_c0_g1**	sp|P42892|ECE1_HUMAN	75.21	601	0.0	Endothelin-converting enzyme 1 OS=Homo sapiens OX=9606 GN=ECE1 PE=1 SV=2
**DN34049_c16_g1**	sp|P30431|VM3JA_BOTJA	83.48	466	0.0	Zinc metalloproteinase-disintegrin-like jararhagin (Fragment) OS=Bothrops jararaca OX=8724 PE=1 SV=1
**DN34049_c16_g1**	sp|Q8QG88|VM36A_BOTIN	98.10	526	0.0	Zinc metalloproteinase-disintegrin-like BITM06A OS=Bothrops insularis OX=8723 PE=2 SV=1
**DN30958_c0_g1**	sp|P42892|ECE1_HUMAN	75.21	601	0.0	Endothelin-converting enzyme 1 OS=Homo sapiens OX=9606 GN=ECE1 PE=1 SV=2
**DN34049_c16_g1**	sp|P30431|VM3JA_BOTJA	83.48	466	0.0	Zinc metalloproteinase-disintegrin-like jararhagin (Fragment) OS=Bothrops jararaca OX=8724 PE=1 SV=1
**DN28767_c0_g1**	sp|J3S9D9|RCN2V_CROAD	98.59	284	3.00E-147	Reticulocalbin-2 OS=Crotalus adamanteus OX=8729 PE=1 SV=1
**DN54672_c0_g1**	sp|Q90W38|NGFV_BOTJR	99.17	241	5.00E-143	Venom nerve growth factor OS=Bothrops jararacussu OX=8726 GN=NGF PE=2 SV=1
**DN33061_c11_g1**	sp|B2D0J4|VDPP4_APIME	34.94	747	2.00E-127	Venom dipeptidyl peptidase 4 OS=Apis mellifera OX=7460 PE=1 SV=1
**DN19402_c0_g1**	sp|Q7ZTA0|CRVP_AGKPI	87.92	240	1.00E-123	Cysteine-rich venom protein piscivorin OS=Agkistrodon piscivorus piscivorus OX=8716 PE=1 SV=1
**DN21958_c0_g1**	sp|Q7ZTA0|CRVP_AGKPI	87.92	240	4.00E-123	Cysteine-rich venom protein piscivorin OS=Agkistrodon piscivorus piscivorus OX=8716 PE=1 SV=1
**DN31826_c0_g1**	sp|Q71QJ4|VSP04_TRIST	80.31	259	1.00E-115	Snake venom serine protease homolog KN4 OS=Trimeresurus stejnegeri OX=39682 PE=2 SV=1
**DN31826_c0_g1**	sp|Q71QJ4|VSP04_TRIST	79.92	259	3.00E-115	Snake venom serine protease homolog KN4 OS=Trimeresurus stejnegeri OX=39682 PE=2 SV=1
**DN33023_c0_g1**	sp|C0K3N4|VEGFA_AGKPI	96.88	192	3.00E-114	Vascular endothelial growth factor A OS=Agkistrodon piscivorus piscivorus OX=8716 PE=2 SV=1
**DN33023_c0_g1**	sp|P67860|VEGFA_PROFL	97.22	216	4.00E-114	Vascular endothelial growth factor A OS=Protobothrops flavoviridis OX=88087 PE=1 SV=1
**DN28278_c2_g1**	sp|A3QVN9|HYAL1_BITAR	47.22	396	9.00E-108	Hyaluronidase-1 OS=Bitis arietans OX=8692 PE=2 SV=1
**DN28278_c2_g1**	sp|A3QVN9|HYAL1_BITAR	47.22	396	9.00E-108	Hyaluronidase-1 OS=Bitis arietans OX=8692 PE=2 SV=1
**DN30499_c0_g1**	sp|B2BS84|VKT_AUSLA	84.92	252	9.00E-107	Putative Kunitz-type serine protease inhibitor OS=Austrelaps labialis OX=471292 PE=2 SV=1
**DN19402_c0_g1**	sp|Q7ZTA0|CRVP_AGKPI	90.45	199	1.00E-102	Cysteine-rich venom protein piscivorin OS=Agkistrodon piscivorus piscivorus OX=8716 PE=1 SV=1
**DN19402_c0_g1**	sp|Q7ZTA0|CRVP_AGKPI	90.45	199	1.00E-102	Cysteine-rich venom protein piscivorin OS=Agkistrodon piscivorus piscivorus OX=8716 PE=1 SV=1
**DN27551_c0_g1**	sp|J3SFJ3|TCTP_CROAD	98.84	172	1.00E-97	Translationally-controlled tumor protein homolog OS=Crotalus adamanteus OX=8729 PE=2 SV=1
**DN27551_c0_g1**	sp|J3SFJ3|TCTP_CROAD	98.84	172	1.00E-97	Translationally-controlled tumor protein homolog OS=Crotalus adamanteus OX=8729 PE=2 SV=1
**DN33592_c1_g1**	sp|Q90YA8|QPCT_GLOBL	53.14	318	1.00E-96	Glutaminyl-peptide cyclotransferase OS=Gloydius blomhoffii OX=242054 GN=QPCT PE=2 SV=1
**DN33592_c1_g1**	sp|Q90YA8|QPCT_GLOBL	53.14	318	1.00E-96	Glutaminyl-peptide cyclotransferase OS=Gloydius blomhoffii OX=242054 GN=QPCT PE=2 SV=1
**DN31826_c0_g1**	sp|P81661|VSPA_BOTJA	80.00	200	5.00E-89	Thrombin-like enzyme bothrombin OS=Bothrops jararaca OX=8724 PE=1 SV=1
**DN31826_c0_g1**	sp|P81661|VSPA_BOTJA	80.00	200	5.00E-89	Thrombin-like enzyme bothrombin OS=Bothrops jararaca OX=8724 PE=1 SV=1
**DN62927_c0_g1**	sp|Q6QX33|LECG_BOTIN	98.10	158	3.00E-88	C-type lectin BiL OS=Bothrops insularis OX=8723 PE=1 SV=1
**DN33311_c1_g1**	sp|Q90249|PA2B1_BOTJR	99.27	137	2.00E-83	Basic phospholipase A2 homolog bothropstoxin-1 OS=Bothrops jararacussu OX=8726 PE=1 SV=3
**DN21580_c0_g1**	sp|Q90X23|TXVE_BOTJA	100.00	135	2.00E-80	Snake venom vascular endothelial growth factor toxin OS=Bothrops jararaca OX=8724 PE=1 SV=1
**DN23222_c0_g1**	sp|Q90X23|TXVE_BOTJA	100.00	135	4.00E-80	Snake venom vascular endothelial growth factor toxin OS=Bothrops jararaca OX=8724 PE=1 SV=1
**DN32061_c4_g1**	sp|Q9PSM5|SL1B_BOTJA	100.00	122	2.00E-76	Snaclec GPIB-binding protein subunit beta OS=Bothrops jararaca OX=8724 PE=1 SV=1
**DN32061_c4_g1**	sp|Q9PSM5|SL1B_BOTJA	100.00	122	2.00E-76	Snaclec GPIB-binding protein subunit beta OS=Bothrops jararaca OX=8724 PE=1 SV=1
**DN33219_c0_g1**	sp|Q9PSM5|SL1B_BOTJA	98.36	122	2.00E-75	Snaclec GPIB-binding protein subunit beta OS=Bothrops jararaca OX=8724 PE=1 SV=1
**DN32061_c4_g1**	sp|Q9PSM5|SL1B_BOTJA	98.36	122	3.00E-75	Snaclec GPIB-binding protein subunit beta OS=Bothrops jararaca OX=8724 PE=1 SV=1
**DN32061_c4_g1**	sp|Q9PSM6|SL1A_BOTJA	97.18	142	2.00E-74	Snaclec GPIB-binding protein subunit alpha OS=Bothrops jararaca OX=8724 PE=1 SV=1
**DN32061_c4_g1**	sp|Q9PSM6|SL1A_BOTJA	97.18	142	2.00E-74	Snaclec GPIB-binding protein subunit alpha OS=Bothrops jararaca OX=8724 PE=1 SV=1
**DN32061_c4_g1**	sp|Q9PSM6|SL1A_BOTJA	97.18	142	2.00E-74	Snaclec GPIB-binding protein subunit alpha OS=Bothrops jararaca OX=8724 PE=1 SV=1
**DN33311_c1_g1**	sp|Q2HZ28|PA2A_BOTER	92.86	140	1.00E-69	Acidic phospholipase A2 BE-I-PLA2 OS=Bothrops erythromelas OX=44710 PE=1 SV=1
**DN31371_c0_g1**	sp|Q2HZ28|PA2A_BOTER	92.81	139	4.00E-69	Acidic phospholipase A2 BE-I-PLA2 OS=Bothrops erythromelas OX=44710 PE=1 SV=1
**DN30554_c0_g1**	sp|Q90249|PA2B1_BOTJR	97.46	118	1.00E-68	Basic phospholipase A2 homolog bothropstoxin-1 OS=Bothrops jararacussu OX=8726 PE=1 SV=3
**DN30554_c0_g1**	sp|Q90249|PA2B1_BOTJR	97.46	118	1.00E-68	Basic phospholipase A2 homolog bothropstoxin-1 OS=Bothrops jararacussu OX=8726 PE=1 SV=3
**DN30554_c0_g1**	sp|Q90249|PA2B1_BOTJR	97.46	118	1.00E-68	Basic phospholipase A2 homolog bothropstoxin-1 OS=Bothrops jararacussu OX=8726 PE=1 SV=3
**DN30554_c0_g1**	sp|Q90249|PA2B1_BOTJR	97.46	118	1.00E-68	Basic phospholipase A2 homolog bothropstoxin-1 OS=Bothrops jararacussu OX=8726 PE=1 SV=3
**DN34270_c0_g1**	sp|Q8MMH3|ARMT1_PIMHY	35.11	393	8.00E-68	Putative protein-glutamate O-methyltransferase OS=Pimpla hypochondriaca OX=135724 GN=vpr2 PE=1 SV=1
**DN18185_c0_g1**	sp|J3S9D9|RCN2V_CROAD	43.27	275	2.00E-66	Reticulocalbin-2 OS=Crotalus adamanteus OX=8729 PE=1 SV=1
**DN18185_c0_g1**	sp|J3S9D9|RCN2V_CROAD	43.27	275	2.00E-66	Reticulocalbin-2 OS=Crotalus adamanteus OX=8729 PE=1 SV=1
**DN33311_c1_g1**	sp|Q2HZ28|PA2A_BOTER	88.57	140	1.00E-65	Acidic phospholipase A2 BE-I-PLA2 OS=Bothrops erythromelas OX=44710 PE=1 SV=1
**DN31371_c0_g1**	sp|Q2HZ28|PA2A_BOTER	88.49	139	3.00E-65	Acidic phospholipase A2 BE-I-PLA2 OS=Bothrops erythromelas OX=44710 PE=1 SV=1
**DN18185_c0_g1**	sp|J3S9D9|RCN2V_CROAD	43.64	275	3.00E-65	Reticulocalbin-2 OS=Crotalus adamanteus OX=8729 PE=1 SV=1

#qseqid	sseqid	pident	length	evalue	hit_descr
**DN18185_c0_g1**	sp|J3S9D9|RCN2V_CROAD	43.64	275	3.00E-65	Reticulocalbin-2 OS=Crotalus adamanteus OX=8729 PE=1 SV=1
**DN32061_c4_g1**	sp|Q56EB0|SLAB_BOTJA	85.91	149	1.00E-63	Snaclec bothrojaracin subunit beta OS=Bothrops jararaca OX=8724 PE=1 SV=1
**DN33311_c1_g1**	sp|Q2HZ28|PA2A_BOTER	87.86	140	3.00E-63	Acidic phospholipase A2 BE-I-PLA2 OS=Bothrops erythromelas OX=44710 PE=1 SV=1
**DN33219_c0_g1**	sp|Q9DEF8|SLAB_DEIAC	79.45	146	6.00E-63	Snaclec anticoagulant protein subunit B OS=Deinagkistrodon acutus OX=36307 PE=1 SV=1
**DN33311_c1_g1**	sp|Q8QG87|PA2A_BOTIN	87.14	140	5.00E-62	Acidic phospholipase A2 BITP01A OS=Bothrops insularis OX=8723 PE=1 SV=1
**DN32061_c4_g1**	sp|Q9DEF8|SLAB_DEIAC	76.98	126	5.00E-62	Snaclec anticoagulant protein subunit B OS=Deinagkistrodon acutus OX=36307 PE=1 SV=1
**DN32061_c4_g1**	sp|Q9DEF8|SLAB_DEIAC	76.98	126	5.00E-62	Snaclec anticoagulant protein subunit B OS=Deinagkistrodon acutus OX=36307 PE=1 SV=1
**DN32406_c0_g1**	sp|J3SE80|CYT2_CROAD	92.50	120	6.00E-62	Cystatin-2 OS=Crotalus adamanteus OX=8729 PE=2 SV=1
**DN32406_c0_g1**	sp|J3SE80|CYT2_CROAD	92.50	120	6.00E-62	Cystatin-2 OS=Crotalus adamanteus OX=8729 PE=2 SV=1
**DN33311_c1_g1**	sp|Q8QG87|PA2A_BOTIN	86.43	140	1.00E-61	Acidic phospholipase A2 BITP01A OS=Bothrops insularis OX=8723 PE=1 SV=1
**DN32061_c4_g1**	sp|Q9PSM6|SL1A_BOTJA	85.11	141	2.00E-61	Snaclec GPIB-binding protein subunit alpha OS=Bothrops jararaca OX=8724 PE=1 SV=1
**DN32061_c4_g1**	sp|Q9PSM6|SL1A_BOTJA	85.11	141	2.00E-61	Snaclec GPIB-binding protein subunit alpha OS=Bothrops jararaca OX=8724 PE=1 SV=1
**DN32061_c4_g1**	sp|Q9PSM6|SL1A_BOTJA	84.40	141	5.00E-60	Snaclec GPIB-binding protein subunit alpha OS=Bothrops jararaca OX=8724 PE=1 SV=1
**DN33219_c0_g1**	sp|Q9DEF8|SLAB_DEIAC	76.55	145	6.00E-60	Snaclec anticoagulant protein subunit B OS=Deinagkistrodon acutus OX=36307 PE=1 SV=1
**DN33219_c0_g1**	sp|Q9DEF8|SLAB_DEIAC	76.55	145	6.00E-60	Snaclec anticoagulant protein subunit B OS=Deinagkistrodon acutus OX=36307 PE=1 SV=1
**DN32061_c4_g1**	sp|Q9DEF8|SLAB_DEIAC	76.55	145	2.00E-59	Snaclec anticoagulant protein subunit B OS=Deinagkistrodon acutus OX=36307 PE=1 SV=1
**DN32061_c4_g1**	sp|Q9DEF8|SLAB_DEIAC	76.55	145	2.00E-59	Snaclec anticoagulant protein subunit B OS=Deinagkistrodon acutus OX=36307 PE=1 SV=1
**DN32061_c4_g1**	sp|Q9PSM5|SL1B_BOTJA	76.23	122	2.00E-58	Snaclec GPIB-binding protein subunit beta OS=Bothrops jararaca OX=8724 PE=1 SV=1
**DN32061_c4_g1**	sp|Q9DEF8|SLAB_DEIAC	73.60	125	3.00E-58	Snaclec anticoagulant protein subunit B OS=Deinagkistrodon acutus OX=36307 PE=1 SV=1
**DN32061_c4_g1**	sp|Q9DEF8|SLAB_DEIAC	73.60	125	3.00E-58	Snaclec anticoagulant protein subunit B OS=Deinagkistrodon acutus OX=36307 PE=1 SV=1
**DN33311_c1_g1**	sp|Q8QG87|PA2A_BOTIN	82.86	140	8.00E-57	Acidic phospholipase A2 BITP01A OS=Bothrops insularis OX=8723 PE=1 SV=1
**DN20299_c0_g1**	sp|A7X4K1|WAP1_PHIOL	75.37	134	9.00E-57	Waprin-Phi1 OS=Philodryas olfersii OX=120305 PE=2 SV=1
**DN19230_c0_g1**	sp|J3S9D9|RCN2V_CROAD	39.68	252	6.00E-56	Reticulocalbin-2 OS=Crotalus adamanteus OX=8729 PE=1 SV=1
**DN19230_c0_g1**	sp|J3S9D9|RCN2V_CROAD	39.68	252	6.00E-56	Reticulocalbin-2 OS=Crotalus adamanteus OX=8729 PE=1 SV=1
**DN32587_c0_g1**	sp|J3S9D9|RCN2V_CROAD	40.86	279	2.00E-55	Reticulocalbin-2 OS=Crotalus adamanteus OX=8729 PE=1 SV=1
**DN32587_c0_g1**	sp|J3S9D9|RCN2V_CROAD	40.86	279	2.00E-55	Reticulocalbin-2 OS=Crotalus adamanteus OX=8729 PE=1 SV=1
**DN22973_c0_g1**	sp|J3RYX9|CYT1_CROAD	87.77	139	3.00E-51	Cystatin-1 OS=Crotalus adamanteus OX=8729 PE=2 SV=1
**DN33075_c1_g1**	sp|P16354|PA23_HELSU	47.66	128	1.00E-38	Phospholipase A2 isozymes PA3A/PA3B/PA5 OS=Heloderma suspectum OX=8554 PE=1 SV=3
**DN33075_c1_g1**	sp|P16354|PA23_HELSU	47.66	128	1.00E-38	Phospholipase A2 isozymes PA3A/PA3B/PA5 OS=Heloderma suspectum OX=8554 PE=1 SV=3
**DN44012_c0_g1**	sp|G3LU44|TCTP_LOXIN	44.94	158	2.00E-36	Translationally-controlled tumor protein homolog OS=Loxosceles intermedia OX=58218 PE=2 SV=1
**DN40083_c0_g1**	sp|Q6QX33|LECG_BOTIN	42.41	158	4.00E-35	C-type lectin BiL OS=Bothrops insularis OX=8723 PE=1 SV=1
**DN26118_c0_g1**	sp|Q8JIV8|SL_DEIAC	46.15	143	1.00E-34	Snaclec clone 2100755 OS=Deinagkistrodon acutus OX=36307 PE=2 SV=1
**DN26118_c0_g1**	sp|Q8JIV8|SL_DEIAC	46.15	143	1.00E-34	Snaclec clone 2100755 OS=Deinagkistrodon acutus OX=36307 PE=2 SV=1
**DN30398_c0_g1**	sp|Q5BLY5|ACPH1_APIME	27.20	364	2.00E-33	Venom acid phosphatase Acph-1 OS=Apis mellifera OX=7460 PE=1 SV=1
**DN5343_c0_g1**	sp|P0DSI3|VA3_DINQU	40.00	210	3.00E-33	Venom allergen 3 homolog OS=Dinoponera quadriceps OX=609295 PE=2 SV=1
**DN5343_c0_g1**	sp|P0DSI3|VA3_DINQU	40.00	210	3.00E-33	Venom allergen 3 homolog OS=Dinoponera quadriceps OX=609295 PE=2 SV=1
**DN26118_c0_g1**	sp|A7X3Z7|LECM2_ERYPO	46.10	141	1.00E-32	C-type lectin lectoxin-Lio2 OS=Erythrolamprus poecilogyrus OX=338838 PE=2 SV=1
**DN26118_c0_g1**	sp|A7X3Z7|LECM2_ERYPO	46.10	141	1.00E-32	C-type lectin lectoxin-Lio2 OS=Erythrolamprus poecilogyrus OX=338838 PE=2 SV=1
**DN20749_c0_g1**	sp|Q9TXD8|ISOHC_AGEAP	38.43	216	2.00E-32	Venom peptide isomerase heavy chain OS=Agelenopsis aperta OX=6908 PE=1 SV=1
**DN21627_c0_g1**	sp|Q9BMK4|PA2_APICC	42.86	133	4.00E-29	Phospholipase A2 OS=Apis cerana cerana OX=94128 PE=2 SV=1
